# Identification of highly selective type II kinase inhibitors with chiral peptidomimetic tails

**DOI:** 10.1080/14756366.2022.2068148

**Published:** 2022-04-28

**Authors:** Seo-Jung Han, Jae Eun Jung, Do Hee Oh, Minsup Kim, Jae-Min Kim, Kyung-Sook Chung, Hee-Soo Han, Jeong-Hun Lee, Kyung-Tae Lee, Hee Jin Jeong, In Ho Park, Eunkyeong Jeon, Jeon-Soo Shin, Dongkeun Hwang, Art E. Cho, Duck-Hyung Lee, Taebo Sim

**Affiliations:** a Chemical Kinomics Research Center, Korea Institute of Science and Technology, Seoul, Republic of Korea; b Division of Bio-Medical Science & Technology, KIST School, UST, Seoul, Republic of Korea; c Department of Chemistry, Sogang University, Seoul, Republic of Korea; d Drug Discovery Institute, inCerebro Co., Ltd., Seoul, Republic of Korea; e Department of Pharmaceutical Biochemistry, College of Pharmacy, Kyung Hee University, Seoul, Republic of Korea; f Department of Biomedical and Pharmaceutical Sciences, Graduate School, Kyung Hee University, Seoul, Republic of Korea; g Department of Life and Nanopharmaceutical Sciences, College of Pharmacy, Kyung Hee University, Seoul, Republic of Korea; h Severance Biomedical Science Institute, Yonsei University College of Medicine, Seoul, Republic of Korea; i Institute of Immunology and Immunological Diseases, Yonsei University College of Medicine, Seoul, Republic of Korea; j Department of Microbiology, Yonsei University College of Medicine, Seoul, Republic of Korea; k Department of Bioinformatics, Korea University, Sejong, Republic of Korea

**Keywords:** Type-II kinase, Lck, DSS-induced colitis

## Abstract

Identification of highly selective type II kinase inhibitors is described. Two different chiral peptidomimetic scaffolds were introduced on the tail region of non-selective type II kinase inhibitor GNF-7 to enhance the selectivity. Kinome-wide selectivity profiling analysis showed that type II kinase inhibitor **7a** potently inhibited Lck kinase with great selectivity (IC_50_ of 23.0 nM). It was found that **7a** and its derivatives possessed high selectivity for Lck over even structurally conserved all Src family kinases. We also observed that **7a** inhibited Lck activation in Jurkat T cells. Moreover, **7a** was found to alleviate clinical symptoms in DSS-induced colitis mice. This study provides a novel insight into the design of selective type II kinase inhibitors by adopting chiral peptidomimetic moieties on the tail region.

## 1. Introduction

Most signal transduction processes are mediated through a phosphotransfer reactions catalysed by kinases. However, overexpression or mutation of kinases causes tumour cell proliferation and survival. Therefore, kinases are pursued as invaluable targets and a tremendous amount of effort has been devoted towards the discovery of small molecular kinase inhibitors for the treatment of cancer for decades^1^. Small molecule kinase inhibitors have been classified by binding modes with protein kinases. Type I inhibitors are the most commonly encountered and occupy the ATP-binding site of the active conformation of kinases (i.e. DFG-in conformation). In contrast to type I inhibitors, type II kinase inhibitors recognise the ATP-binding pocket of inactive DFG-out conformation of kinase proteins. Besides ATP competitive inhibitors (e.g. type I, type II), allosteric and covalent inhibitors have also been investigated^1–4^. Although a significant number of small molecular kinase inhibitors have been developed, discovery of selective kinase inhibitors remains challenging. The rationale behind this is that the structure of ATP-binding site in all of the kinase proteins is highly conserved^1^. In addition, designing an allosteric kinase inhibitor, which is the most selective inhibitor, is difficult since it highly relies on an empirical exercise^2a^. Although selective kinases have been uncovered using subtle 3-dimensional structural differences among the kinases^5^, discovery of highly selective kinase inhibitors still remains a largely unmet challenge. Type II kinase inhibitors were anticipated to be more selective compared to type I kinase inhibitors in the early phase since the hydrophobic pocket generated by the DFG-out conformation is not quite conserved in contrast to ATP binding pocket and DFG-out conformations are more dynamic. However, type II kinase inhibitors, developed to date have been proven to be largely less selective than type I kinase inhibitors.^2a^

**Figure 1.**
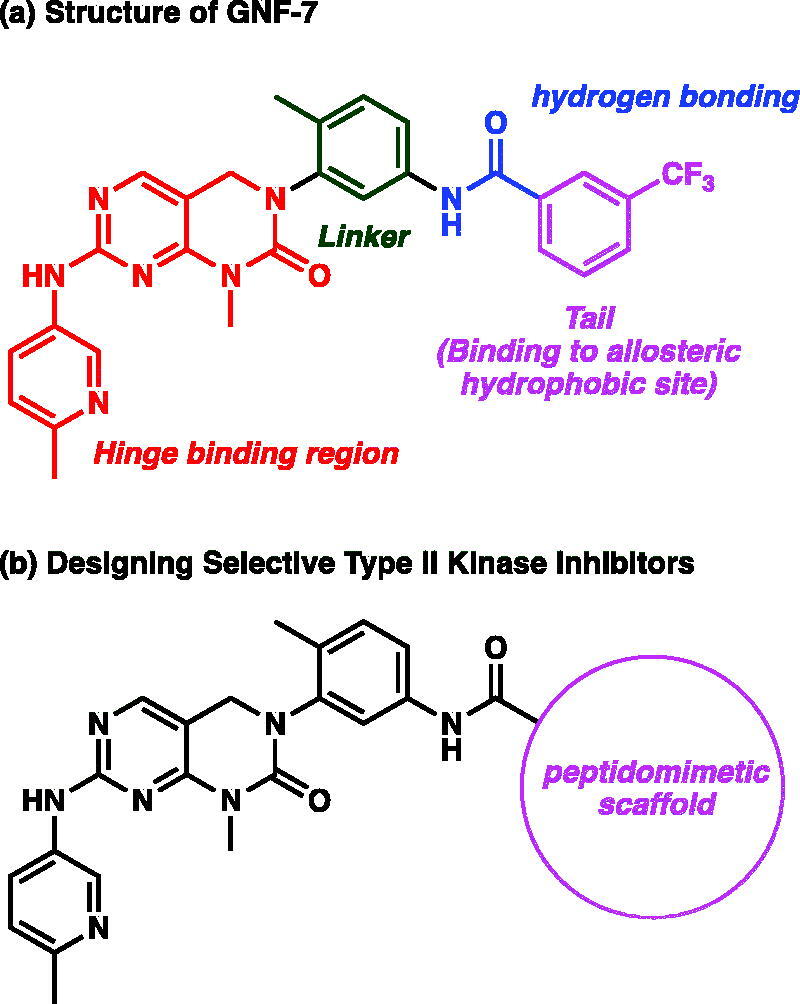
(a) Structure of GNF-7. (b) Designing selective type II kinase inhibitors with peptidomimetic scaffolds.

We noticed that a number of the less selective type II kinase inhibitors possess achiral and limited chemotype tails, which interact with allosteric site of the inactive conformation of kinases. We envisioned that interactions of flat and achiral tail fragments with 3-dimensional structural kinases would be highly limited and resulted in less selective profile^6^. Thus, we were curious whether type II kinase inhibitors containing chiral tails would be more selective among all of the kinases by affording various binding modes^7^^,^^8^. We were particularly interested in peptidomimetic structure as tail scaffolds. To our best knowledge, development of selective type II kinase inhibitors that consists of peptidomimetic tails is unprecedented. To investigate how peptidomimetic tail structure affects kinase selectivities, we chose the core structure of GNF-7 (Figure 1)^9^. GNF-7 has been discovered as a type II T315I Bcr-Abl kinase inhibitor and possesses remarkable potencies against many kinases with highly low kinase selectivity. Among the peptidomimetic structure, we were attracted to synthetically easily accessible solution phase turn mimetic libraries^10^.

## 2. Results and discussion

### 2.1. Chemistry

**Scheme 1.**
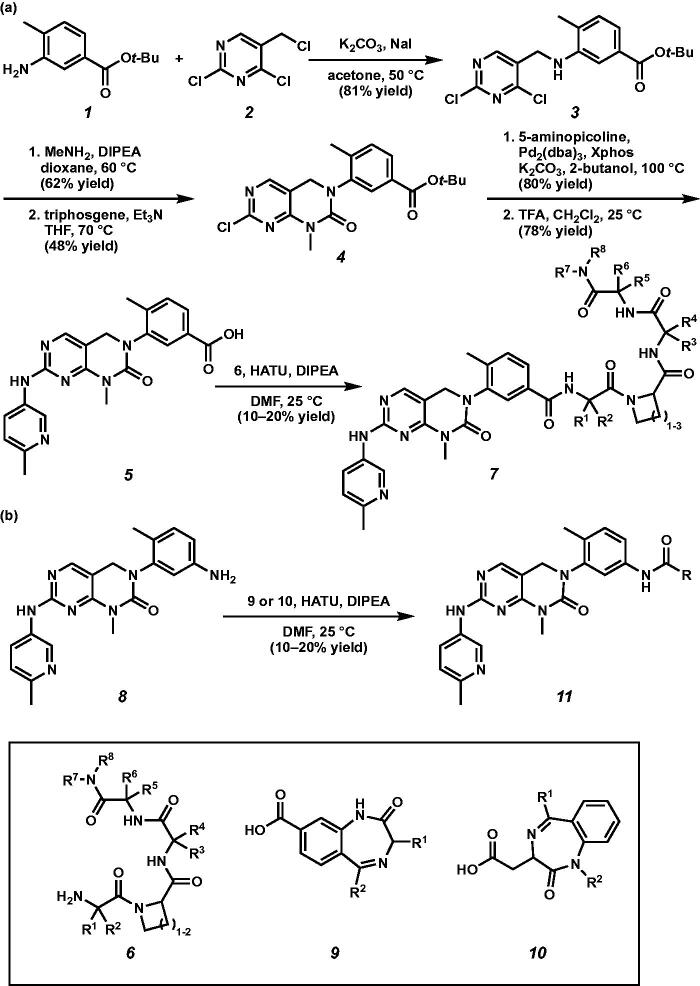
(a) Synthesis of kinase inhibitors containing turn mimetic amide scaffolds. (b) Synthesis of kinase inhibitors possessing benzodiazepines.

We initially prepared kinase inhibitors, containing small molecular β-turn mimetic scaffolds developed by the Miller group (Scheme 1(a))^10b,f^. Alkylation of chloromethyl pyrimidine **2** with aniline **1** provided *tert*-butyl ester **3** under basic conditions in 81% yield. Nucleophilic aromatic substitution of 2,4-dichloropyrimidine **3** with methylamine, followed by cyclic urea formation using triphosgene afforded urea **4**. Buchwald coupling of chloropyrimidine **4** with 5-aminopicoline and subsequent removal of *tert*-butyl group under acidic conditions smoothly generated acid **5**. Amide coupling of acid **5** with various β-turn mimetic scaffolds **6** produced amides **7** in 10–20% yields. We also prepared kinase inhibitors containing benzodiazepines as turn mimetic scaffolds (Scheme 1(b)). Amide coupling of benzodiazepines **9** and **10**^10d,e^ with aniline **8**^9a,c^ afforded amides **11** in 10–20% yields.

### 2.2. *In vitro* kinase inhibitory activities

Kinase-inhibitory activities of both **7** and **11** against four selected kinases were assessed by *in vitro* kinase assay (Table 1). To our delight, both **7a** and **11b** showed high degree of selectivities among selected kinases even between structurally similar Lck and c-Src (over 10-fold selectivities). Replacement of L-Pro with D-Pro led to lower selectivities between Lck and c-Src (**7a** and **7b**). This is not surprising since stereochemical alteration of Pro at *i* + 1 might potentially change 3-dimensional conformation of the structure^10b^. The substituents at N-1, C-3, and C-5 positions on the 1,4-benzodiazepin-2-one ring significantly affected selectivities. In all cases, the activities on Lck kinases were found to be superior to those on other kinases. Gratifyingly, **11b** possessed over 10-fold selectivity for Lck over c-Src. The *iso*-butyl group at C-3 position in the 1,4-benzodiazepin-2-one surpassed the methyl group in respect of the selectivity (**11b** and **11d**). Also, the 2-butyl group at the C-3 position causes almost no selectivity (**11e**).

**Table 1.** *In vitro* potency profiling on selected kinases.

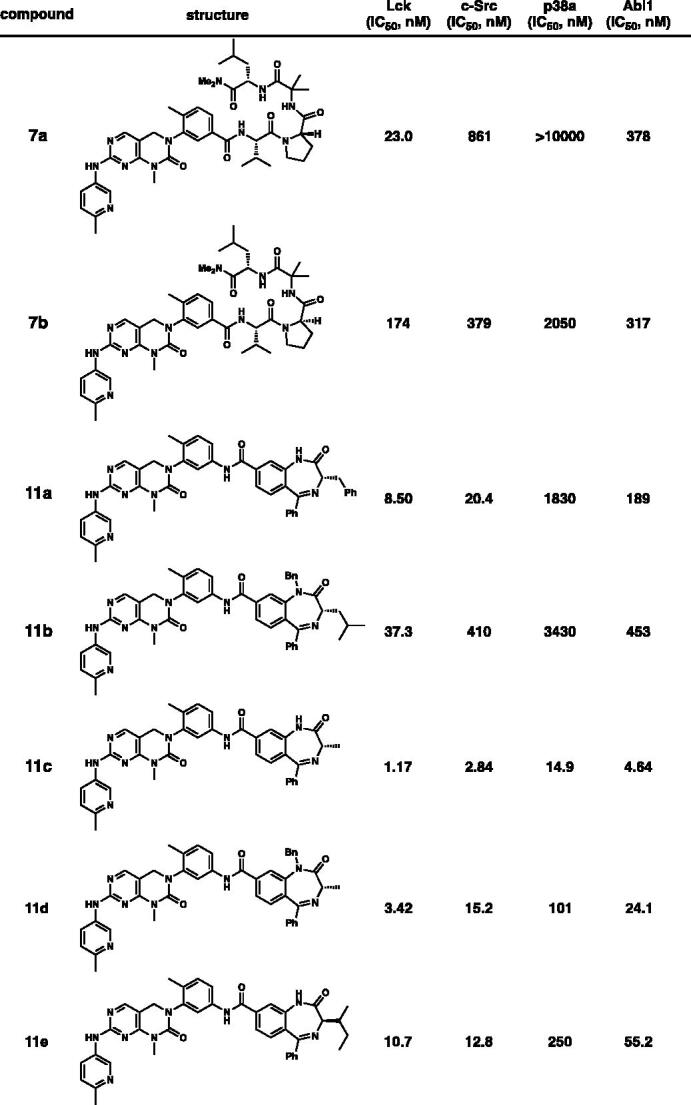

With the exciting initial data in hand, selectivities between Lck and c-Src were investigated on derivatives **7** containing the β-turn mimetic scaffolds (Table 2). Kinase-inhibitory activities of derivatives **7** against Lck and c-Src were assessed by *in vitro* kinase assay. Interestingly, **7c** possessing alternative stereochemistry compared to **7a** showed significantly (>10-fold) diminished selectivity. Changing *i* + 2 functional groups to cyclopropane (**7d** and **7e**), cyclobutane (**7f**), and even glycine (**7g** and **7h**) exhibited high selectivities. However, we observed diminished selectivity with benzyl substitution at *i* + 2 position (**7i**). High selectivity was kept with piperidine scaffold at *i* + 1 (**7j**). In contrast to the pyrrolidine and the piperidine groups, addition of the azetidine at *i* + 1 (**7k**) resulted in over 10-fold lower selectivity. The selectivities were not decreased by replacement of *iso*-butyl with valine at *i* + 3 (**7l**, **7m**, **7n**, and **7o**). Substituents at *i* significantly affected selectivities (**7p**, **7q**, **7r**, **7s**, and **7t**). High selectivities were observed with *iso*-butyl and cyclohexyl substitution at *i* (over 10-fold selectivities, **7q** and **7s**). Compared to **7a**, The selectivities of **7v** and **7w** on Lck and c-Src were highly diminished^10f^.

**Table 2.** *In vitro* potency profiling on Lck and c-Src.

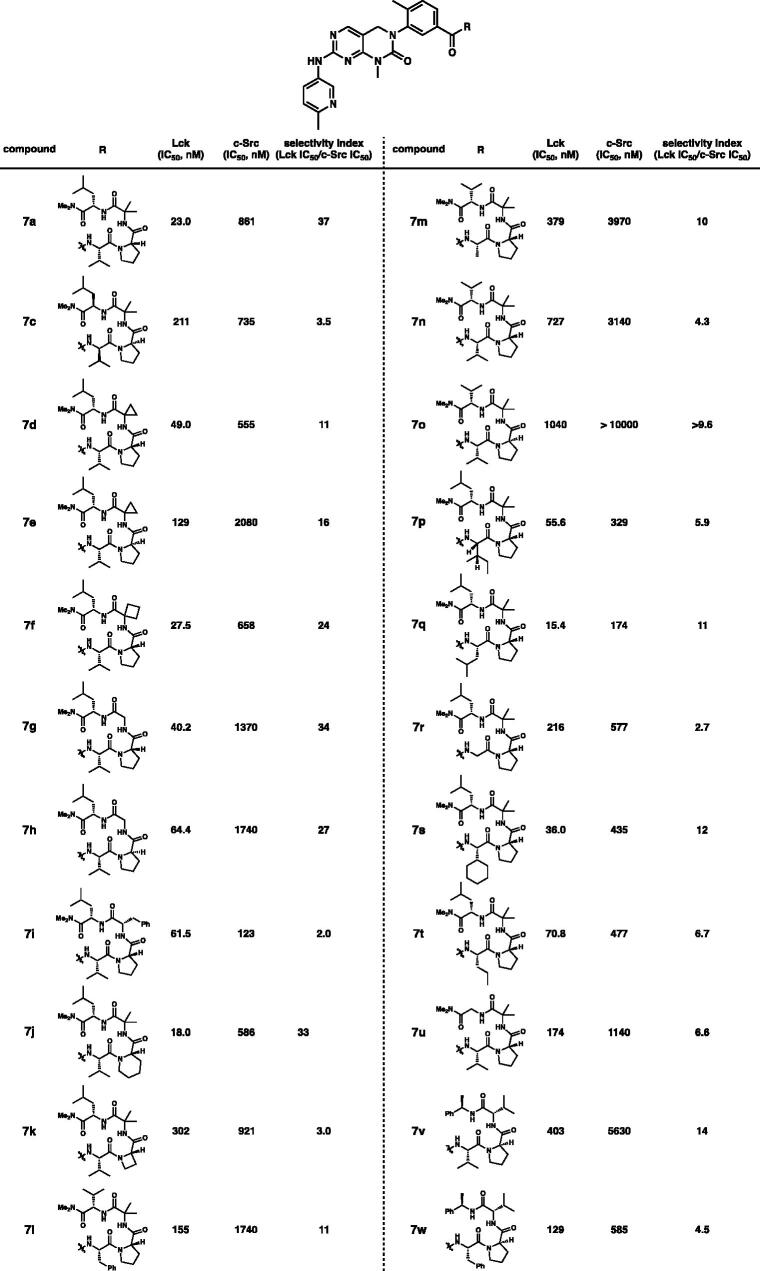

Additionally, we investigated the selectivities of derivatives containing benzodiazepine scaffolds on Lck and c-Src kinases (Table 3). Kinase-inhibitory activities of derivatives **11** against Lck and c-Src were assessed by *in vitro* kinase assay. The selectivities of **11f** was similar to those of its enantiomer **11a** and *p*-fluorobenzyl **11g**. It is noteworthy that the benzyl group at N-1 position of the 1,4-benzodiazepin-2-one moiety (**11b)** was superior to the methyl, allyl, *iso*-butyl, methoxyethyl, and *N*-benzylacetamide groups at the corresponding position in terms of selectivities (**11h**, **11i**, **11j**, **11n**, and **11o**). The methyl group in **11k** and **11l** at C-5 position is slightly more favourable than the phenyl group (**11a** and **11f**) at the corresponding position as regards the selectivity. We observed that introduction of γ-turn mimicry^10e^ at the tail position resulted in almost no selectivity for Lck over c-Src (**11p** and **11q**).

**Table 3.** *In vitro* potency profiling on Lck and c-Src.

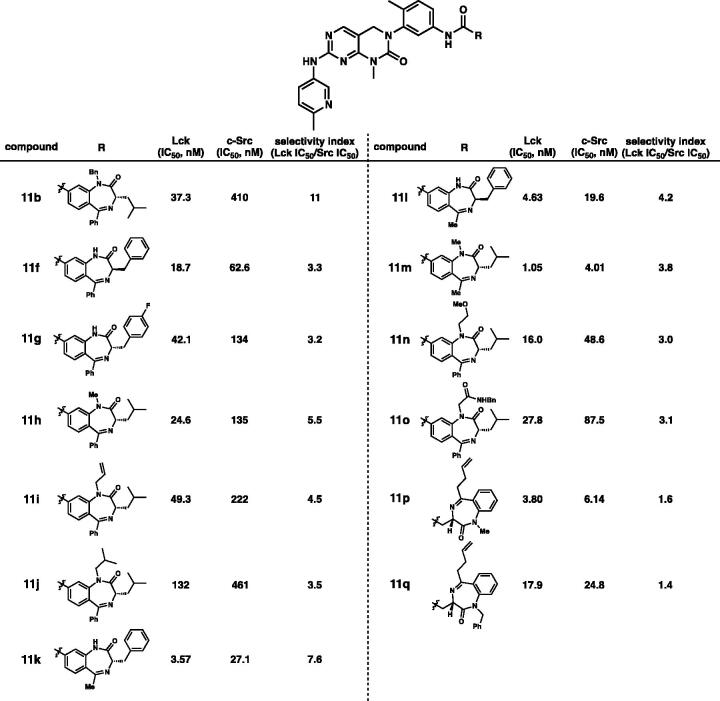

We explored *in vitro* potencies of **7a** and **11b** at both 14 µΜ (Km) and 1 mM ATP concentrations to investigate whether these inhibitors are ATP competitive inhibitors (Table 4). We observed that the IC_50_ values were increased dramatically at 1 mM ATP concentration compared to those at Km ATP concentrations. Thus, we concluded that both **7a** and **11b** are ATP competitive kinase inhibitors.

**Table 4.** Biochemical IC_50_ values of **7a** and **11b** on Lck at Km and 1 mM ATP concentration.

Kinase	ATP conc.	IC_50_ (nM)		
		7a	11b	Staurosporine (ref)
Lck	14 *µ*M	190	510	1.32
Lck	1 mM	21000	>100000	15.6

### 2.3. Kinome-wide selectivities of 7a described on a kinome phylogenetic tree

**Figure 2.**
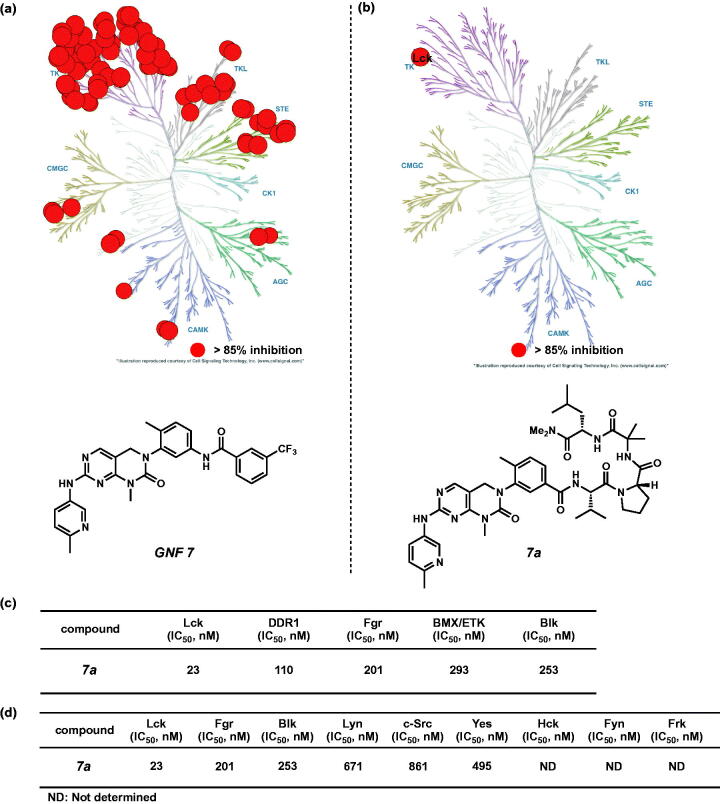
This illustration was reproduced courtesy of Cell Signalling Technology, Inc. (www.cellsignal.com). (a) Kinome phylogenetic tree description of the GNF 7 selectivity profile. (b) Kinome-wide selectivities of **7a** described on a kinome phylogenetic tree. (c) *In vitro* IC_50_ values of **7a** against kinases, which were inhibited greater than 70%. (d) *In vitro* IC_50_ values of **7a** against Src family kinases.

We were pleased to confirm that **7a** possesses higher selectivity for Lck over other 373 kinases at 1 µM concentration compared to GNF 7 (Figure 2, Supplementary Table S1–S4). We also obtained IC_50_ values of **7a** against five kinases (Lck, DDR1, Fgr, Bmx, and Blk), which were inhibited greater than 70% in the kinome-wide profiling analysis (Figure 2c, Supplementary Table S1). As shown in Figure 2(c), **7a** has an IC_50_ value of 23 nM against Lck and possesses 5 to 13-fold selectivity over these four kinases. Furthermore, **7a** showed more than 10-fold selectivities over other structurally similar Src family kinases (Figure 2d).

### 2.4. Docking into Lck binding site

**Figure 3.**
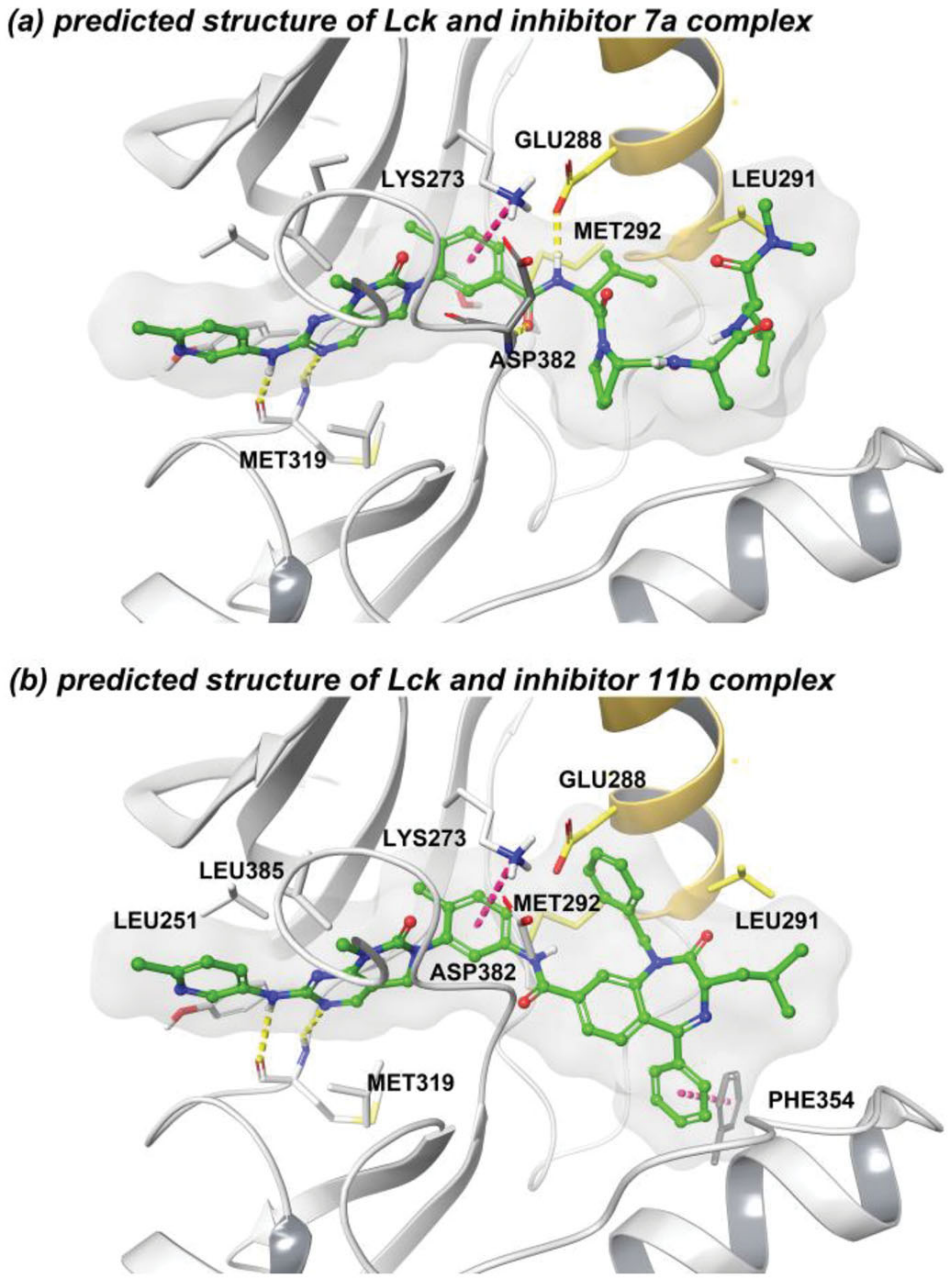
(a) Predicted structure of Lck and **7a** complex. (b) Predicted structure of Lck and **11b** complex. (red dashed line: π–π interaction/cation-π interaction, yellow dashed line: hydrogen-bonding).

Docking of **7a** and **11b** with Lck kinase by long time (3 μs) molecular dynamics (MD) simulation revealed that turn peptidomimetic scaffolds were located at an allosteric binding site. Also, hydrophobic groups of the peptidomimetic scaffolds interacted with the allosteric helix of the binding site (C-helix in Figure 3). The results reveal that the amide moiety of **7a** participates in hydrogen-bonding networks with Glu288 in Lck. Additionally, the phenyl group of **11b** interacts with Phe354 by π–π stacking interaction. The core scaffolds of **7a** and **11b** were interacted with kinases hinge region through similar binding poses of the crystal structure of imatinib with kinases (Supplementary Figure S1). The Asp from the “DFG-motif” interacts with the amide functional group of **7a** and **11b**. Moreover, phenyl moieties on the linker region of **7a** and **11b** interacts with Lys273 by cation-π interaction.

### 2.5. Watermap application

**Figure 4.**
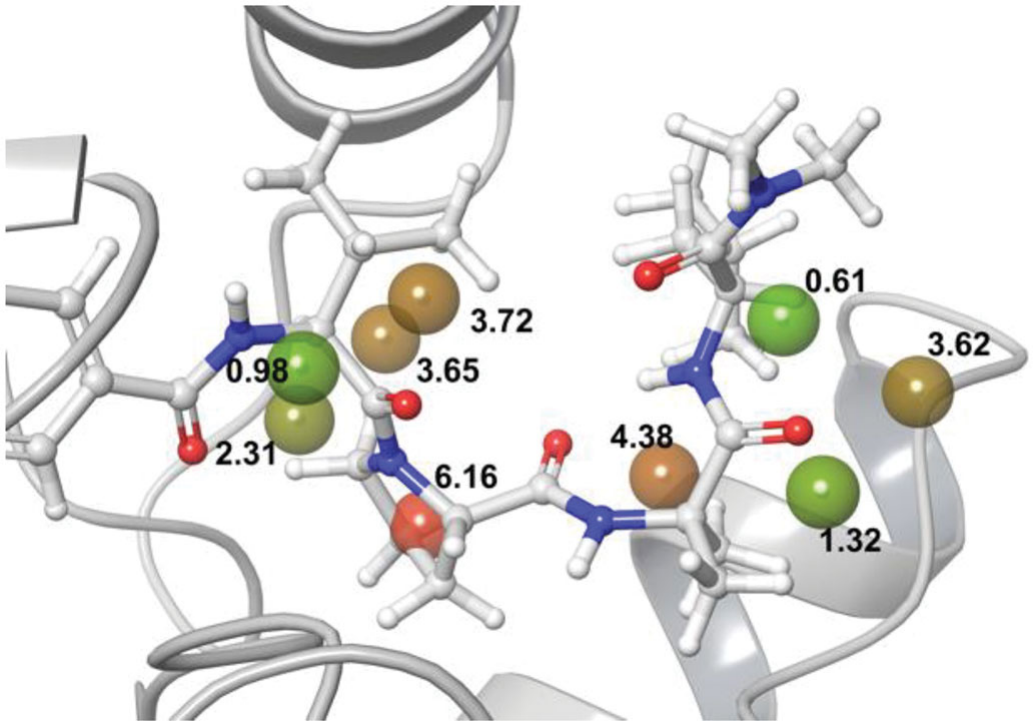
Analysis results of Lck and superimpose of **7a** at the allosteric site by using WaterMap application. Coloured sphere: hydration sites occupied by the inhibitor. The number on the sphere: ΔG energy (kcal/mol). Green sphere: low energy. Red sphere: high energy over 5 kcal/mol.

Next, we further analysed binding sites using WaterMap application^11^^,^^12^. With WaterMap results, many hydration sites with unstable energy (0 > kcal/mol) were found in the allosteric binding site (Supplementary Figure S2). Therefore, when **7a** was located at the unstable hydration sites, high binding free energy compensation could be obtained. Five hydration sites with a high energy of over 3.00 kcal/mol were found in Lck, and the peptidomimetic scaffold of **7a** occupied these sites (Figure 4). We envisioned that the energetically unstable regions at the allosteric site of Lck could be compensated with interaction of terminal turn peptidomimetic tail of **7a**, which would contribute to high selectivity of **7a** for Lck kinase.

**Figure 5.**
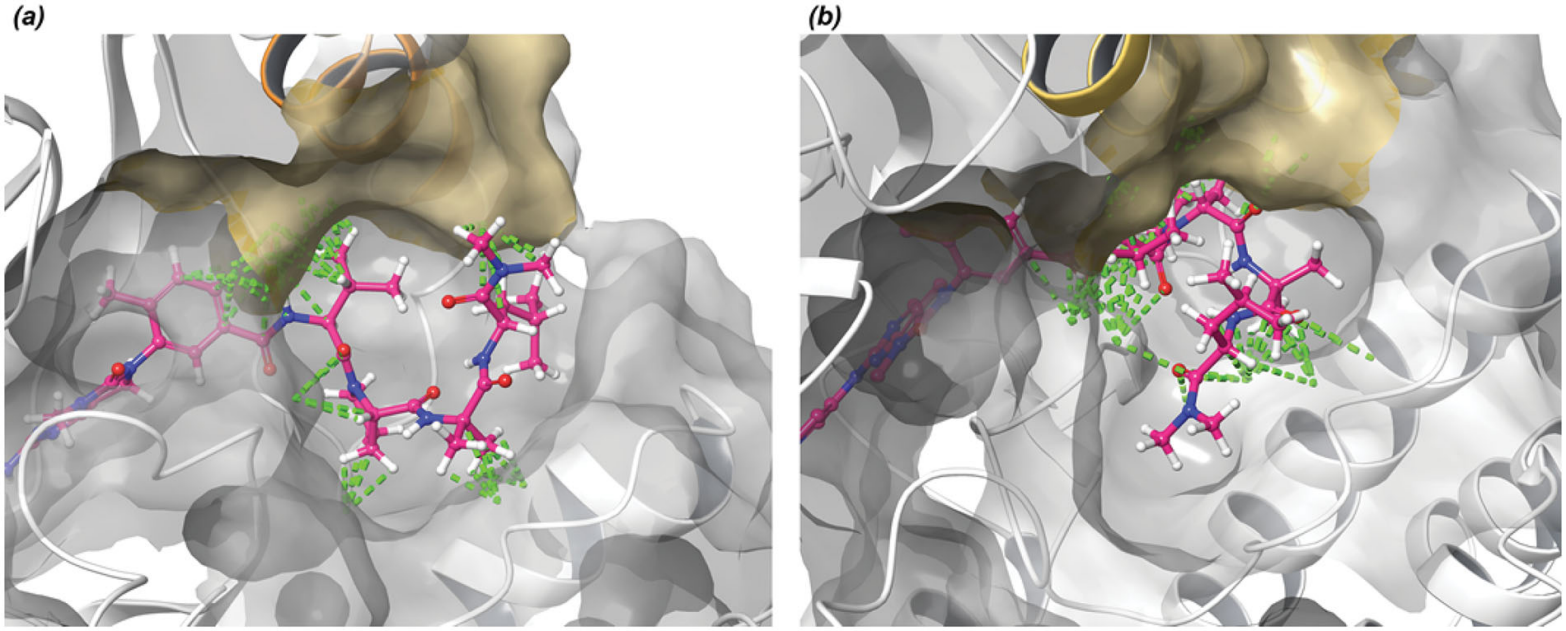
(a) Analysis results of Lck and superimpose of turn scaffold of **7a** at the allosteric site by using WaterMap application. (b) Analysis results of Lck and superimpose of turn scaffold of **7c**, which is enantiomer of **7a** at the allosteric site by using WaterMap application. (green dashed line: van der Waals interaction).

With the interesting WaterMap calculation result, we attempted to explain the IC_50_ difference of **7a** and its enantiomer **7c** on Lck by the WaterMap application method (Figure 5). The U-shaped tail of **7a** is located in the allosteric binding site on Lck. Also, amino acids at *i* and *i* + 3 of **7a** tail interact with allosteric helix. However, turn structure of **7c** is not fully located at the allosteric binding site of Lck, which is expected to be highly important for kinase selectivities. Although the amino acid at *i* of **7c** tail interacts with allosteric helix, the amino acid at *i* + 3 is extruded outside and exposed to solvent. Thus, the tail structure of **7c** could have high fluctuation, which results in lower selectivities (Figure 5).

### 2.6. Inhibitory effect of 7a on Lck activation

**Figure 6.**
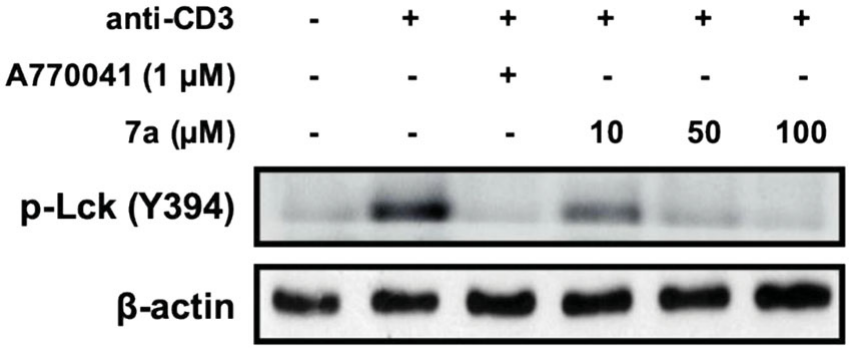
Effect of **7a** on the Lck (Y394) activation in anti-CD3-treated Jurkat cells. After treatment with CD3 antibody for coating in plates, cells were seeded and then treated with various concentrations of **7a** (10, 50, or 100 μM) or A770041 (1 μM) used as a positive control. The phosphorylation of Lck tyrosine in Jurkat cells was activated by anti-CD3 mAb. Total cellular protein was resolved by SDS-PAGE, transferred to PVDF membranes, and detected with specific p-Lck (Y394) antibody. β-Actin was used as an internal control.

We investigated whether Lck activation was affected by **7a** in Jurkat cell line (Figure 6). Western blot analysis revealed that **7a** markedly reduced the phosphorylated Lck levels at tyrosine 394 residue in a concentration-dependent manner in anti-CD3-treated Jurkat T cells. These results suggested that **7a** inhibited the anti-CD3-activated Lck, similar to positive control A770041.

### 2.7. *In vivo* experiment with dextran sulphate sodium (DSS)-induced colitis model

**Figure 7.**
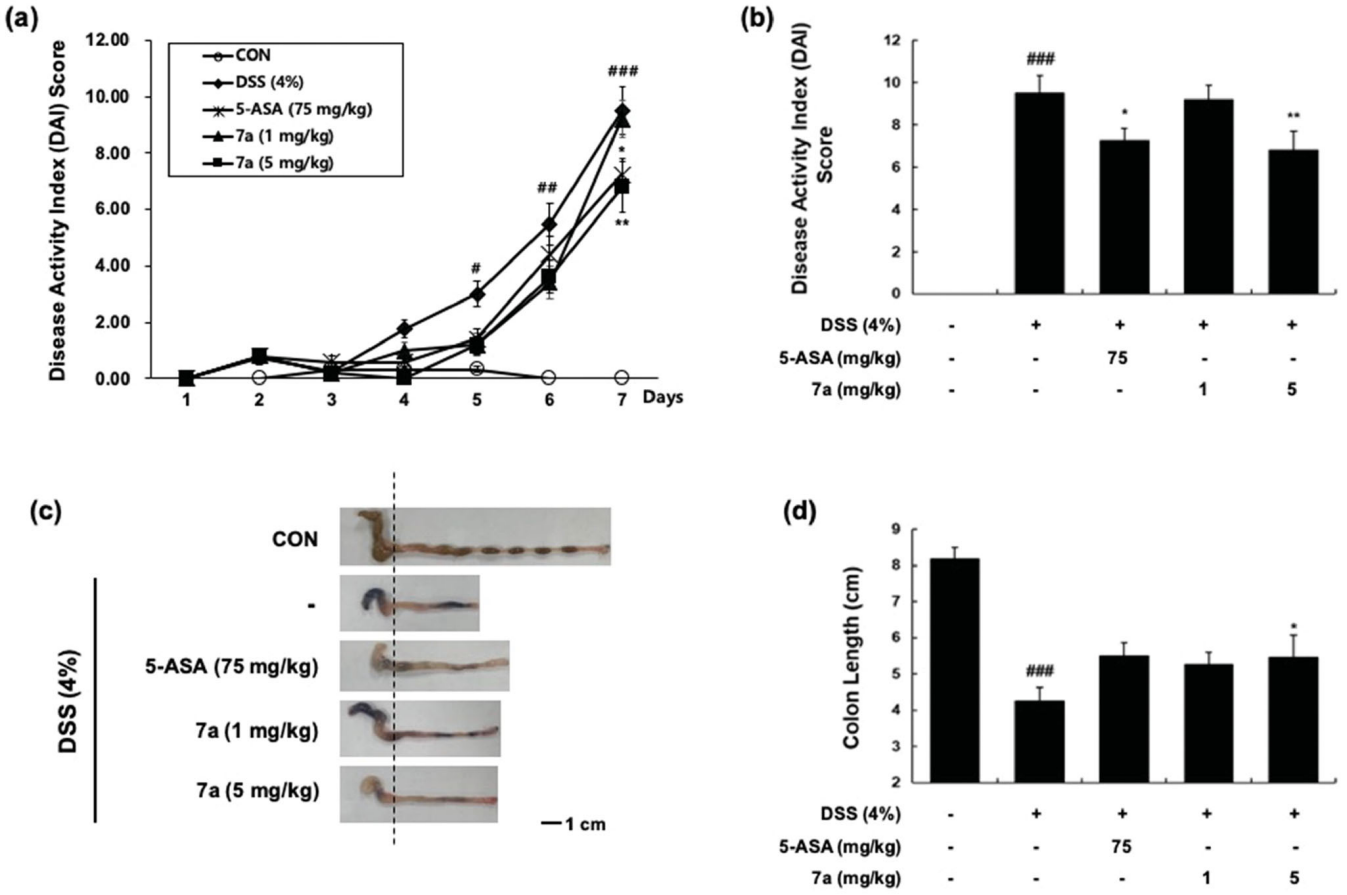
Effect of **7a** on the progression of DSS-induced colitis. (a) Disease activity index (DAI) levels during total experiments periods and (b) at the end of the experiment (day 7). (c, d) Colon length was measured at the end of the experiment (day 7). Data are presented as mean ± SE (*n* = 6). ^#^*p* < 0.05, ^##^*p* < 0.01, ^###^*p* < 0.001 vs. the vehicle-treated control group; *p < 0.05, **p < 0.01 vs. the DSS-treated group. The significance between groups was determined by ANOVA and Dunnett's post-hoc test.

Inflammatory bowel disease (IBD), which is a chronic and immune-mediated disorder of the gastrointestinal tract encompasses Crohn’s disease (CD) and ulcerative colitis (UC)^13^. Although the exact cause of IBD is unclear, it is widely accepted that an excessive immune response against normal components of microflora results in IBD. Especially, excessive T cell activation plays a pivotal role in mucosal damage in both CD and UC^14^. Lck plays a crucial role in activation of TCR-linked signal transduction pathways, leading to T cell activation and proliferation^15^. Additionally, it is reported that overexpression of Lck leads to IBD^15^. Hence, we evaluated the potential of our selective Lck kinase inhibitor **7a** for IBD treatment with dextran sulphate sodium (DSS)-induced colitis model. DSS administration induces acute colonic damage, and changes in clinical parameters can be monitored^16^. To determine the recovery effect of **7a** in DSS-induced colitis, we assessed the clinical symptom including disease activity index (DAI) and colon length. The DAI scores were evaluated by body weight loss, stool consistency, and occult/gross bleeding (Table 5). During the administration of DSS (4%) for 7 days, DAI values were significantly increased (Figure 7a). We discovered that **7a** treatment (5 mg/kg, *i.p.*) improved the symptom changes at the end of experiments (Figure 7b). In addition, the colon length of DSS-treated group was significantly shorter than that of the vehicle-administered control group (8.17 ± 0.32 cm vs. 4.25 ± 0.38 cm, *p < 0.001*), while **7a** treatment (5 mg/kg, *i.p.*) recovered the DSS-induced colon shortening (4.25 ± 0.38 cm vs. 5.47 ± 0.60 cm, *p < 0.05,* Figure 7(C), D).

**Table 5.** Assessment of the disease activity index (DAI)

DAI score	Bodyweight loss (%)	Stool consistency	Occult/gross bleeding
0	None	Normal	Negative
1	1–5		
2	5–10	Loose stools	Hemoccult positive
3	10–20		
4	>20	Diarrhea	Gross bleeding

## 3. Conclusion

In conclusion, we discovered highly selective type II kinase inhibitors by introducing chiral turn peptidomimetic moieties on the tail region for the first time. It turned out that **7a**, a novel type II kinase inhibitor, is a potent and exceptionally selective Lck inhibitor. Based on kinome-wide selectivity profiling data, it was confirmed that **7a** possesses high selectivity. Kinases selectivities were highly affected by subtle changes of the substituents of peptidomimetic scaffolds. To the best of our knowledge, it has never been reported that low selectivity of a type II kinase inhibitor is dramatically enhanced by adopting chiral peptidomimetic tail. The western blot analysis revealed that **7a** is capable of inhibiting Lck activation in anti-CD3-treated Jurkat T cells. Finally, we discovered that **7a** could alleviate of clinical symptoms in DSS-induced colitis mice. This study may shed a bright light on the design of selective type II kinase inhibitors by adopting chiral peptidomimetic tails.

## 4. Experimental

### 4.1. Chemistry

#### 4.1.1. General procedures

Unless otherwise stated, reactions were performed in flame-dried glassware under a nitrogen atmosphere using dry solvents. Reaction progress was monitored by thin-layer chromatography (TLC). Purified water was obtained using a Barnstead NANOpure Infinity UV/UF system. Brine solutions are saturated aqueous solutions of sodium chloride. Commercially available reagents were purchased from Sigma-Aldrich, Acros Organics, Combi-Blocks, TCI or Alfa Aesar and used as received unless otherwise stated. Reaction temperatures were controlled by an IKAmag temperature modulator unless otherwise indicated. TLC was performed using E. Merck silica gel 60 F254 precoated glass plates (0.25 mm) and visualised by UV fluorescence quenching, KMnO_4_, or Ninhydrin staining. Silicycle Silia*Flash* P60 Academic Silica gel (particle size 0.040–0.064 mm) was used for flash column chromatography. 1H NMR spectra were recorded on Bruker 400 MHz and 600 MHz spectrometer and are reported relative to residual CDCl_3_ (δ 7.26 ppm), CD_3_OD (δ 3.31 ppm) or (CD_3_)_2_SO (δ 2.50 ppm). 13C NMR spectra are recorded on Bruker 400 MHz and 600 MHz spectrometer (101 MHz & 151 MHz) and are reported relative to CDCl_3_ (δ 7.26 ppm), CD_3_OD (δ 3.31 ppm) or (CD_3_)_2_SO (δ 2.50 ppm). 19F NMR spectrum is recorded on Bruker 600 MHz spectrometer (377 MHz) and is reported relative to (CD_3_)_2_SO (δ 2.50 ppm). Data for 1H NMR are reported as follows: s = singlet, d = doublet, t = triplet, q = quartette, p = pentet, sept = septuplet, m = multiplet, br s = broad singlet, br d = broad doublet, app = apparent. Data for 13C are reported in terms of chemical shifts (δ ppm). Data for 19F is reported in terms of chemical shifts (δ ppm). The purity of final compounds was determined to be ≥95% using HPLC analyses performed on an Agilent 1100 series with a Poroshell C18 column (pore size: 120 Å; particle size: 4 µm, dimensions: 4.6 × 150 mm). Some final compounds are purified using PREP HPLC performed on an Agilent 1260 Infinity II with Agilent Prep-C18 column (particle size: 10 µm, dimensions: 250 × 21.2 mm). IR spectra were obtained using a Nicolet Avatar 330 FT-IR spectrometer and Bruker Alpha Platinum-ATR using thin films deposited on NaCl plates and reported in frequency of absorption (cm^−1^). Optical rotations were measured with a Rudolph AUTOPOL I automatic polarimeter operating on the sodium D-line (589 nm), using a 100 nm path-length cell and are reported as: [α]_D_^T^ (concentration in g/100 ml, solvent). High resolution mass spectra (HRMS) were obtained from Waters SYNAPT G2 TOF with a Waters Multimode source in electrospray ionisation (ESI+), atmospheric pressure chemical ionisation (APCI+), or mixed ionisation mode (MM: ESI-APCI+).

#### 4.1.2. Tert-butyl 3-amino-4-methylbenzoate (1)

To a solution of *tert*-butyl 4-methyl-3-nitrobenzoate (113 mg, 0.475 mmol, 1.00 equiv) in EtOH (1.18 ml) was added Pd/C (24.0 mg, 0.574 mmol), cyclohexene (1.18 ml). The reaction mixture was stirred for 16 h at 80 °C. Solids were removed via a filtration through a celite plug and the resulting solution was concentrated under reduced pressure. The filtrate was purified by flash column chromatography (4:1 hexanes:EtOAc) on silica gel to give aminobenzoate **1** (91.0 mg, 92% yield) as a yellow liquid. *R_f_:* 0.55 (4:1 hexanes:EtOAc); ^1^H NMR (400 MHz, DMSO) δ 7.17 (d, *J* = 1.4 Hz, 1H), 7.00 (t, *J* = 1.2 Hz, 2H), 5.07 (s, 2H), 2.08 (s, 3H), 1.51 (s, 9H); ^13 ^C NMR (101 MHz, DMSO) δ 165.60, 146.64, 129.79, 129.70, 126.17, 116.80, 114.09, 79.75, 30.69, 27.87, 17.57; IR (Neat) 3470, 3377, 2975, 2927, 2857, 1697, 1625, 1577, 1508, 1477, 1456, 1423, 1392, 1367, 1300, 1247, 1164, 1144, 1108, 1071, 1032, 997, 948, 886, 851, 825, 760, 642, 562, 527, 440 cm^−1^; HRMS (MM: ESI-APCI+) m/z calc’d for C_12_H_18_NO_2_ [M + H]^+^: 208.1338; found: 208.1334.

#### 4.1.3. Tert-butyl 3-(((2,4-dichloropyrimidin-5-yl)methyl)amino)-4-methylbenzoate (3)

To a solution of aminobenzoate **1** (1.20 g, 5.97 mmol, 1.00 equiv), 2,4-dichloro-5-(chloromethyl)pyrimidine **2** (1.40 g, 7.16 mmol, 1.20 equiv) in acetone (7.46 ml) was added NaI (1.34 g, 8.95 mmol, 1.50 equiv), K_2_CO_3_ (1.34 g, 11.3 mmol, 1.90 equiv). The reaction mixture was stirred for 10 h at 50 °C. The resulting suspension was filtered and washed with CH_2_Cl_2_. The filtrate was concentrated *in vacuo* and purified by flash column chromatography (4:1 hexanes:EtOAc) on silica gel to give dichloropyrimidine **3** (2.10 g, 81% yield) as an orange liquid. *R_f_:* 0.5 (4:1 hexanes:EtOAc); ^1^H NMR (400 MHz, CDCl_3_) δ 8.51 (s, 1H), 7.36 (dd, *J* = 7.7, 1.6 Hz, 1H), 7.13 (dd, *J* = 7.7, 0.8 Hz, 1H), 7.07 (d, *J* = 1.6 Hz, 1H), 4.55 (s, 2H), 2.25 (s, 3H), 1.55 (s, 9H); ^13 ^C NMR (101 MHz, CDCl_3_) δ 165.97, 161.40, 159.49, 159.38, 144.22, 131.17, 130.41, 129.61, 127.51, 119.87, 110.29, 80.90, 42.65, 28.23, 17.72; IR (Neat) 3411, 2977, 2931, 1699, 1610, 1580, 1560, 1519, 1474, 1450, 1422, 1384, 1367, 1349, 1299, 1247, 1163, 1115, 1093, 1065, 1032, 993, 951, 916, 856, 819, 798, 760, 732, 703, 687, 648, 468, 439, 419 cm^−1^; HRMS (MM: ESI-APCI+) m/z calc’d for C_17_H_19_Cl_2_N_3_O_2_Na [M + Na]^+^: 390.0754; found: 390.0745.

#### 4.1.4. Tert-butyl 3-(7-chloro-1-methyl-2-oxo-1,4-dihydropyrimido[4,5-d]pyrimidin-3(2H)-yl)-4-methylbenzoate (4)

To a solution of dichloropyrimidine **3** (1.50 g, 4.00 mmol, 1.00 equiv) in 1,4-dioxane (13.6 ml) was added MeNH_2_ (8.14 ml, 8.14 mmol, 1.50 equiv), *i*-Pr_2_NEt (2.12 ml, 12.2 mmol, 3.00 equiv). The reaction mixture was stirred for 1.5 h at 60 °C and then diluted with CH_2_Cl_2_ and water. The aqueous phase was extracted with CH_2_Cl_2_. The combined organic phases were washed with brine, dried over MgSO_4_ and concentrated *in vacuo*. The residue was purified by flash column chromatography (1:1 hexane:EtOAc) on silica gel to give *tert*-butyl 3-(((2-chloro-4-(methylamino)pyrimidin-5-yl)methyl)amino)-4-methylbenzoate (0.920 g, 62% yield) as a pale-yellow liquid. *R_f_*: 0.18 (4:1 hexanes:EtOAc); ^1^H NMR (400 MHz, CDCl_3_) δ 7.94 (s, 1H), 7.45 (dd, *J* = 7.7, 1.6 Hz, 1H), 7.39 (d, *J* = 1.6 Hz, 1H), 7.15 (d, *J* = 7.8 Hz, 1H), 4.20 (s, 2H), 3.04 (d, *J* = 4.9 Hz, 3H), 2.19 (s, 3H), 1.59 (s, 9H); ^13^C NMR (101 MHz, CDCl_3_) δ 166.08, 163.32, 160.61, 154.74, 145.11, 131.32, 130.36, 128.86, 120.96, 112.60, 112.05, 81.11, 43.76, 28.36, 28.11, 17.92; IR (Neat) 3361, 2955, 2923, 2853, 1705, 1597, 1580, 1514, 1455, 1423, 1397, 1367, 1342, 1300, 1270, 1247, 1166, 1115, 1069, 1031, 992, 934, 873, 851, 779, 761, 737, 700, 517, 463 cm^−1^; HRMS (MM: ESI-APCI+) m/z calc’d for C_18_H_24_ClN_4_O_2_ [M + H]^+^: 363.1588; found: 363.1585.

To a solution of *tert*-butyl 3-(((2-chloro-4-(methylamino)pyrimidin-5-yl)methyl)amino)-4-methylbenzoate (0.500 g, 1.40 mmol, 1.00 equiv) in THF (4.60 ml) was added triphosgene (0.200 g, 0.700 mmol, 0.500 equiv), Et_3_N (0.950 ml, 7.00 mmol, 5.00 equiv) at 0 °C under N_2_ atmosphere. The reaction mixture was stirred for 1 h at 70 °C and then diluted with CH_2_Cl_2_ and water. The aqueous phase was extracted with CH_2_Cl_2_. The combined organic phases were washed with brine, dried over MgSO_4_ and concentrated *in vacuo* and washed with isopropyl ether to give methyl urea **4** (0.920 g, 62% yield) as a pale-yellow solid. *R_f_*: 0.66 (1:1 hexane:EtOAc); ^1^H NMR (400 MHz, CDCl_3_) δ 8.12 (s, 1H), 7.90 (dd, *J* = 7.9, 1.7 Hz, 1H), 7.84 (d, *J* = 1.7 Hz, 1H), 7.36 (d, *J* = 8.0 Hz, 1H), 4.83 (d, *J* = 14.8 Hz, 1H), 4.55 (d, *J* = 14.8 Hz, 1H), 3.47 (s, 3H), 2.28 (s, 3H), 1.58 (s, 9H); ^13 ^C NMR (101 MHz, CDCl_3_) δ 164.89, 160.45, 158.98, 153.47, 151.88, 140.88, 140.32, 131.86, 131.48, 129.55, 128.06, 110.34, 81.54, 47.15, 29.05, 28.33, 18.01; IR (Neat) 2978, 1688, 1584, 1471, 1432, 1395, 1360, 1336, 1288, 1253, 1212, 1157, 1143, 1126, 1107, 1069, 1035, 976, 932, 874, 849, 825, 791, 764, 749, 734, 694, 637, 531, 475, 454, 418 cm^−1^; HRMS (MM: ESI-APCI+) m/z calc’d for C_19_H_22_ClN_4_O_3_ [M + H]^+^: 389.1380; found: 389.1378.

#### 4.1.5. 4-Methyl-3-(1-methyl-7-((6-methylpyridin-3-yl)amino)-2-oxo-1,4-dihydropyrimido[4,5-d]pyrimidin-3(2H)-yl)benzoic acid (5)

To a solution of methyl urea **4** (1.70 g, 4.50 mmol, 1.00 equiv) in butan-2-ol (22.5 ml) was added 5-aminopicoline (0.500 g, 4.54 mmol, 1.01 equiv), K_2_CO_3_ (3.10 g, 22.5 mmol, 5.00 equiv), Xphos (0.400 g, 0.800 mmol, 0.200 equiv), Pd_2_(dba)_3_ (0.800 g, 0.800 mmol, 0.200 equiv). The reaction mixture was stirred for 2 h at 100 °C. Solids were removed via a filtration through a celite plug and the resulting solution was concentrated under reduced pressure. The filtrate was purified by flash column chromatography (1:20 to 1:10 MeOH:CH_2_Cl_2_) on silica gel to give *tert*-butyl 4-methyl-3-(1-methyl-7-((6-methylpyridin-3-yl)amino)-2-oxo-1,4-dihydropyrimido[4,5-*d*]pyrimidin-3(2*H*)-yl)benzoate (1.65 g, 80% yield) as a white solid. *R_f_:* 0.53 (1:1 hexane:THF); ^1^H NMR (400 MHz, CDCl_3_) δ 8.74 (d, *J* = 2.6 Hz, 1H), 8.01 (dd, *J* = 8.4, 2.7 Hz, 1H), 7.99 (s, 1H), 7.88 (dd, *J* = 7.9, 1.8 Hz, 1H), 7.85 (d, *J* = 1.7 Hz, 1H), 7.34 (d, *J* = 7.9 Hz, 1H), 7.29 (s, 1H), 7.16 (d, *J* = 8.4 Hz, 1H), 4.75 (dd, *J* = 13.8, 1.1 Hz, 1H), 4.52 − 4.41 (m, 1H), 3.45 (s, 3H), 2.56 (s, 3H), 2.28 (s, 3H), 1.58 (s, 9H); ^13 ^C NMR (101 MHz, CDCl_3_) δ 164.89, 159.20, 157.76, 152.73, 152.61, 151.96, 140.87, 140.73, 140.03, 133.83, 131.51, 131.17, 129.10, 128.02, 127.45, 123.15, 103.08, 81.25, 47.38, 28.72, 28.19, 23.43, 17.90; IR (Neat) 3277, 2956, 2925, 2854, 1709, 1681, 1605, 1576, 1532, 1491, 1413, 1369, 1331, 1290, 1255, 1235, 1167, 1126, 1071, 1032, 951, 848, 786, 752, 682, 643, 520, 454 cm^−1^; HRMS (MM: ESI-APCI+) m/z calc’d for C_25_H_29_N_6_O_3_ [M + H]^+^: 461.2301; found: 461.2301.

To a solution of *tert*-butyl 4-methyl-3-(1-methyl-7-((6-methylpyridin-3-yl)amino)-2-oxo-1,4-dihydropyrimido[4,5-d]pyrimidin-3(2*H*)-yl)benzoate (0.460 g, 1.00 mmol, 1.00 equiv) in CH_2_Cl_2_ (10.0 ml) was added trifluoroacetic acid (1.50 ml, 20.0 mmol, 20.0 equiv). The reaction mixture was stirred for 12 h at 23 °C. The resulting solution was concentrated under reduced pressure and washed with ether to give benzoic acid **5** (0.400 g, 78% yield) as a white solid. *R_f_:* 0.13 (1:10 MeOH:CH_2_Cl_2_); ^1^H NMR (400 MHz, MeOD) δ 9.49 (d, *J* = 2.5 Hz, 1H), 8.47 (dd, *J* = 8.9, 2.6 Hz, 1H), 8.22 (d, *J* = 1.1 Hz, 1H), 7.98 (d, *J* = 1.8 Hz, 1H), 7.94 (dd, *J* = 7.9, 1.8 Hz, 1H), 7.80 (d, *J* = 8.9 Hz, 1H), 7.45 (d, *J* = 8.0 Hz, 1H), 4.83 (d, *J* = 1.1 Hz, 1H), 4.61 (dd, *J* = 14.2, 0.9 Hz, 1H), 3.47 (s, 3H), 2.72 (s, 3H), 2.30 (s, 3H); ^13^C NMR (101 MHz, MeOD) δ 167.51, 158.20, 157.69, 153.21, 152.77, 145.38, 141.51, 140.91, 138.76, 134.47, 131.06, 130.09, 129.12, 129.05, 128.40, 127.49, 105.25, 46.79, 27.77, 17.36, 16.47.IR (Neat) 3041, 2923, 1674, 1602, 1566, 1503, 1467, 1421, 1340, 1286, 1265, 1234, 1182, 1128, 1107, 1068, 1024, 872, 840, 795, 767, 745, 720, 704, 683, 640, 618, 564, 517, 447 cm^−1^; HRMS (MM: ESI-APCI+) m/z calc’d for C_21_H_21_N_6_O_3_ [M + H]^+^: 405.1675; found: 405.1674.

#### 4.1.6. General Procedure a for synthesis of amides 7

To a solution of benzoic acid **5** (1.20 equiv) in DMF (0.100 M) was added amine **6** (1.00 equiv), *i*-Pr_2_NEt (5.00 equiv), HATU (2.00 equiv). The reaction mixture was stirred for 12 h at 23 °C and then diluted with CH_2_Cl_2_ and sat. NaHCO_3_. The aqueous phase was extracted with CH_2_Cl_2_. The combined organic phases were washed with brine, dried over MgSO_4_ and concentrated *in vacuo*. The residue was purified by flash column chromatography (1:10 MeOH:CH_2_Cl_2_) on silica gel to give amide **7** (10–20% yield) as a white solid.

#### 4.1.7. General Procedure B for synthesis of amides 11

To a solution of aniline **8** (1.20 equiv) and carboxylic acid **9** or **10** (1.00 equv) in DMF (0.100 M) was added HATU (2.00 equiv) and *i*-Pr_2_NEt (5.00 equiv) at 23 °C. The reaction mixture was stirred for 16 h at 23 °C. The reaction was diluted with CH_2_Cl_2_ and quenched by addition of sat. NaHCO_3_. The phases were separated and the aqueous phase was extracted with EtOAc. The combined organic layer was washed with brine, dried over MgSO_4_, and concentrated *in vacuo*. The residue was purified by column chromatography (1:20 MeOH:CH_2_Cl_2_) on silica gel to afford amide **11** (10–20% yield).

**Note:** Spectra of compounds **7** were also acquired in methanol-d or acetone-d. The integrations of the major and minor peaks changed, providing evidence that species are indeed conformers/rotamers.

#### 4.1.8. (S)-N-(1-(((S)-1-(dimethylamino)-4-methyl-1-oxopentan-2-yl)amino)-2-methyl-1-oxopropan-2-yl)-1-((4-methyl-3-(1-methyl-7-((6-methylpyridin-3-yl)amino)-2-oxo-1,4-dihydropyrimido[4,5-d]pyrimidin-3(2H)-yl)benzoyl)-L-valyl)pyrrolidine-2-carboxamide (7a)

(Due to the distinct presence of rotameric isomers, the ^1^H NMR and ^13^C NMR contained extra peaks. See the attached spectrum in the supporting information) (Purity: 99%; HPLC); *R_f_:* 0.35 (1:10 = MeOH:CH_2_Cl_2_); [α]_D_^28^ = −102 (*c* 0.0590, MeOH);) ^1^H NMR (400 MHz, DMSO) δ 9.64 (s, 1H), 8.79 (d, *J* = 2.7 Hz, 1H), 8.42 (d, *J* = 8.0 Hz, 1H), 8.14 (s, 1H), 8.05 (dd, *J* = 8.5, 2.7 Hz, 1H), 8.00 (s, 1H), 7.93 (dd, *J* = 22.4, 1.8 Hz, 1H), 7.87 − 7.77 (m, 1H), 7.40 (d, *J* = 8.0 Hz, 1H), 7.27 (d, *J* = 8.5 Hz, 1H), 7.17 (d, *J* = 8.5 Hz, 1H), 4.86 − 4.66 (m, 2H), 4.56 − 4.44 (m, 2H), 4.28 (dd, *J* = 7.8, 4.8 Hz, 1H), 3.89 (d, *J* = 9.6 Hz, 1H), 3.63 (t, *J* = 8.2 Hz, 1H), 3.32 (s, 3H), 2.98 (s, 3H), 2.78 (s, 3H), 2.40 (s, 3H), 2.20 (s, 3H), 2.15 (d, *J* = 23.1 Hz, 1H), 2.07 − 1.91 (m, 2H), 1.86 (q, *J* = 5.6 Hz, 2H), 1.50 (dq, *J* = 11.4, 5.7, 5.1 Hz, 1H), 1.46 − 1.40 (m, 1H), 1.40 − 1.37 (m, 1H), 1.35 (s, 3H), 1.30 (s, 3H), 1.02 − 0.92 (m, 6H), 0.85 (dd, *J* = 11.2, 6.3 Hz, 6H); ^13^C NMR (101 MHz, DMSO) δ 173.25, 171.11, 170.96, 170.19, 170.14, 165.56, 165.39, 158.93, 156.93, 153.29, 153.22, 152.17, 152.12, 150.23, 141.10, 141.05, 140.04, 139.26, 134.68, 132.84, 132.74, 130.62, 126.84, 126.78, 126.56, 126.50, 126.24, 122.46, 102.91, 59.92, 59.88, 56.76, 55.99, 47.32, 46.69, 46.57, 40.91, 36.41, 35.13, 29.87, 28.88, 28.21, 25.41, 25.38, 24.70, 24.68, 24.59, 24.07, 23.23, 22.99, 22.97, 21.94, 21.92, 19.14, 19.06, 19.01, 17.28, 17.26; IR (Neat) 3300, 2959, 1632, 1607, 1530, 1496, 1411, 1332, 1240, 1174, 733 cm^−1^; HRMS (MM: ESI-APCI+) m/z calc’d for C_43_H_60_N_11_O_6_ [M + H]^+^: 826.4728; found: 826.4734.

#### 4.1.9. (R)-N-(1-(((S)-1-(dimethylamino)-4-methyl-1-oxopentan-2-yl)amino)-2-methyl-1-oxopropan-2-yl)-1-((4-methyl-3-(1-methyl-7-((6-methylpyridin-3-yl)amino)-2-oxo-1,4-dihydropyrimido[4,5-d]pyrimidin-3(2H)-yl)benzoyl)-L-valyl)pyrrolidine-2-carboxamide (7b)

(Due to the distinct presence of rotameric isomers, the ^1^H NMR and ^13 ^C NMR contained extra peaks. See the attached spectrum in the supporting information) (Purity: 96%; HPLC); *R_f_*: 0.34 (1:10 = MeOH:CH_2_Cl_2_); [α]_D_^30^ = −176.0 (c 0.00063, MeOH); ^1^H NMR (400 MHz, DMSO) δ 9.64 (d, *J* = 7.0 Hz, 2H), 8.79 (t, *J* = 2.2 Hz, 2H), 8.71 − 8.46 (m, 2H), 8.18 − 8.11 (m, 2H), 8.05 (dd, *J* = 8.5, 2.7 Hz, 2H), 8.00 − 7.87 (m, 3H), 7.86 − 7.70 (m, 3H), 7.41 (dt, *J* = 21.0, 8.1 Hz, 4H), 7.18 (d, *J* = 8.5 Hz, 2H), 4.84 − 4.59 (m, 4H), 4.56 − 4.36 (m, 4H), 4.30 (d, *J* = 10.6 Hz, 2H), 3.85 − 3.52 (m, 5H), 3.05 − 2.79 (m, 6H), 2.66 (d, *J* = 23.7 Hz, 6H), 2.40 (s, 6H), 2.21 − 2.16 (m, 6H), 2.06 − 1.93 (m, 3H), 1.87 (s, 3H), 1.61 (t, *J* = 8.8 Hz, 3H), 1.39 (d, *J* = 34.3 Hz, 3H), 1.32 (d, *J* = 5.0 Hz, 2H), 1.28 (d, *J* = 5.6 Hz, 6H), 1.23 (s, 6H), 1.03 (d, *J* = 6.1 Hz, 7H), 0.95 (t, *J* = 6.7 Hz, 9H), 0.90 − 0.86 (m, 3H), 0.84 (dt, *J* = 7.7, 3.8 Hz, 12H).; ^13^C NMR (151 MHz, DMSO) δ 173.91, 173.79, 173.78, 173.76, 173.53, 173.50, 171.99, 171.95, 171.93, 171.82, 171.62, 171.60, 171.37, 170.53, 166.74, 159.71, 159.65, 159.29, 157.28, 157.24, 152.78, 152.49, 150.91, 150.56, 141.41, 140.38, 139.70, 135.05, 134.96, 133.53, 130.78, 126.58, 123.11, 122.83, 103.26, 60.39, 57.43, 56.37, 47.65, 47.62, 47.28, 47.13, 47.11, 47.08, 46.92, 46.79, 36.90, 35.53, 29.90, 28.90, 28.58, 26.68, 26.45, 26.23, 25.97, 25.34, 25.09, 24.79, 24.73, 24.32, 24.09, 23.75, 23.60, 23.58, 23.54, 23.52, 22.04, 21.85, 21.79, 21.73, 19.84, 19.53, 19.49, 19.27, 19.22, 17.71, 17.34; IR (Neat) 3306, 2922, 2852, 1720, 1670, 1631, 1600, 1572, 1531, 1404, 1333, 1294, 1259, 1152, 1025, 1006, 817, 756, 732, 699, 668 cm^−1^; HRMS (MM: ESI-APCI+) m/z calc’d for C_43_H_60_N_11_O_6_ [M + H]^+^: 826.4728; found: 826.4738.

#### 4.1.10. (S)-3-benzyl-N-(4-methyl-3-(1-methyl-7-((6-methylpyridin-3-yl)amino)-2-oxo-1,4-dihydropyrimido[4,5-d]pyrimidin-3(2H)-yl)phenyl)-2-oxo-5-phenyl-2,3-dihydro-1H-benzo[e][1,4]diazepine-8-carboxamide (11a)

(Purity: 96%; HPLC); *R_f_*: 0.35 (1:10 MeOH:CH_2_Cl_2_); [α]_D_^25^ = +90.7 (c 0.150, MeOH); ^1^H NMR (400 MHz, DMSO) δ 10.83 (s, 1H), 10.50 (s, 1H), 9.64 (s, 1H), 8.80 (d, *J* = 2.6 Hz, 1H), 8.16 (d, *J* = 1.6 Hz, 1H), 8.05 (dd, *J* = 8.4, 2.6 Hz, 1H), 7.83 (dd, *J* = 4.0, 2.1 Hz, 1H), 7.76 (s, 1H), 7.69 (dd, *J* = 8.2, 1.7 Hz, 1H), 7.59 (dd, *J* = 8.4, 2.2 Hz, 1H), 7.51 (dt, *J* = 8.6, 4.2 Hz, 1H), 7.44 (d, *J* = 4.4 Hz, 4H), 7.40 − 7.24 (m, 6H), 7.18 (dd, *J* = 8.0, 5.8 Hz, 2H), 4.71 (d, *J* = 14.0 Hz, 1H), 4.52 (d, *J* = 14.0 Hz, 1H), 3.73 (dd, *J* = 8.1, 5.5 Hz, 1H), 3.42 (td, *J* = 18.2, 16.0, 6.9 Hz, 2H), 3.32 (s, 3H), 2.40 (s, 3H), 2.13 (s, 3H); ^13^C NMR (101 MHz, DMSO) δ 170.58, 167.78, 164.89, 159.42, 157.49, 153.73, 152.56, 150.69, 141.65, 140.51, 139.76, 139.67, 139.01, 138.21, 135.18, 131.35, 131.25, 131.20, 130.93, 130.19, 129.78, 129.14, 128.81, 128.55, 126.71, 126.51, 122.93, 121.84, 121.21, 120.08, 119.52, 119.49, 103.42, 65.58, 47.12, 37.61, 28.71, 23.71, 17.28; IR (Neat) 3357, 2923, 2853, 1667, 1461, 1376, 1256, 1079, 746, 698, 668 cm^−1^; HRMS (MM: ESI-APCI+) m/z calc’d for C_43_H_38_N_9_O_3_ [M + H]^+^: 728.3098; found: 728.3102.

#### 4.1.11. (S)-1-benzyl-3-isobutyl-N-(4-methyl-3-(1-methyl-7-((6-methylpyridin-3-yl)amino)-2-oxo-1,4-dihydropyrimido[4,5-d]pyrimidin-3(2H)-yl)phenyl)-2-oxo-5-phenyl-2,3-dihydro-1H-benzo[e][1,4]diazepine-8-carboxamide (11b)

(Purity: 98%; HPLC); *R_f_*: 0.35 (1:10 MeOH:CH_2_Cl_2_); [α]_D_^26^ = −74.4 (c 0.0941, MeOH); ^1^H NMR (400 MHz, DMSO) δ 10.49 (s, 1H), 9.62 (s, 1H), 8.78 (d, *J* = 2.6 Hz, 1H), 8.20 (d, *J* = 1.6 Hz, 1H), 8.15 (s, 1H), 8.05 (dd, *J* = 8.4, 2.7 Hz, 1H), 7.78 (d, *J* = 2.8 Hz, 1H), 7.74 (dd, *J* = 8.2, 1.6 Hz, 1H), 7.62 − 7.57 (m, 1H), 7.53 − 7.48 (m, 1H), 7.42 (dd, *J* = 8.3, 6.7 Hz, 2H), 7.33 − 7.26 (m, 4H), 7.18 (d, *J* = 8.5 Hz, 1H), 7.13 − 7.06 (m, 3H), 6.97 − 6.93 (m, 2H), 5.57 (d, *J* = 15.6 Hz, 1H), 5.01 (d, *J* = 15.6 Hz, 1H), 4.69 (d, *J* = 14.1 Hz, 1H), 4.51 (d, *J* = 14.1 Hz, 1H), 3.74 − 3.64 (m, 2H), 3.33 (s, 3H), 2.40 (s, 3H), 2.13 (s, 3H), 1.93 − 1.83 (m, 2H), 0.94 (d, *J* = 6.2 Hz, 3H), 0.77 (d, *J* = 6.0 Hz, 3H); ^13 ^C NMR (101 MHz, DMSO) δ 169.16, 167.47, 164.31, 159.06, 157.14, 153.40, 152.29, 150.40, 141.62, 141.29, 140.10, 138.01, 137.67, 137.14, 134.83, 132.43, 131.18, 130.95, 130.65, 129.73, 129.12, 128.53, 128.43, 127.28, 127.16, 126.52, 123.83, 122.69, 122.42, 119.98, 119.45, 103.08, 61.40, 49.11, 46.76, 28.36, 24.42, 23.41, 23.28, 22.08, 16.89; IR (Neat) 3285, 3031, 2952, 2867, 1661, 1597, 1576, 1530, 1507, 1491, 1466, 1408, 1319, 1293, 1231, 1185, 1143, 1119, 1078, 1029, 991, 908, 824, 784, 737, 696, 553, 518, 461, 411 cm^−1^; HRMS (MM: ESI-APCI+) m/z calc’d for C_47_H_46_N_9_O_3_ [M + H]^+^: 784.3724; found: 784.3715.

#### 4.1.12. (S)-3-methyl-N-(4-methyl-3-(1-methyl-7-((6-methylpyridin-3-yl)amino)-2-oxo-1,4-dihydropyrimido[4,5-d]pyrimidin-3(2H)-yl)phenyl)-2-oxo-5-phenyl-2,3-dihydro-1H-benzo[e][1,4]diazepine-8-carboxamide (11c)

(Purity: 97%; HPLC); *R_f_:* 0.35 (1:10 MeOH:CH_2_Cl_2_); [α]_D_^26^ = +40.5 (*c* 0.124, MeOH); ^1^H NMR (400 MHz, DMSO) δ 10.76 (s, 1H), 10.53 (s, 1H), 9.63 (s, 1H), 8.79 (d, *J* = 2.6 Hz, 1H), 8.16 (d, *J* = 1.4 Hz, 1H), 8.05 (dd, *J* = 8.4, 2.7 Hz, 1H), 7.84 (t, *J* = 2.1 Hz, 1H), 7.78 (d, *J* = 1.7 Hz, 1H), 7.72 (dd, *J* = 8.2, 1.7 Hz, 1H), 7.62 (dd, *J* = 8.3, 2.2 Hz, 1H), 7.49 (dt, *J* = 7.0, 2.4 Hz, 3H), 7.48 − 7.38 (m, 4H), 7.31 (d, *J* = 8.5 Hz, 1H), 7.17 (d, *J* = 8.5 Hz, 1H), 4.71 (d, *J* = 14.0 Hz, 1H), 4.53 (d, *J* = 14.0 Hz, 1H), 3.70 (q, *J* = 6.3 Hz, 1H), 3.34 (s, 3H), 2.40 (s, 3H), 2.13 (s, 3H), 1.55 (d, *J* = 6.4 Hz, 3H); ^13^C NMR (101 MHz, DMSO) δ 171.34, 167.14, 164.49, 158.97, 157.04, 153.28, 152.13, 150.25, 141.19, 140.04, 139.39, 138.62, 137.76, 137.65, 134.73, 130.90, 130.80, 130.66, 130.35, 129.31, 128.93, 128.33, 126.29, 122.50, 121.26, 120.73, 119.68, 119.11, 102.97, 58.67, 46.67, 28.26, 23.23, 17.25, 16.82; IR (Neat) 3268, 3055, 2930, 2854, 1381, 1600, 1576, 1534, 1508, 1446, 1412, 1321, 1294, 1234, 1216, 1187, 1145, 1120, 1031, 949, 844, 784, 735, 698, 657, 557, 457 cm^−1^; HRMS (MM: ESI-APCI+) m/z calc’d for C_37_H_33_N_9_O_3_Na [M + Na]^+^: 674.2604; found: 674.2606.

#### 4.1.13. (S)-1-benzyl-3-methyl-N-(4-methyl-3-(1-methyl-7-((6-methylpyridin-3-yl)amino)-2-oxo-1,4-dihydropyrimido[4,5-d]pyrimidin-3(2H)-yl)phenyl)-2-oxo-5-phenyl-2,3-dihydro-1H-benzo[e][1,4]diazepine-8-carboxamide (11d)

(Purity: 97%; HPLC); *R_f_:* 0.35 (1:10 MeOH:CH_2_Cl_2_); [α]_D_^30^ = −3.70 (*c* 0.172, MeOH); ^1^H NMR (600 MHz, DMSO) δ 10.47 (s, 1H), 9.64 (s, 1H), 8.80 (d, *J* = 2.7 Hz, 1H), 8.21 (d, *J* = 1.7 Hz, 1H), 8.16 (s, 1H), 8.06 (dd, *J* = 8.4, 2.7 Hz, 1H), 7.81 (dd, *J* = 4.7, 2.2 Hz, 1H), 7.75 (dd, *J* = 8.1, 1.7 Hz, 1H), 7.61 (ddd, *J* = 7.3, 4.6, 2.1 Hz, 1H), 7.51 (t, *J* = 7.4 Hz, 1H), 7.42 (t, *J* = 7.6 Hz, 2H), 7.31 (dq, *J* = 6.7, 2.4, 1.6 Hz, 4H), 7.18 (d, *J* = 8.5 Hz, 1H), 7.15 − 7.08 (m, 3H), 7.00 − 6.95 (m, 2H), 5.58 (d, *J* = 15.6 Hz, 1H), 5.04 (d, *J* = 15.6 Hz, 1H), 4.70 (d, *J* = 14.0 Hz, 1H), 4.53 (d, *J* = 14.0 Hz, 1H), 3.86 (q, *J* = 6.2 Hz, 1H), 3.34 (s, 3H), 2.41 (s, 3H), 2.14 (s, 3H), 1.61 (d, *J* = 6.3 Hz, 3H); ^13^C NMR (151 MHz, DMSO) δ 169.71, 167.06, 164.08, 158.93, 157.02, 153.24, 152.11, 150.17, 141.48, 141.18, 139.94, 137.86, 137.59, 137.45, 137.08, 134.72, 132.49, 130.96, 130.78, 130.44, 129.61, 129.00, 128.38, 128.25, 127.10, 127.04, 126.29, 123.54, 122.49, 122.28, 119.79, 119.29, 102.95, 58.37, 53.60, 48.93, 46.63, 41.84, 28.23, 23.18, 18.08, 17.48, 16.79, 16.72; IR (Neat) 3301, 2918, 2850, 1725, 1670, 1598, 1532, 1507, 1494, 1446, 1411, 1377, 1290, 1263, 1187, 1144, 1120, 1028, 963, 895, 822, 805, 785, 733, 698, 660, 555, 504, 460 cm^−1^; HRMS (MM: ESI-APCI+) m/z calc’d for C_44_H_39_N_9_O_3_Na [M + Na]^+^: 764.3074; found: 764.3049.

#### 4.1.14. (R)-3-((R)-s-butyl)-N-(4-methyl-3-(1-methyl-7-((6-methylpyridin-3-yl)amino)-2-oxo-1,4-dihydropyrimido[4,5-d]pyrimidin-3(2H)-yl)phenyl)-2-oxo-5-phenyl-2,3-dihydro-1H-benzo[e][1,4]diazepine-8-carboxamide (11e)

(Purity: 96%; HPLC); *R_f_:* 0.35 (1:10 MeOH:CH_2_Cl_2_); [α]_D_^28^ = −26.2 (*c* 0.0765, MeOH); ^1^H NMR (400 MHz, DMSO) δ 10.78 (s, 1H), 10.53 (s, 1H), 9.64 (s, 1H), 8.80 (d, *J* = 2.7 Hz, 1H), 8.16 (d, *J* = 1.7 Hz, 1H), 8.05 (dd, *J* = 8.5, 2.7 Hz, 1H), 7.85 (dd, *J* = 4.5, 2.2 Hz, 1H), 7.79 (d, *J* = 1.7 Hz, 1H), 7.72 (dd, *J* = 8.2, 1.7 Hz, 1H), 7.62 (dd, *J* = 8.3, 2.2 Hz, 1H), 7.54 − 7.41 (m, 6H), 7.31 (d, *J* = 8.4 Hz, 1H), 7.18 (d, *J* = 8.5 Hz, 1H), 4.72 (d, *J* = 13.9 Hz, 1H), 4.53 (d, *J* = 14.0 Hz, 1H), 3.34 (s, 3H), 3.13 (d, *J* = 9.5 Hz, 1H), 2.41 (s, 3H), 2.14 (s, 3H), 2.00 − 1.90 (m, 1H), 1.31 − 1.21 (m, 2H), 0.97 (d, *J* = 6.6 Hz, 3H), 0.91 (t, *J* = 7.4 Hz, 3H); ^13^C NMR (101 MHz, DMSO) δ 169.69, 167.45, 164.96, 159.43, 157.49, 154.35, 153.76, 152.57, 150.69, 141.65, 140.52, 139.77, 139.17, 138.24, 135.18, 131.33, 131.25, 131.13, 130.86, 129.77, 129.12, 128.84, 126.71, 122.92, 121.91, 121.09, 119.53, 103.43, 47.12, 40.63, 40.43, 40.22, 40.01, 39.80, 39.59, 39.38, 35.04, 28.71, 24.97, 23.70, 17.28, 16.53, 11.24; IR (Neat) 3239, 2957, 2924, 2854, 1688, 1658, 1600, 1577, 1534, 1508, 1494, 1465, 1412, 1321, 1296, 1231, 1188, 1145, 1120, 1033, 842, 785, 738, 698, 658, 558, 411 cm^−1^; HRMS (MM: ESI-APCI+) m/z calc’d for C_40_H_39_N_9_O_3_Na [M + Na]^+^: 716.3074; found: 716.3068.

#### 4.1.15. (R)-N-(1-(((R)-1-(dimethylamino)-4-methyl-1-oxopentan-2-yl)amino)-2-methyl-1-oxopropan-2-yl)-1-((4-methyl-3-(1-methyl-7-((6-methylpyridin-3-yl)amino)-2-oxo-1,4-dihydropyrimido[4,5-d]pyrimidin-3(2H)-yl)benzoyl)-D-valyl)pyrrolidine-2-carboxamide (7c)

(Due to the distinct presence of rotameric isomers, the ^1^H NMR and ^13 ^C NMR contained extra peaks. See the attached spectrum in the supporting information) (Purity: 95%; HPLC); *R_f_:* 0.35 (1:10 MeOH:CH_2_Cl_2_); [α]_D_^27^ = +48.6 (*c* 0.0410, MeOH); ^1^H NMR (400 MHz, DMSO) δ 9.64 (s, 1H), 8.79 (d, *J* = 2.7 Hz, 1H), 8.42 (d, *J* = 8.0 Hz, 1H), 8.14 (s, 1H), 8.05 (dd, *J* = 8.5, 2.7 Hz, 1H), 8.00 (s, 1H), 7.93 (dd, *J* = 22.4, 1.8 Hz, 1H), 7.87 − 7.77 (m, 1H), 7.40 (d, *J* = 8.0 Hz, 1H), 7.27 (d, *J* = 8.5 Hz, 1H), 7.17 (d, *J* = 8.5 Hz, 1H), 4.86 − 4.66 (m, 2H), 4.56 − 4.44 (m, 2H), 4.28 (dd, *J* = 7.8, 4.8 Hz, 1H), 3.89 (d, *J* = 9.6 Hz, 1H), 3.63 (t, *J* = 8.2 Hz, 1H), 3.32 (s, 3H), 2.98 (s, 3H), 2.78 (s, 3H), 2.40 (s, 3H), 2.20 (s, 3H), 2.15 (d, *J* = 23.1 Hz, 1H), 2.07 − 1.91 (m, 2H), 1.86 (q, *J* = 5.6 Hz, 2H), 1.50 (dq, *J* = 11.4, 5.7, 5.1 Hz, 1H), 1.46 − 1.40 (m, 1H), 1.40 − 1.37 (m, 1H), 1.35 (s, 3H), 1.30 (s, 3H), 1.27 − 1.23 (m, 1H), 1.02 − 0.92 (m, 6H), 0.85 (dd, *J* = 11.2, 6.3 Hz, 6H); ^13^C NMR (101 MHz, DMSO) δ 173.25, 171.11, 170.96, 170.19, 170.14, 165.56, 165.39, 158.93, 156.93, 153.29, 153.22, 152.17, 152.12, 150.23, 141.10, 141.05, 140.04, 139.26, 134.68, 132.84, 132.74, 130.62, 126.84, 126.78, 126.56, 126.50, 126.24, 122.46, 102.91, 59.92, 59.88, 56.76, 55.99, 47.32, 46.69, 46.57, 40.91, 36.41, 35.13, 29.87, 28.88, 28.21, 25.41, 25.38, 24.70, 24.68, 24.59, 24.07, 23.23, 22.99, 22.97, 21.94, 21.92, 19.14, 19.06, 19.01, 17.28, 17.26; IR (Neat) 3300, 2959, 1632, 1607, 1530, 1496, 1411, 1332, 1240, 1174, 733 cm^−1^; IR (Neat) 3293, 2958, 1630, 1607, 1529, 1494, 1413, 1333, 1290, 1234, 1142, 1114, 1033, 845, 787, 735 cm^−1^; HRMS (MM: ESI-APCI+) m/z calc’d for C_43_H_60_N_11_O_6_ [M + H]^+^: 826.4728; found: 826.4733.

#### 4.1.16. (S)-N-(1-(((S)-1-(dimethylamino)-4-methyl-1-oxopentan-2-yl)carbamoyl)cyclopropyl)-1-((4-methyl-3-(1-methyl-7-((6-methylpyridin-3-yl)amino)-2-oxo-1,4-dihydropyrimido[4,5-d]pyrimidin-3(2H)-yl)benzoyl)-L-valyl)pyrrolidine-2-carboxamide (7d)

(Due to the distinct presence of rotameric isomers, the ^1^H NMR and ^13^C NMR contained extra peaks. See the attached spectrum in the supporting information) (Purity: 98%; HPLC); *R_f_:* 0.35 (1:10 MeOH:CH_2_Cl_2_); [α]_D_^27^ = −18.9 (*c* 0.0520, MeOH); ^1^H NMR (400 MHz, DMSO) δ 9.64 (s, 1H), 8.79 (d, *J* = 2.6 Hz, 1H), 8.74 (d, *J* = 2.1 Hz, 1H), 8.42 − 8.36 (m, 1H), 8.14 (d, *J* = 2.9 Hz, 1H), 8.05 (dd, *J* = 8.4, 2.7 Hz, 1H), 7.93 (dd, *J* = 24.4, 1.8 Hz, 1H), 7.84 (ddd, *J* = 8.1, 3.9, 1.8 Hz, 1H), 7.41 (dd, *J* = 8.2, 3.5 Hz, 2H), 7.18 (d, *J* = 8.5 Hz, 1H), 4.84 − 4.71 (m, 2H), 4.58 − 4.47 (m, 2H), 4.13 (t, *J* = 7.1 Hz, 1H), 3.97 − 3.87 (m, 1H), 3.68 − 3.57 (m, 1H), 3.33 (s, 3H), 3.00 (s, 3H), 2.77 (d, *J* = 3.0 Hz, 3H), 2.40 (s, 3H), 2.20 (s, 3H), 2.07 − 1.95 (m, 2H), 1.89 − 1.71 (m, 2H), 1.57 − 1.45 (m, 2H), 1.43 − 1.34 (m, 1H), 1.33 − 1.12 (m, 3H), 0.96 (dd, *J* = 6.7 Hz, 6H), 0.85 − 0.80 (m, 8H).; ^13^C NMR (101 MHz, DMSO) δ 173.14, 171.52, 171.02, 170.59, 170.53, 166.05, 165.89, 159.41, 157.42, 153.76, 153.69, 152.62, 152.59, 150.71, 141.61, 141.54, 140.51, 139.78, 135.16, 133.32, 133.21, 131.11, 127.31, 127.06, 126.91, 126.72, 122.94, 103.40, 60.61, 60.56, 57.15, 57.09, 47.89, 47.53, 47.04, 41.32, 36.98, 35.64, 34.10, 30.30, 29.36, 28.68, 25.28, 24.56, 24.54, 23.70, 23.33, 23.29, 22.50, 22.46, 19.74, 19.69, 19.42, 19.29, 17.76, 16.86, 16.21; IR (Neat) 3296, 2957, 2871, 1627, 1606, 1575, 1525, 1492, 1410, 1332, 1290, 1233, 1195, 1140, 1112, 1071, 1034, 938, 831, 786, 731, 700, 621, 559, 516, 457 cm^−1^; HRMS (MM: ESI-APCI+) m/z calc’d for C_43_H_57_N_11_O_6_Na [M + Na]^+^: 846.4391; found: 846.4385.

#### 4.1.17. (R)-N-(1-(((S)-1-(dimethylamino)-4-methyl-1-oxopentan-2-yl)carbamoyl)cyclopropyl)-1-((4-methyl-3-(1-methyl-7-((6-methylpyridin-3-yl)amino)-2-oxo-1,4-dihydropyrimido[4,5-d]pyrimidin-3(2H)-yl)benzoyl)-L-valyl)pyrrolidine-2-carboxamide (7e)

(Due to the distinct presence of rotameric isomers, the ^1^H NMR and ^13^C NMR contained extra peaks. See the attached spectrum in the supporting information) (Purity: 98%; HPLC); *R_f_:* 0.35 (1:10 MeOH:CH_2_Cl_2_); [α]_D_^31^ = −150.0 (*c* 0.0530, MeOH); ^1^H NMR (600 MHz, DMSO) δ 9.63 (d, *J* = 8.3 Hz, 1H), 8.79 (t, *J* = 2.8 Hz, 1H), 8.65 − 8.42 (m, 2H), 8.10 (d, *J* = 21.1 Hz, 1H), 8.05 (dd, *J* = 8.5, 2.8 Hz, 1H), 7.90 − 7.81 (m, 2H), 7.80 − 7.73 (m, 1H), 7.36 (dd, *J* = 15.5, 8.0 Hz, 1H), 7.18 (d, *J* = 8.5 Hz, 1H), 4.75 (td, *J* = 19.3, 17.8, 10.8 Hz, 2H), 4.63 (dt, *J* = 25.8, 9.0 Hz, 1H), 4.40 (dd, *J* = 87.9, 14.0 Hz, 1H), 4.23 − 4.17 (m, 1H), 3.68 (d, *J* = 6.9 Hz, 2H), 3.33 (s, 3H), 3.00 (d, *J* = 34.1 Hz, 3H), 2.60 (d, *J* = 42.9 Hz, 3H), 2.40 (s, 3H), 2.17 (d, *J* = 14.0 Hz, 4H), 2.11 − 2.03 (m, 1H), 1.97 (m, 1H), 1.86 (m, 1H), 1.79 (m, 1H), 1.72 (m, 1H), 1.65 (m, 1H), 1.39 (m, 1H), 1.25 − 1.05 (m, 3H), 0.94 (d, *J* = 7.4 Hz, 6H), 0.86 (m, 6H), 0.81 − 0.74 (m, 2H); ^13^C NMR (151 MHz, DMSO) δ 173.34, 173.14, 172.13, 172.08, 171.12, 170.99, 170.53, 170.49, 167.06, 166.18, 159.29, 159.27, 157.29, 157.26, 153.66, 152.47, 152.38, 150.59, 141.43, 141.32, 140.41, 139.60, 139.14, 135.06, 134.04, 133.30, 130.83, 130.65, 127.00, 126.61, 122.82, 103.37, 60.79, 60.69, 57.03, 56.85, 47.64, 47.32, 47.23, 46.89, 46.69, 40.96, 40.69, 36.93, 35.53, 35.34, 34.05, 30.21, 28.87, 28.77, 28.58, 24.98, 24.95, 24.32, 23.61, 23.59, 23.54, 23.49, 21.82, 19.64, 19.57, 19.00, 18.92, 17.72, 17.70, 16.27, 16.20, 16.00, 15.95; IR (Neat) 3294, 2957, 2923, 2853, 2360, 2340, 2628, 1574, 1533, 1496, 1414, 1333, 1294, 1236, 1149, 1115, 1026, 1007, 821, 753 cm^−1^; HRMS (MM: ESI-APCI+) m/z calc’d for C_43_H_58_N_11_O_6_ [M + H]^+^: 824.4572; found: 824.4574.

#### 4.1.18. (S)-N-(1-(((S)-1-(dimethylamino)-4-methyl-1-oxopentan-2-yl)carbamoyl)cyclobutyl)-1-((4-methyl-3-(1-methyl-7-((6-methylpyridin-3-yl)amino)-2-oxo-1,4-dihydropyrimido[4,5-d]pyrimidin-3(2H)-yl)benzoyl)-L-valyl)pyrrolidine-2-carboxamide (7f)

(Due to the distinct presence of rotameric isomers, the ^1^H NMR and ^13^C NMR contained extra peaks. See the attached spectrum in the supporting information) (Purity: 96%; HPLC); *R_f_:* 0.35 (1:10 = MeOH:CH_2_Cl_2_); [α]_D_^31^ = −56.7 (*c* 0.0530, MeOH); ^1^H NMR (400 MHz, DMSO) δ 9.68 (s, 1H), 8.81 (d, *J* = 2.7 Hz, 1H), 8.49 (s, 1H), 8.44 (d, *J* = 8.1 Hz, 1H), 8.15 (s, 1H), 8.07 (dd, *J* = 8.5, 2.7 Hz, 1H), 7.94 (dd, *J* = 23.1, 1.8 Hz, 1H), 7.88 − 7.78 (m, 1H), 7.40 (d, *J* = 8.1 Hz, 1H), 7.19 (dd, *J* = 8.7, 5.2 Hz, 2H), 4.84 − 4.71 (m, 2H), 4.56 − 4.48 (m, 2H), 4.26 (dd, *J* = 7.9, 5.3 Hz, 1H), 3.89 (ddt, *J* = 13.0, 8.8, 4.2 Hz, 1H), 3.64 (dq, *J* = 10.9, 5.4, 3.9 Hz, 1H), 3.33 (d, *J* = 2.4 Hz, 3H), 2.99 (s, 3H), 2.78 (d, *J* = 3.2 Hz, 3H), 2.41 (s, 3H), 2.40 − 2.33 (m, 1H), 2.20 (s, 3H), 2.17 (dd, *J* = 8.1, 5.4 Hz, 1H), 2.12 − 1.92 (m, 4H), 1.84 (ddt, *J* = 20.9, 14.2, 7.0 Hz, 4H), 1.53 − 1.40 (m, 2H), 1.38 − 1.31 (m, 1H), 1.26 (t, *J* = 6.1 Hz, 1H), 0.99 − 0.93 (m, 6H), 0.86 (dd, *J* = 16.6, 6.2 Hz, 6H); ^13^C NMR (101 MHz, DMSO) δ 172.72, 171.72, 171.59, 170.64, 170.59, 166.04, 165.87, 159.37, 157.42, 153.75, 153.69, 152.63, 152.58, 150.52, 141.58, 141.53, 140.14, 139.74, 135.27, 133.32, 133.22, 131.09, 127.32, 127.26, 127.04, 126.99, 123.10, 103.45, 60.26, 60.22, 58.87, 57.20, 54.08, 47.81, 47.04, 41.63, 36.89, 35.59, 31.13, 30.79, 30.33, 29.44, 28.68, 25.11, 25.09, 24.52, 23.52, 23.48, 23.46, 22.35, 22.33, 19.67, 19.65, 19.45, 19.40, 18.55, 17.74, 17.20, 15.82; IR (Neat) 3288, 2955, 2360, 2339, 1606, 1527, 1493, 1411, 1331, 1288, 1233, 1196, 1140, 1113, 1033, 953, 843, 787, 732 cm^−1^; HRMS (MM: ESI-APCI+) m/z calc’d for C_44_H_60_N_11_O_6_ [M + H]^+^: 838.4728; found: 838.4726.

#### 4.1.19. (S)-N-(2-(((S)-1-(dimethylamino)-4-methyl-1-oxopentan-2-yl)amino)-2-oxoethyl)-1-((4-methyl-3-(1-methyl-7-((6-methylpyridin-3-yl)amino)-2-oxo-1,4-dihydropyrimido[4,5-d]pyrimidin-3(2H)-yl)benzoyl)-L-valyl)pyrrolidine-2-carboxamide (7g)

(Due to the distinct presence of rotameric isomers, the ^1^H NMR and ^13^C NMR contained extra peaks. See the attached spectrum in the supporting information) (Purity: 95%; HPLC); *R_f_:* 0.35 (1:10 MeOH:CH_2_Cl_2_); [α]_D_^30^ = −12.1 (*c* 0.0820, MeOH); ^1^H NMR (600 MHz, DMSO) δ 9.75 (s, 1H), 8.85 (s, 1H), 8.45 (dd, *J* = 8.2, 3.5 Hz, 1H), 8.19 (t, *J* = 5.1 Hz, 1H), 8.16 (s, 1H), 8.13 − 8.07 (m, 1H), 7.93 (d, *J* = 28.2 Hz, 1H), 7.86 (d, *J* = 8.5 Hz, 1H), 7.83 (t, *J* = 8.5 Hz, 1H), 7.40 (d, *J* = 7.9 Hz, 1H), 7.26 (d, *J* = 8.5 Hz, 1H), 4.81 − 4.75 (m, 2H), 4.52 (dt, *J* = 17.3, 6.5 Hz, 2H), 4.32 (dd, *J* = 8.0, 4.6 Hz, 1H), 3.90 (dt, *J* = 10.1, 5.8 Hz, 1H), 3.71 − 3.65 (m, 3H), 3.34 (d, *J* = 3.2 Hz, 3H), 3.02 (s, 3H), 2.80 (s, 3H), 2.43 (s, 3H), 2.20 (s, 3H), 2.17 (t, *J* = 7.3 Hz, 1H), 2.07 − 2.03 (m, 1H), 1.98 − 1.94 (m, 1H), 1.85 (t, *J* = 6.0 Hz, 1H), 1.55 − 1.51 (m, 1H), 1.41 (s, 1H), 1.27 − 1.25 (m, 2H), 0.99 − 0.93 (m, 6H), 0.86 (d, *J* = 6.6 Hz, 6H); ^13^C NMR (151 MHz, DMSO) δ 172.22, 171.61, 170.48, 170.43, 168.86, 168.62, 165.86, 165.71, 161.57, 159.16, 157.34, 153.60, 152.50, 152.44, 149.97, 141.44, 141.39, 139.62, 139.10, 135.44, 133.18, 133.08, 130.98, 127.56, 127.21, 126.89, 123.46, 103.52, 63.15, 61.03, 60.28, 59.96, 59.92, 57.12, 57.09, 53.93, 47.81, 47.61, 46.92, 46.86, 42.16, 41.07, 36.88, 35.53, 30.84, 30.21, 30.18, 29.65, 29.40, 28.60, 25.11, 24.88, 24.86, 24.48, 23.46, 23.44, 23.38, 23.37, 23.03, 22.04, 22.02, 22.00, 19.53, 19.49, 19.44, 19.40, 19.34, 19.10, 18.43, 17.82, 17.65, 17.63, 17.09, 12.83;); IR (Neat) 3291, 2958, 1739, 1630, 1574, 1533, 1496, 1415, 1336, 1291, 1232, 1142, 1115, 1033, 844, 787, 736 cm^−1^; HRMS (MM: ESI-APCI+) m/z calc’d for C_41_H_56_N_11_O_6_ [M + H]^+^: 798.4415; found: 798.4420.

#### 4.1.20. (R)-N-(2-(((S)-1-(dimethylamino)-4-methyl-1-oxopentan-2-yl)amino)-2-oxoethyl)-1-((4-methyl-3-(1-methyl-7-((6-methylpyridin-3-yl)amino)-2-oxo-1,4-dihydropyrimido[4,5-d]pyrimidin-3(2H)-yl)benzoyl)-L-valyl)pyrrolidine-2-carboxamide (7h)

(Due to the distinct presence of rotameric isomers, the ^1^H NMR and ^13^C NMR contained extra peaks. See the attached spectrum in the supporting information) (Purity: 98%; HPLC); *R_f_:* 0.35 (1:10 MeOH:CH_2_Cl_2_); [α]_D_^32^ = −51.0 (*c* 0.0590, MeOH); ^1^H NMR (600 MHz, DMSO) δ 9.65 (s, 1H), 8.79 (s, 1H), 8.48 (dd, *J* = 16.2, 8.2 Hz, 1H), 8.14 (d, *J* = 7.4 Hz, 1H), 8.05 (dd, *J* = 8.5, 2.5 Hz, 2H), 7.92 − 7.88 (m, 1H), 7.86(dd, *J* = 8.5, 2.0 Hz, 1H), 7.82 − 7.80 (m, 1H), 7.39 − 7.37 (m, 1H), 7.18 (s, 1H), 4.78 − 4.75 (m, 2H), 4.57 − 4.55 (m, 1H), 4.52 − 4.48 (m, 1H), 4.45 (d, *J* = 14.0 Hz, 1H), 4.31 (dt, *J* = 8.2, 4.0 Hz, 1H), 3.85 − 3.77 (m, 2H), 3.67 − 3.64 (m, 2H), 3.33 (s, 3H), 3.00 (d, *J* = 11.9 Hz, 3H), 2.73 (d, *J* = 11.7 Hz, 3H), 2.40 (s, 3H), 2.20 (d, *J* = 3.5 Hz, 3H), 1.99 (t, *J* = 6.3 Hz, 2H), 1.88 (dt, *J* = 8.6, 5.1 Hz, 2H), 1.61 − 1.55 (m, 2H), 1.35 (ddd, *J* = 8.9, 4.4, 2.1 Hz, 1H), 0.95 (d, *J* = 6.7 Hz, 3H), 0.93 (d, *J* = 6.6 Hz, 3H), 0.85 (s, 6H); ^13^C NMR (151 MHz, DMSO) δ 172.21, 172.14, 171.89, 171.73, 170.52, 168.66, 168.61, 168.46, 166.39, 166.16, 159.29, 157.29, 153.63, 152.48, 152.41, 150.60, 150.58, 141.44, 141.40, 140.39, 139.67, 139.53, 135.06, 133.33, 133.12, 130.92, 130.86, 127.19, 126.96, 126.79, 126.59, 122.83, 103.32, 60.36, 57.17, 57.14, 47.50, 46.93, 46.88, 46.82, 42.32, 42.28, 41.20, 40.90, 40.79, 40.42, 36.88, 35.54, 35.52, 35.48, 31.06, 30.08, 30.06, 29.49, 29.43, 28.58, 24.55, 24.51, 24.42, 24.39, 23.60, 23.50, 21.95, 21.79, 19.54, 19.52, 19.12, 17.71, 17.68; IR (Neat) 3290, 2957, 2361, 2340, 1629, 1608, 1574, 1533, 1494, 1412, 1333, 1289, 1235, 1142, 1115, 1028, 840, 787, 736 cm^−1^; HRMS (MM: ESI-APCI+) m/z calc’d for C_41_H_56_N_11_O_6_ [M + H]^+^: 798.4415; found: 798.4425.

#### 4.1.21. (S)-N-((S)-1-(((S)-1-(dimethylamino)-4-methyl-1-oxopentan-2-yl)amino)-1-oxo-3-phenylpropan-2-yl)-1-((4-methyl-3-(1-methyl-7-((6-methylpyridin-3-yl)amino)-2-oxo-1,4-dihydropyrimido[4,5-d]pyrimidin-3(2H)-yl)benzoyl)-L-valyl)pyrrolidine-2-carboxamide (7i)

(Due to the distinct presence of rotameric isomers, the ^1^H NMR and ^13^C NMR contained extra peaks. See the attached spectrum in the supporting information) (Purity: 95%; HPLC); *R_f_:* 0.35 (1:10 MeOH:CH_2_Cl_2_); [α]_D_^29^ = −15.5 (*c* 0.130, MeOH); ^1^H NMR (400 MHz, DMSO) δ 9.64 (d, *J* = 4.8 Hz, 1H), 8.79 (d, *J* = 2.6 Hz, 1H), 8.51 (d, *J* = 8.1 Hz, 1H), 8.15 (d, *J* = 4.8 Hz, 1H), 8.09 − 7.97 (m, 2H), 7.97 − 7.87 (m, 2H), 7.81 (td, *J* = 7.7, 1.9 Hz, 1H), 7.46 − 7.33 (m, 1H), 7.27 − 7.09 (m, 6H), 4.76 (ddd, *J* = 17.7, 14.0, 9.9 Hz, 2H), 4.56 − 4.31 (m, 4H), 3.85 (dq, *J* = 11.9, 6.9, 5.9 Hz, 1H), 3.62 (dt, *J* = 11.8, 6.1 Hz, 1H), 3.32 (s, 3H), 3.02 − 2.95 (m, 1H), 2.93 (s, 3H), 2.91 − 2.82 (m, 1H), 2.80 (s, 3H), 2.40 (s, 3H), 2.20 (s, 3H), 2.15 − 2.08 (m, 1H), 1.97 (dt, *J* = 11.1, 8.0 Hz, 1H), 1.83 (tt, *J* = 13.3, 6.6 Hz, 2H), 1.54 (dq, *J* = 12.8, 6.6 Hz, 1H), 1.39 (tdd, *J* = 13.7, 9.0, 5.0 Hz, 2H), 1.18 − 1.09 (m, 1H), 1.03 − 0.90 (m, 6H), 0.91 − 0.72 (m, 6H); ^13^C NMR (101 MHz, DMSO) δ 171.76, 171.56, 170.66, 170.61, 165.96, 165.82, 159.41, 157.42, 153.73, 152.65, 152.57, 150.70, 141.56, 141.51, 140.52, 139.71, 138.01, 135.16, 133.26, 133.18, 131.07, 129.74, 129.63, 128.42, 127.32, 127.04, 126.97, 126.72, 126.65, 122.93, 103.39, 59.83, 57.32, 54.09, 47.66, 47.05, 46.95, 37.68, 36.91, 35.63, 30.33, 30.29, 29.59, 28.69, 24.76, 24.47, 23.71, 23.57, 22.15, 19.63, 19.59, 17.77, 17.74, 17.61; IR (Neat) 3388, 3300, 2955, 2924, 2854, 1728, 1672, 1460, 1415, 1377, 1260, 1018, 952, 799, 705, 667, 609, 554, 533, 498, 469, 447, 411 cm^−1^; HRMS (MM: ESI-APCI+) m/z calc’d for C_48_H_62_N_11_O_6_ [M + H]^+^: 888.4885; found: 888.4888.

#### 4.1.22. (S)-N-(1-(((S)-1-(dimethylamino)-4-methyl-1-oxopentan-2-yl)amino)-2-methyl-1-oxopropan-2-yl)-1-((4-methyl-3-(1-methyl-7-((6-methylpyridin-3-yl)amino)-2-oxo-1,4-dihydropyrimido[4,5-d]pyrimidin-3(2H)-yl)benzoyl)-L-valyl)piperidine-2-carboxamide (7j)

(Due to the distinct presence of rotameric isomers, the ^1^H NMR and ^13^C NMR contained extra peaks. See the attached spectrum in the supporting information) (Purity: 96%; HPLC); *R_f_:* 0.35 (1:10 MeOH:CH_2_Cl_2_); [α]_D_^31^ = −14.2 (*c* 0.0710, MeOH); ^1^H NMR (600 MHz, DMSO) δ 9.65 (d, *J* = 3.2 Hz, 2H), 8.79 (d, *J* = 2.7 Hz, 2H), 8.75 (dd, *J* = 10.0, 7.0 Hz, 1H), 8.47 (dd, *J* = 15.8, 8.6 Hz, 1H), 8.18 − 8.13 (m, 2H), 8.08 − 8.03 (m, 3H), 7.99 (dd, *J* = 12.1, 1.9 Hz, 1H), 7.94 (dd, *J* = 32.2, 1.9 Hz, 1H), 7.88 − 7.83 (m, 1H), 7.81 (ddd, *J* = 7.2, 4.8, 2.0 Hz, 1H), 7.65 (d, *J* = 4.2 Hz, 1H), 7.46 (t, *J* = 9.0 Hz, 1H), 7.40 (dq, *J* = 8.9, 4.9, 3.7 Hz, 3H), 7.18 (d, *J* = 8.5 Hz, 2H), 5.04 (q, *J* = 4.3, 3.8 Hz, 1H), 4.93 (d, *J* = 3.2 Hz, 1H), 4.87 − 4.76 (m, 4H), 4.73 (td, *J* = 9.6, 5.3 Hz, 2H), 4.70 − 4.62 (m, 1H), 4.56 − 4.42 (m, 3H), 4.07 (t, *J* = 13.9 Hz, 1H), 3.33 (d, *J* = 3.7 Hz, 6H), 2.99 − 2.96 (m, 6H), 2.81 − 2.76 (m, 6H), 2.70 (q, *J* = 11.7 Hz, 1H), 2.40 (s, 6H), 2.20 (d, *J* = 2.3 Hz, 6H), 2.18 − 2.15 (m, 1H), 2.10 (d, *J* = 12.2 Hz, 1H), 1.63 − 1.49 (m, 8H), 1.45 (p, *J* = 6.0, 5.5 Hz, 3H), 1.42 − 1.40 (m, 6H), 1.39 (d, *J* = 5.3 Hz, 1H), 1.37 (s, 3H), 1.34 (s, 3H), 1.31 − 1.17 (m, 4H), 0.98 (d, *J* = 6.8 Hz, 3H), 0.93 (d, *J* = 6.7 Hz, 3H), 0.90 (d, *J* = 6.6 Hz, 3H), 0.86 (d, *J* = 6.5 Hz, 3H), 0.83 (dd, *J* = 10.0, 6.4 Hz, 6H), 0.70 (t, *J* = 6.3 Hz, 3H);^13^C NMR (151 MHz, DMSO) δ 206.85, 173.70, 171.94, 171.89, 171.52, 171.44, 171.40, 171.32, 170.25, 169.31, 166.40, 166.22, 165.66, 165.49, 159.30, 159.29, 157.30, 153.59, 152.54, 152.47, 152.44, 152.39, 150.60, 150.59, 141.49, 141.47, 141.38, 140.39, 140.01, 139.63, 135.05, 133.26, 133.13, 132.45, 130.99, 127.06, 126.82, 126.61, 122.83, 103.30, 56.59, 56.54, 56.34, 56.15, 56.05, 55.26, 54.55, 52.46, 47.09, 47.06, 46.94, 43.56, 41.33, 36.77, 36.68, 35.49, 35.38, 31.06, 30.31, 30.24, 30.17, 28.58, 27.06, 26.91, 25.42, 25.31, 25.24, 25.11, 25.07, 25.04, 24.99, 24.35, 24.25, 23.59, 23.52, 22.08, 21.77, 21.73, 20.62, 20.33, 19.90, 19.87, 19.85, 19.82, 19.66, 18.94, 17.72, 17.67, 17.65, 17.63; IR (Neat) 3289, 2932, 1606, 1575, 1530, 1493, 1411, 1333, 1265, 1231, 1193, 1139, 1114, 1019, 952, 842, 787, 733 cm^−1^; HRMS (MM: ESI-APCI+) m/z calc’d for C_44_H_62_N_11_O_6_ [M + H]^+^: 840.4885; found: 840.4897.

#### 4.1.23. (S)-N-(1-(((S)-1-(dimethylamino)-4-methyl-1-oxopentan-2-yl)amino)-2-methyl-1-oxopropan-2-yl)-1-((4-methyl-3-(1-methyl-7-((6-methylpyridin-3-yl)amino)-2-oxo-1,4-dihydropyrimido[4,5-d]pyrimidin-3(2H)-yl)benzoyl)-L-valyl)azetidine-2-carboxamide (7k)

(Due to the distinct presence of rotameric isomers, the ^1^H NMR and ^13^C NMR contained extra peaks. See the attached spectrum in the supporting information) (Purity: 99%; HPLC); *R_f_:* 0.35 (1:10 MeOH:CH_2_Cl_2_); [α]_D_^28^ = −40.5 (*c* 0.0220, MeOH); ^1^H NMR (600 MHz, DMSO) δ 9.65 (s, 1H), 8.79 (d, *J* = 2.6 Hz, 1H), 8.54 (d, *J* = 8.0 Hz, 1H), 8.15 (d, *J* = 4.2 Hz, 1H), 8.10 − 8.03 (m, 2H), 7.97 − 7.89 (m, 1H), 7.82 (t, *J* = 8.5 Hz, 1H), 7.41 (d, *J* = 7.9 Hz, 1H), 7.34 (d, *J* = 8.5 Hz, 1H), 7.18 (d, *J* = 8.5 Hz, 1H), 4.78 (t, *J* = 14.8 Hz, 1H), 4.73 (td, *J* = 9.1, 4.8 Hz, 1H), 4.65 (dd, *J* = 9.0, 5.9 Hz, 1H), 4.52 (dd, *J* = 14.0, 5.7 Hz, 1H), 4.37 − 4.32 (m, 1H), 4.20 − 4.13 (m, 2H), 3.34 (d, *J* = 3.9 Hz, 3H), 2.99 (s, 3H), 2.80 (s, 3H), 2.41 (d, *J* = 8.5 Hz, 3H), 2.38 (d, *J* = 8.5 Hz, 1H), 2.20 (s, 3H), 2.17 − 2.07 (m, 2H), 1.53 (d, *J* = 13.5 Hz, 1H), 1.46 − 1.39 (m, 2H), 1.36 (s, 3H), 1.35 (s, 3H), 0.96 (dd, *J* = 15.1, 6.5 Hz, 6H), 0.86 (dd, *J* = 16.2, 6.5 Hz, 6H); ^13^C NMR (151 MHz, DMSO) δ 173.39, 171.78, 171.63, 169.45, 165.93, 165.78, 159.30, 157.31, 153.66, 152.55, 150.60, 141.46, 140.41, 139.66, 135.04, 133.02, 130.99, 127.21, 126.89, 126.61, 122.83, 103.29, 63.15, 60.81, 56.35, 54.98, 54.94, 49.19, 47.12, 46.94, 41.18, 36.78, 35.52, 29.56, 28.59, 25.40, 25.39, 25.13, 25.12, 24.39, 23.60, 23.51, 22.04, 19.61, 19.38, 17.67, 17.64; IR (Neat) 3293, 2926, 1635, 1607, 1531, 1495, 1467, 1412, 1333, 1290, 1241, 1142, 1071, 845, 787, 734 cm^−1^; HRMS (MM: ESI-APCI+) m/z calc’d for C_42_H_58_N_11_O_6_ [M + H]^+^:812.4572; found: 812.4561.

#### 4.1.24. (S)-N-(1-(((S)-1-(dimethylamino)-3-methyl-1-oxobutan-2-yl)amino)-2-methyl-1-oxopropan-2-yl)-1-((4-methyl-3-(1-methyl-7-((6-methylpyridin-3-yl)amino)-2-oxo-1,4-dihydropyrimido[4,5-d]pyrimidin-3(2H)-yl)benzoyl)-L-phenylalanyl)pyrrolidine-2-carboxamide (7l)

(Due to the distinct presence of rotameric isomers, the ^1^H NMR and ^13^C NMR contained extra peaks. See the attached spectrum in the supporting information) (Purity: 99%; HPLC); *R_f_:* 0.35 (1:10 MeOH:CH_2_Cl_2_); [α]_D_^29^ = −46.5 (*c* 0.0667, MeOH); ^1^H NMR (400 MHz, DMSO) δ 9.64 (s, 1H), 8.79 (d, *J* = 2.6 Hz, 1H), 8.42 (d, *J* = 7.3 Hz, 1H), 8.13 (s, 1H), 8.10 (s, 1H), 8.05 (dd, *J* = 8.5, 2.7 Hz, 1H), 7.96 − 7.88 (m, 1H), 7.85 − 7.78 (m, 1H), 7.41 (dd, *J* = 10.6, 8.0 Hz, 2H), 7.23 (dd, *J* = 8.0, 6.4 Hz, 2H), 7.20 − 7.14 (m, 4H), 4.88 − 4.71 (m, 2H), 4.49 (dd, *J* = 14.0, 8.9 Hz, 2H), 4.28 (t, *J* = 6.4 Hz, 1H), 3.91 (m, 1H), 3.67 − 3.56 (m, 1H), 3.33 (s, 3H), 2.96 (dd, *J* = 13.4, 7.9 Hz, 1H), 2.80 (m, 1H), 2.72 (s, 3H), 2.70 (d, *J* = 2.1 Hz, 3H), 2.40 (s, 3H), 2.19 (s, 3H), 2.17 − 2.08 (m, 1H), 2.08 − 1.90 (m, 2H), 1.83 (d, *J* = 6.2 Hz, 2H), 1.28 (d, *J* = 17.4 Hz, 6H), 0.93 (dd, *J* = 11.5, 6.6 Hz, 6H); ^13^C NMR (101 MHz, DMSO) δ 173.58, 171.55, 170.73, 166.06, 159.41, 152.59, 150.72, 140.52, 139.71, 138.04, 135.15, 131.09, 129.75, 128.47, 126.77, 122.93, 103.40, 60.34, 57.27, 56.40, 50.48, 47.82, 47.03, 38.23, 36.77, 35.56, 30.27, 29.33, 28.67, 25.88, 25.11, 23.70, 19.53, 19.48, 17.74; IR (Neat) 3298, 2924, 1628, 1605, 1576, 1528, 1493, 1448, 1411, 1332, 1288, 1294, 1235, 1194, 1142, 1112, 1075, 1031, 943, 830, 787, 732, 700, 623, 548, 514, 480, 458 cm^−1^; HRMS (MM: ESI-APCI+) m/z calc’d for C_46_H_58_N_11_O_6_ [M + H]^+^: 860.4572; found: 860.4589.

#### 4.1.25. (S)-N-(1-(((S)-1-(dimethylamino)-3-methyl-1-oxobutan-2-yl)amino)-2-methyl-1-oxopropan-2-yl)-1-((4-methyl-3-(1-methyl-7-((6-methylpyridin-3-yl)amino)-2-oxo-1,4-dihydropyrimido[4,5-d]pyrimidin-3(2H)-yl)benzoyl)-L-alanyl)pyrrolidine-2-carboxamide (7m)

(Due to the distinct presence of rotameric isomers, the ^1^H NMR and ^13^C NMR contained extra peaks. See the attached spectrum in the supporting information) (Purity: 96%; HPLC); *R_f_:* 0.35 (1:10 = MeOH:CH_2_Cl_2_); [α]_D_^30^ = −42.0 (*c* 0.0667, MeOH); ^1^H NMR (400 MHz, DMSO) δ 9.62 (s, 1H), 8.79 (d, *J* = 2.6 Hz, 1H), 8.41 (d, *J* = 8.0 Hz, 1H), 8.15 (d, *J* = 4.6 Hz, 2H), 8.05 (dd, *J* = 8.5, 2.7 Hz, 1H), 7.95 − 7.88 (m, 1H), 7.85 − 7.78 (m, 1H), 7.40 (d, *J* = 7.9 Hz, 1H), 7.37 (d, *J* = 4.8 Hz, 1H), 7.24 (d, *J* = 7.6 Hz, 1H), 7.18 (d, *J* = 8.4 Hz, 1H), 4.78 (dd, *J* = 14.0, 6.5 Hz, 1H), 4.63 (t, *J* = 7.0 Hz, 1H), 4.54 − 4.48 (m, 1H), 4.46 (d, *J* = 7.6 Hz, 1H), 4.28 (dd, *J* = 7.6, 5.6 Hz, 1H), 3.96 (d, *J* = 9.9 Hz, 1H), 3.62 (d, *J* = 8.8 Hz, 1H), 3.51 (d, *J* = 24.5 Hz, 2H), 3.33 (s, 3H), 2.97 (d, *J* = 1.7 Hz, 3H), 2.78 (s, 3H), 2.41 (s, 3H), 2.20 (s, 3H), 2.17 − 2.10 (m, 1H), 2.06 − 1.91 (m, 2H), 1.82 (td, *J* = 11.9, 11.4, 6.3 Hz, 2H), 1.34 (s, 3H), 1.28 (s, 3H), 1.14 (d, *J* = 6.8 Hz, 3H), 0.95 (t, *J* = 6.5 Hz, 6H); ^13^C NMR (101 MHz, DMSO) δ 173.38, 171.98, 171.96, 171.61, 170.61, 166.06, 159.41, 157.43, 152.65, 150.72, 141.53, 140.52, 139.72, 135.15, 133.30, 133.04, 132.85, 131.09, 129.33, 129.03, 127.23, 127.00, 126.75, 122.93, 103.41, 60.30, 57.38, 56.33, 51.77, 47.87, 47.05, 45.10, 36.84, 35.65, 31.15, 30.20, 29.40, 28.68, 26.48, 25.15, 24.61, 23.69, 19.63, 19.42, 17.74; IR (Neat) 3294, 2962, 2965, 1735, 1632, 1609, 1575, 1531, 1495, 1414, 1333, 1291, 1236, 1196, 1143, 1114, 1034, 947, 832, 787, 740, 699, 625, 598, 513, 452, 418 cm^−1^; HRMS (MM: ESI-APCI+) m/z calc’d for C_40_H_54_N_11_O_6_ [M + H]^+^:784.4259; found: 784.4248.

#### 4.1.26. (S)-N-(1-(((S)-1-(dimethylamino)-3-methyl-1-oxobutan-2-yl)amino)-2-methyl-1-oxopropan-2-yl)-1-((4-methyl-3-(1-methyl-7-((6-methylpyridin-3-yl)amino)-2-oxo-1,4-dihydropyrimido[4,5-d]pyrimidin-3(2H)-yl)benzoyl)-L-valyl)pyrrolidine-2-carboxamide (7n)

(Due to the distinct presence of rotameric isomers, the ^1^H NMR and ^13^C NMR contained extra peaks. See the attached spectrum in the supporting information) (Purity: 96%; HPLC); *R_f_:* 0.35 (1:10 MeOH:CH_2_Cl_2_); [α]_D_^29^ = −90.7 (*c* 0.0882, MeOH); ^1^H NMR (400 MHz, DMSO) δ 9.64 (s, 1H), 8.79 (d, *J* = 2.7 Hz, 1H), 8.48 − 8.41 (m, 1H), 8.15 (d, *J* = 1.7 Hz, 1H), 8.08 − 8.02 (m, 2H), 7.93 (dd, *J* = 20.3, 1.9 Hz, 1H), 7.82 (ddd, *J* = 8.0, 3.9, 1.8 Hz, 1H), 7.40 (d, *J* = 8.0 Hz, 1H), 7.18 (d, *J* = 8.5 Hz, 1H), 7.08 (dd, *J* = 9.0, 5.7 Hz, 1H), 4.79 (dd, *J* = 14.1, 8.7 Hz, 1H), 4.54 − 4.49 (m, 2H), 4.29 (td, *J* = 8.9, 8.2, 3.6 Hz, 1H), 3.90 (s, 1H), 3.65 (d, *J* = 7.5 Hz, 1H), 3.33 (s, 3H), 3.00 (t, *J* = 1.3 Hz, 3H), 2.80 (d, *J* = 0.9 Hz, 3H), 2.40 (s, 3H), 2.20 (s, 3H), 2.14 (d, *J* = 13.7 Hz, 1H), 1.97 (dt, *J* = 13.7, 7.2 Hz, 3H), 1.85 (d, *J* = 6.5 Hz, 3H), 1.36 (s, 3H), 1.29 (d, *J* = 2.2 Hz, 3H), 0.94 (dd, *J* = 6.7, 3.0 Hz, 6H), 0.81 (d, *J* = 6.6 Hz, 3H), 0.78 − 0.75 (m, 3H); ^13^C NMR (101 MHz, DMSO) δ 173.92, 171.63, 171.13, 170.61, 153.71, 150.72, 141.54, 140.52, 135.16, 131.09, 127.32, 127.00, 126.73, 122.94, 60.20, 57.32, 56.54, 53.68, 47.74, 47.05, 37.18, 35.46, 30.81, 30.30, 29.45, 28.68, 25.91, 25.27, 24.99, 23.70, 19.95, 19.59, 18.26, 17.75; IR (Neat) 3239, 2957, 2924, 2854, 1688, 1658, 1600, 1577, 1534, 1508, 1494, 1465, 1412, 1321, 1296, 1231, 1188, 1145, 1120, 1033, 842, 785, 738, 698, 659, 558, 464, 411 cm^−1^; HRMS (MM: ESI-APCI+) m/z calc’d for C_42_H_58_N_11_O_6_ [M + H]^+^: 812.4572; found: 812.4582.

#### 4.1.27. (R)-N-(1-(((S)-1-(dimethylamino)-3-methyl-1-oxobutan-2-yl)amino)-2-methyl-1-oxopropan-2-yl)-1-((4-methyl-3-(1-methyl-7-((6-methylpyridin-3-yl)amino)-2-oxo-1,4-dihydropyrimido[4,5-d]pyrimidin-3(2H)-yl)benzoyl)-L-valyl)pyrrolidine-2-carboxamide (7o)

(Due to the distinct presence of rotameric isomers, the ^1^H NMR and ^13^C NMR contained extra peaks. See the attached spectrum in the supporting information) (Purity: 99%; HPLC); *R_f_:* 0.35 (1:10 MeOH:CH_2_Cl_2_); [α]_D_^29^ = +37.8 (*c* 0.0530, MeOH); ^1^H NMR (400 MHz, DMSO) δ 9.65 (s, 1H), 8.79 (d, *J* = 2.6 Hz, 1H), 8.48 − 8.41 (m, 1H), 8.15 (d, *J* = 1.7 Hz, 1H), 8.07 (s, 1H), 8.05 (dd, *J* = 8.4, 2.7 Hz, 1H), 7.93 (dd, *J* = 20.4, 1.8 Hz, 1H), 7.82 (ddd, *J* = 8.0, 4.0, 1.8 Hz, 1H), 7.40 (d, *J* = 8.0 Hz, 1H), 7.18 (d, *J* = 8.5 Hz, 1H), 7.09 (d, *J* = 9.0 Hz, 1H), 4.79 (dd, *J* = 14.1, 8.8 Hz, 1H), 4.50 (ddd, *J* = 19.3, 10.1, 5.6 Hz, 3H), 4.31 (dd, *J* = 7.9, 4.7 Hz, 1H), 3.94 − 3.83 (m, 1H), 3.68 − 3.55 (m, 1H), 3.33 (s, 3H), 3.00 (d, *J* = 1.4 Hz, 3H), 2.80 (s, 3H), 2.40 (s, 3H), 2.20 (s, 3H), 2.17 − 2.08 (m, 1H), 1.98 (m, 3H), 1.87 (dd, *J* = 11.4, 5.7 Hz, 2H), 1.36 (s, 3H), 1.29 (s, 3H), 0.94 (dd, *J* = 6.7, 2.9 Hz, 6H), 0.79 (dd, *J* = 16.8, 6.7 Hz, 6H); ^13^C NMR (101 MHz, DMSO) δ 173.91, 171.63, 171.12, 159.41, 157.41, 150.71, 140.52, 139.70, 135.15, 131.08, 127.25, 126.99, 126.73, 122.93, 103.38, 60.20, 57.31, 56.54, 53.68, 47.73, 47.04, 37.17, 35.45, 30.81, 30.30, 29.44, 28.68, 25.90, 25.27, 24.99, 23.70, 19.95, 19.59, 18.25, 17.74; IR (Neat) 3296, 2957, 2871, 1627, 1606, 1575, 1525, 1492, 1410, 1332, 1290, 1233, 1195, 1140, 1112, 1071, 1034, 938, 831, 786, 731, 700, 621, 559, 516, 457, 408 cm^−1^; HRMS (MM: ESI-APCI+) m/z calc’d for C_42_H_58_N_11_O_6_ [M + H]^+^: 812.4572; found: 812.4584.

#### 4.1.28. (S)-N-(1-(((S)-1-(dimethylamino)-4-methyl-1-oxopentan-2-yl)amino)-2-methyl-1-oxopropan-2-yl)-1-((4-methyl-3-(1-methyl-7-((6-methylpyridin-3-yl)amino)-2-oxo-1,4-dihydropyrimido[4,5-d]pyrimidin-3(2H)-yl)benzoyl)-L-isoleucyl)pyrrolidine-2-carboxamide (7p)

(Due to the distinct presence of rotameric isomers, the ^1^H NMR and ^13^C NMR contained extra peaks. See the attached spectrum in the supporting information) (Purity: 96%; HPLC); *R_f_:* 0.35 (1:10 MeOH:CH_2_Cl_2_); [α]_D_^28^ = −54.7 (*c* 0.0500, MeOH); ^1^H NMR (600 MHz, DMSO) δ 9.64 (s, 1H), 8.79 (d, *J* = 2.6 Hz, 1H), 8.46 (d, *J* = 8.1 Hz, 1H), 8.15 (s, 1H), 8.05 (dd, *J* = 8.4, 2.6 Hz, 1H), 8.00 (s, 1H), 7.92 (dd, *J* = 27.4, 1.9 Hz, 1H), 7.82 (ddd, *J* = 8.2, 4.5, 1.9 Hz, 1H), 7.40 (d, *J* = 8.1 Hz, 1H), 7.25 (d, *J* = 8.6 Hz, 1H), 7.18 (d, *J* = 8.5 Hz, 1H), 4.78 (t, *J* = 13.6 Hz, 1H), 4.72 (td, *J* = 8.6, 5.2 Hz, 1H), 4.60 − 4.48 (m, 2H), 4.28 (dd, *J* = 7.8, 5.3 Hz, 1H), 3.93 (d, *J* = 10.4 Hz, 1H), 3.62 (d, *J* = 8.4 Hz, 1H), 3.33 (s, 3H), 3.01 − 2.94 (m, 3H), 2.78 (s, 3H), 2.40 (s, 3H), 2.20 (s, 3H), 2.06 − 1.95 (m, 3H), 1.86 (d, *J* = 11.8 Hz, 2H), 1.61 − 1.40 (m, 4H), 1.35 (s, 3H), 1.30 (s, 3H), 1.20 − 1.13 (m, 1H), 0.94 (d, *J* = 6.8 Hz, 3H), 0.90 − 0.79 (m, 9H); ^13^C NMR (151 MHz, DMSO) δ 173.61, 171.48, 171.30, 170.67, 170.62, 165.80, 165.67, 159.30, 157.30, 153.60, 152.55, 152.47, 150.60, 141.45, 141.42, 140.40, 139.64, 139.62, 135.05, 133.13, 133.05, 130.98, 127.22, 126.91, 126.61, 122.83, 103.27, 60.26, 60.24, 56.33, 55.72, 47.72, 47.04, 46.93, 41.24, 36.78, 36.01, 35.98, 35.50, 29.27, 28.58, 25.84, 25.82, 25.07, 24.96, 24.93, 24.91, 24.46, 23.60, 23.36, 22.33, 17.66, 17.63, 15.29, 10.91; IR (Neat) 3300, 2959, 2360, 2340, 1629, 1609, 1531, 1496, 1414, 1332, 1290, 1240, 1143, 1115, 845, 737 cm^−1^; HRMS (MM: ESI-APCI+) m/z calc’d for C_44_H_62_N_11_O_6_ [M + H]^+^: 840.4885; found: 840.4885.

#### 4.1.29. (S)-N-(1-(((S)-1-(dimethylamino)-4-methyl-1-oxopentan-2-yl)amino)-2-methyl-1-oxopropan-2-yl)-1-((4-methyl-3-(1-methyl-7-((6-methylpyridin-3-yl)amino)-2-oxo-1,4-dihydropyrimido[4,5-d]pyrimidin-3(2H)-yl)benzoyl)-L-leucyl)pyrrolidine-2-carboxamide (7q)

(Due to the distinct presence of rotameric isomers, the ^1^H NMR and ^13^C NMR contained extra peaks. See the attached spectrum in the supporting information) (Purity: 95%; HPLC); *R_f_:* 0.35 (1:10 MeOH:CH_2_Cl_2_); [α]_D_^26^ = −34.0 (*c* 0.171, MeOH); ^1^H NMR (400 MHz, DMSO) δ 9.63 (s, 1H), 8.78 (d, *J* = 2.6 Hz, 1H), 8.38 (dd, *J* = 8.2, 2.3 Hz, 1H), 8.14 (s, 1H), 8.07 − 8.00 (m, 2H), 7.91 (dd, *J* = 27.5, 1.9 Hz, 1H), 7.84 (dd, *J* = 8.0, 1.9 Hz, 1H), 7.41 (dd, *J* = 8.7, 2.6 Hz, 2H), 7.18 (d, *J* = 8.5 Hz, 1H), 4.84 − 4.63 (m, 3H), 4.51 (dd, *J* = 14.2, 5.3 Hz, 1H), 4.26 (dd, *J* = 7.6, 4.9 Hz, 1H), 3.73 (q, *J* = 6.5 Hz, 1H), 3.55 (dd, *J* = 15.4, 6.4 Hz, 2H), 3.33 (d, *J* = 2.0 Hz, 3H), 2.95 (d, *J* = 2.1 Hz, 3H), 2.77 (s, 3H), 2.40 (s, 3H), 2.20 (s, 3H), 2.06 − 1.94 (m, 2H), 1.87 (td, *J* = 10.4, 5.7 Hz, 2H), 1.73 (t, *J* = 11.4 Hz, 2H), 1.51 (tt, *J* = 17.5, 9.3 Hz, 2H), 1.40 (d, *J* = 8.9 Hz, 1H), 1.36 (s, 3H), 1.30 (s, 3H), 0.96 − 0.89 (m, 6H), 0.85 (dd, *J* = 9.8, 6.5 Hz, 6H); ^13^C NMR (101 MHz, DMSO) δ 173.79, 171.50, 171.47, 171.26, 159.40, 157.40, 150.74, 140.49, 139.82, 135.14, 131.16, 126.79, 122.98, 103.38, 60.49, 56.50, 49.92, 47.30, 47.04, 41.39, 36.81, 35.63, 29.12, 28.68, 26.01, 25.18, 24.99, 24.85, 24.49, 23.76, 23.66, 23.49, 22.43, 21.68, 21.65, 17.74.IR (Neat) 3298, 2924, 1628, 1605, 1576, 1528, 1493, 1448, 1411, 1332, 1288, 1264, 1235, 1194, 1142, 1112, 1075, 1031, 943, 830, 787, 732, 700, 623, 548, 514, 480, 458 cm^−1^; HRMS (MM: ESI-APCI+) m/z calc’d for C_44_H_62_N_11_O_6_ [M + H]^+^: 840.4806; found: 840.4885.

#### 4.1.30. (S)-N-(1-(((S)-1-(dimethylamino)-4-methyl-1-oxopentan-2-yl)amino)-2-methyl-1-oxopropan-2-yl)-1-((4-methyl-3-(1-methyl-7-((6-methylpyridin-3-yl)amino)-2-oxo-1,4-dihydropyrimido[4,5-d]pyrimidin-3(2H)-yl)benzoyl)glycyl)pyrrolidine-2-carboxamide (7r)

(Due to the distinct presence of rotameric isomers, the ^1^H NMR and ^13^C NMR contained extra peaks. See the attached spectrum in the supporting information) (Purity: 97%; HPLC); *R_f_:* 0.35 (1:10 MeOH:CH_2_Cl_2_); [α]_D_^30^ = −27.0 (*c* 0.100, MeOH); ^1^H NMR (600 MHz, DMSO) δ 9.64 (d, *J* = 2.4 Hz, 1H), 8.79 (d, *J* = 2.7 Hz, 1H), 8.17 − 8.09 (m, 2H), 8.05 (d, *J* = 8.6, 2.5 Hz, 1H), 7.96 (d, *J* = 22.1, 1.8 Hz, 1H), 7.86 (dd, *J* = 13.2, 5.9 Hz, 1H), 7.42 (d, *J* = 8.0 Hz, 1H), 7.33 (dd, *J* = 18.9, 8.8 Hz, 1H), 7.17 (d, *J* = 8.4 Hz, 1H), 4.80 − 4.71 (m, 1H), 4.68 (m, *J* = 8.9, 5.2 Hz, 1H), 4.52 (d, *J* = 14.0, 4.2 Hz, 1H), 4.29 (m, *J* = 5.8, 5.2 Hz, 1H), 3.97 (m, *J* = 21.2, 17.0, 4.6 Hz, 1H), 3.58 (m, *J* = 6.2 Hz, 1H), 3.32 (s, 3H), 3.00 − 2.91 (m, 3H), 2.79 (d, 3H), 2.40 (s, 3H), 2.21 (s, 3H), 2.11 − 1.95 (m, 2H), 1.93 − 1.74 (m, 2H), 1.50 (qd, *J* = 13.7, 6.5 Hz, 1H), 1.39 (m, 2H), 1.34 (s, *3*H), 1.29 (s, 3H), 0.79 (m, 6H).^13^C NMR (151 MHz, DMSO) δ 173.59, 173.45, 171.68, 171.45, 171.44, 171.41, 171.20, 167.88, 167.51, 167.48, 165.76, 165.66, 159.30, 157.34, 157.31, 153.58, 152.50, 152.48, 152.46, 150.59, 141.58, 141.56, 141.53, 140.41, 140.39, 139.74, 139.72, 139.60, 139.58, 135.05, 133.30, 133.15, 133.12, 131.10, 131.07, 126.95, 126.89, 126.82, 126.70, 126.61, 126.59, 122.82, 103.29, 60.23, 59.40, 56.44, 56.40, 47.15, 46.99, 46.98, 46.94, 46.50, 42.40, 41.88, 41.32, 36.81, 36.65, 36.62, 35.49, 35.40, 35.37, 32.05, 29.02, 28.99, 28.59, 28.57, 28.55, 26.33, 26.27, 25.65, 24.88, 24.85, 24.84, 24.59, 24.42, 24.08, 24.05, 23.59, 23.49, 23.42, 23.36, 22.33, 22.20, 22.18, 22.07, 22.05, 17.64; IR (Neat) 3402, 2926, 1610, 1533, 1499, 1463, 1417, 1276, 1120, 846, 750 cm^−1^; HRMS (MM: ESI-APCI+) m/z calc’d for C_40_H_54_N_11_O_6_ [M + H]^+^: 784.4259; found: 784.4257.

#### 4.1.31. (S)-1-((S)-2-cyclohexyl-2-(4-methyl-3-(1-methyl-7-((6-methylpyridin-3-yl)amino)-2-oxo-1,4-dihydropyrimido[4,5-d]pyrimidin-3(2H)-yl)benzamido)acetyl)-N-(1-(((S)-1-(dimethylamino)-4-methyl-1-oxopentan-2-yl)amino)-2-methyl-1-oxopropan-2-yl)pyrrolidine-2-carboxamide (7s)

(Due to the distinct presence of rotameric isomers, the ^1^H NMR and ^13^C NMR contained extra peaks. See the attached spectrum in the supporting information) (Purity: 97%; HPLC); *R_f_:* 0.35 (1:10 MeOH:CH_2_Cl_2_); [α]_D_^29^ = −51.4 (*c* 0.0390, MeOH); ^1^H NMR (400 MHz, DMSO) δ 9.66 (s, 1H), 8.81 (d, *J* = 2.6 Hz, 1H), 8.38 (d, *J* = 8.3 Hz, 1H), 8.16 (s, 1H), 8.07 (dd, *J* = 8.5, 2.7 Hz, 1H), 7.94 (dd, *J* = 17.8, 1.8 Hz, 2H), 7.84 (d, *J* = 8.0 Hz, 1H), 7.45 − 7.37 (m, 2H), 7.19 (d, *J* = 8.5 Hz, 1H), 4.80 (dd, *J* = 14.0, 7.5 Hz, 1H), 4.77 − 4.65 (m, 1H), 4.63 − 4.48 (m, 2H), 4.29 (t, *J* = 6.2 Hz, 1H), 3.90 (s, 1H), 3.64 (s, 1H), 3.35 (s, 3H), 3.00 (s, 3H), 2.80 (s, 3H), 2.42 (s, 3H), 2.22 (s, 3H), 2.14 − 1.77 (m, 8H), 1.65 (d, *J* = 19.5 Hz, 2H), 1.57 (m, 1H), 1.50 − 1.32 (m, 8H), 1.21 (d, *J* = 32.8 Hz, 3H), 1.05 (d, *J* = 15.2 Hz, 2H), 0.92 − 0.86 (m, 6H); ^13^C NMR (101 MHz, DMSO) δ 177.65, 173.79, 171.55, 171.21, 170.71, 169.57, 159.41, 153.76, 150.71, 141.57, 140.51, 139.73, 138.69, 135.16, 131.10, 127.38, 126.97, 126.72, 122.94, 117.93, 103.38, 84.38, 56.45, 47.79, 47.25, 41.35, 36.87, 35.62, 29.13, 28.68, 25.51, 25.28, 25.06, 24.64, 23.70, 22.46, 17.76; IR (Neat) 3300, 2927, 2854, 2360, 2340, 1629, 1609, 1530, 1496, 1414, 1334, 1289, 1236, 1192, 1143, 1117, 845, 788, 737 cm^−1^; HRMS (MM: ESI-APCI+) m/z calc’d for C_46_H_64_N_11_O_6_ [M + H]^+^: 866.5041; found: 866.5021.

#### 4.1.32. (S)-N-(1-(((S)-1-(dimethylamino)-4-methyl-1-oxopentan-2-yl)amino)-2-methyl-1-oxopropan-2-yl)-1-((S)-2-(4-methyl-3-(1-methyl-7-((6-methylpyridin-3-yl)amino)-2-oxo-1,4-dihydropyrimido[4,5-d]pyrimidin-3(2H)-yl)benzamido)pentanoyl)pyrrolidine-2-carboxamide (7t)

(Due to the distinct presence of rotameric isomers, the ^1^H NMR and ^13^C NMR contained extra peaks. See the attached spectrum in the supporting information) (Purity: 99%; HPLC); *R_f_:* 0.35 (1:10 MeOH:CH_2_Cl_2_); [α]_D_^30^ = +24.8 (*c* 0.0556, MeOH); ^1^H NMR (400 MHz, DMSO) δ 9.65 (s, 1H), 8.80 (d, *J* = 2.6 Hz, 1H), 8.41 − 8.34 (m, 1H), 8.15 (s, 1H), 8.08 − 7.99 (m, 2H), 7.98 − 7.89 (m, 1H), 7.87 − 7.82 (m, 1H), 7.41 (t, *J* = 7.2 Hz, 2H), 7.18 (d, *J* = 8.5 Hz, 1H), 4.83 − 4.66 (m, 3H), 4.53 (dd, *J* = 14.0, 5.1 Hz, 1H), 4.27 (t, *J* = 6.2 Hz, 1H), 3.75 (d, *J* = 7.8 Hz, 1H), 3.58 (s, 1H), 3.33 (s, 3H), 2.97 (d, *J* = 2.4 Hz, 3H), 2.79 (s, 3H), 2.41 (s, 3H), 2.21 (s, 3H), 1.99 (dd, *J* = 14.4, 7.9 Hz, 2H), 1.88 (s, 2H), 1.76 − 1.69 (m, 2H), 1.53 (dt, *J* = 15.1, 7.7 Hz, 2H), 1.42 (d, *J* = 18.8 Hz, 3H), 1.37 (s, 3H), 1.31 (s, 3H), 0.92 (d, *J* = 7.2 Hz, 3H), 0.85 (dd, *J* = 9.6, 6.4 Hz, 6H); ^13^C NMR (101 MHz, DMSO) δ 173.76, 171.48, 171.02, 165.80, 165.68, 159.41, 157.42, 152.61, 150.72, 141.62, 140.52, 139.79, 135.16, 131.15, 126.73, 122.94, 103.38, 60.50, 56.50, 51.45, 47.41, 47.26, 47.06, 41.39, 36.82, 35.59, 33.53, 29.15, 28.68, 26.04, 25.18, 25.04, 24.50, 23.71, 23.49, 22.45, 19.21, 17.76, 14.21; IR (Neat) 3288, 2955, 2918, 2849, 1727, 1633, 1607, 1577, 1532, 1497, 1462, 1411, 1378, 1294, 1118, 1099, 1019, 800, 739 cm^−1^; HRMS (MM: ESI-APCI+) m/z calc’d for C_43_H_59_N_11_O_6_Na [M + Na]^+^: 848.4547; found: 848.4550.

#### 4.1.33. (S)-N-(1-((2-(dimethylamino)-2-oxoethyl)amino)-2-methyl-1-oxopropan-2-yl)-1-((4-methyl-3-(1-methyl-7-((6-methylpyridin-3-yl)amino)-2-oxo-1,4-dihydropyrimido[4,5-d]pyrimidin-3(2H)-yl)benzoyl)-L-valyl)pyrrolidine-2-carboxamide (7u)

(Due to the distinct presence of rotameric isomers, the ^1^H NMR and ^13^C NMR contained extra peaks. See the attached spectrum in the supporting information) (Purity: 96%; HPLC); *R_f_:* 0.35 (1:10 MeOH:CH_2_Cl_2_); [α]_D_^27^ = −59.5 (*c* 0.0240, MeOH); ^1^H NMR (600 MHz, DMSO) δ 9.65 (s, 1H), 8.79 (d, *J* = 2.6 Hz, 1H), 8.49 (dd, *J* = 8.2, 4.1 Hz, 1H), 8.26 (s, 1H), 8.15 (d, *J* = 3.7 Hz, 1H), 8.05 (dd, *J* = 8.4, 2.7 Hz, 1H), 7.93 (dd, *J* = 29.6, 1.9 Hz, 1H), 7.82 (ddd, *J* = 7.8, 5.8, 1.9 Hz, 1H), 7.47 (t, *J* = 5.3 Hz, 1H), 7.40 (dd, *J* = 8.2, 1.9 Hz, 1H), 7.18 (d, *J* = 8.5 Hz, 1H), 4.78 (dd, *J* = 16.0, 14.1 Hz, 1H), 4.53 − 4.45 (m, 2H), 4.30 (t, *J* = 6.8 Hz, 1H), 4.00 (dd, *J* = 16.6, 5.8 Hz, 1H), 3.96 (q, *J* = 7.7 Hz, 1H), 3.61 (ddd, *J* = 16.8, 12.0, 6.1 Hz, 2H), 3.33 (s, 3H), 2.93 (s, 3H), 2.80 (s, 3H), 2.40 (s, 3H), 2.20 (d, *J* = 1.5 Hz, 3H), 2.14 (dd, *J* = 13.5, 6.4 Hz, 1H), 2.08 − 1.97 (m, 2H), 1.86 (qd, *J* = 12.1, 11.4, 5.3 Hz, 2H), 1.34 (d, *J* = 12.4 Hz, 6H), 0.97 − 0.92 (m, 6H); ^13^C NMR (151 MHz, DMSO) δ 174.17, 171.68, 170.83, 170.79, 168.40, 165.92, 165.77, 159.30, 157.30, 153.60, 152.55, 152.48, 150.60, 141.45, 141.42, 140.40, 139.63, 135.04, 133.13, 133.04, 131.00, 127.21, 127.15, 126.91, 126.61, 122.83, 103.28, 63.15, 60.24, 60.21, 57.21, 56.39, 47.80, 46.93, 35.98, 35.38, 30.42, 30.37, 29.12, 28.58, 25.96, 25.94, 25.16, 25.13, 25.12, 23.60, 19.40, 19.38, 19.17, 17.66, 17.63; IR (Neat) 3289, 2927, 1605, 1575, 1528, 1493, 1411, 1332, 1289, 1234, 1195, 1142, 1113, 1033, 947, 842, 786, 731 cm^−1^; HRMS (MM: ESI-APCI+) m/z calc’d for C_39_H_52_N_11_O_6_ [M + H]^+^: 770.4102; found: 770.4101.

#### 4.1.34. (R)-N-((S)-3-methyl-1-oxo-1-(((R)-1-phenylethyl)amino)butan-2-yl)-1-((4-methyl-3-(1-methyl-7-((6-methylpyridin-3-yl)amino)-2-oxo-1,4-dihydropyrimido[4,5-d]pyrimidin-3(2H)-yl)benzoyl)-L-valyl)pyrrolidine-2-carboxamide (7v)

(Due to the distinct presence of rotameric isomers, the ^1^H NMR and ^13^C NMR contained extra peaks. See the attached spectrum in the supporting information) (Purity: 95%; HPLC); *R_f_:* 0.35 (1:10 MeOH:CH_2_Cl_2_); [α]_D_^29^ = +4.25 (*c* 0.230, MeOH); ^1^H NMR (400 MHz, DMSO) δ 10.60 (s, 1H), 8.93 − 8.89 (m, 1H), 8.84 (s, 1H), 8.53 (dd, *J* = 7.3, 3.1 Hz, 1H), 8.20 (d, *J* = 8.0 Hz, 1H), 8.07 (dd, *J* = 8.4, 2.7 Hz, 1H), 7.94 − 7.87 (m, 1H), 7.83 (s, 1H), 7.45 (d, *J* = 8.0 Hz, 1H), 7.40 − 7.32 (m, 2H), 7.31 − 7.24 (m, 5H), 4.92 − 4.79 (m, 1H), 4.45 − 4.34 (m, 2H), 4.09 − 3.99 (m, 1H), 3.96 (s, 1H), 3.66 − 3.58 (m, 1H), 3.53 (d, *J* = 4.0 Hz, 3H), 3.46 − 3.37 (m, 1H), 3.67 − 3.56 (m, 1H), 2.45 (s, 3H), 2.13 (s, 3H), 2.08 − 1.98 (m, 2H), 1.97 − 1.75 (m, 5H), 1.27 (d, *J* = 7.0 Hz, 3H), 0.98 (d, *J* = 6.6 Hz, 6H), 0.76 (t, *J* = 6.6 Hz, 3H), 0.62 (dd, *J* = 6.9, 4.3 Hz, 3H); ^13^C NMR (101 MHz, DMSO) δ 172.56, 171.70, 170.91, 170.32, 170.23, 166.19, 165.84, 161.86, 160.08, 159.76, 159.68, 158.24, 152.92, 151.03, 150.97, 144.74, 144.63, 141.79, 140.38, 134.97, 133.63, 132.87, 132.75, 130.85, 130.71, 129.02, 128.58, 128.50, 128.40, 127.02, 126.94, 126.62, 126.46, 123.20, 101.11, 60.34, 59.75, 58.39, 57.91, 57.12, 48.21, 48.05, 47.67, 47.12, 32.92, 31.30, 30.37, 30.14, 29.87, 29.57, 29.42, 24.48, 23.86, 22.77, 22.73, 22.42, 19.79, 19.66, 19.58, 19.47, 19.41, 18.84, 18.49, 17.45; IR (Neat) 3294, 2956, 2922, 2853, 1725, 1664, 1595, 1571, 1529, 1494, 1462, 1398, 1377, 1333, 1300, 1267, 1236, 1207, 1174, 1115, 1070, 1030, 958, 916, 885, 829, 804, 756, 732, 699, 670, 629, 596, 560, 501, 460, 447, 426 cm^−1^; HRMS (MM: ESI-APCI+) m/z calc’d for C_44_H_55_N_10_O_5_ [M + H]^+^: 803.4357; found: 803.4371.

#### 4.1.35. (R)-N-((S)-3-methyl-1-oxo-1-(((R)-1-phenylethyl)amino)butan-2-yl)-1-((4-methyl-3-(1-methyl-7-((6-methylpyridin-3-yl)amino)-2-oxo-1,4-dihydropyrimido[4,5-d]pyrimidin-3(2H)-yl)benzoyl)-L-phenylalanyl)pyrrolidine-2-carboxamide (7w)

(Due to the distinct presence of rotameric isomers, the ^1^H NMR and ^13^C NMR contained extra peaks. See the attached spectrum) (Purity: 99%; HPLC); *R_f_:* 0.35 (1:10 MeOH:CH_2_Cl_2_); [α]_D_^27^ = +104 (*c* 0.0560, MeOH); (Due to the distinct presence of rotameric isomers, the ^1^H NMR and ^13^C NMR contained extra peaks. See the attached spectrum in the supporting information) ^1^H NMR (400 MHz, DMSO) δ 9.66 (d, *J* = 3.8 Hz, 1H), 8.83 (t, *J* = 5.6 Hz, 1H), 8.80 − 8.75 (m, 1H), 8.21 − 8.10 (m, 2H), 8.05 (dt, *J* = 8.4, 2.2 Hz, 1H), 7.89 (dd, *J* = 3.7, 1.8 Hz, 1H), 7.80 − 7.73 (m, 1H), 7.49 − 7.32 (m, 2H), 7.32 − 7.26 (m, 5H), 7.25 − 7.09 (m, 5H), 6.98 − 6.89 (m, 1H), 4.91 − 4.83 (m, 1H), 4.80 (dd, *J* = 8.9, 6.9 Hz, 1H), 4.67 (d, *J* = 14.0 Hz, 1H), 4.55 − 4.50 (m, 1H), 4.34 − 4.21 (m, 1H), 3.69 (s, 1H), 3.17 (d, *J* = 5.2 Hz, 1H), 3.06 (d, *J* = 7.6 Hz, 1H), 3.02 − 2.84 (m, 1H), 2.41 (s, 3H), 2.19 (d, *J* = 14.1 Hz, 3H), 2.08 (s, 3H), 2.06 − 1.92 (m, 1H), 1.84 − 1.65 (m, 3H), 1.29 − 1.21 (m, 3H), 0.92 − 0.64 (m, 6H);^13^C NMR (101 MHz, DMSO) δ 171.65, 171.62, 170.78, 170.73, 170.30, 170.27, 170.14, 165.98, 165.82, 159.45, 157.42, 153.73, 152.53, 152.51, 150.73, 144.80, 144.76, 141.53, 140.54, 139.93, 139.79, 138.78, 137.81, 137.77, 135.15, 132.83, 132.78, 131.01, 129.77, 129.59, 128.66, 128.58, 128.34, 127.13, 126.99, 126.81, 126.78, 126.74, 126.42, 122.93, 103.29, 60.34, 58.51, 58.42, 53.96, 53.85, 53.62, 49.07, 48.46, 48.17, 47.16, 47.04, 31.15, 30.26, 29.78, 28.68, 23.70, 22.75, 22.70, 19.73, 19.60, 19.46, 18.54, 18.47, 18.14, 17.76); IR (Neat) 3291, 2966, 1640, 1607, 1574, 1531, 1496, 1451, 1414, 1332, 1292, 1235, 1193, 1145, 1118, 1029, 843, 739, 701 cm^−1^; HRMS (MM: ESI-APCI+) m/z calc’d for C_48_H_55_N_10_O_5_ [M + H]^+^: 851.4357; found: 851.4377.

#### 4.1.36. (R)-3-benzyl-N-(4-methyl-3-(1-methyl-7-((6-methylpyridin-3-yl)amino)-2-oxo-1,4-dihydropyrimido[4,5-d]pyrimidin-3(2H)-yl)phenyl)-2-oxo-5-phenyl-2,3-dihydro-1H-benzo[e][1,4]diazepine-8-carboxamide (11f)

(Purity: 96%; HPLC); *R_f_:* 0.35 (1:10 MeOH:CH_2_Cl_2_); [α]_D_^21^ = −62.7 (*c* 0.0390, MeOH); ^1^H NMR (400 MHz, DMSO) δ 10.85 (s, 1H), 10.57 (s, 1H), 9.65 (s, 1H), 8.80 (d, *J* = 2.6 Hz, 1H), 8.16 (d, *J* = 1.7 Hz, 1H), 8.05 (dd, *J* = 8.4, 2.7 Hz, 1H), 7.85 (dd, *J* = 4.2, 2.2 Hz, 1H), 7.79 (d, *J* = 1.7 Hz, 1H), 7.72 (dd, *J* = 8.2, 1.7 Hz, 1H), 7.62 (dd, *J* = 8.3, 2.2 Hz, 1H), 7.53 − 7.48 (m, 1H), 7.46 − 7.42 (m, 4H), 7.37 − 7.33 (m, 3H), 7.28 (td, *J* = 8.1, 6.2 Hz, 3H), 7.18 (dd, *J* = 8.0, 5.3 Hz, 2H), 4.71 (d, *J* = 14.0 Hz, 1H), 4.52 (d, *J* = 14.1 Hz, 1H), 3.73 (dd, *J* = 8.1, 5.4 Hz, 1H), 3.47 − 3.35 (m, 2H), 2.40 (s, 3H), 2.13 (s, 3H); ^13^C NMR (101 MHz, DMSO) δ 170.54, 167.78, 159.42, 157.48, 152.55, 150.67, 141.62, 140.52, 139.76, 139.66, 139.01, 138.18, 135.18, 131.30, 131.14, 130.91, 130.18, 129.76, 129.12, 128.80, 128.54, 126.72, 126.49, 122.92, 121.27, 47.12, 40.63, 40.43, 40.22, 40.01, 39.80, 39.59, 39.38, 28.70, 23.70, 17.27; IR (Neat) 3298, 2925, 1671, 1601, 1535, 1413, 1321, 1119, 1032, 747, 698 cm^−1^; HRMS (MM: ESI-APCI+) m/z calc’d for C_43_H_37_N_9_O_3_Na [M + Na]^+^: 750.2917; found: 750.2933.

#### 4.1.37. (S)-3-(4-fluorobenzyl)-N-(4-methyl-3-(1-methyl-7-((6-methylpyridin-3-yl)amino)-2-oxo-1,4-dihydropyrimido[4,5-d]pyrimidin-3(2H)-yl)phenyl)-2-oxo-5-phenyl-2,3-dihydro-1H-benzo[e][1,4]diazepine-8-carboxamide (11g)

(Purity: 95%; HPLC); *R_f_:* 0.35 (1:10 MeOH:CH_2_Cl_2_); [α]_D_^30^ = +0.706 (*c* 0.0940, MeOH);); ^1^H NMR (600 MHz, DMSO) δ 10.83 (s, 1H), 10.51 (s, 1H), 9.64 (s, 1H), 8.80 (s, 1H), 8.16 (s, 1H), 8.05 (d, *J* = 7.2 Hz, 1H), 7.84 (d, *J* = 5.8 Hz, 1H), 7.76 (s, 1H), 7.70 (d, *J* = 8.3 Hz, 1H), 7.60 (d, *J* = 8.3 Hz, 1H), 7.51 (m, 2H), 7.47 − 7.35 (m, 8H), 7.30 (d, *J* = 8.4 Hz, 1H), 7.18 (d, *J* = 8.4 Hz, 1H), 7.10 (t, *J* = 8.7 Hz, 3H), 4.71 (d, *J* = 14.0 Hz, 1H), 4.53 (d, *J* = 14.0 Hz, 1H), 3.73 (t, *J* = 6.8 Hz, 1H), 3.41 (dd, *J* = 13.2, 5.3 Hz, 2H), 2.40 (s, 3H), 2.13 (s, 3H); ^13^C NMR (151 MHz, DMSO) δ 170.10, 167.41, 164.40, 161.62, 160.02, 158.94, 157.01, 153.26, 152.09, 150.22, 141.17, 140.03, 139.28, 138.53, 137.73, 135.25 (d, *J* = 2.9 Hz), 134.71, 131.51 (d, *J* = 7.6 Hz), 130.87, 130.77, 130.75, 129.57 (d, *J* = 271.9 Hz), 129.30, 129.25, 128.35, 126.23, 122.47, 121.37, 120.76, 119.62, 119.06, 119.03, 114.72 (d, *J* = 20.9 Hz), 102.95, 65.00, 46.64, 38.25, 36.26, 28.24, 23.24, 16.81; ^19^F NMR (377 MHz, DMSO) δ −117.19; IR (Neat) 3324, 2925, 2854, 1674, 1607, 1511, 1463, 1408, 1261, 1100, 748, 669 cm^−1^; HRMS (MM: ESI-APCI+) m/z calc’d for C_43_H_37_FN_9_O_3_ [M + H]^+^: 746.3003; found: 746.3005.

#### 4.1.38. (S)-3-isobutyl-1-methyl-N-(4-methyl-3-(1-methyl-7-((6-methylpyridin-3-yl)amino)-2-oxo-1,4-dihydropyrimido[4,5-d]pyrimidin-3(2H)-yl)phenyl)-2-oxo-5-phenyl-2,3-dihydro-1H-benzo[e][1,4]diazepine-8-carboxamide (11h)

(Purity: 99%; HPLC); *R_f_:* 0.35 (1:10 MeOH:CH_2_Cl_2_); [α]_D_^26^ = +55.8 (*c* 0.110, MeOH); ^1^H NMR (600 MHz, DMSO) δ 10.53 (s, 1H), 9.76 (s, 1H), 8.87 (t, *J* = 2.5 Hz, 1H), 8.18 (s, 1H), 8.12 (d, *J* = 8.5 Hz, 1H), 8.06 (s, 1H), 7.85 − 7.81 (m, 1H), 7.80 (d, *J* = 8.2 Hz, 1H), 7.65 − 7.61 (m, 1H), 7.55 (d, *J* = 7.6 Hz, 2H), 7.52 (d, *J* = 7.3 Hz, 1H), 7.46 (dd, *J* = 11.7, 7.8 Hz, 3H), 7.33 (d, *J* = 8.4 Hz, 1H), 7.27 (d, *J* = 8.5 Hz, 1H), 4.72 (d, *J* = 14.0 Hz, 1H), 4.55 (d, *J* = 14.0 Hz, 1H), 3.62 (ddq, *J* = 9.6, 6.8, 3.4, 2.8 Hz, 1H), 3.57 (dt, *J* = 8.9, 5.4 Hz, 1H), 3.35 (s, 3H), 3.14 (qd, *J* = 7.4, 4.1 Hz, 2H), 2.44 (s, 3H), 2.15 (s, 4H), 1.92 − 1.78 (m, 2H), 0.94 (d, *J* = 6.4 Hz, 3H), 0.76 (d, *J* = 6.3 Hz, 3H); ^13^C NMR (151 MHz, DMSO) δ 170.28, 167.36, 164.71, 159.15, 157.42, 153.61, 152.45, 149.85, 143.69, 141.53, 138.36, 138.03, 137.98, 135.53, 131.31, 131.17, 130.91, 130.80, 130.16, 129.57, 128.76, 123.56, 123.31, 121.38, 120.16, 119.61, 103.59, 61.52, 53.96, 46.99, 42.21, 35.10, 28.64, 24.53, 23.74, 22.93, 22.14, 18.45, 17.17, 17.09, 12.86; IR (Neat) 2954, 2360, 2340, 1659, 1599, 1533, 1492, 1411, 1323, 1266, 1188, 1143, 1030, 837, 784, 733, 698 cm^−1^; HRMS (MM: ESI-APCI+) m/z calc’d for C_41_H_42_N_9_O_3_ [M + H]^+^: 708.3411; found: 708.3407.

#### 4.1.39. (S)-1-allyl-3-isobutyl-N-(4-methyl-3-(1-methyl-7-((6-methylpyridin-3-yl)amino)-2-oxo-1,4-dihydropyrimido[4,5-d]pyrimidin-3(2H)-yl)phenyl)-2-oxo-5-phenyl-2,3-dihydro-1H-benzo[e][1,4]diazepine-8-carboxamide (11i)

(Purity: 96%; HPLC); *R_f_:* 0.35 (1:10 MeOH:CH_2_Cl_2_); [α]_D_^28^ = +44.6 (*c* 0.180, MeOH); ^1^H NMR (600 MHz, DMSO) δ 10.51 (s, 1H), 9.65 (s, 1H), 8.89 − 8.64 (m, 1H), 8.14 (d, *J* = 31.3 Hz, 2H), 8.08 − 8.01 (m, 1H), 7.81 (m, 2H), 7.62 (d, *J* = 8.3 Hz, 1H), 7.57 − 7.39 (m, 6H), 7.32 (d, *J* = 8.3 Hz, 1H), 7.18 (d, *J* = 8.3 Hz, 1H), 5.76 (ddt, *J* = 16.1, 10.3, 5.0 Hz, 1H), 5.04 (d, *J* = 10.4 Hz, 1H), 4.98 (d, *J* = 17.3 Hz, 1H), 4.73 (dd, *J* = 18.1, 7.8 Hz, 2H), 4.60 − 4.50 (m, 2H), 3.64 (dd, *J* = 8.8, 4.5 Hz, 1H), 3.34 (s, 3H), 2.40 (s, 3H), 2.14 (s, 3H), 2.11 (m, 1H), 1.88 (dd, *J* = 12.9, 7.1 Hz, 2H), 0.94 (d, *J* = 5.7 Hz, 3H), 0.77 (d, *J* = 5.6 Hz, 3H); ^13^C NMR (151 MHz, DMSO) δ 169.22, 167.53, 164.63, 159.31, 157.38, 153.62, 152.48, 150.58, 142.44, 141.56, 140.39, 138.37, 138.02, 138.00, 135.06, 133.77, 131.85, 131.33, 131.16, 130.93, 130.15, 129.45, 128.84, 126.59, 123.70, 122.83, 122.08, 120.15, 120.13, 119.63, 119.60, 116.64, 103.31, 61.69, 49.16, 47.00, 40.42, 28.61, 24.63, 23.66, 23.60, 22.30, 17.17; IR (Neat) 3294, 2953, 2360, 2340, 1663, 1598, 1532, 1491, 1410, 1293, 1231, 1187, 1142, 1119, 1030, 989, 914, 841, 784, 739, 697 cm^−1^; HRMS (MM: ESI-APCI+) m/z calc’d for C_43_H_44_N_9_O_3_ [M + H]^+^: 734.3567; found: 734.3565.

#### 4.1.40. (S)-1,3-diisobutyl-N-(4-methyl-3-(1-methyl-7-((6-methylpyridin-3-yl)amino)-2-oxo-1,4-dihydropyrimido[4,5-d]pyrimidin-3(2H)-yl)phenyl)-2-oxo-5-phenyl-2,3-dihydro-1H-benzo[e][1,4]diazepine-8-carboxamide (11j)

(Purity: 97%; HPLC); *R_f_:* 0.35 (1:10 MeOH:CH_2_Cl_2_); [α]_D_^31^ = +21.4 (*c* 0.0889, MeOH); ^1^H NMR (600 MHz, DMSO) δ 10.48 (s, 1H), 9.65 (s, 1H), 8.80 (d, *J* = 2.6 Hz, 1H), 8.17 (d, *J* = 3.0 Hz, 2H), 8.05 (dd, *J* = 8.6, 2.7 Hz, 1H), 7.86 − 7.77 (m, 2H), 7.64 (dt, *J* = 5.9, 2.9 Hz, 1H), 7.57 − 7.51 (m, 3H), 7.48 (t, *J* = 7.4 Hz, 2H), 7.44 (d, *J* = 8.1 Hz, 1H), 7.33 (d, *J* = 8.4 Hz, 1H), 7.18 (d, *J* = 8.5 Hz, 1H), 4.71 (d, *J* = 13.9 Hz, 1H), 4.54 (d, *J* = 14.0 Hz, 1H), 4.21 (dd, *J* = 13.8, 8.9 Hz, 1H), 3.66 (dd, *J* = 13.8, 5.9 Hz, 1H), 3.57 (dd, *J* = 8.9, 4.4 Hz, 1H), 3.34 (s, 3H), 2.41 (s, 3H), 2.15 (s, 3H), 2.14 − 2.09 (m, 1H), 1.83 (tt, *J* = 13.0, 7.4 Hz, 2H), 1.66 (p, *J* = 6.7 Hz, 1H), 0.93 (d, *J* = 6.1 Hz, 3H), 0.75 (d, *J* = 6.1 Hz, 3H), 0.71 (d, *J* = 6.6 Hz, 3H), 0.53 (d, *J* = 6.6 Hz, 3H); ^13^C NMR (151 MHz, DMSO) δ 170.12, 167.31, 164.54, 159.31, 157.39, 153.63, 152.48, 150.58, 142.48, 141.55, 140.39, 138.16, 137.97, 135.06, 132.44, 131.34, 131.15, 130.93, 130.09, 129.35, 128.88, 126.59, 123.91, 122.83, 122.41, 120.25, 119.77, 119.73, 103.31, 61.72, 52.75, 47.01, 31.32, 28.61, 27.11, 24.59, 23.71, 23.60, 22.43, 22.24, 20.23, 19.50, 17.18, 14.33; IR (Neat) 3239, 2957, 2924, 2854, 1688, 1658, 1600, 1577, 1534, 1508, 1494, 1465, 1412, 1321, 1296, 1231, 1188, 1145, 1120, 1033, 842, 785, 738, 698, 659, 558, 464, 411 cm^−1^; HRMS (MM: ESI-APCI+) m/z calc’d for C_44_H_48_N_9_O_3_ [M + H]^+^: 750.3880; found: 750.3883.

#### 4.1.41. (S)-3-benzyl-5-methyl-N-(4-methyl-3-(1-methyl-7-((6-methylpyridin-3-yl)amino)-2-oxo-1,4-dihydropyrimido[4,5-d]pyrimidin-3(2H)-yl)phenyl)-2-oxo-2,3-dihydro-1H-benzo[e][1,4]diazepine-8-carboxamide (11k)

(Purity: 99%; HPLC); *R_f_:* 0.35 (1:10 MeOH:CH_2_Cl_2_); [α]_D_^33^ = +60.3 (*c* 0.110, MeOH); ^1^H NMR (600 MHz, DMSO) δ 10.66 (s, 1H), 10.44 (s, 1H), 9.66 (s, 1H), 8.81 (d, *J* = 2.7 Hz, 1H), 8.15 (d, *J* = 3.0 Hz, 1H), 8.06 (dd, *J* = 8.5, 2.6 Hz, 1H), 7.88 − 7.79 (m, 2H), 7.75 − 7.71 (m, 1H), 7.64 (s, 1H), 7.59 (d, *J* = 8.2 Hz, 1H), 7.29 (d, *J* = 8.4 Hz, 1H), 7.27 − 7.16 (m, 5H), 7.14 (t, *J* = 7.3 Hz, 1H), 4.70 (d, *J* = 13.7 Hz, 1H), 4.55 − 4.45 (m, 1H), 3.55 (d, *J* = 14.0 Hz, 1H), 3.44 − 3.40 (m, 1H), 3.34 (s, 3H), 3.16 (dt, *J* = 15.0, 7.5 Hz, 1H), 2.41 (s, 6H), 2.13 (s, 3H); ^13^C NMR (151 MHz, DMSO) δ 169.93, 167.27, 164.76, 159.28, 157.37, 153.59, 152.45, 150.45, 141.52, 140.14, 139.58, 138.08, 137.61, 137.59, 135.14, 131.19, 131.12, 130.88, 129.90, 129.87, 129.14, 128.40, 126.75, 126.30, 122.93, 122.23, 120.87, 119.95, 119.42, 119.38, 103.34, 64.85, 46.99, 37.34, 28.61, 25.86, 23.49, 17.16; IR (Neat) 3278, 2923, 2853, 2360, 2340, 1669, 1600, 1534, 1496, 1411, 1296, 1236, 1189, 1145, 1113, 1072, 1029, 834, 786, 737, 700 cm^−1^; HRMS (MM: ESI-APCI+) m/z calc’d for C_38_H_36_N_9_O_3_ [M + H]^+^: 666.2941; found: 666.2943.

#### 4.1.42. (R)-3-benzyl-5-methyl-N-(4-methyl-3-(1-methyl-7-((6-methylpyridin-3-yl)amino)-2-oxo-1,4-dihydropyrimido[4,5-d]pyrimidin-3(2H)-yl)phenyl)-2-oxo-2,3-dihydro-1H-benzo[e][1,4]diazepine-8-carboxamide (11l)

(Purity: 98%; HPLC); *R_f_:* 0.35 (1:10 MeOH:CH_2_Cl_2_); [α]_D_^25^ = −73.5 (*c* 0.0890, MeOH); ^1^H NMR (600 MHz, DMSO) δ 10.66 (s, 1H), 10.44 (s, 1H), 9.66 (s, 1H), 8.81 (d, *J* = 2.7 Hz, 1H), 8.15 (d, *J* = 3.0 Hz, 1H), 8.06 (dd, *J* = 8.5, 2.6 Hz, 1H), 7.88 − 7.79 (m, 2H), 7.75 − 7.71 (m, 1H), 7.64 (s, 1H), 7.59 (d, *J* = 8.2 Hz, 1H), 7.29 (d, *J* = 8.4 Hz, 1H), 7.27 − 7.16 (m, 5H), 7.14 (t, *J* = 7.3 Hz, 1H), 4.70 (d, *J* = 13.7 Hz, 1H), 4.55 − 4.45 (m, 1H), 3.55 (d, *J* = 14.0 Hz, 1H), 3.44 − 3.40 (m, 1H), 3.34 (s, 3H), 3.16 (dt, *J* = 15.0, 7.5 Hz, 1H), 2.41 (s, 6H), 2.13 (s, 3H); ^13^C NMR (151 MHz, DMSO) δ 169.93, 167.27, 164.76, 159.28, 157.37, 153.59, 152.45, 150.45, 141.52, 140.14, 139.58, 138.08, 137.61, 137.59, 135.14, 131.19, 131.12, 130.88, 129.90, 129.87, 129.14, 128.40, 126.75, 126.30, 122.93, 122.23, 120.87, 119.95, 119.42, 119.38, 103.34, 64.85, 46.99, 37.34, 28.61, 25.86, 23.49, 17.16; IR (Neat) 3279, 2921, 1668, 1598, 1531, 1494, 1408, 1239, 1186, 1143, 1112, 1071, 1026, 826, 785, 732, 698 cm^−1^; HRMS (MM: ESI-APCI+) m/z calc’d for C_38_H_36_N_9_O_3_ [M + H]^+^: 666.2941; found: 666.2947.

#### 4.1.43. (S)-3-isobutyl-1,5-dimethyl-N-(4-methyl-3-(1-methyl-7-((6-methylpyridin-3-yl)amino)-2-oxo-1,4-dihydropyrimido[4,5-d]pyrimidin-3(2H)-yl)phenyl)-2-oxo-2,3-dihydro-1H-benzo[e][1,4]diazepine-8-carboxamide (11m)

(Purity: 98%; HPLC); *R_f_:* 0.35 (1:10 MeOH:CH_2_Cl_2_); [α]_D_^30^ = +36.0 (*c* 0.0500, MeOH); ^1^H NMR (600 MHz, DMSO) δ 10.45 (s, 1H), 9.64 (s, 1H), 8.80 (d, *J* = 2.7 Hz, 1H), 8.16 (s, 1H), 8.05 (dd, *J* = 8.5, 2.7 Hz, 1H), 7.95 (s, 1H), 7.88 (d, *J* = 8.1 Hz, 1H), 7.85 − 7.77 (m, 2H), 7.68 − 7.61 (m, 1H), 7.32 (d, *J* = 8.4 Hz, 1H), 7.17 (d, *J* = 8.4 Hz, 1H), 4.70 (d, *J* = 13.9 Hz, 1H), 4.53 (d, *J* = 14.0 Hz, 1H), 3.40 (m, 1H), 3.36 (s, 3H), 3.34 (s, 3H), 2.43 (s, 3H), 2.40 (s, 3H), 2.14 (s, 3H), 1.93 (ddd, *J* = 13.5, 8.4, 5.2 Hz, 1H), 1.72 (ddp, *J* = 33.0, 13.6, 6.6, 6.1 Hz, 2H), 0.85 (d, *J* = 6.4 Hz, 3H), 0.70 (d, *J* = 6.3 Hz, 3H); ^13^C NMR (151 MHz, DMSO) δ 170.07, 167.18, 164.65, 159.31, 157.39, 153.61, 152.48, 150.57, 141.77, 141.54, 140.39, 138.00, 137.50, 135.06, 132.63, 131.27, 131.14, 128.14, 126.59, 123.59, 122.82, 121.23, 121.20, 120.14, 119.66, 103.31, 60.66, 47.00, 35.06, 28.61, 25.44, 24.23, 23.60, 23.58, 22.11, 17.17; IR (Neat) 3304, 2954, 1666, 1602, 1535, 1508, 1413, 1319, 1187, 1144, 1120, 1023, 827, 786, 746 cm^−1^; HRMS (MM: ESI-APCI+) m/z calc’d for C_36_H_40_N_9_O_3_ [M + H]^+^: 646.3254; found: 646.3265.

#### 4.1.44. (S)-3-isobutyl-1-(2-methoxyethyl)-N-(4-methyl-3-(1-methyl-7-((6-methylpyridin-3-yl)amino)-2-oxo-1,4-dihydropyrimido[4,5-d]pyrimidin-3(2H)-yl)phenyl)-2-oxo-5-phenyl-2,3-dihydro-1H-benzo[e][1,4]diazepine-8-carboxamide (11n)

(Purity: 98%; HPLC); *R_f_:* 0.35 (1:10 MeOH:CH_2_Cl_2_); [α]_D_^23^ = +20.3 (*c* 0.0720, MeOH); ^1^H NMR (600 MHz, DMSO) δ 10.51 (s, 1H), 9.65 (s, 1H), 8.80 (d, *J* = 2.7 Hz, 1H), 8.16 (d, *J* = 2.8 Hz, 2H), 8.05 (dd, *J* = 8.5, 2.7 Hz, 1H), 7.85 − 7.76 (m, 2H), 7.64 (dd, *J* = 8.4, 2.2 Hz, 1H), 7.55 − 7.50 (m, 3H), 7.49 − 7.44 (m, 2H), 7.39 (d, *J* = 8.1 Hz, 1H), 7.33 (d, *J* = 8.4 Hz, 1H), 7.18 (d, *J* = 8.5 Hz, 1H), 4.71 (d, *J* = 14.0 Hz, 1H), 4.54 (d, *J* = 14.0 Hz, 1H), 4.40 (dt, *J* = 14.5, 5.6 Hz, 1H), 3.99 (ddd, *J* = 14.4, 6.5, 4.2 Hz, 1H), 3.58 (dd, *J* = 8.7, 4.5 Hz, 1H), 3.42 − 3.35 (m, 2H), 3.34 (s, 3H), 2.95 (s, 3H), 2.41 (s, 3H), 2.14 (s, 3H), 2.11 (q, *J* = 8.8 Hz, 1H), 1.86 − 1.80 (m, 2H), 0.93 (d, *J* = 5.9 Hz, 3H), 0.76 (d, *J* = 5.9 Hz, 3H); ^13^C NMR (151 MHz, DMSO) δ 169.32, 167.58, 164.67, 159.31, 157.39, 153.62, 152.48, 150.58, 142.78, 141.56, 140.39, 138.48, 138.04, 138.03, 137.81, 135.06, 132.37, 131.29, 131.16, 131.14, 130.78, 129.81, 129.42, 128.69, 126.59, 123.91, 122.86, 122.83, 120.16, 120.14, 119.64, 119.60, 103.31, 69.70, 61.57, 58.27, 47.01, 28.61, 24.63, 23.67, 23.60, 22.29, 17.18; IR (Neat) 3292, 2953, 2360, 1662, 1598, 1533, 1492, 1411, 1293, 1238, 1187, 1144, 1119, 1022, 909, 825, 784, 737, 697 cm^−1^; HRMS (MM: ESI-APCI+) m/z calc’d for C_43_H_46_N_9_O_4_ [M + H]^+^: 752.3673; found: 752.3667.

#### 4.1.45. (S)-1-(2-(benzylamino)-2-oxoethyl)-3-isobutyl-N-(4-methyl-3-(1-methyl-7-((6-methylpyridin-3-yl)amino)-2-oxo-1,4-dihydropyrimido[4,5-d]pyrimidin-3(2H)-yl)phenyl)-2-oxo-5-phenyl-2,3-dihydro-1H-benzo[e][1,4]diazepine-8-carboxamide (11o)

(Purity: 96%; HPLC); *R_f_:* 0.35 (1:10 MeOH:CH_2_Cl_2_); [α]_D_^30^ = +39.0 (*c* 0.0670, MeOH); ^1^H NMR (600 MHz, DMSO) δ 10.54 (s, 1H), 9.65 (s, 1H), 8.81 (d, *J* = 2.8 Hz, 1H), 8.67 (t, *J* = 6.0 Hz, 1H), 8.17 (s, 1H), 8.09 − 7.99 (m, 2H), 7.82 (q, *J* = 2.4 Hz, 1H), 7.80 − 7.77 (m, 1H), 7.63 (dd, *J* = 8.1, 2.4 Hz, 1H), 7.56 − 7.53 (m, 2H), 7.51 (t, *J* = 7.3 Hz, 1H), 7.45 (t, *J* = 7.5 Hz, 2H), 7.40 (d, *J* = 8.1 Hz, 1H), 7.34 (d, *J* = 8.5 Hz, 1H), 7.25 (dt, *J* = 15.7, 7.6 Hz, 4H), 7.18 (dd, *J* = 8.1, 4.6 Hz, 2H), 4.66 (dtd, *J* = 46.8, 16.2, 15.1, 3.8 Hz, 3H), 4.55 (d, *J* = 14.0 Hz, 1H), 4.34 (dd, *J* = 15.4, 6.0 Hz, 1H), 4.26 (dd, *J* = 15.4, 5.7 Hz, 1H), 3.66 (qd, *J* = 4.5, 1.9 Hz, 1H), 3.35 (s, 3H), 2.41 (s, 3H), 2.15 (s, 3H), 2.13 (d, *J* = 8.8 Hz, 1H), 1.90 − 1.82 (m, 2H), 0.95 (d, *J* = 6.1 Hz, 3H), 0.78 (d, *J* = 5.9 Hz, 3H); ^13^C NMR (151 MHz, DMSO) δ 169.56, 167.99, 167.90, 164.84, 159.32, 157.39, 153.62, 152.49, 150.55, 143.08, 141.58, 140.35, 139.43, 138.73, 138.07, 138.05, 137.94, 135.08, 131.62, 131.32, 131.19, 130.70, 130.04, 129.66, 128.61, 128.59, 127.44, 127.08, 126.63, 123.43, 122.84, 122.13, 122.12, 120.09, 119.60, 119.56, 103.30, 63.16, 61.40, 50.68, 47.02, 42.48, 40.22, 28.61, 24.61, 23.73, 23.57, 22.25, 17.18; IR (Neat) 3294, 2953, 2360, 1661, 1597, 1531, 1409, 1292, 1233, 1187, 1144, 1119, 1028, 908, 826, 784, 736, 697 cm^−1^; HRMS (MM: ESI-APCI+) m/z calc’d for C_49_H_49_N_10_O_4_ [M + H]^+^: 841.3938; found: 841.3947.

#### 4.1.46. (S)-2-(5-(but-3-en-1-yl)-1-methyl-2-oxo-2,3-dihydro-1H-benzo[e][1,4]diazepin-3-yl)-N-(4-methyl-3-(1-methyl-7-((6-methylpyridin-3-yl)amino)-2-oxo-1,4-dihydropyrimido[4,5-d]pyrimidin-3(2H)-yl)phenyl)acetamide (11p)

(Purity: 97%; HPLC); *R_f_:* 0.35 (1:10 MeOH:CH_2_Cl_2_); [α]_D_^30^ = −17.0 (*c* 0.0780, MeOH); ^1^H NMR (600 MHz, DMSO) δ 10.18 (s, 1H), 9.69 (s, 1H), 8.83 (s, 1H), 8.13 (s, 1H), 8.08 (d, *J* = 8.5 Hz, 1H), 7.71 (d, *J* = 7.8 Hz, 1H), 7.67 (d, *J* = 14.5 Hz, 1H), 7.60 (t, *J* = 7.9 Hz, 1H), 7.50 (d, *J* = 8.4 Hz, 1H), 7.32 (t, *J* = 8.2 Hz, 2H), 7.22 (dd, *J* = 14.1, 8.4 Hz, 2H), 5.67 (dt, *J* = 16.6, 8.4 Hz, 1H), 4.84 (d, *J* = 13.6 Hz, 2H), 4.63 (d, *J* = 14.0 Hz, 1H), 4.47 (d, *J* = 14.1 Hz, 1H), 3.90 (d, *J* = 7.2 Hz, 1H), 3.31 (s, 3H), 3.25 (s, 3H), 3.12 (td, *J* = 15.8, 6.9 Hz, 1H), 2.97 (ddt, *J* = 30.1, 14.6, 7.4 Hz, 2H), 2.75 (dt, *J* = 15.4, 7.9 Hz, 1H), 2.42 (s, 3H), 2.21 (m, 1H), 2.14 (m, 1H), 2.08 (s, 3H); ^13^C NMR (151 MHz, DMSO) δ 170.61, 169.82, 169.67, 159.18, 157.39, 153.53, 152.39, 150.08, 142.17, 141.47, 138.45, 137.66, 131.59, 131.07, 130.00, 129.83, 127.62, 127.32, 124.88, 123.29, 122.25, 118.45, 118.04, 115.54, 103.48, 59.82, 46.92, 39.00, 36.78, 34.93, 31.22, 31.20, 28.58, 23.16, 18.45, 17.04; IR (Neat) 3305, 2924, 2361, 1666, 1601, 1535, 1493, 1413, 1293, 1235, 1194, 1144, 1117, 1025, 845, 750 cm^−1^; HRMS (MM: ESI-APCI+) m/z calc’d for C_36_H_38_N_9_O_3_ [M + H]^+^: 644.3098; found: 644.3099.

#### 4.1.47. (S)-2-(1-benzyl-5-(but-3-en-1-yl)-2-oxo-2,3-dihydro-1H-benzo[e][1,4]diazepin-3-yl)-N-(4-methyl-3-(1-methyl-7-((6-methylpyridin-3-yl)amino)-2-oxo-1,4-dihydropyrimido[4,5-d]pyrimidin-3(2H)-yl)phenyl)acetamide (11q)

(Purity: 98%; HPLC); *R_f_:* 0.35 (1:10 MeOH:CH_2_Cl_2_); [α]_D_^25^ = +45.8 (*c* 0.0610, MeOH); ^1^H NMR (400 MHz, DMSO) δ 10.20 (s, 1H), 9.63 (s, 1H), 8.79 (d, *J* = 2.6 Hz, 1H), 8.13 (d, *J* = 1.7 Hz, 1H), 8.05 (dd, *J* = 8.4, 2.7 Hz, 1H), 7.72 − 7.62 (m, 2H), 7.62 − 7.48 (m, 2H), 7.34 (td, *J* = 8.3, 2.2 Hz, 1H), 7.27 (t, *J* = 7.4 Hz, 1H), 7.24 − 7.13 (m, 5H), 7.03 (d, *J* = 7.3 Hz, 2H), 5.79 − 5.66 (m, 1H), 5.33 (dd, *J* = 15.7, 3.8 Hz, 1H), 4.97 − 4.85 (m, 3H), 4.63 (d, *J* = 14.1 Hz, 1H), 4.48 (d, *J* = 14.1 Hz, 1H), 4.02 (t, *J* = 7.1 Hz, 1H), 3.34 (s, 3H), 3.22 (ddd, *J* = 16.8, 9.9, 7.4 Hz, 1H), 3.10 − 2.99 (m, 1H), 2.79 − 2.70 (m, 2H), 2.40 (s, 3H), 2.09 (s, 3H), 1.98 (q, *J* = 7.5 Hz, 2H); ^13^C NMR (101 MHz, DMSO) δ 170.79, 169.79, 169.35, 169.33, 159.41, 157.50, 153.66, 152.53, 150.67, 141.62, 140.53, 140.51, 138.54, 138.04, 137.62, 135.18, 131.66, 131.18, 131.14, 130.15, 128.79, 127.72, 127.61, 127.56, 126.69, 125.49, 122.98, 122.91, 118.63, 118.27, 115.55, 103.42, 59.96, 49.63, 47.06, 39.01, 36.96, 31.09, 31.07, 28.67, 23.70, 17.16; IR (Neat) 3300, 2923, 1667, 1599, 1534, 1491, 1449, 1409, 1330, 1290, 1236, 1186, 1143, 1116, 1079, 1024, 914, 826, 734, 699 cm^−1^; HRMS (MM: ESI-APCI+) m/z calc’d for C_42_H_42_N_9_O_3_ [M + H]^+^: 720.3411; found: 720.3410.

### 4.2. Computational methods

For molecular modelling studies, crystal structures of Lck (PDB: 2PL0), c-Src (PDB: 3OEZ), p38a (PDB: 3HEC) and Abl1 (PDB: 2HYY) were downloaded from the RCSB protein data bank homepage (https://www.rcsb.org). Only the crystal structures bound with Imatinib were selected to obtain the DFG-out state of kinases. The chicken c-Src crystal structure was converted into a human c-Src structure using a homology modelling method. The missing activation loop in p38a crystal structure was restored based on the crystal structure of Lck using Prime loop prediction application^17^. For protein-ligand complex prediction, Glide docking application^18^ in Schrödinger suite was employed. OPLS3e force field and SP mode of Glide were used. Flexible ligand sampling was allowed. Molecular dynamics simulation was performed using Desmond molecular dynamics package^19^ in Schrödinger suite. The kinase-inhibitor complexes were solvated using TIP3P water model and the solvated MD systems were described using OPLS3e force field. The Nose-Hoover chain thermostat and the Martyna-Tobias-Klein barostat methods were used to maintain the system temperature at 300 K and system pressure at 1 bar, respectively. A periodic boundary condition was employed. MD simulations were performed for 3 μs simulation time. Binding free energies of inhibitors were calculated by Prime molecular mechanics/generalized Born surface area (MM/GBSA) application with VSGB2.0 implicit solvation model. WaterMap^20^ application in Schrödinger suites was used.

### 4.3. Biology

#### 4.3.1. *In vitro* kinase assay

Full panel kinase profiling and kinase IC_50_ measurement were performed by using Reaction Biology Corp. (San Diego, USA).

#### 4.3.2. Cell culture and sample treatment

Jurkat cells were purchased from the Korea Cell Line Bank (Seoul, Republic of Korea) and maintained in RPMI-1640 medium containing 10% FBS, streptomycin sulphate, penicillin, HEPES, and sodium bicarbonate in a 5% CO_2_ atmosphere at 37 °C. Jurkat cells were stimulated with 1 μg/ml of plate-bound anti-CD3 mAb (clone HIT3a, BD Bioscience) and then incubated with KITS 1–001 (10, 50, or 100 μM) for 30 min. KIST 1–001 were dissolved in DMSO and added to the culture media in serial dilution (the final concentration of DMSO in all experiments did not exceed 0.1%).

#### 4.3.3. Western blot analysis

The protein of Jurkat cells was extracted using a PRO-PREP (Intron Biotechnology, Seoul, Republic of Korea). The protein concentration was determined using Bio-Rad protein assay reagent according to the manufacturer’s instruction and BSA (Bio-Rad, Hercules, CA, USA) was used as a standard for quantification. Equal protein amounts were separated by 10% sodium dodecyl sulphate-polyacrylamide gel electrophoresis (SDS-PAGE) and transferred to PVDF membranes. The membranes were incubated for 1 h with blocking solution (5% skim milk) at room temperature and followed by incubation for primary antibodies overnight at 4 °C. p-Lck (Y394) antibody (1:500, MBS128234, MyBioSource) was used as a primary antibody, and β-actin (1:1000, sc-81178, Santa Cruz Biotechnology) was used as an internal control. And then membranes were incubated with a 1:2000 dilution of horseradish peroxidase-conjugated secondary antibody for 2 h at room temperature. The membranes were analysed using an enhanced chemiluminescence (ECL) substrate and imaged by LAS-4000 luminescent image analyser (FUJIFILM, Tokyo, Japan).

#### 4.3.4. Animals

Male C57BL/6 mice (21 ± 2 g; 6 weeks) were obtained from Oriental Bio Inc. (Seongnam-si, Korea). All mice were bred under constant conditions (temperature: 22 ± 2 °C, humidity: 40–60%, light/dark cycle: 12 h). All animal experiments were conducted under the university guidelines of the ethical committee for Animal Care and Use of the Kyung Hee University (KHSASP-22–002).

#### 4.3.5. Induction of colitis by dextran sulphate sodium (DSS) and treatment

**Figure 8.**
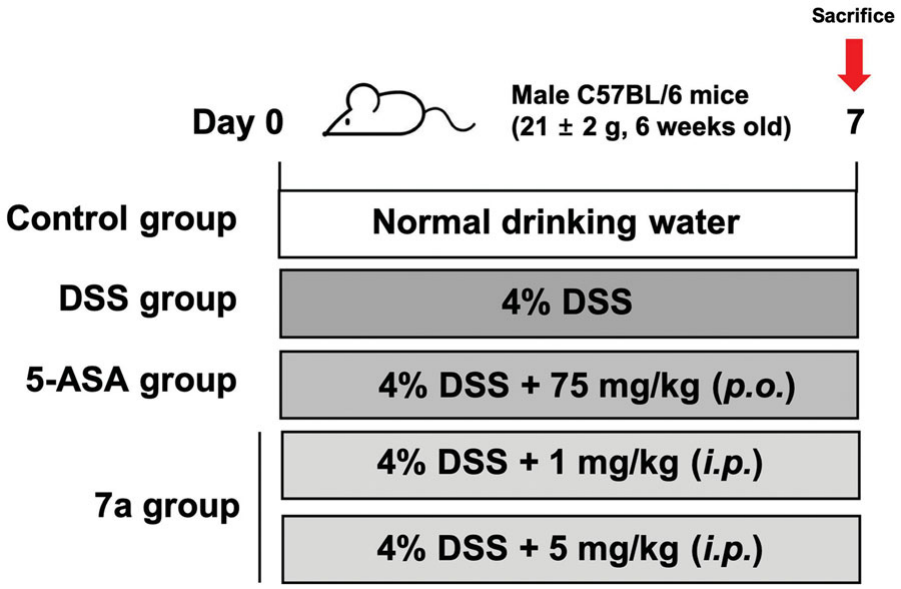
Schematic diagram of DSS-induced colitis mouse model.

Colitis in mice was induced by providing water containing 4% (w/v) DSS for 7 days. Mice were randomly divided into 5 groups (*n* = 6/group, Figure 8) as follows: control group treated with vehicle; DSS plus vehicle group exposed to 4% DSS and treated with vehicle; the other 3 groups consist of mice receiving 4% DSS was treated with 5-ASA (75 mg/kg/day, *p.o.*) as a positive control or **7a** (1, 5 mg/kg/day, *i.p.*) daily for 7 days.

#### 4.3.6. Assessment of the disease activity index (DAI)

To calculate the severity of colitis, body weight, stool consistency, and occult/gross bleeding of all mice were assessed. DAI score was measured every day according to the following table (Table 5). The colon length was measured at end of the experiment.

#### 4.3.7. Statistical analysis

Results are expressed as the mean ± SE of triplicate experiments with similar patterns. Statistically significant values were compared using ANOVA and Dunnett’s *post hoc* test, and *p* values of less than 0.05 were considered statistically significant.

## Supplementary Material

Supplemental Material
