# Supplementary material for: Identification of highly selective type II kinase inhibitors with chiral peptidomimetic tails
Source: J Enzyme Inhib Med Chem. 2022 Apr 28;37(1):1257–77. doi: 10.1080/14756366.2022.2068148 (PMC9067983; doi:10.1080/14756366.2022.2068148)
Supplement: Supplemental Material [file IENZ_A_2068148_SM5927.pdf]

Supplemental material for

Identification of Highly Selective Type II Kinase Inhibitors with Chiral Peptidomimetic Tails

Seo-Jung Han*[a,b], Jae Eun Jung[a,c], Do Hee Oh[a,c], Minsup Kim[d], Jae-Min Kim[e,f], Kyung-Sook Chung[e], Hee-Soo Han[e,g], Jung-Hun Lee[e,g], Kyung-Tae Lee[e,f], Hee Jin Jeong[a], In Ho Park[h,i], Eunkyeong Jeon[i,j], Jeon-Soo Shin[h,i,j], Dongkeun Hwang[a], Art E. Cho[d,k], Duck-Hyung Lee[c], and Taebo Sim*[a,h]

[a]*Chemical Kinomics Research Center, Korea Institute of Science and Technology, 5 Hwarang-ro 14-gil, Seongbuk-gu, Seoul, 02792, Republic of Korea*
[b]*Division of Bio-Medical Science & Technology, KIST School, UST, 5 Hwarang-ro 14-gil, Seoungbuk-gu, Seoul, 02792, Republic of Korea*
[c]*Department of Chemistry, Sogang University, 35 Baekbeom Ro, Seoul, 04107, Republic of Korea*
[d]*Drug Discovery Institute, inCerebro Co., Ltd. 232, Gongneung-ro, Nowon-gu, Seoul, 01811, Republic of Korea*
[e]*Department of Pharmaceutical Biochemistry, College of Pharmacy, Kyung Hee University, 26 Kyungheedae-ro, Seoul 02447, Republic of Korea*
[f]*Department of Biomedical and Pharmaceutical Sciences, Graduate School, Kyung Hee University, 26 Kyungheedae-ro,, Seoul 02447, Republic of Korea*
[g]*Department of Life and Nanopharmaceutical Science, College of Pharmacy, Kyung Hee University, 2 6 Kyungheedae-ro, Seoul 02447, Republic of Korea*
[h]*Severance Biomedical Science Institute, Yonsei University College of Medicine, Seoul, 03722, Republic of Korea*
[i]*Institute of Immunology and Immunological Diseases, Yonsei University College of Medicine, Seoul, 03722, Republic of Korea*
[j]*Department of Microbiology, Yonsei University College of Medicine, Seoul, 03722, Republic of Korea*
[k]*Department of Bioinformatics, Korea University, Sejong, 30019, Republic of Korea*

* *Corresponding Authors; e-mail:sjhan@kist.re.kr*

Table of Contents:

In vitro selectivity profiling of **7a** at 1μM concentration against 373 kinases at 10μM ATP concentration……**S-2**
Computational Figures……**S-6**
^{1}H NMR, ^{13}C NMR, and ^{19}F NMR Spectra……**S-7**
HPLC traces of **7** and **11**……**S-100**

Table S1. *in vitro* selectivity profiling of **7a** at 1μM concentration against 373 kinases at 10μM ATP concentration.

Kinase	% inhibition (relative to DMSO)	Kinase	% inhibition (relative to DMSO)	Kinase	% inhibition (relative to DMSO)
LCK	94	FYN	21	AKT3	10
DDR1	80	EPHA2	21	GSK3a	10
FGR	80	FLT4/VEGFR3	20	MSSK1/STK23	10
BMX/ETK	73	DYRK1/DYRK1A	20	SIK1	10
BLK	73	WNK3	19	CAMKK2	10
LYN	73	FLT3	18	CTK/MATK	10
RAF1	69	FES/FPS	18	ERBB2/HER2	10
c-Src	64	ALK1/ACVRL1	17	ROCK2	10
ABL1	63	CLK3	17	BMPR2	10
YES/YES1	62	RSK1	17	GLK/MAP4K3	9
FMS	60	STK32B/YANK2	16	SNRK	9
DDR2	58	EPHB1	16	RET	9
ERBB4/HER4	53	ALK6/BMPR1B	16	ROS/ROS1	9
LIMK1	51	EPHB4	16	PAK5	9
MEK5	50	MLK2/MAP3K10	16	ARK5/NUAK1	9
PDGFRb	48	RIPK4	15	PKCG	9
CSK	44	CAMK1b	15	JAK1	9
PEAK1	44	FRK/PTK5	15	MSK2/RPS6KA4	9
HCK	38	p38α/MAPK14	15	SLK/STK2	9
FGFR1	37	c-MET	14	FLT1/VEGFR1	9
LYN B	34	ZAK/MLTK	14	AKT1	8
MLK3/MAP3K11	34	IRAK1	14	BRSK2	8
ARAF	34	ROCK1	13	CAMK2a	8
MLK1/MAP3K9	33	p38β/MAPK11	13	PKMYT1	8
PDGFRa	31	GCK/MAP4K2	13	MNK1	8
EPHA4	29	KDR/VEGFR2	12	DYRK2	8
EPHB2	29	LIMK2	12	MAPKAPK5/PRAK	8
RON/MST1R	27	FGFR2	12	PYK2	8
c-Kit	27	SIK3	12	PKN3/PRK3	8
PKCeta	27	MEKK3	11	CAMK1d	8
EPHA3	25	MEK2	11	MEK1	8
KHS/MAP4K5	23	JAK2	11	EPHB3	8
EPHA5	23	CK1d	11	NEK9	8
DYRK3	23	DYRK1B	11	MKK6	8
TRKC	21	PKG2/PRKG2	11	MYLK4	8

Table S2. *in vitro* selectivity profiling of **7a** at 1μM concentration against 373 kinases at 10μM ATP concentration. (cont.)

Kinase	% inhibition (relative to DMSO)	Kinase	% inhibition (relative to DMSO)	Kinase	% inhibition (relative to DMSO)
MST2/STK3	7	FGFR4	5	STK25/YSK1	3
MUSK	7	IRR/INSRR	5	CK2a2	3
PHKg1	7	KSR2	5	ALK3/BMPR1A	3
CDK1/cyclin B	7	PAK1	5	CAMK2b	3
ITK	7	MST1/STK4	5	NLK	3
ERK1	7	ERK5/MAPK7	5	IKKe/IKBKE	3
WNK2	7	SRPK1	5	CDK7/cyclin H	3
TTBK2	7	FAK/PTK2	5	PKCiota	3
MLK4	7	ERN1/IRE1	5	ERK7/MAPK15	3
BRAF	7	MELK	4	SIK2	2
CAMK1g	7	MARK3	4	ERN2/IRE2	2
MASTL	7	GRK6	4	TNK1	2
CDK17/cyclin Y (PCTK2)	7	TAOK2/TAO1	4	STK32C/YANK3	2
CDK16/cyclin Y (PCTAIRE)	6	NIM1	4	JNK2	2
PKA	6	LATS1	4	ALK2/ACVR1	2
CDK5/p35	6	IRAK4	4	MKK4	2
IKKa/CHUK	6	TAK1	4	EPHA7	2
OSR1/OXSR1	6	SRMS	4	PIM1	2
Aurora B	6	PKCb1	4	MAPKAPK3	2
CDK14/cyclin Y (PFTK1)	6	MAPKAPK2	4	MKK7	2
SGK3/SGKL	6	TESK2	4	TEC	2
SNARK/NUAK2	6	STK38L/NDR2	4	NEK7	2
TNIK	6	CDK18/cyclin Y (PCTK3)	4	CDK8/cyclin C	2
ZIPK/DAPK3	6	P38g	4	BRSK1	2
STK22D/TSSK1	6	CHK2	4	CK1g2	2
MRCKa/CDC42BPA	6	CDK4/cyclin D3	3	KSR1	2
BTK	6	PLK3	3	MINK/MINK1	2
ULK2	5	MEKK1	3	Haspin	1
TRKB	5	DYRK4	3	NEK11	1
CLK1	5	PKAcb	3	NEK8	1
CK2a	5	LOK/STK10	3	CAMK1a	1
EPHA6	5	EPHA1	3	JNK3	1
PLK4/SAK	5	TSSK3/STK22C	3	PDK1/PDPK1	1

Table S3. *in vitro* selectivity profiling of **7a** at 1μM concentration against 373 kinases at 10μM ATP concentration (cont.)

Kinase	% inhibition (relative to DMSO)	Kinase	% inhibition (relative to DMSO)	Kinase	% inhibition (relative to DMSO)
PKCepsilon	1	HIPK2	0	CK1epsilon	0
c-MER	1	TESK1	0	ULK3	0
GRK4	1	HIPK1	0	RSK3	0
LATS2	1	TLK1	0	HGK/MAP4K4	0
RIPK2	1	MRCKb/CDC42BPB	0	NEK5	0
PKN1/PRK1	1	EGFR	0	IR	0
TYRO3/SKY	1	ASK1/MAP3K5	0	PAK4	0
MNK2	1	VRK1	0	P38d/MAPK13	0
DAPK2	1	HIPK3	0	Aurora C	0
TYK2	1	PIM3	0	HPK1/MAP4K1	0
GRK5	1	PKCmu/PRKD1	0	CDC7/DBF4	0
HIPK4	1	CDK6/cyclin D1	0	CDK9/cyclin T1	0
CDK2/Cyclin A1	1	DMPK2	0	LKB1	0
COT1/MAP3K8	1	TBK1	0	PLK1	0
EPHA8	0	TTBK1	0	Aurora A	0
STK38/NDR1	0	SBK1	0	p70S6K/RPS6KB1	0
MEKK6	0	GSK3b	0	ALK	0
PKG1a	0	MST3/STK24	0	GRK3	0
ACK1	0	PASK	0	CK1g1	0
RIPK3	0	ALK5/TGFBR1	0	MYO3b	0
CAMK2g	0	CLK2	0	PAK2	0
PKCb2	0	GRK7	0	ALK4/ACVR1B	0
CLK4	0	PHKg2	0	PKD2/PRKD2	0
LRRK2	0	PKCd	0	MARK4	0
DMPK	0	SYK	0	PIM2	0
TXK	0	MARK1	0	SRPK2	0
CDK2/cyclin A	0	CDK2/cyclin E2	0	CDK2/cyclin O	0
CAMK2d	0	p70S6Kb/RPS6KB2	0	STK39/STLK3	0
NEK2	0	STK16	0	CDK3/cyclin E	0
LCK2/ICK	0	TSSK2	0	CDK9/cyclin K	0
MEKK2	0	NEK1	0	MST4	0
STK21/CIT	0	SGK2	0	FGFR3	0
MSK1/RPS6KA5	0	TLK2	0	PKG1b	0
TYK1/LTK	0	FER	0	CDK6/cyclin D3	0

Table S4. *in vitro* selectivity profiling of **7a** at 1μM concentration against 373 kinases at 10μM ATP concentration (cont.)

Kinase	% inhibition (relative to DMSO)	Kinase	% inhibition (relative to DMSO)	Kinase	% inhibition (relative to DMSO)
TAOK3/JIK	0	CAMKK1	0		
DAPK1	0	RSK2	0		
TAOK1	0	PKCtheta	0		
PAK3	0	MYLK3	0		
ZAP70	0	WEE1	0		
DLK/MAP3K12	0	CAMK4	0		
NEK6	0	CDK19/cyclin C	0		
SGK1	0	DCAMKL1	0		
CDK3/cyclin E2	0	CDK5/P25	0		
PKAcg	0	MLCK/MYLK	0		
PKCnu/PRKD3	0	JAK3	0		
MLCK2/MYLK2	0	PBK/TOPK	0		
NEK4	0	MEK3	0		
RSK4	0	YSK4/MAP3K19	0		
IKKb/IKBKB	0	CDK4/cyclin D1	0		
GRK1	0	ULK1	0		
GRK2	0	CHK1	0		
TIE2/TEK	0	AXL	0		
PLK2	0	CK1a1L	0		
ERK2/MAPK1	0	RIPK5	0		
BRK	0	PRKX	0		
MAK	0	CDK1/cyclin A	0		
MARK2/PAR-1Ba	0	PKCzeta	0		
CDK2/cyclin E	0	VRK2	0		
DCAMKL2	0	WNK1	0		
IGF1R	0	CK1a1	0		
MYO3A	0	PKN2/PRK2	0		
SSTK/TSSK6	0	TRKA	0		
PKCa	0	CK1g3	0		
JNK1	0	PAK6	0		
AKT2	0	STK33	0		
CDK9/cyclin T2	0	DRAK1/STK17A	0		
CDK1/cyclin E	0	TGFBR2	0		
NEK3	0				

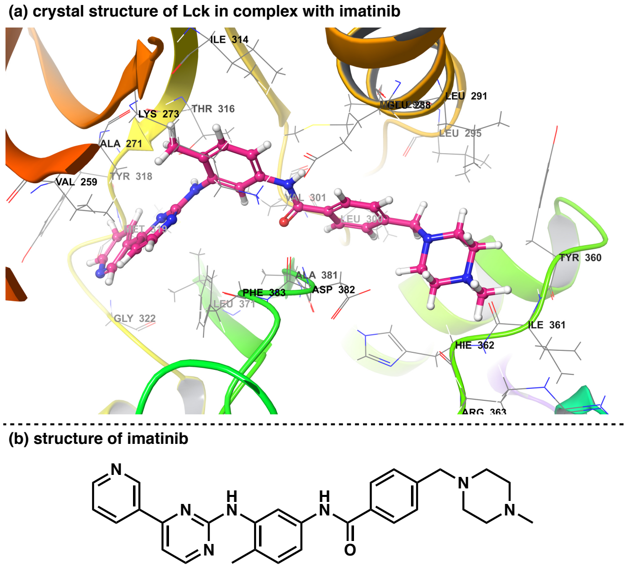

imatinib

Figure S1. (a) Co-crystal structure of Lck in complex with Imatinib. (PDB: 2PL0) (b) Structure of imatinib.

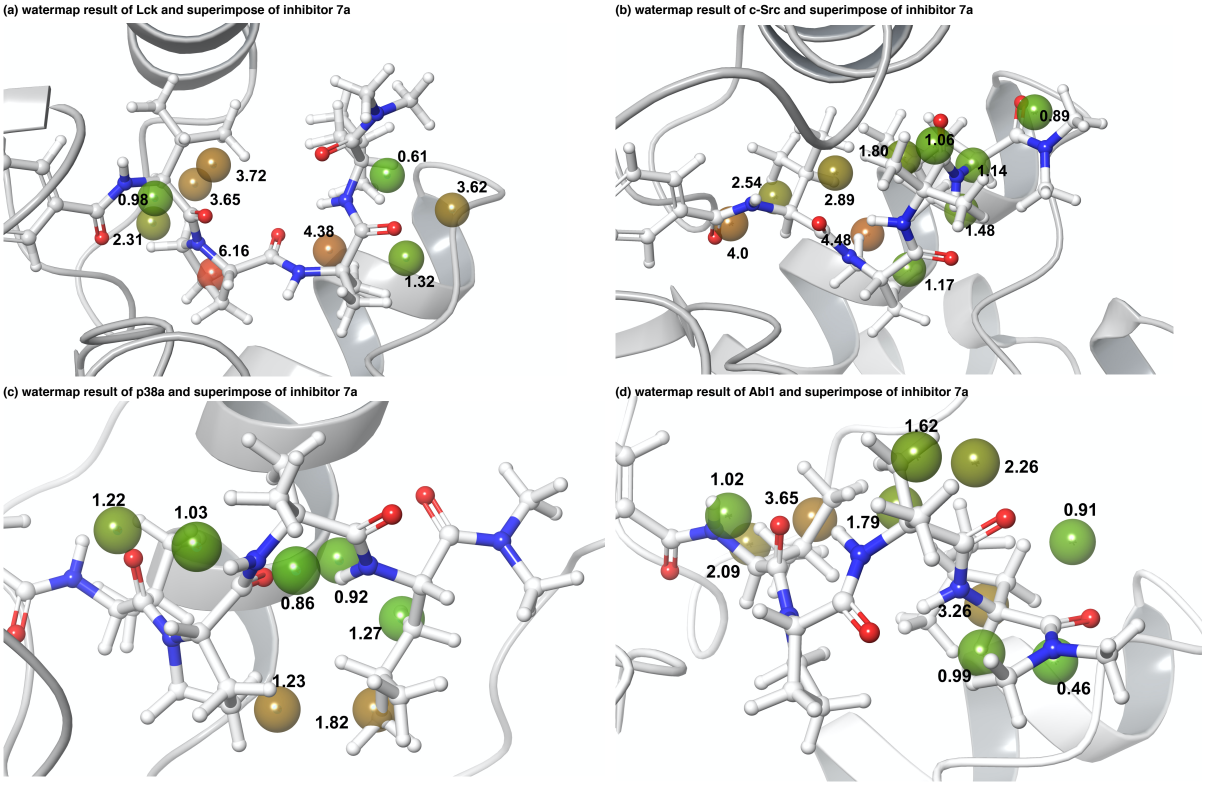

Figure S2. (a) The predicted binding mode of **7a** with Lck using WaterMap application. (b) The predicted binding mode of **7a** with c-Src using WaterMap application. (c) The predicted binding mode of **7a** with p38α using WaterMap application. (d) The predicted binding mode of **7a** with Abl1 using WaterMap application.; Colored sphere: hydration sites occupied by the inhibitor. The number on the sphere: ΔG energy (kcal/mol). Green sphere: low energy around 0 kcal/mol. Red sphere: high energy over 5 kcal/mol.

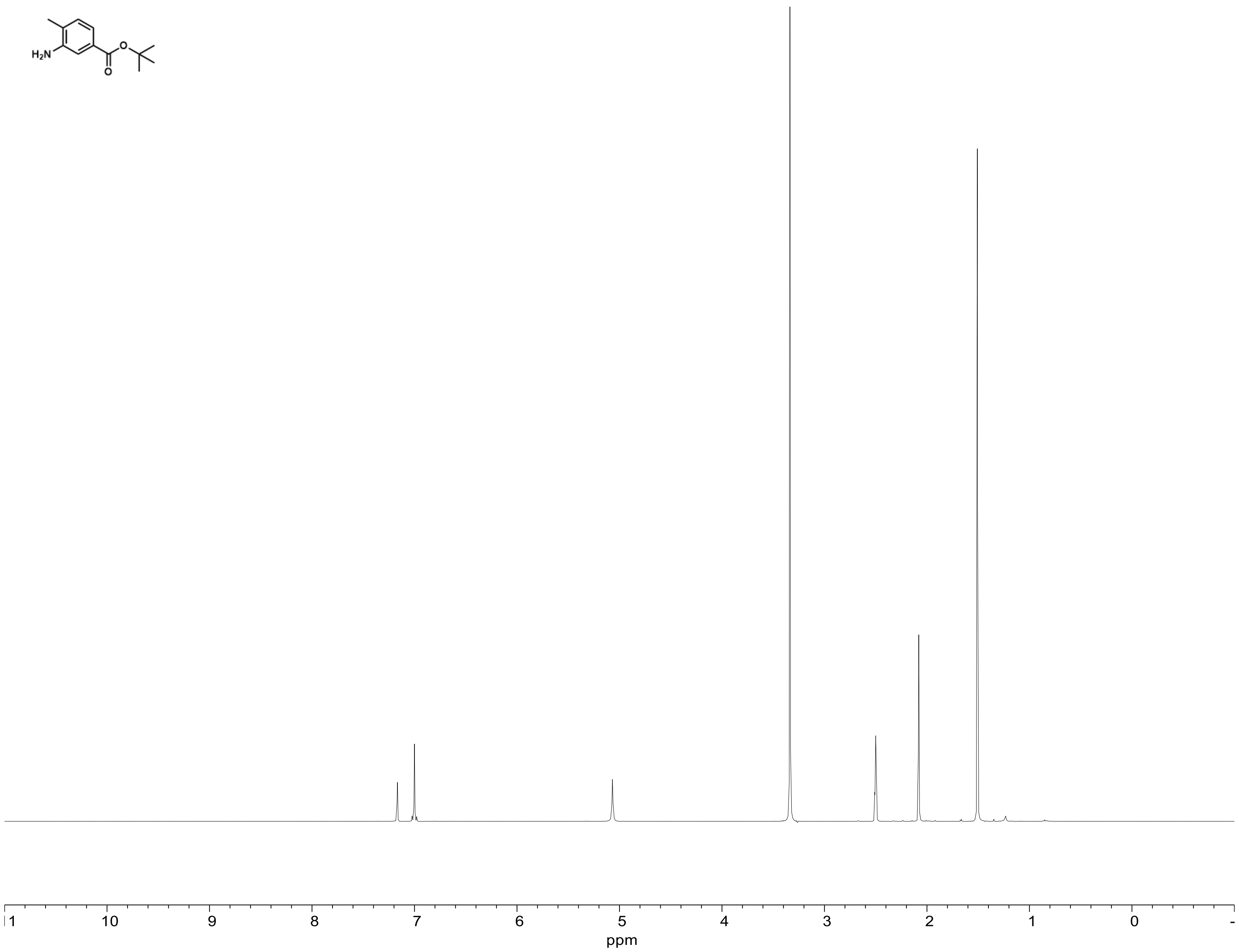

^{1}H NMR (400 MHz, DMSO) of compound **1**.

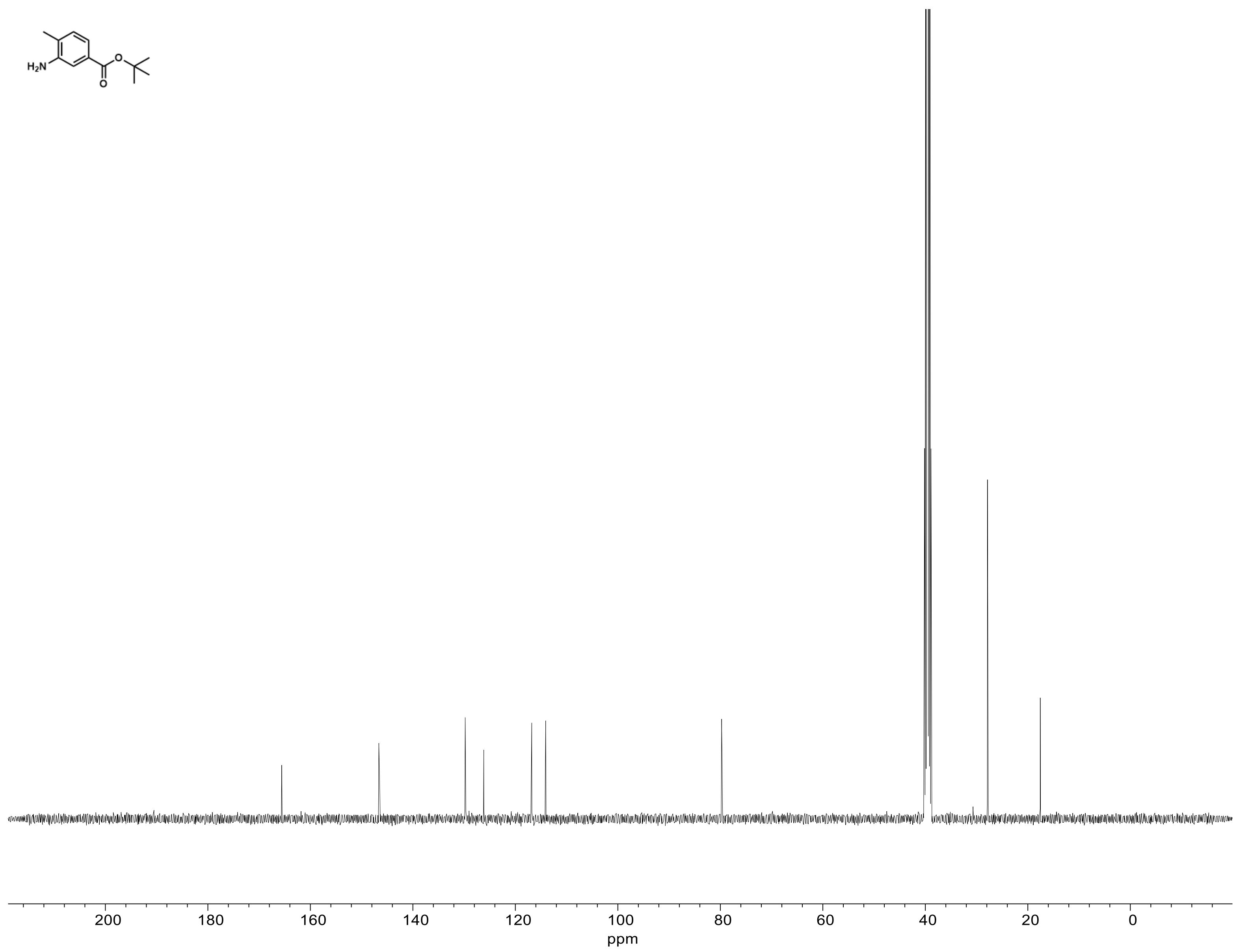

^{13}C NMR (101 MHz, DMSO) of compound **1**.

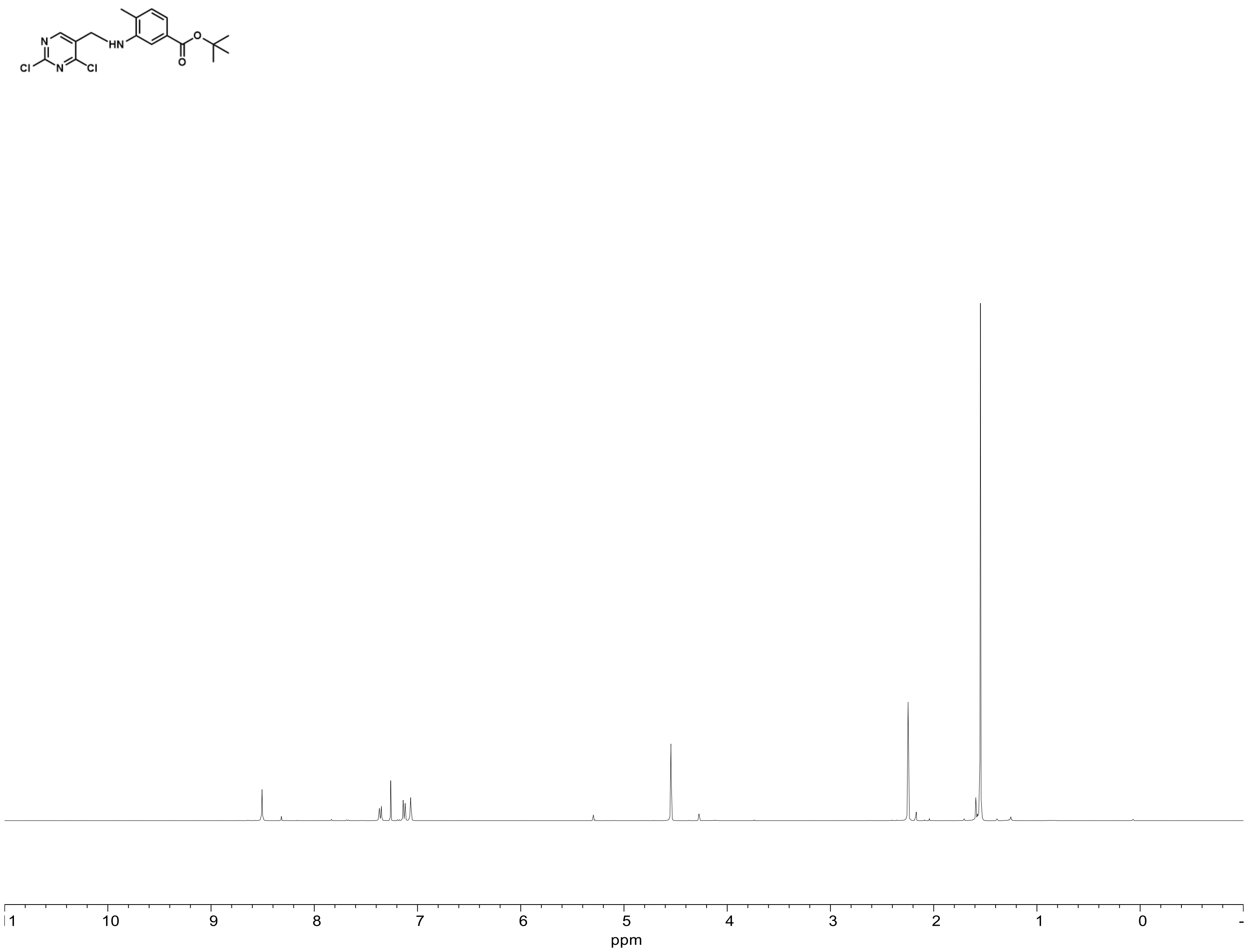

^{1}H NMR (400 MHz, $CDCl_3$) of compound **3**.

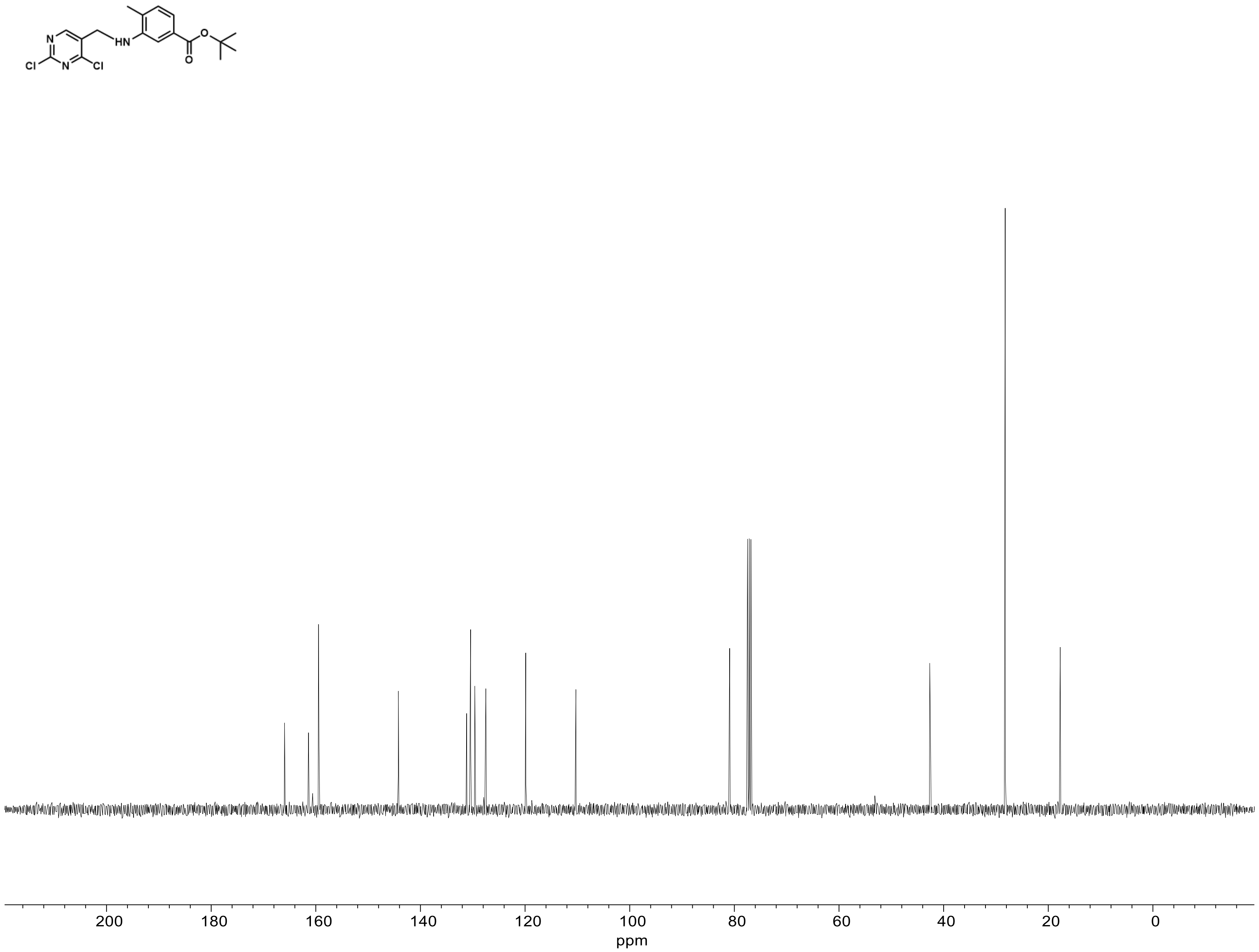

^{13}C NMR (101 MHz, $CDCl_3$) of compound **3**.

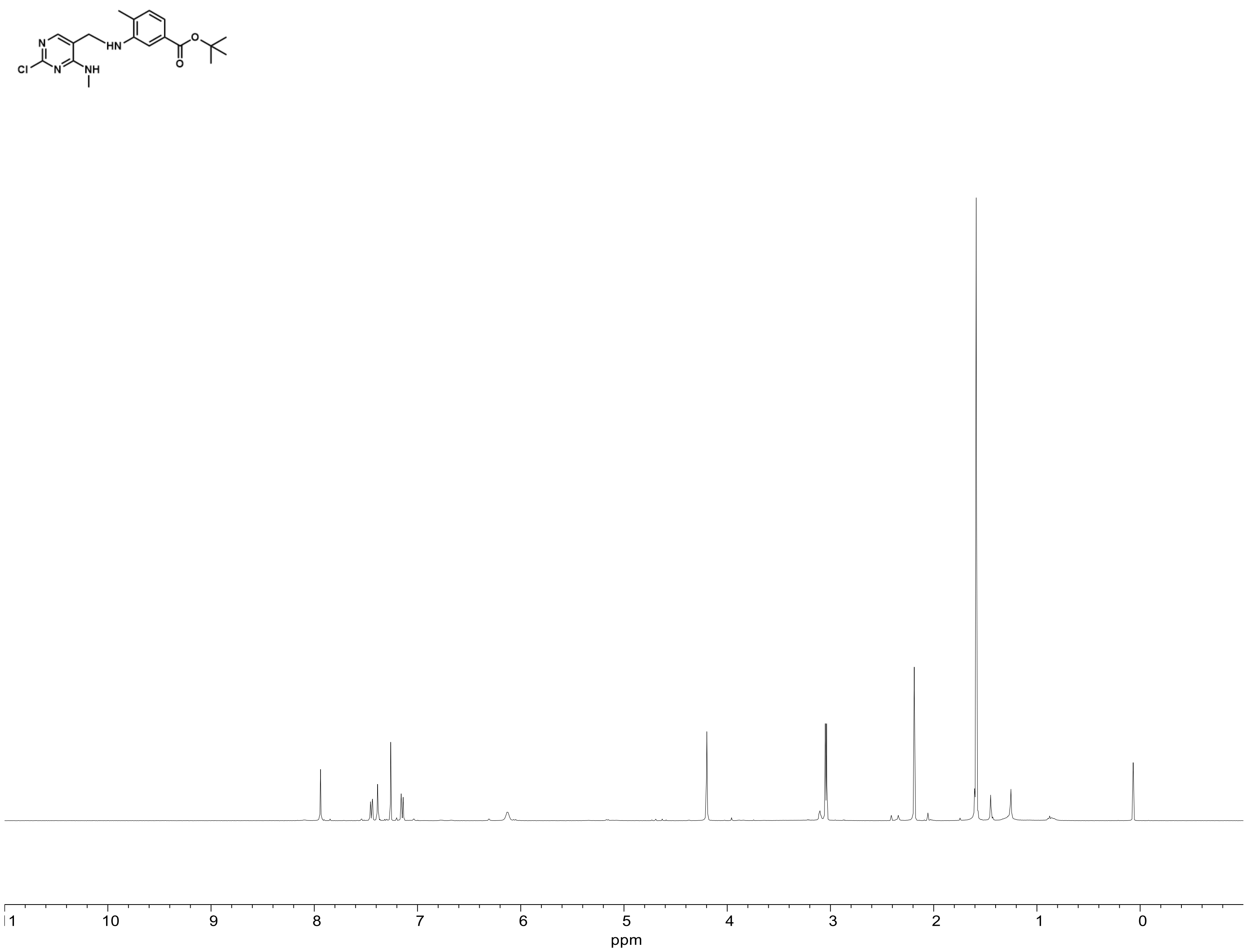

^1H NMR (400 MHz, $CDCl_3$) of compound **SI-2**.

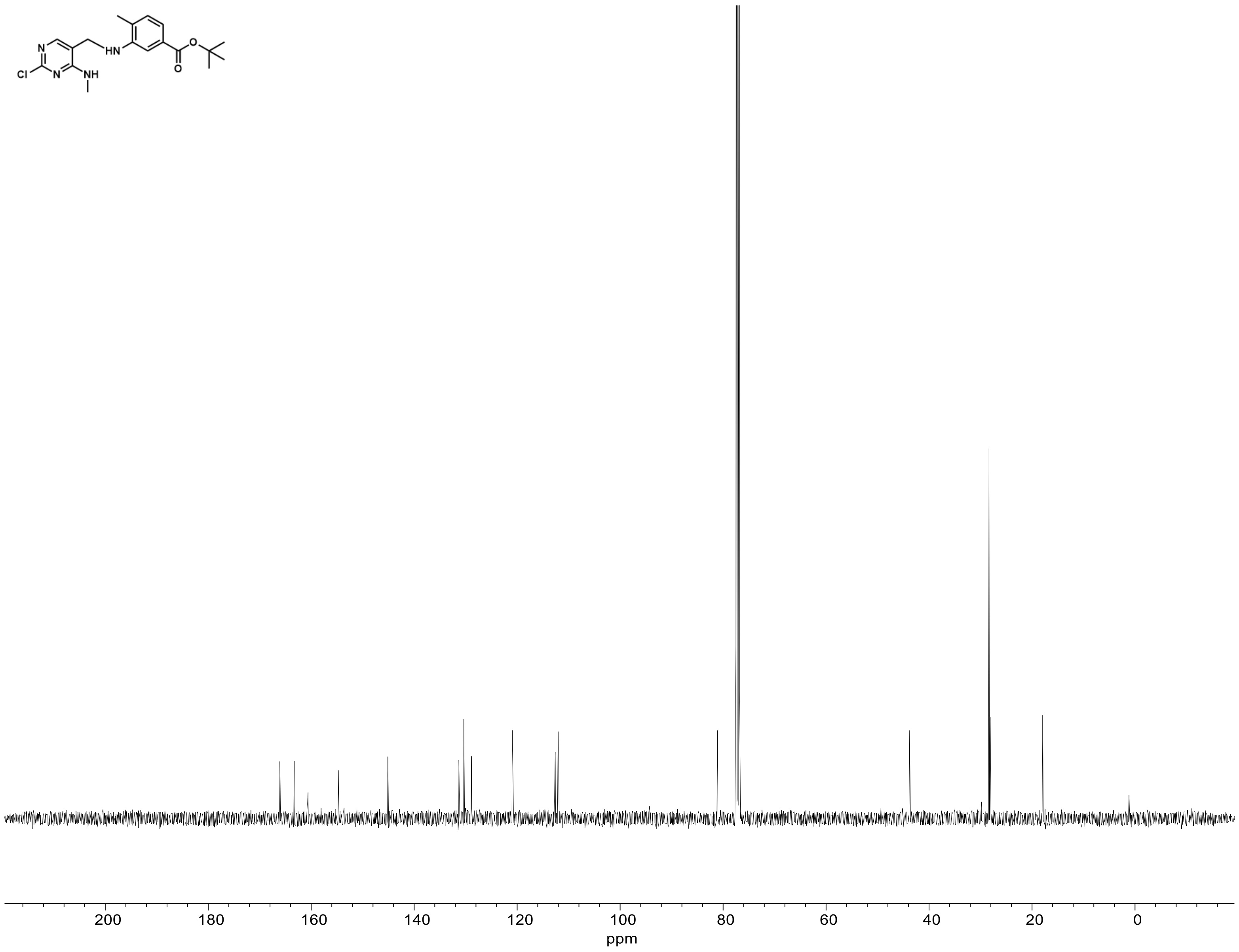

^{13}C NMR (101 MHz, $CDCl_3$) of compound **SI-2**.

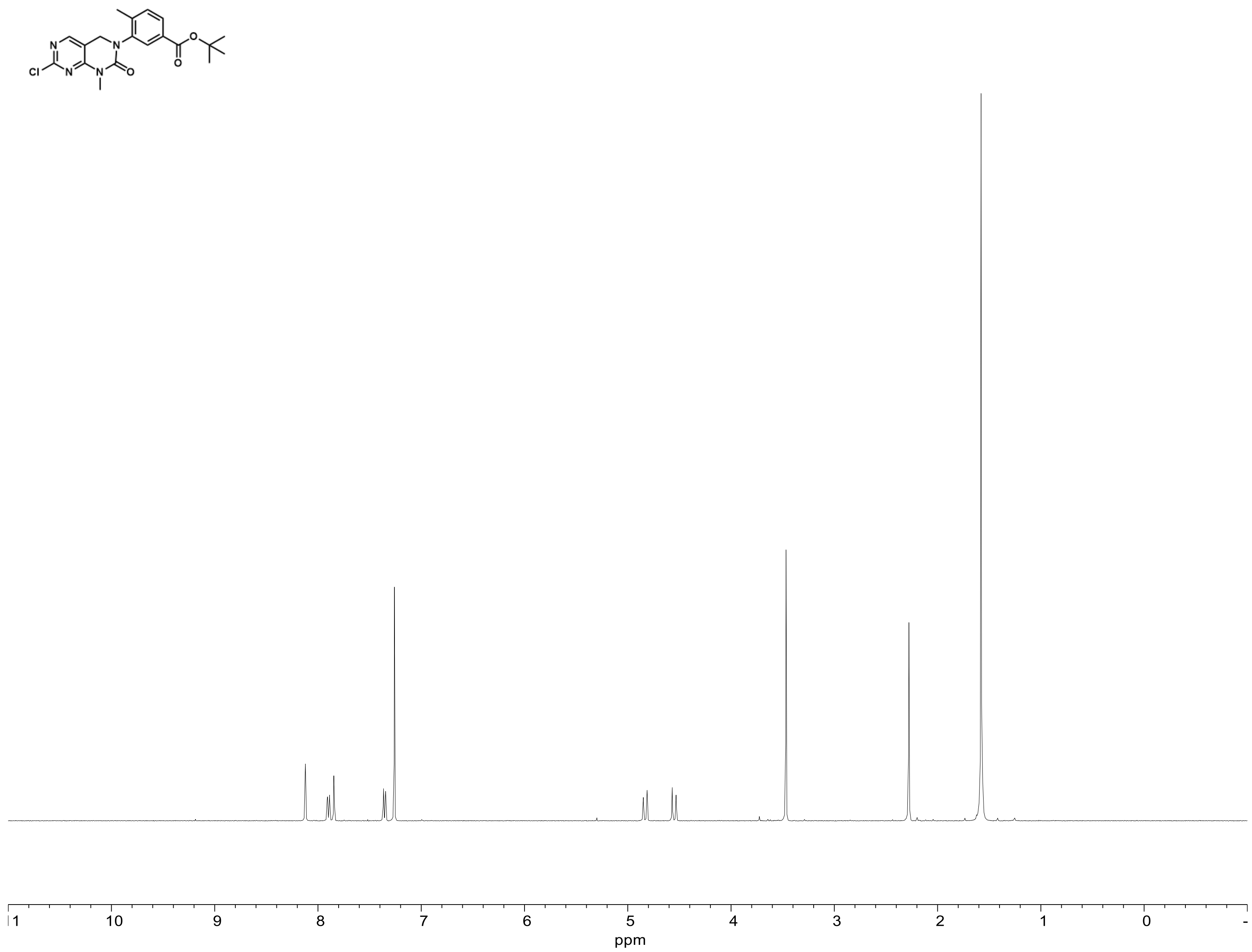

^{1}H NMR (400 MHz, $CDCl_3$) of compound **4**.

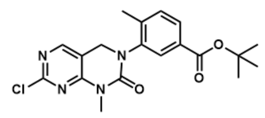

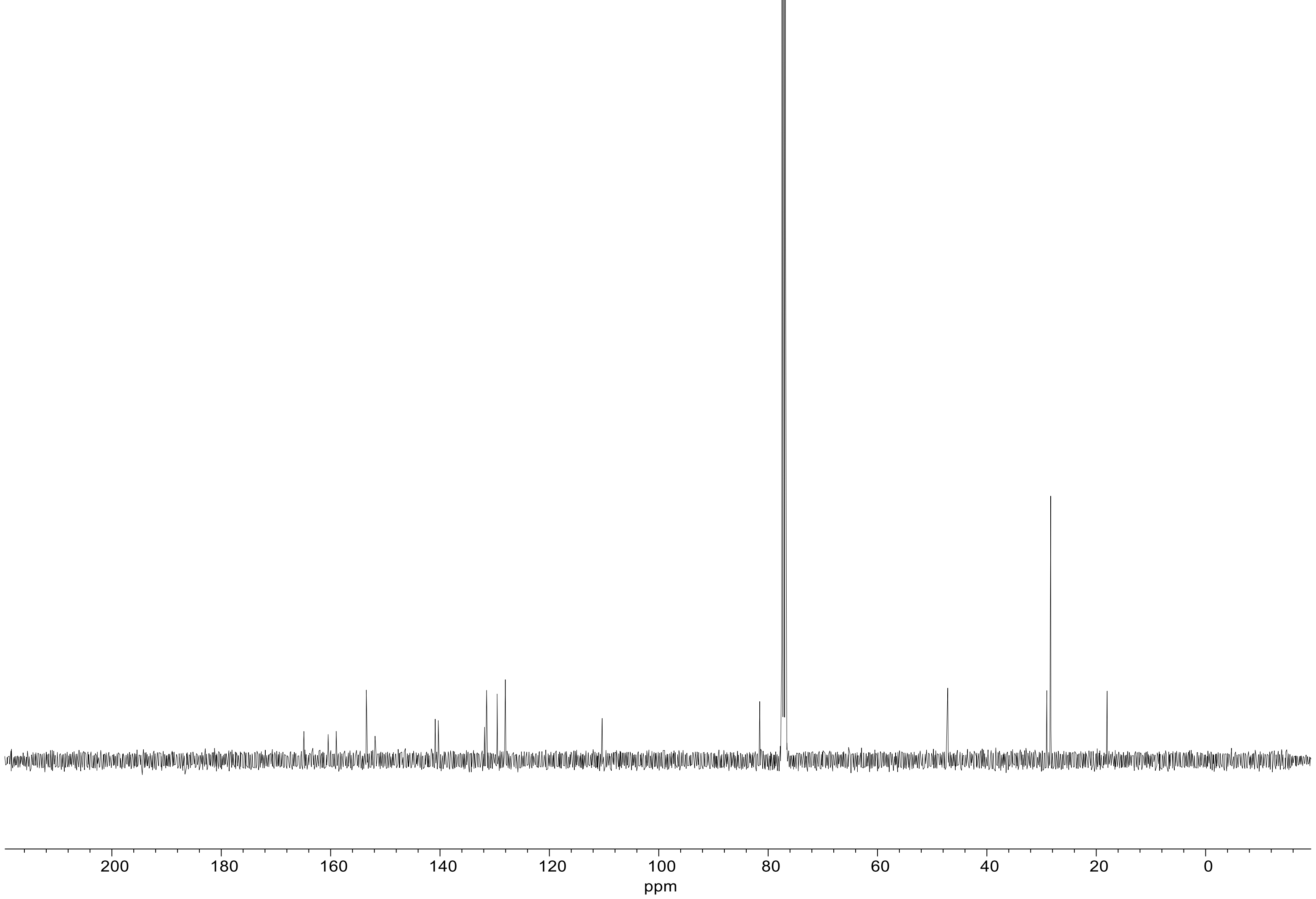

^{13}C NMR (101 MHz, $CDCl_3$) of compound **4**.

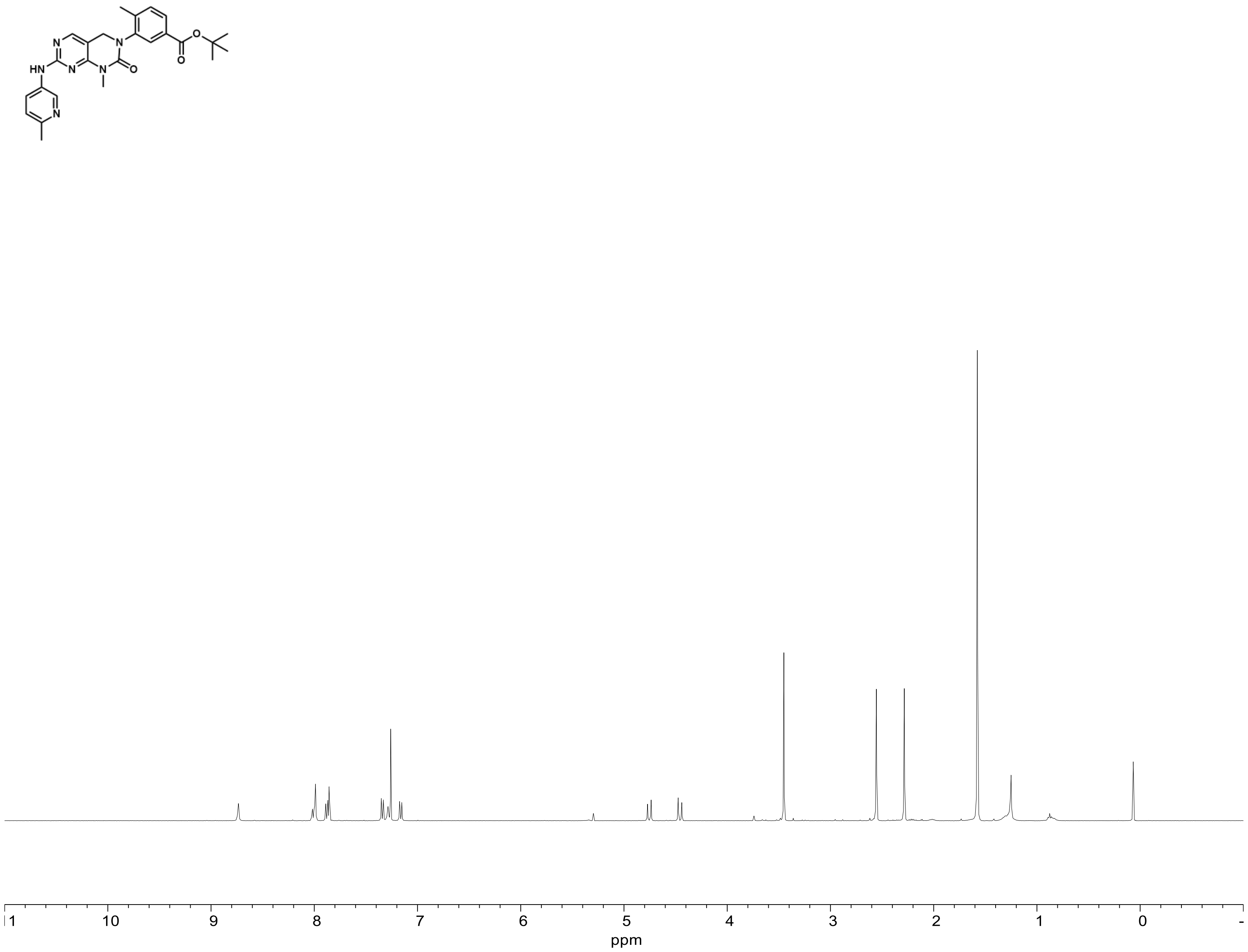

^{1}H NMR (400 MHz, $CDCl_3$) of compound **SI-3**.

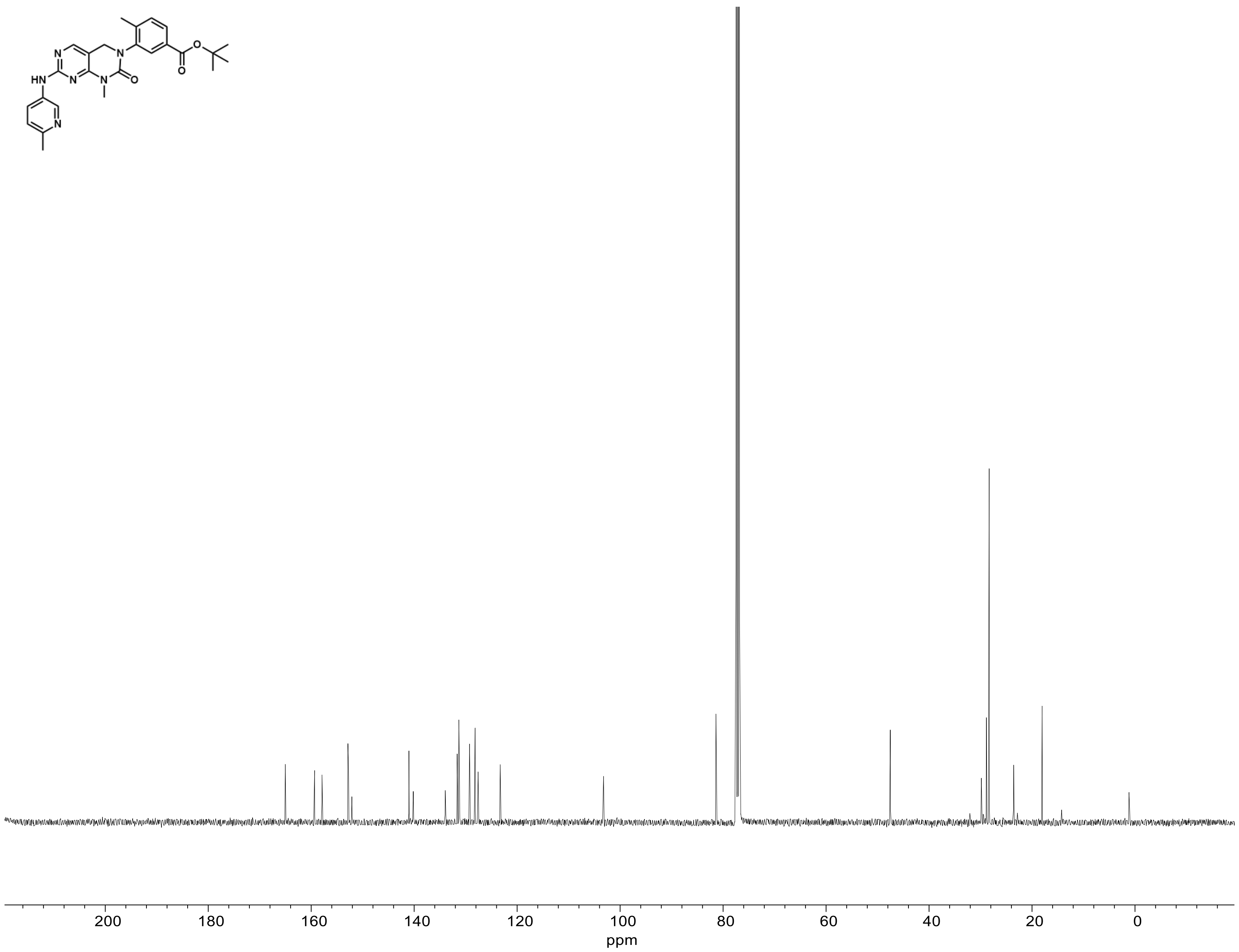

^{13}C NMR (101 MHz, $CDCl_3$) of compound **SI-3**.

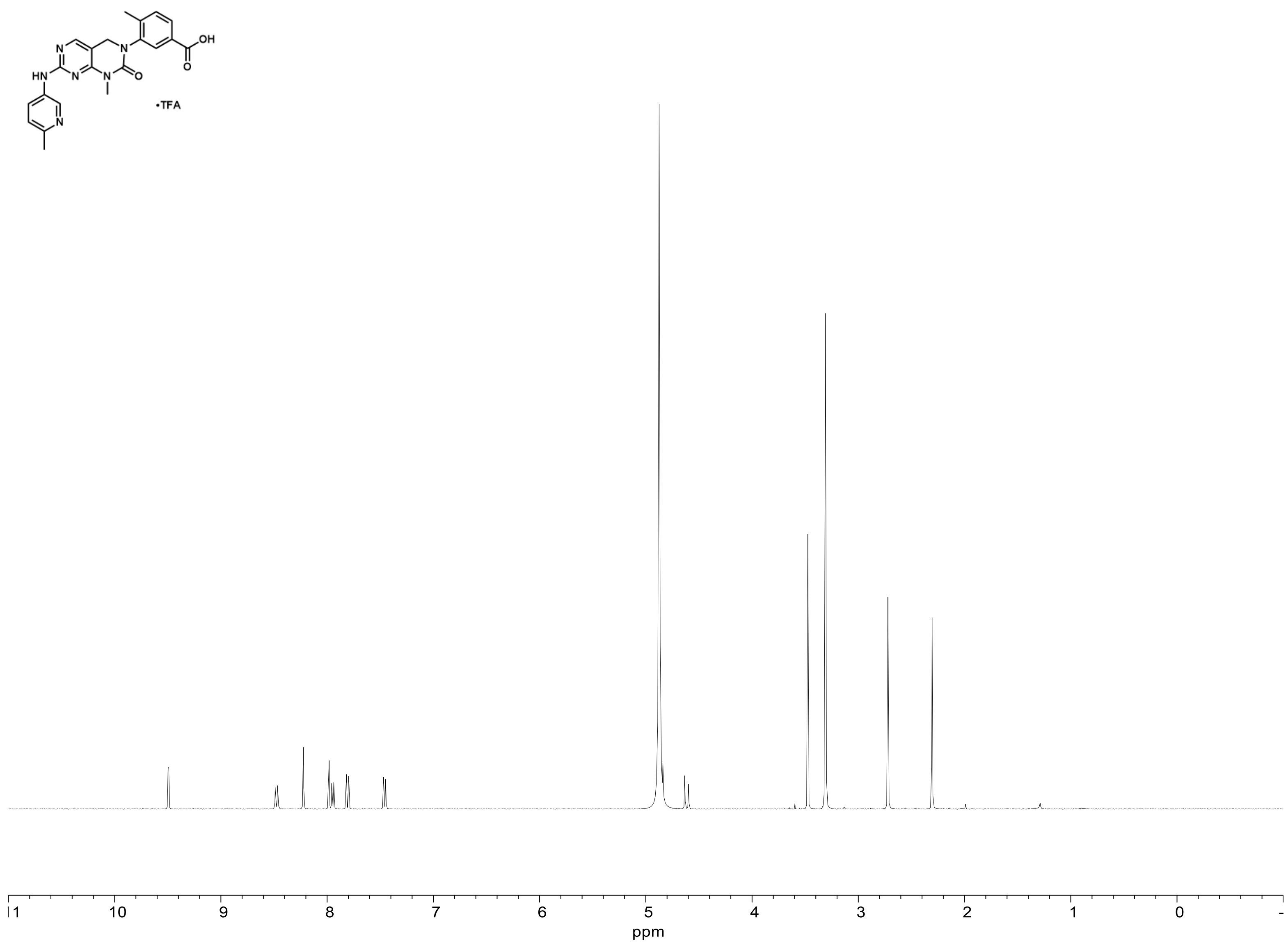

^{1}H NMR (400 MHz, MeOD) of compound **5**.

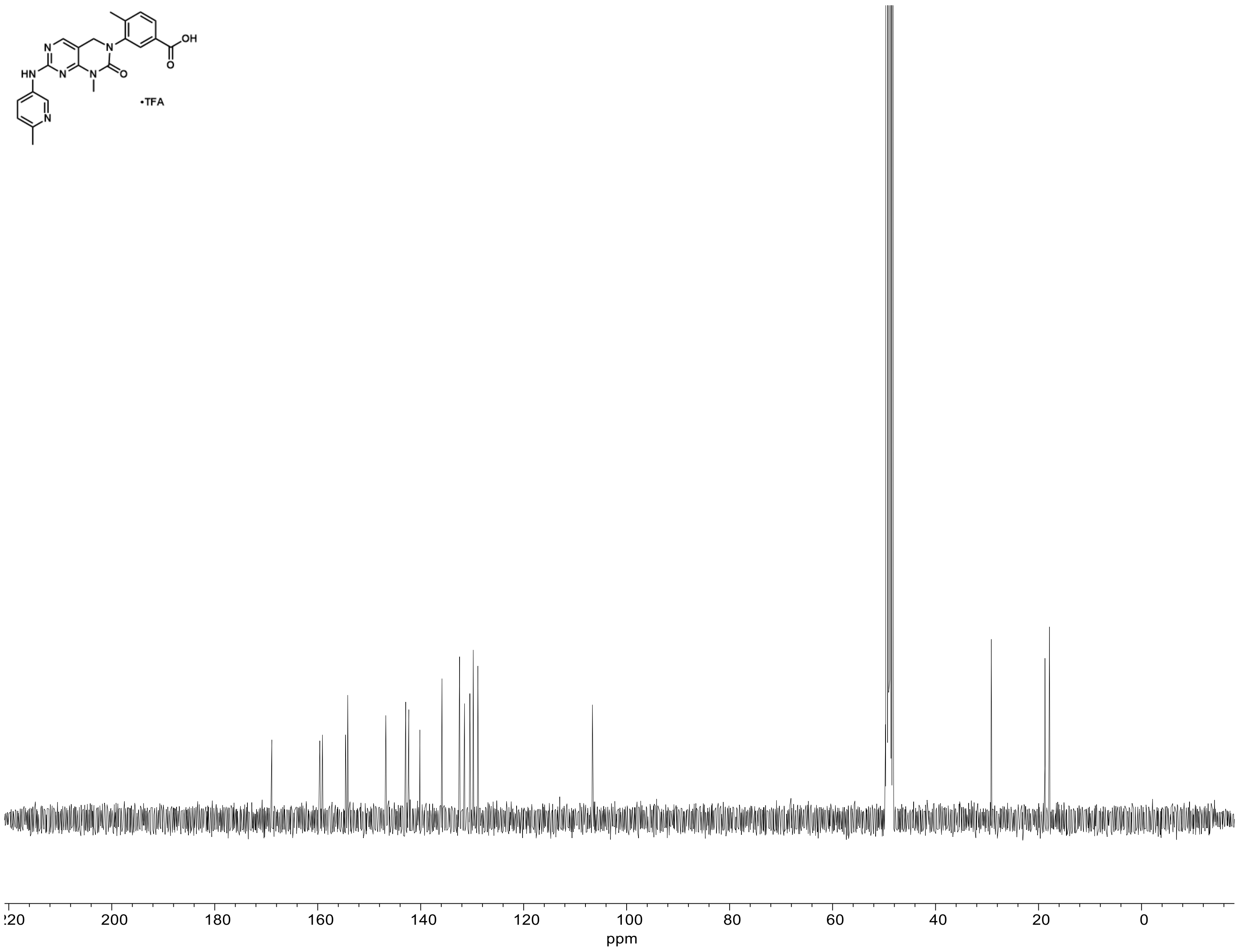

^{13}C NMR (101 MHz, MeOD) of compound **5**.

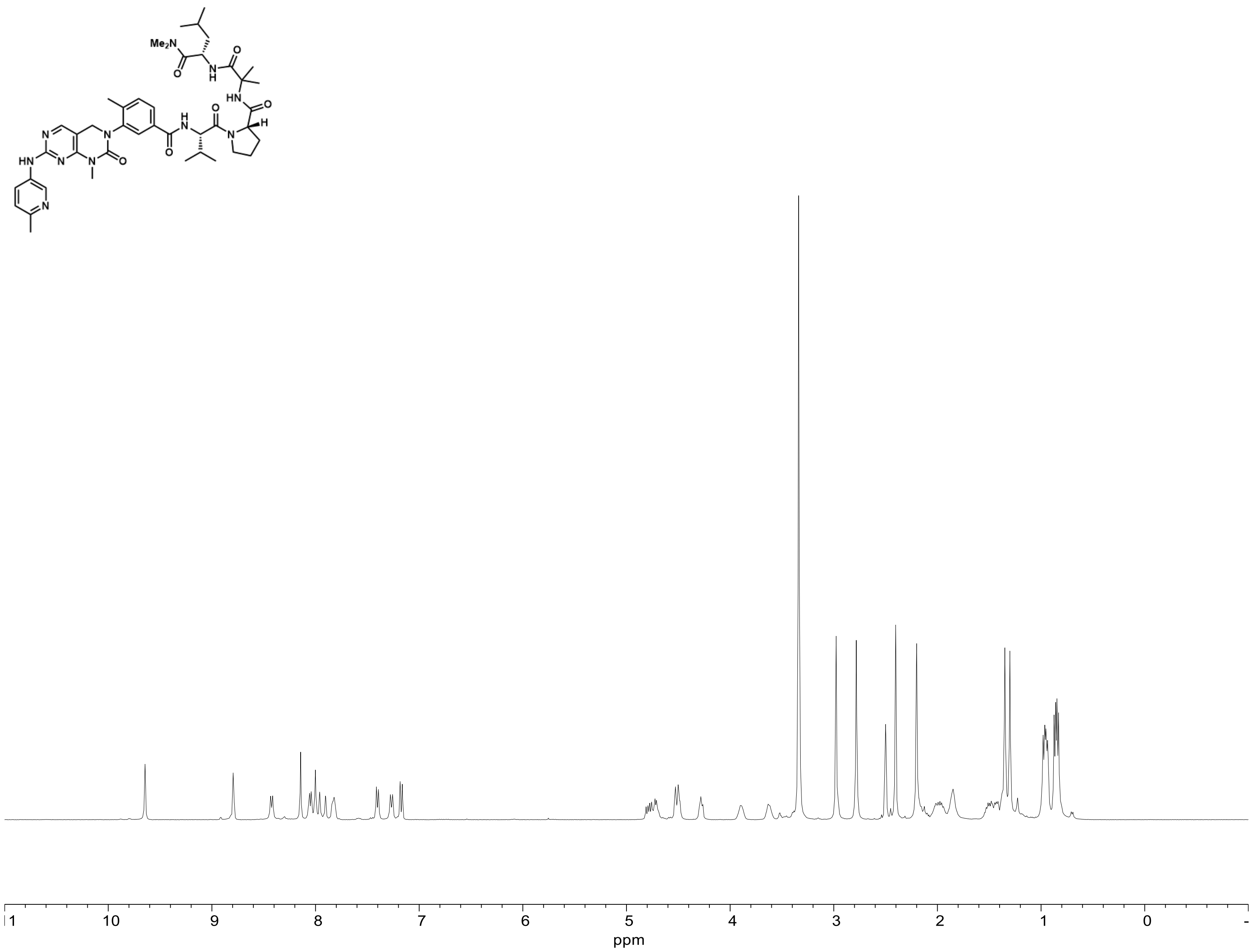

^{1}H NMR (400 MHz, DMSO) of compound **7a**.

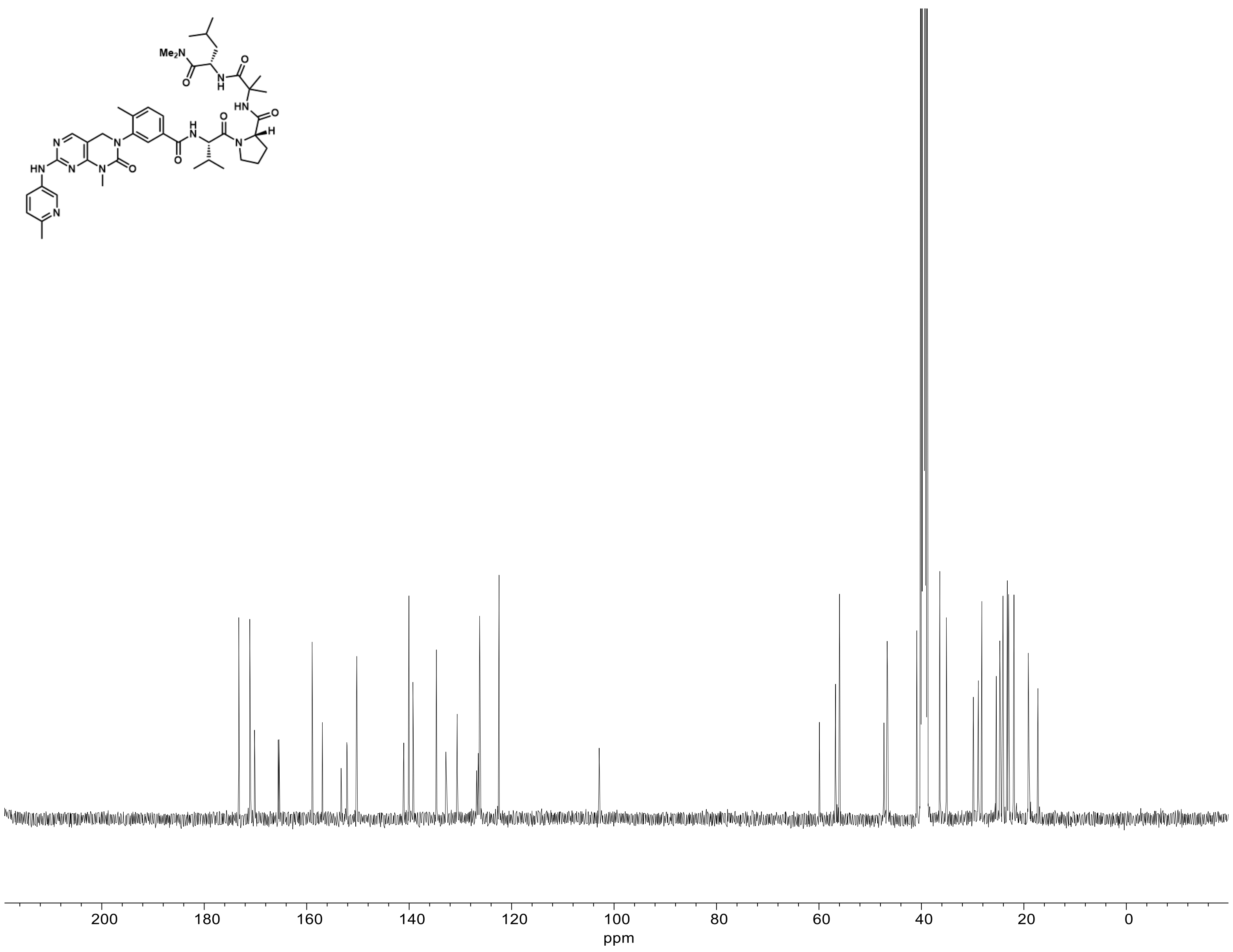

^{13}C NMR (101 MHz, DMSO) of compound **7a**.

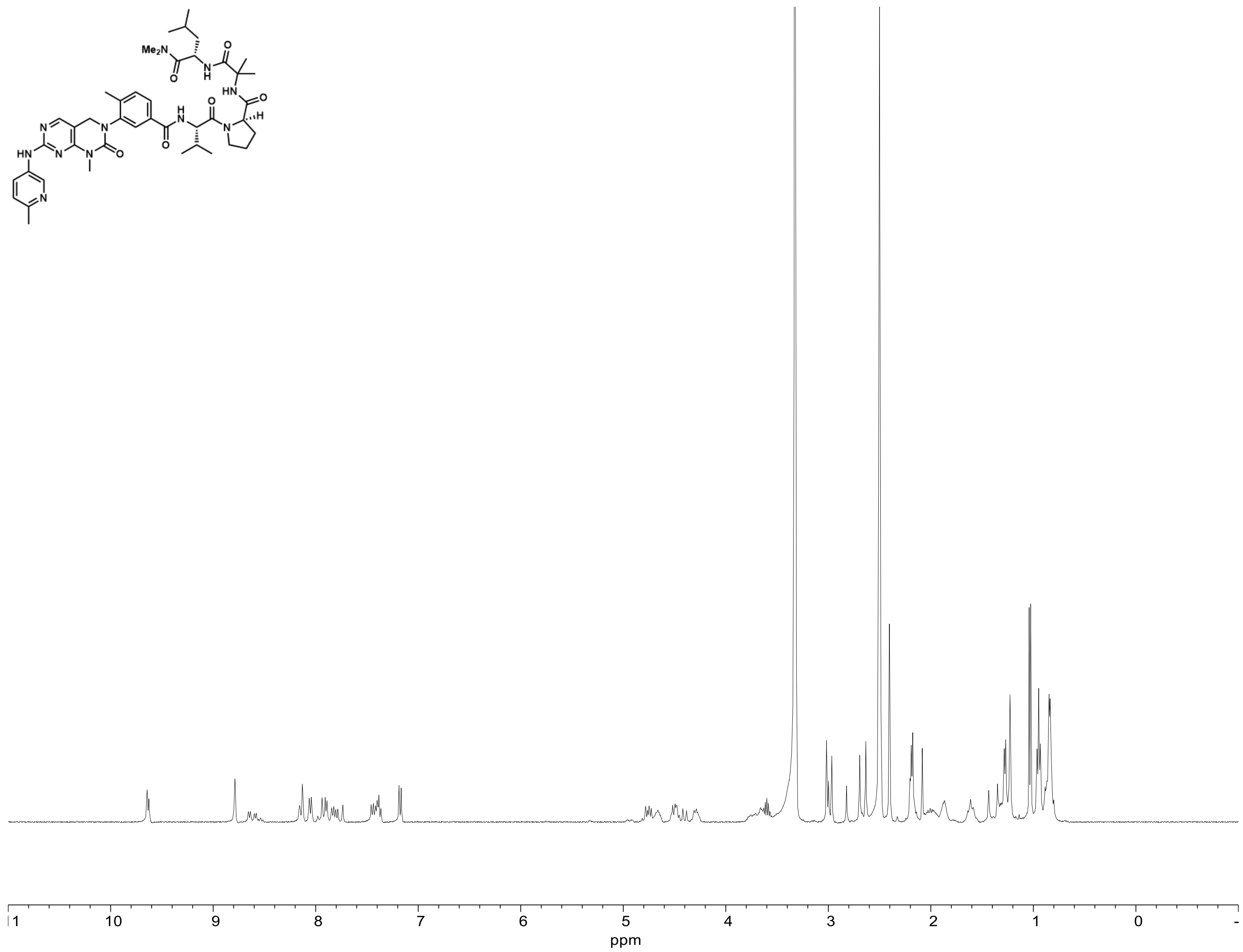

^{1}H NMR (400 MHz, DMSO) of compound **7b**.

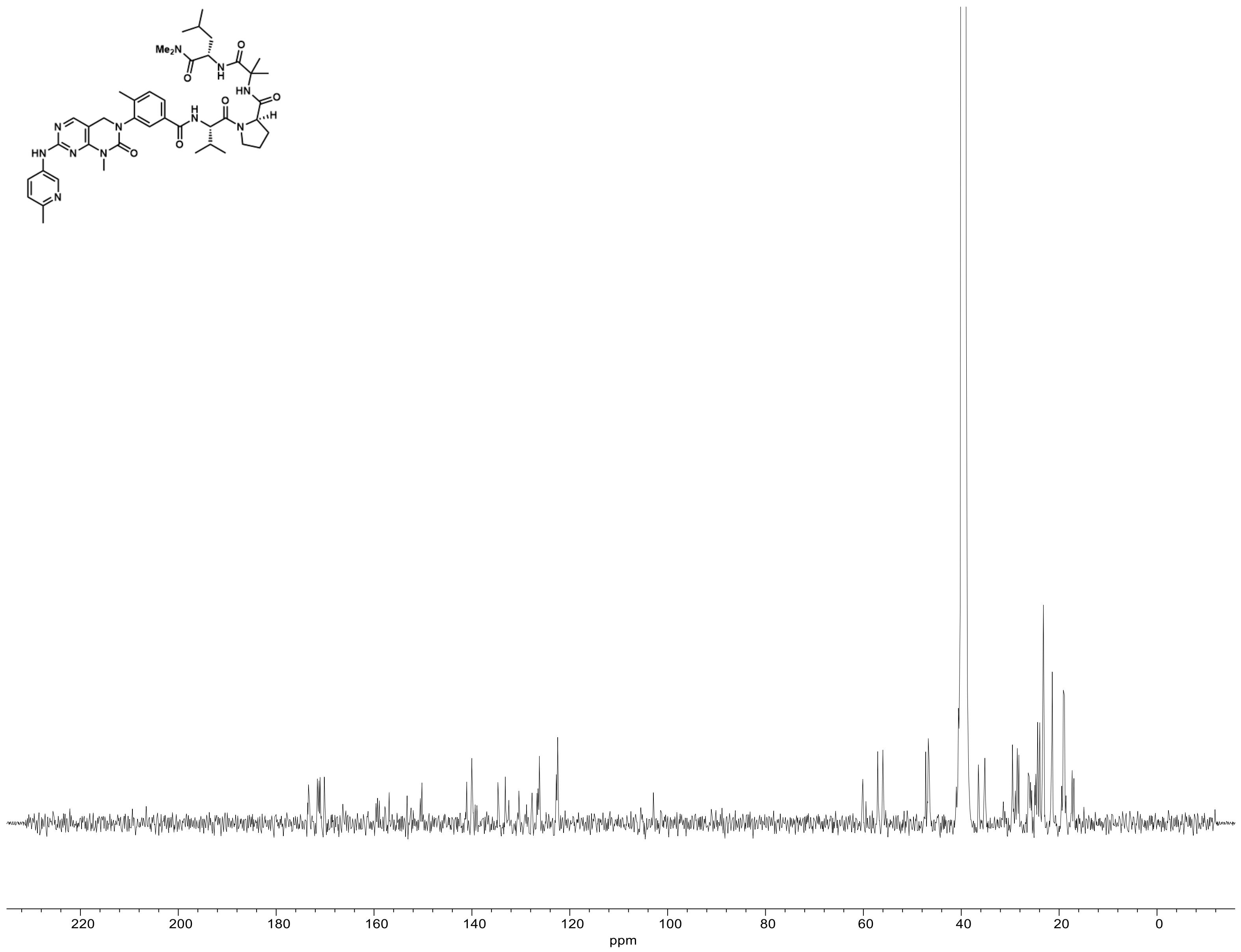

^{13}C NMR (400 MHz, DMSO) of compound **7b**.

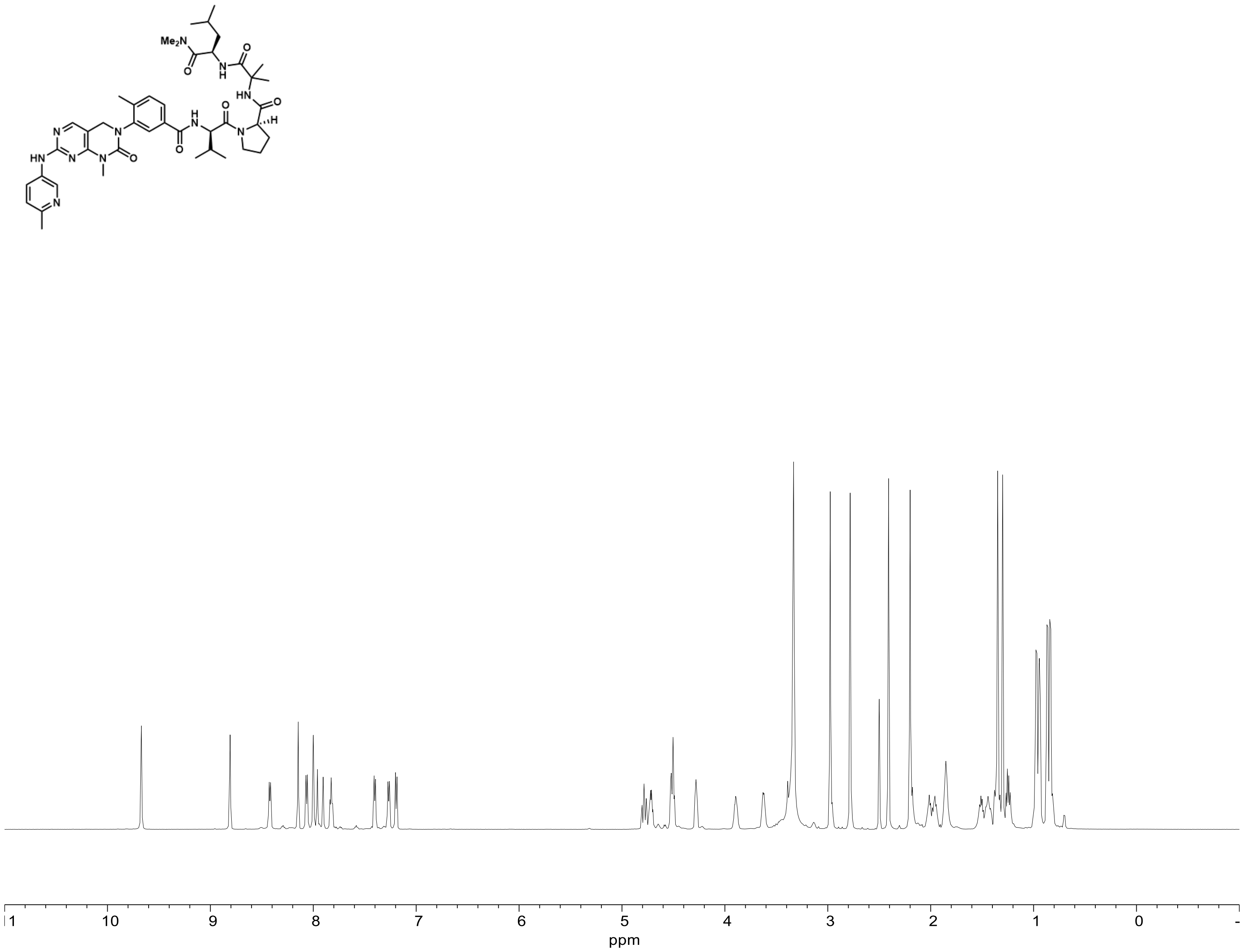

^{1}H NMR (400 MHz, DMSO) of compound **7c**.

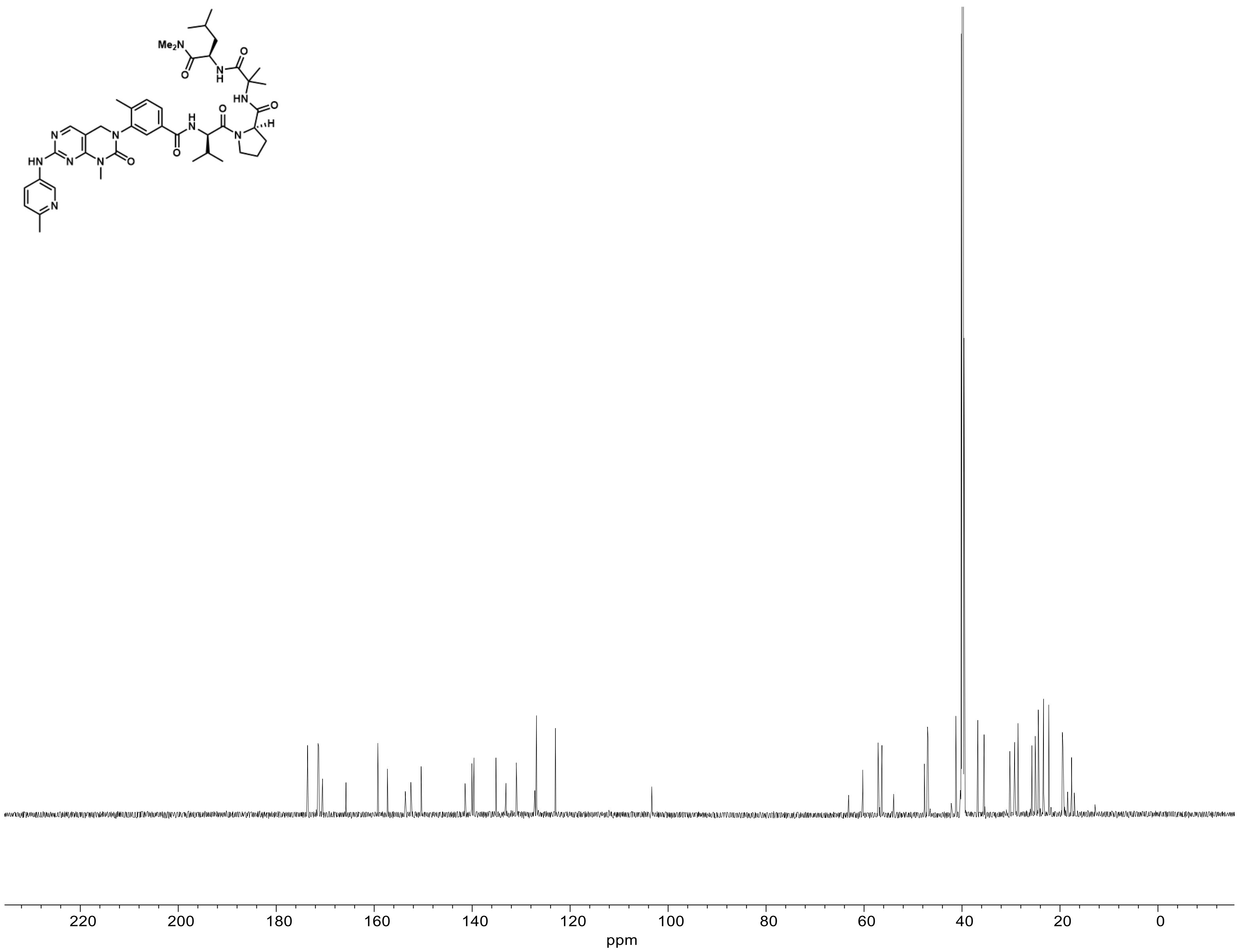

^{13}C NMR (400 MHz, DMSO) of compound **7c**.

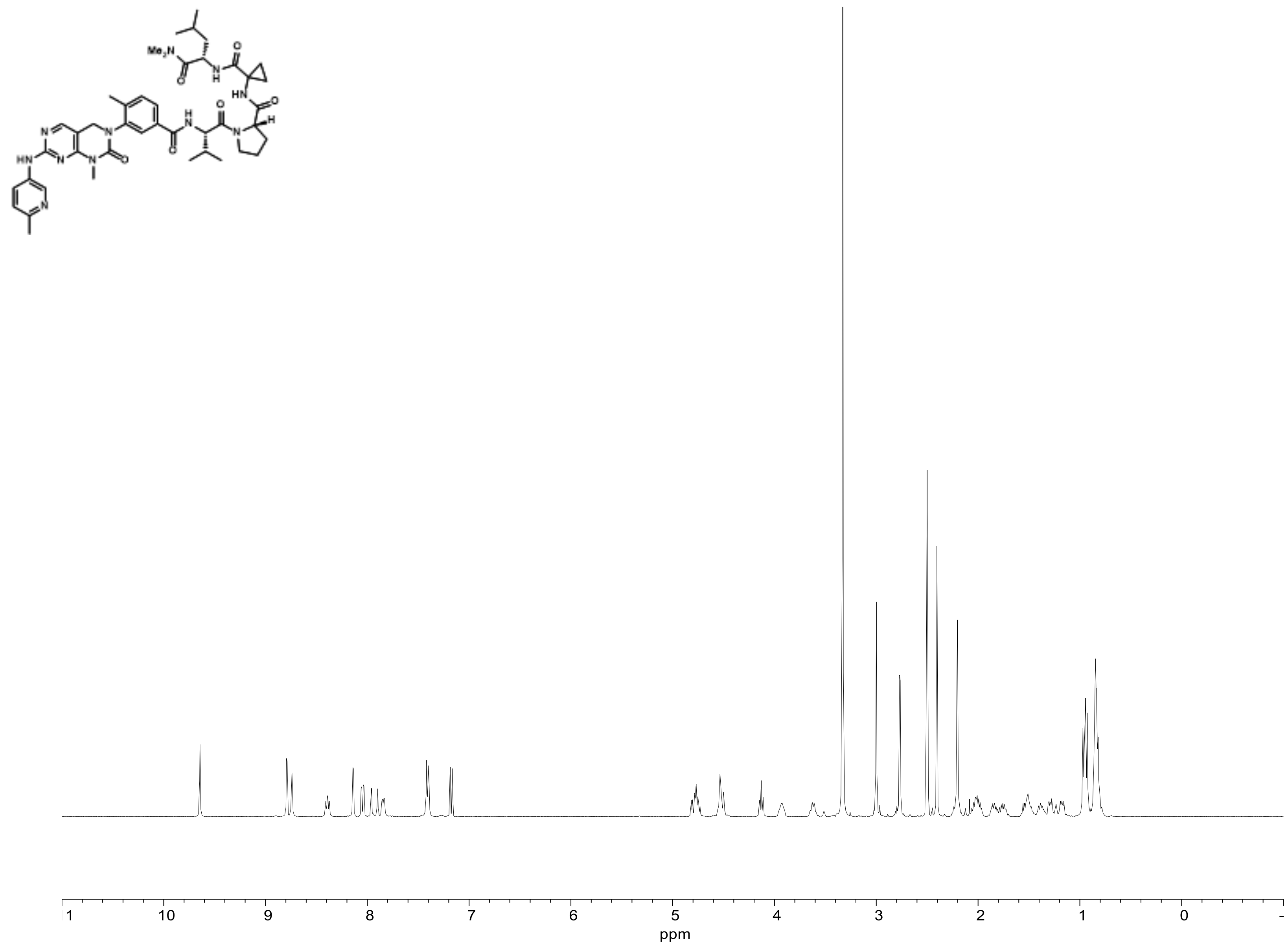

^{1}H NMR (400 MHz, DMSO) of compound **7d**.

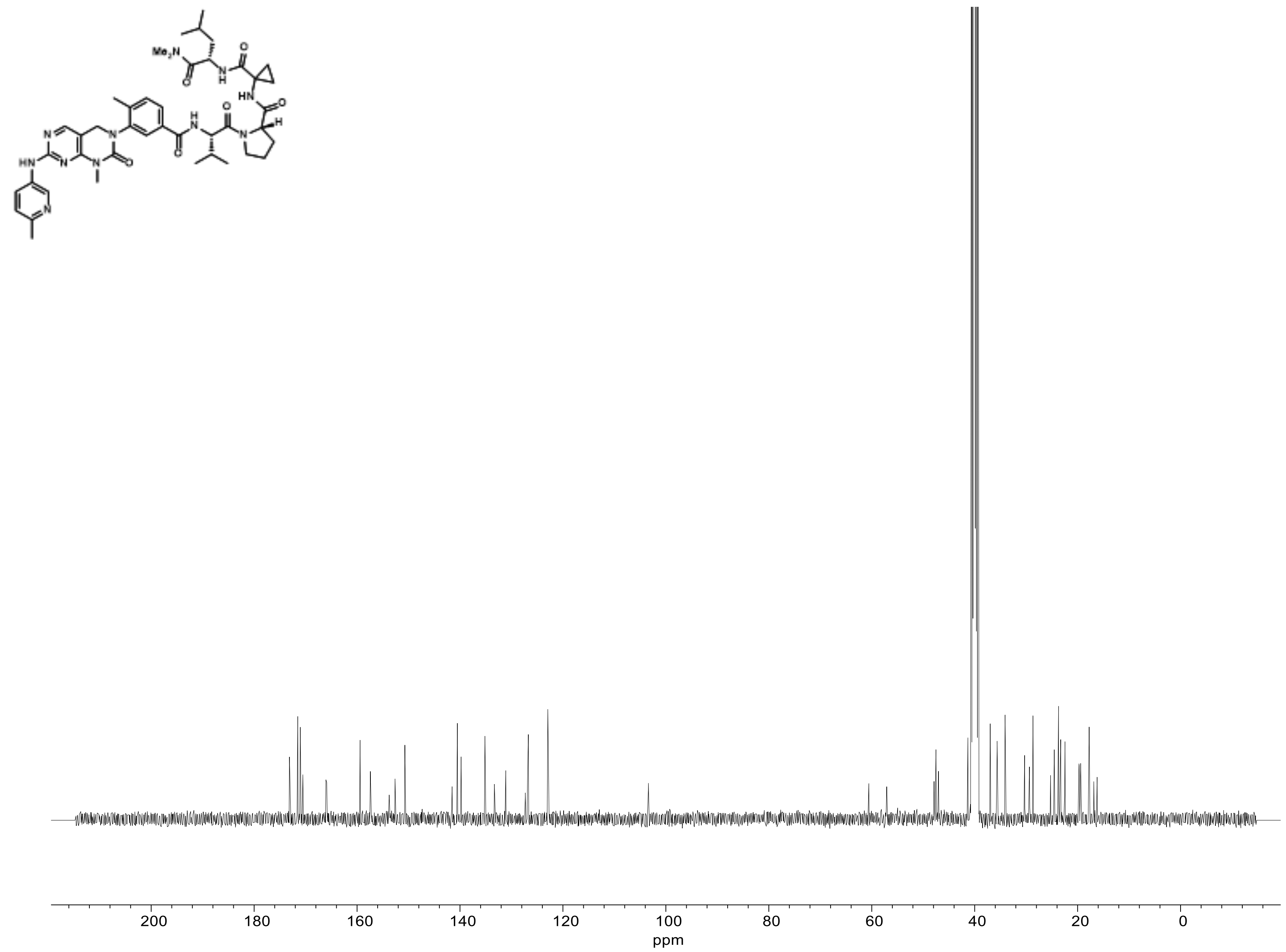

^{13}C NMR (400 MHz, DMSO) of compound **7d**.

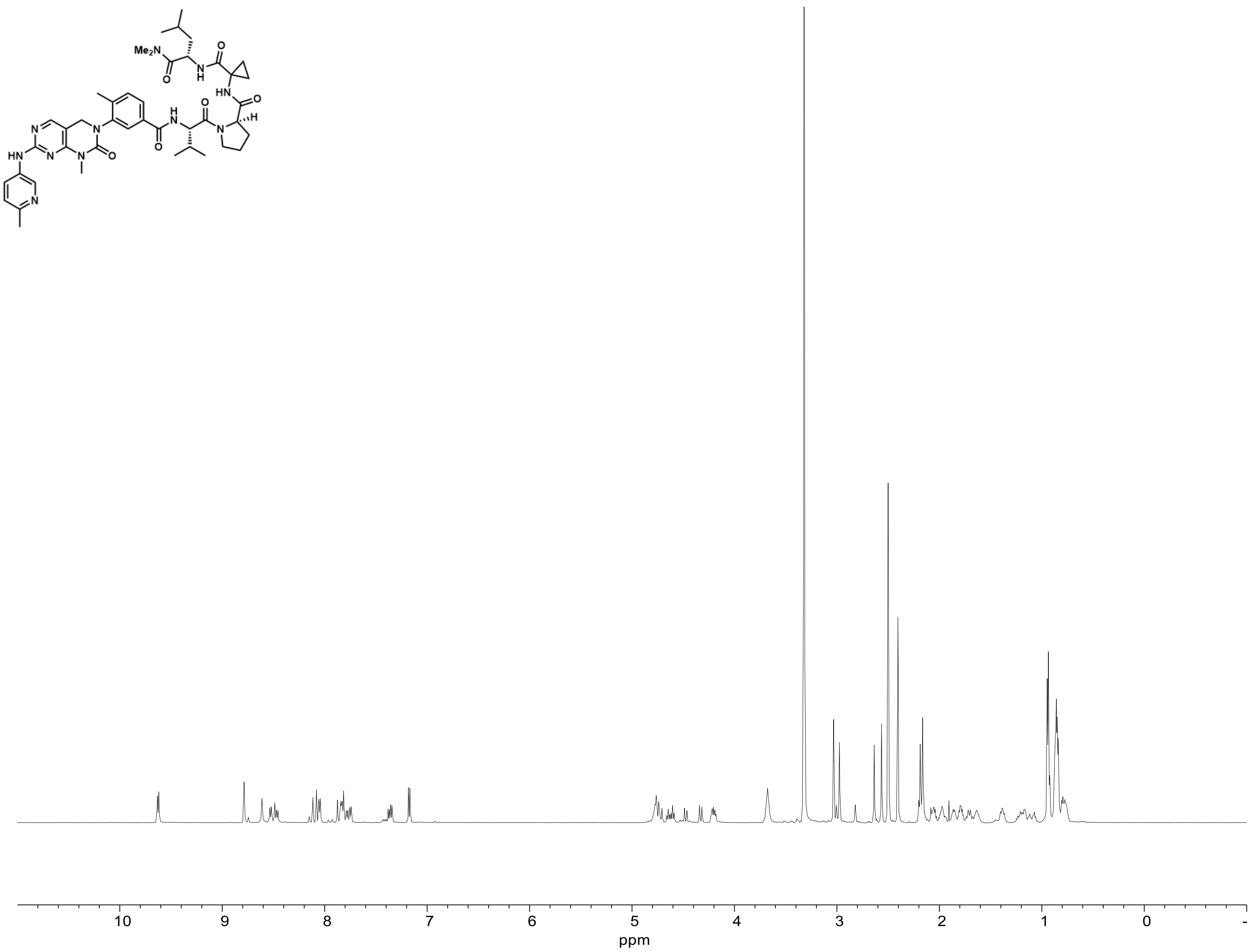

^{1}H NMR (400 MHz, DMSO) of compound **7e**.

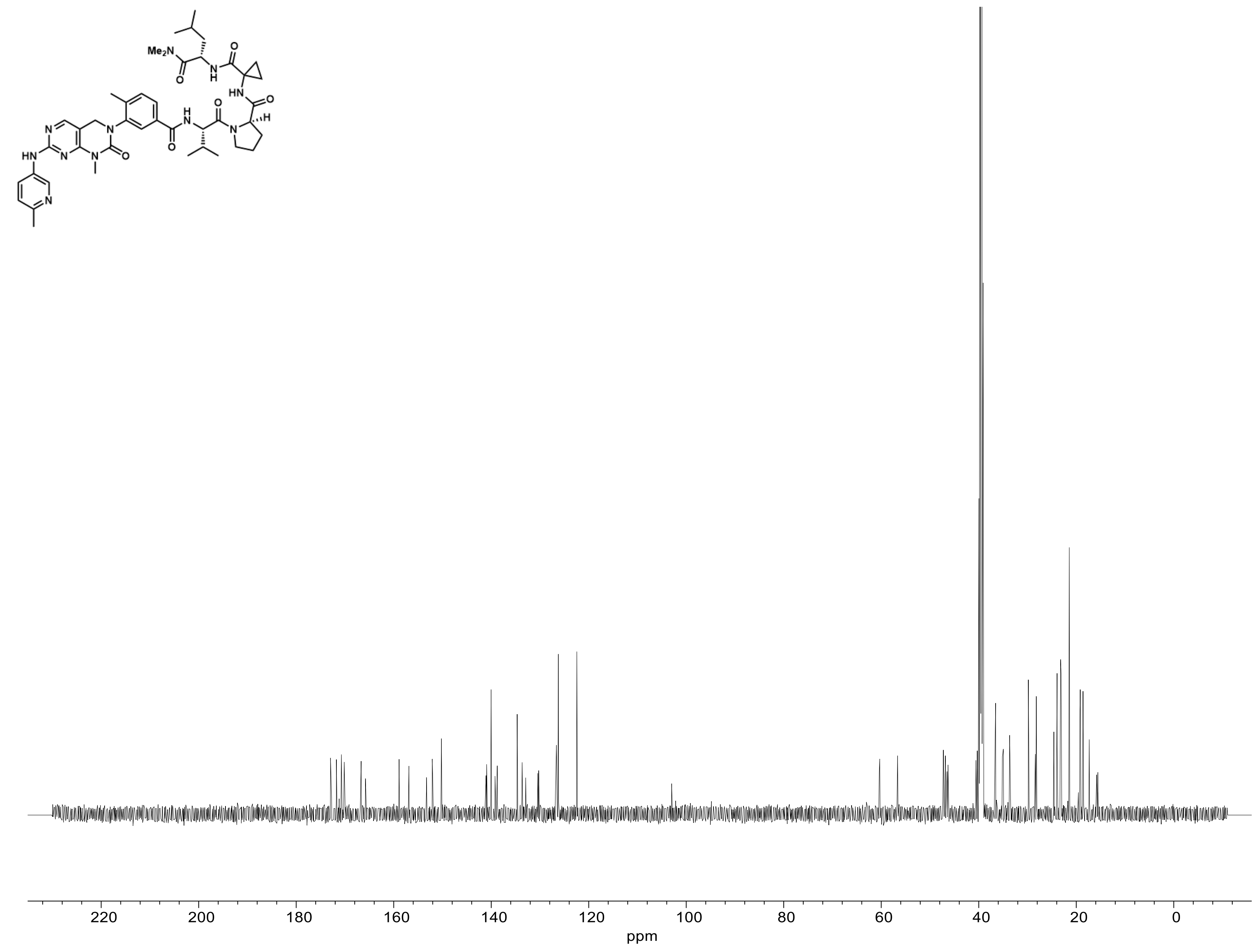

^{13}C NMR (400 MHz, DMSO) of compound **7e**.

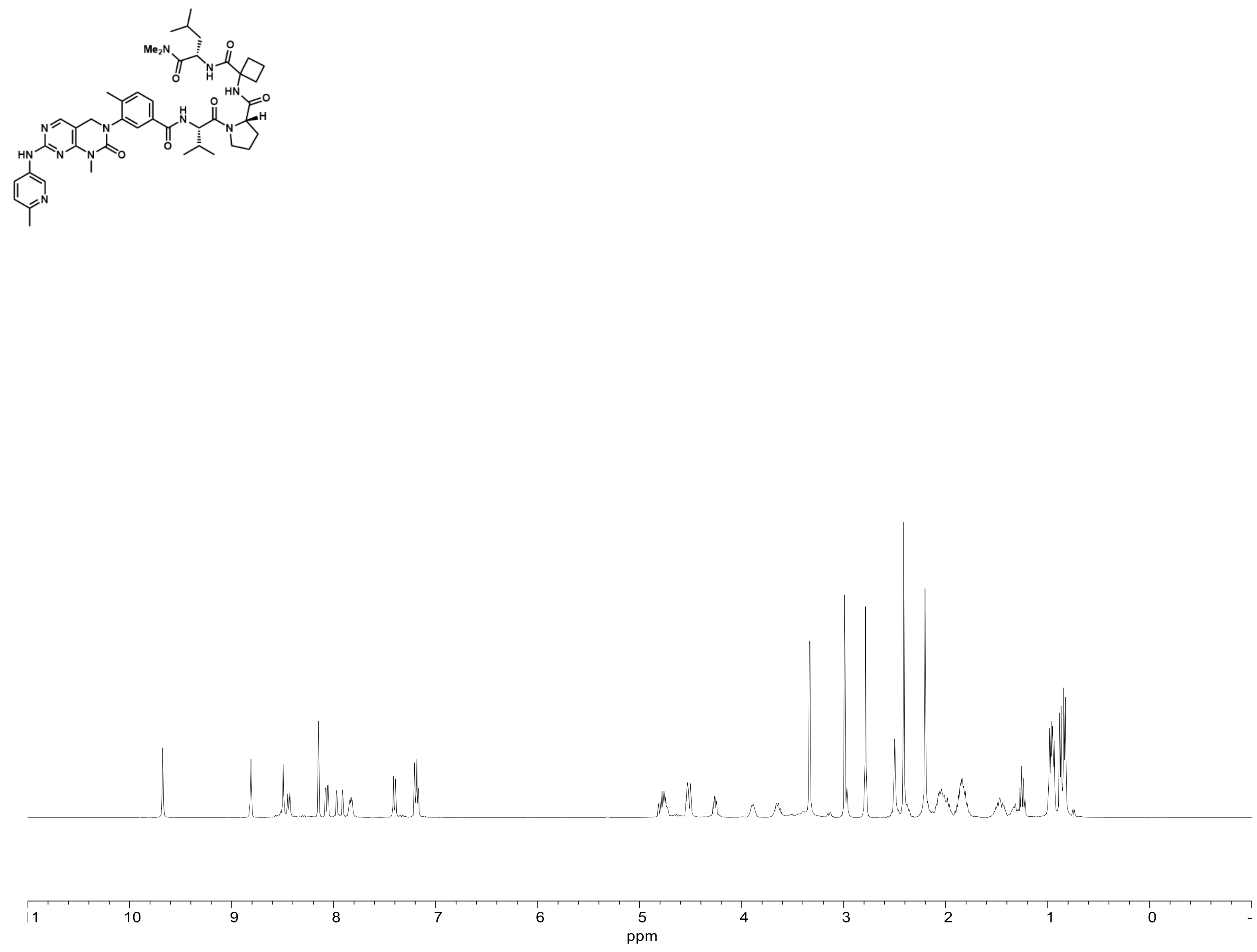

^{1}H NMR (400 MHz, DMSO) of compound **7f**.

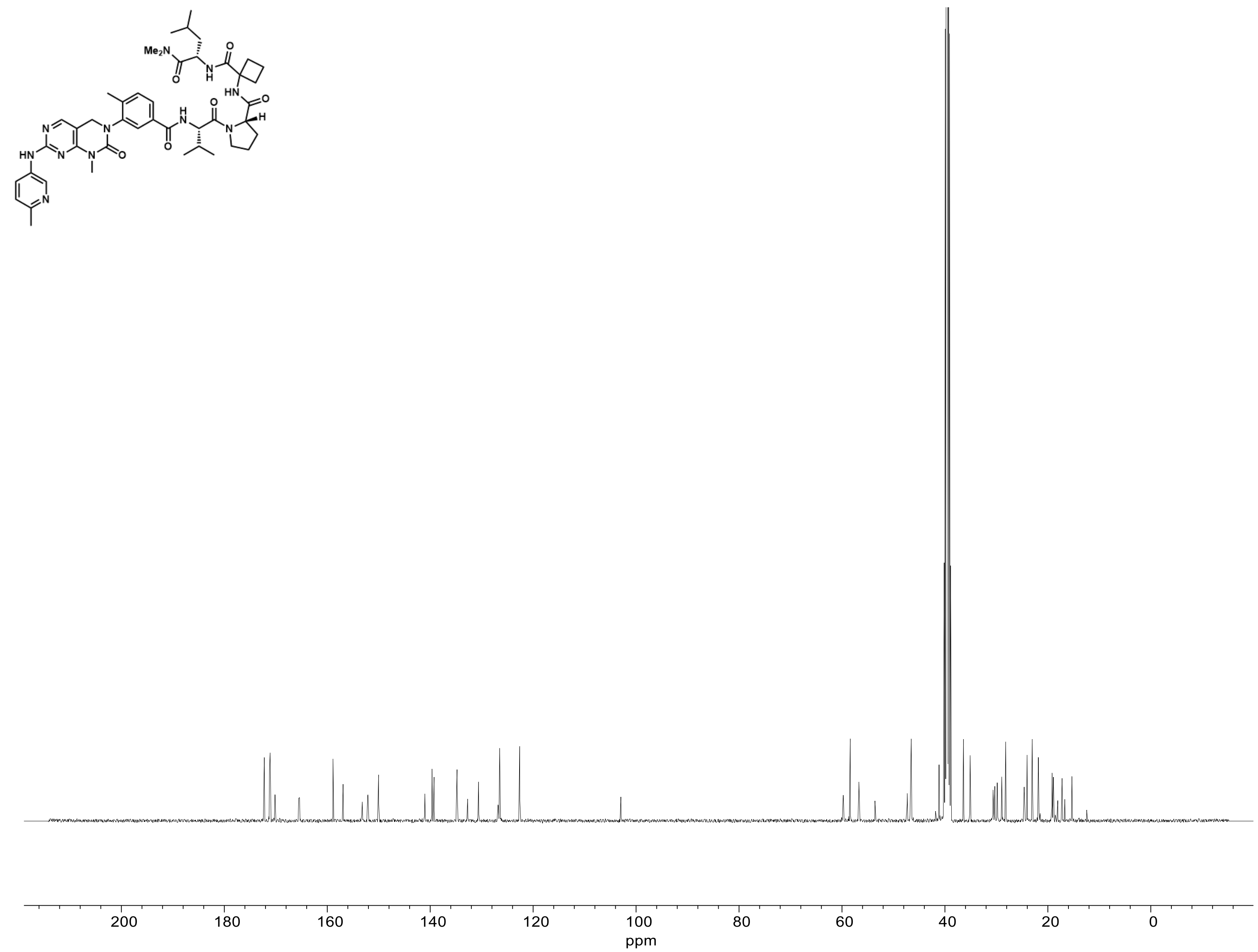

^{13}C NMR (400 MHz, DMSO) of compound **7f**.

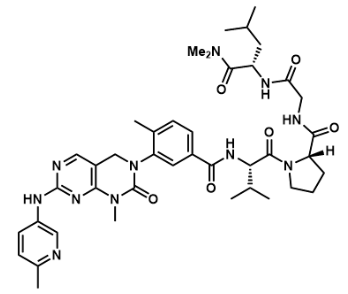

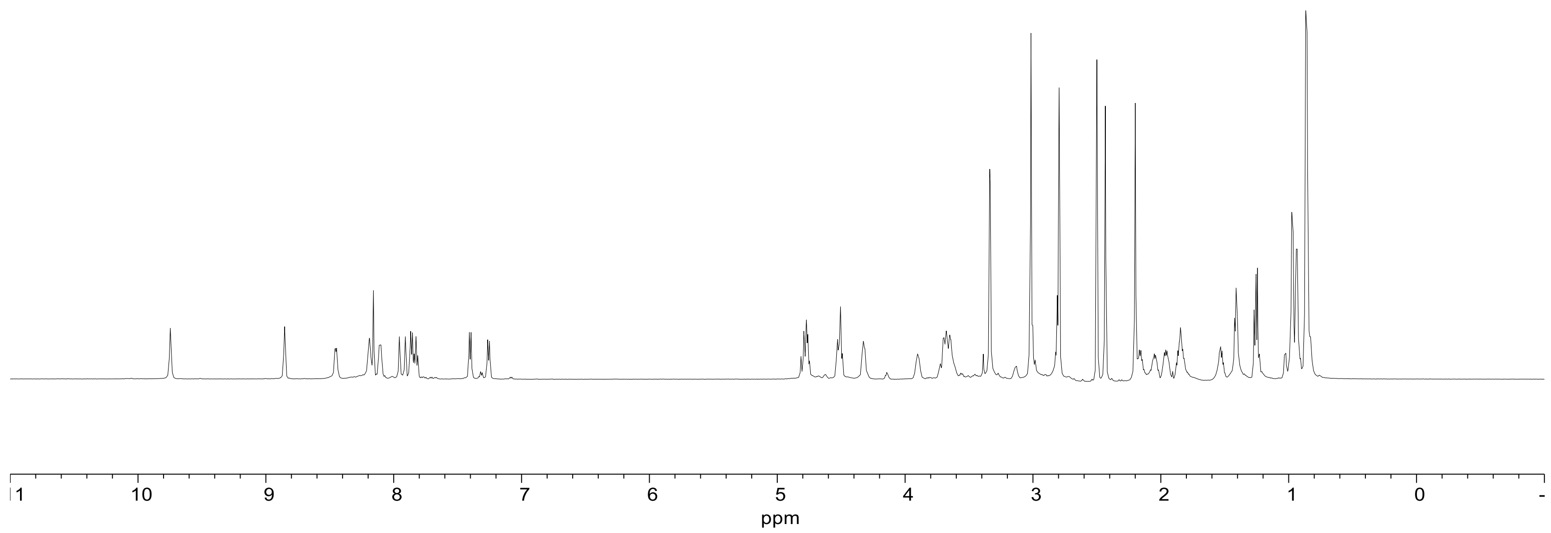

^{1}H NMR (400 MHz, DMSO) of compound **7g**.

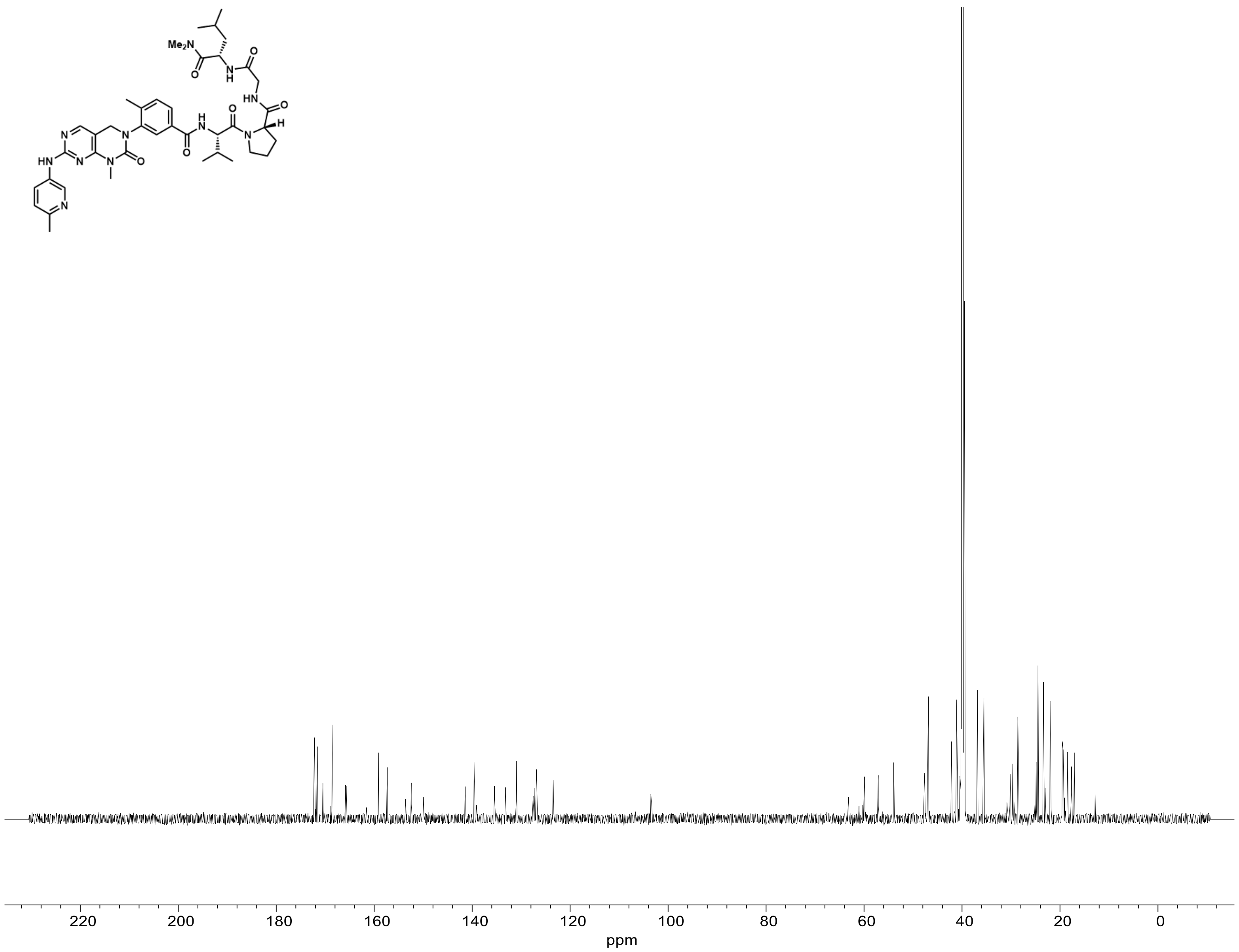

^{13}C NMR (400 MHz, DMSO) of compound **7g**.

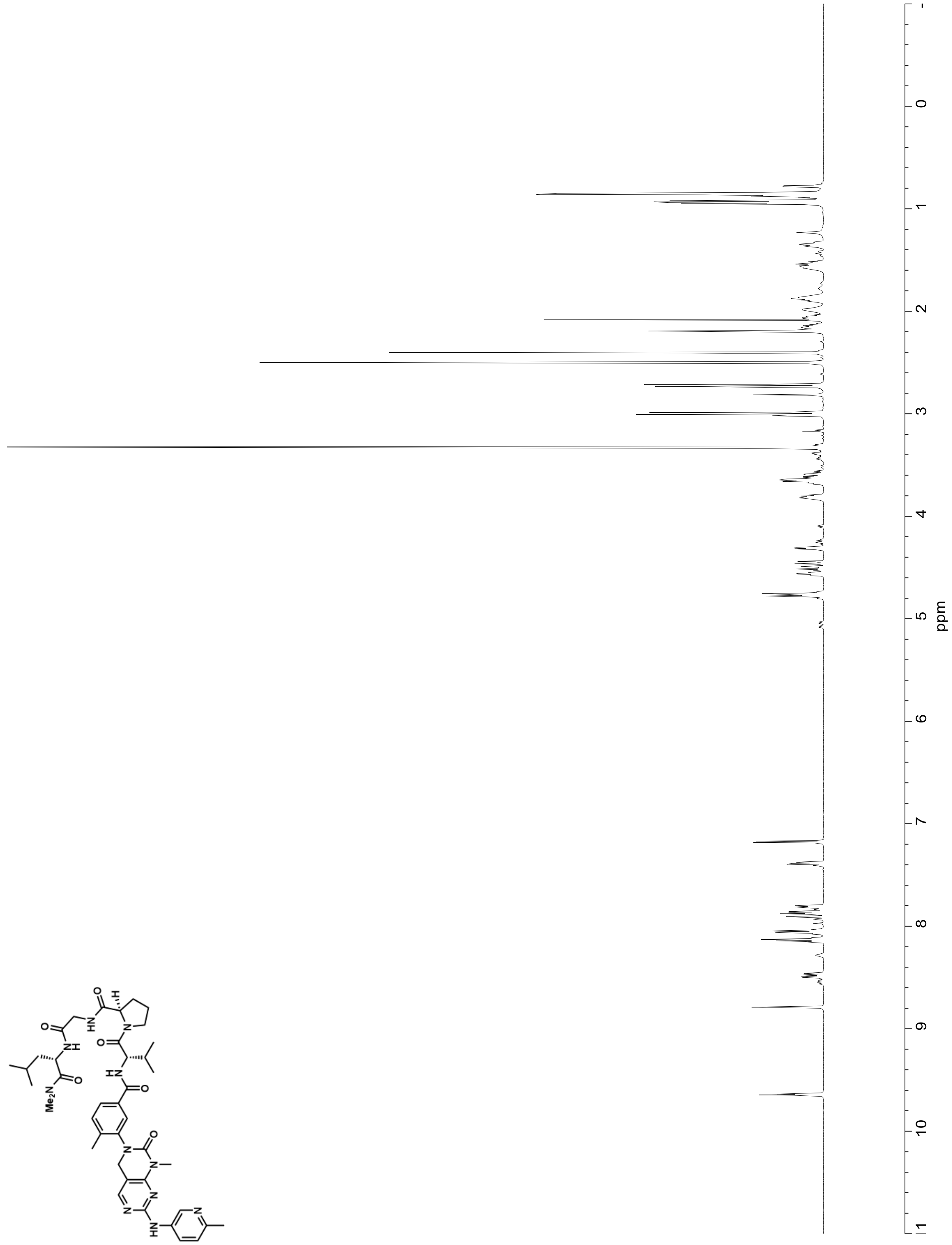

^{1}H NMR (400 MHz, DMSO) of compound **7h**.

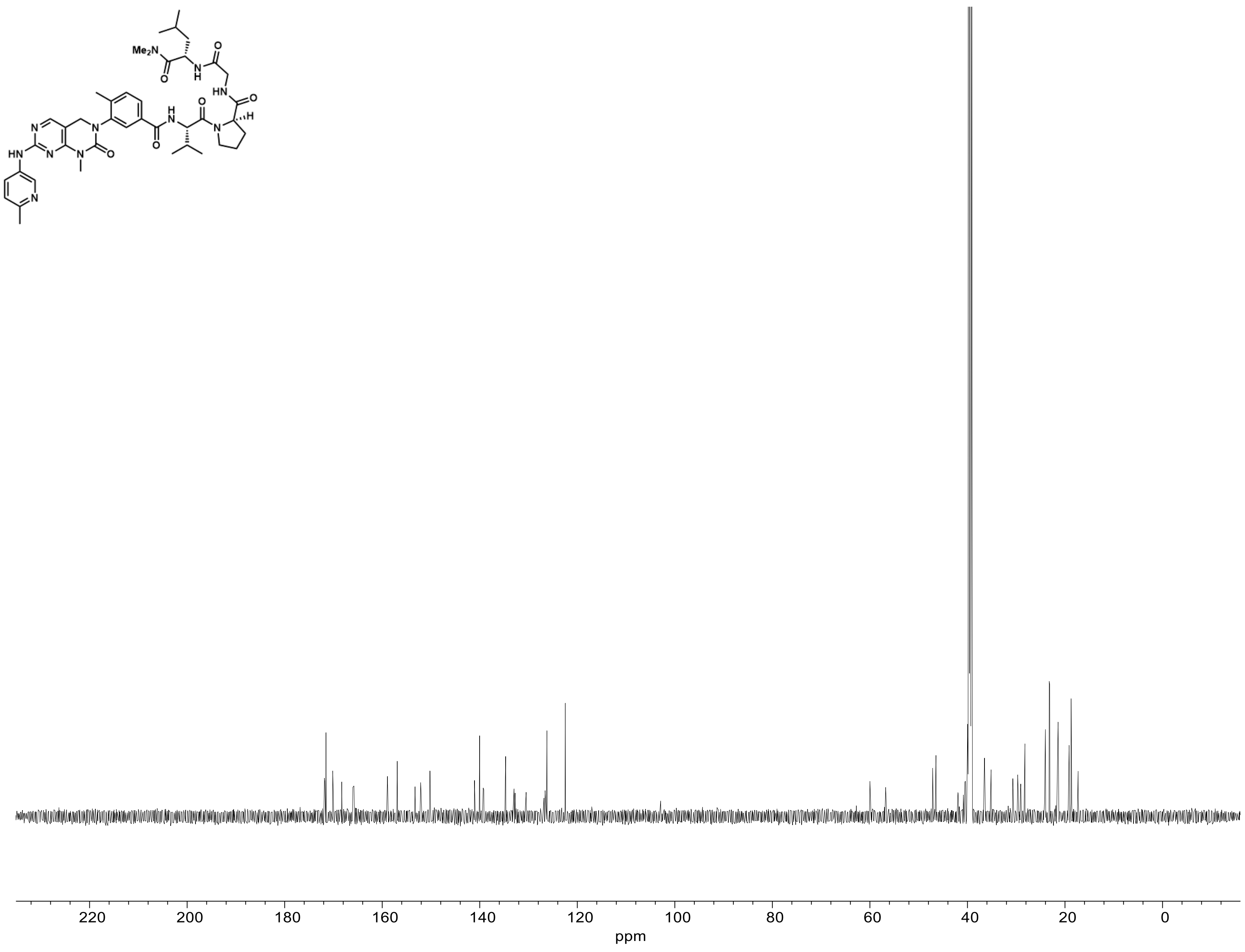

^{13}C NMR (400 MHz, DMSO) of compound **7h**.

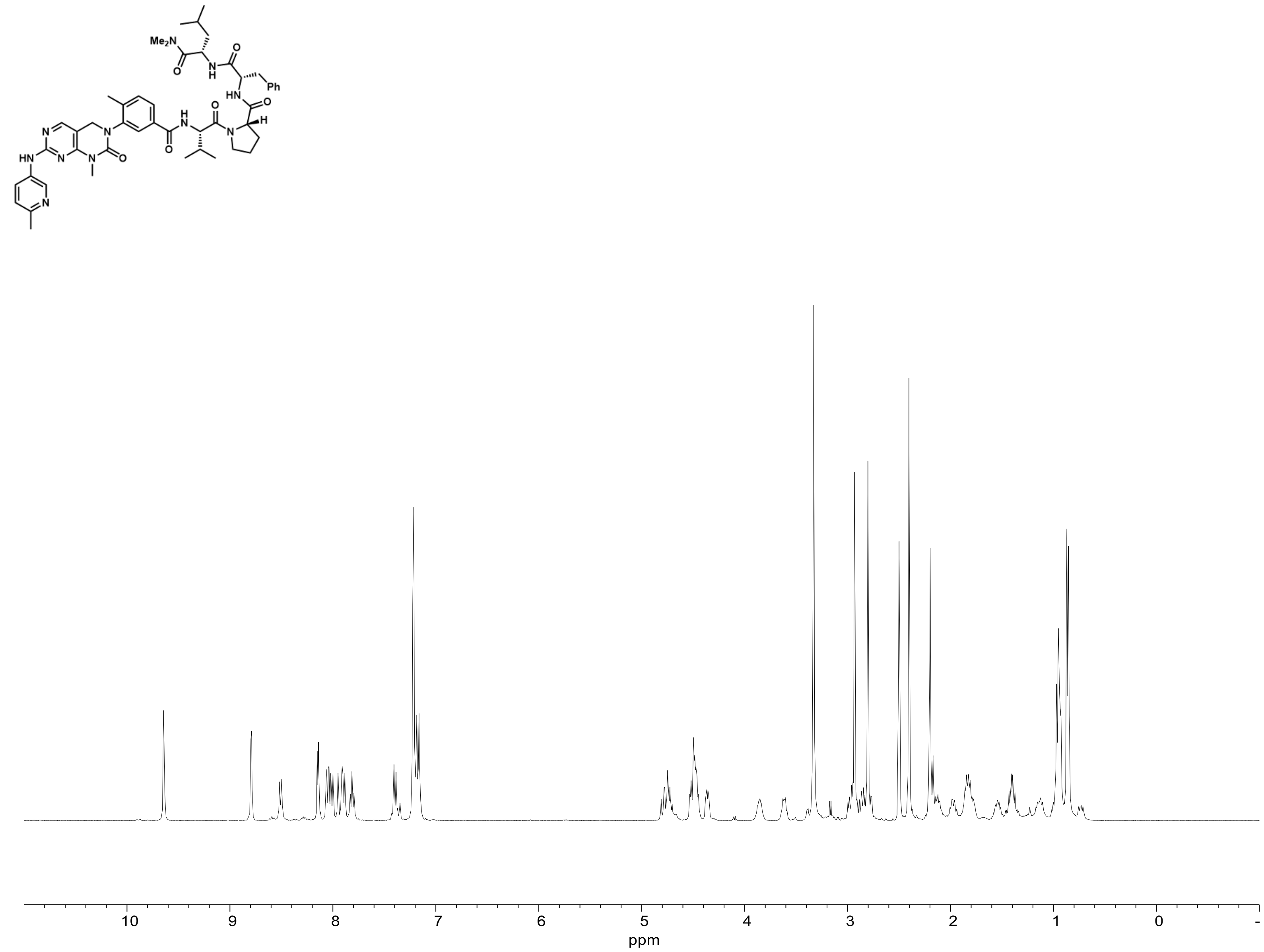

^{1}H NMR (400 MHz, DMSO) of compound **7i**.

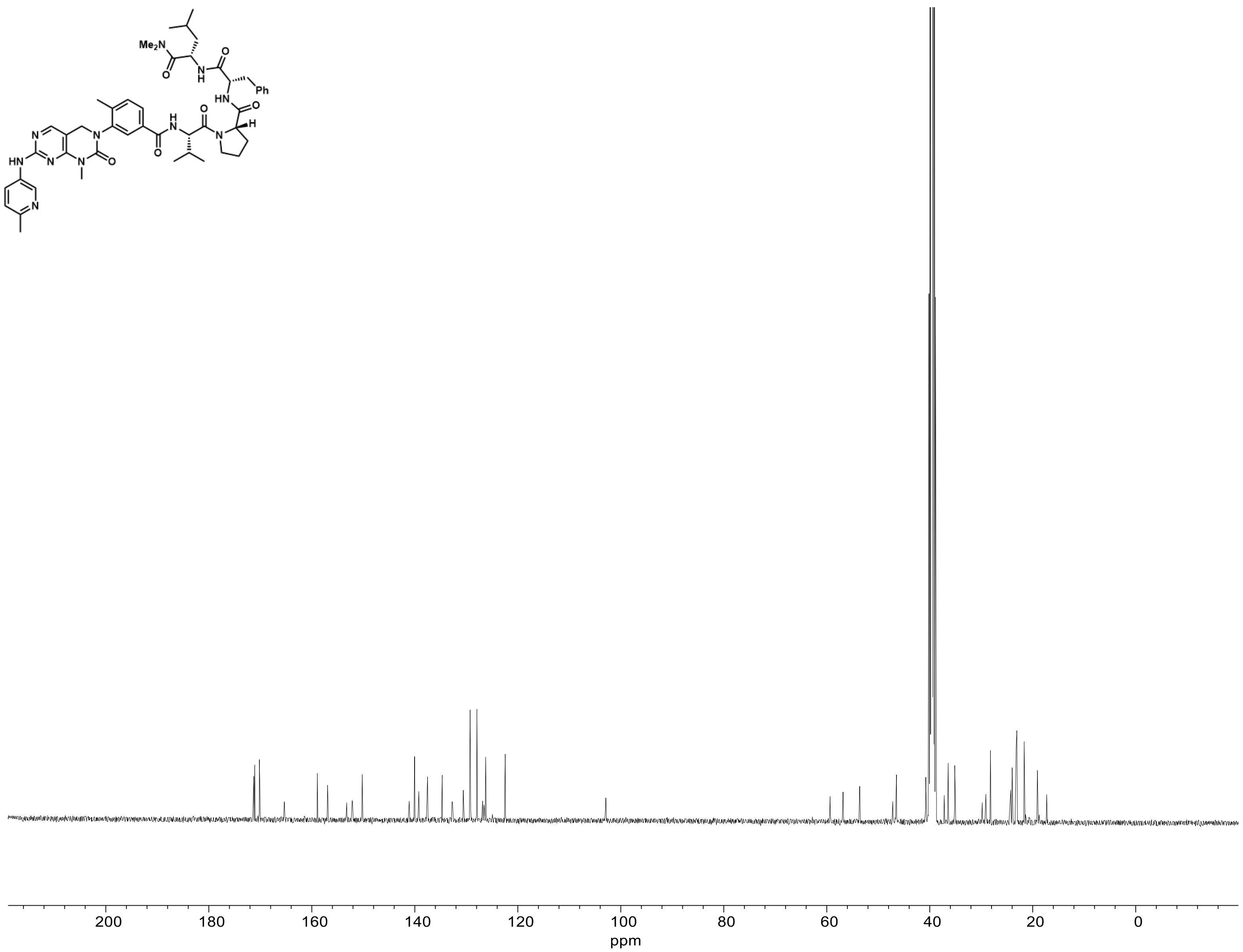

^{13}C NMR (400 MHz, DMSO) of compound **7i**.

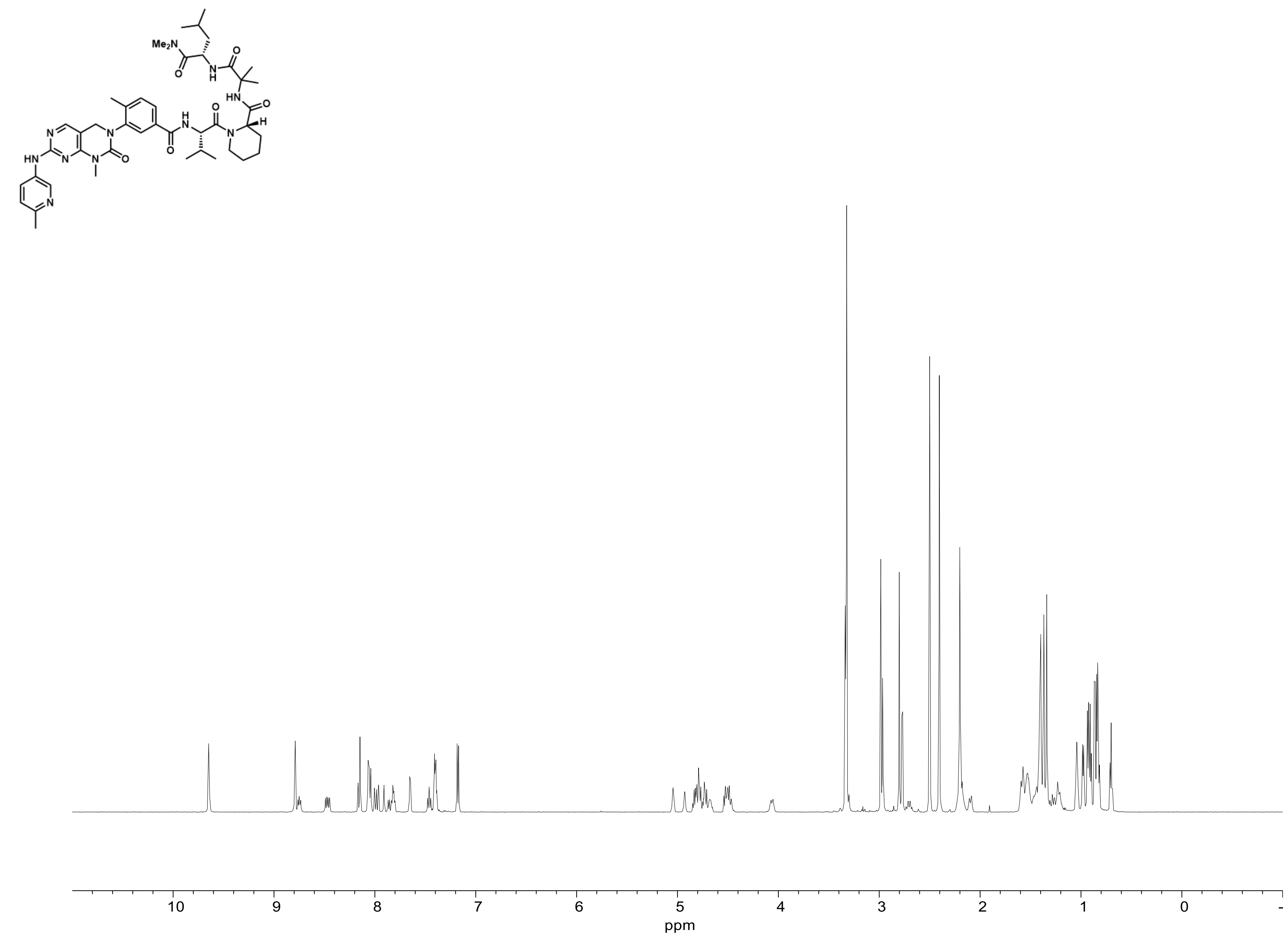

^{1}H NMR (400 MHz, DMSO) of compound **7j**.

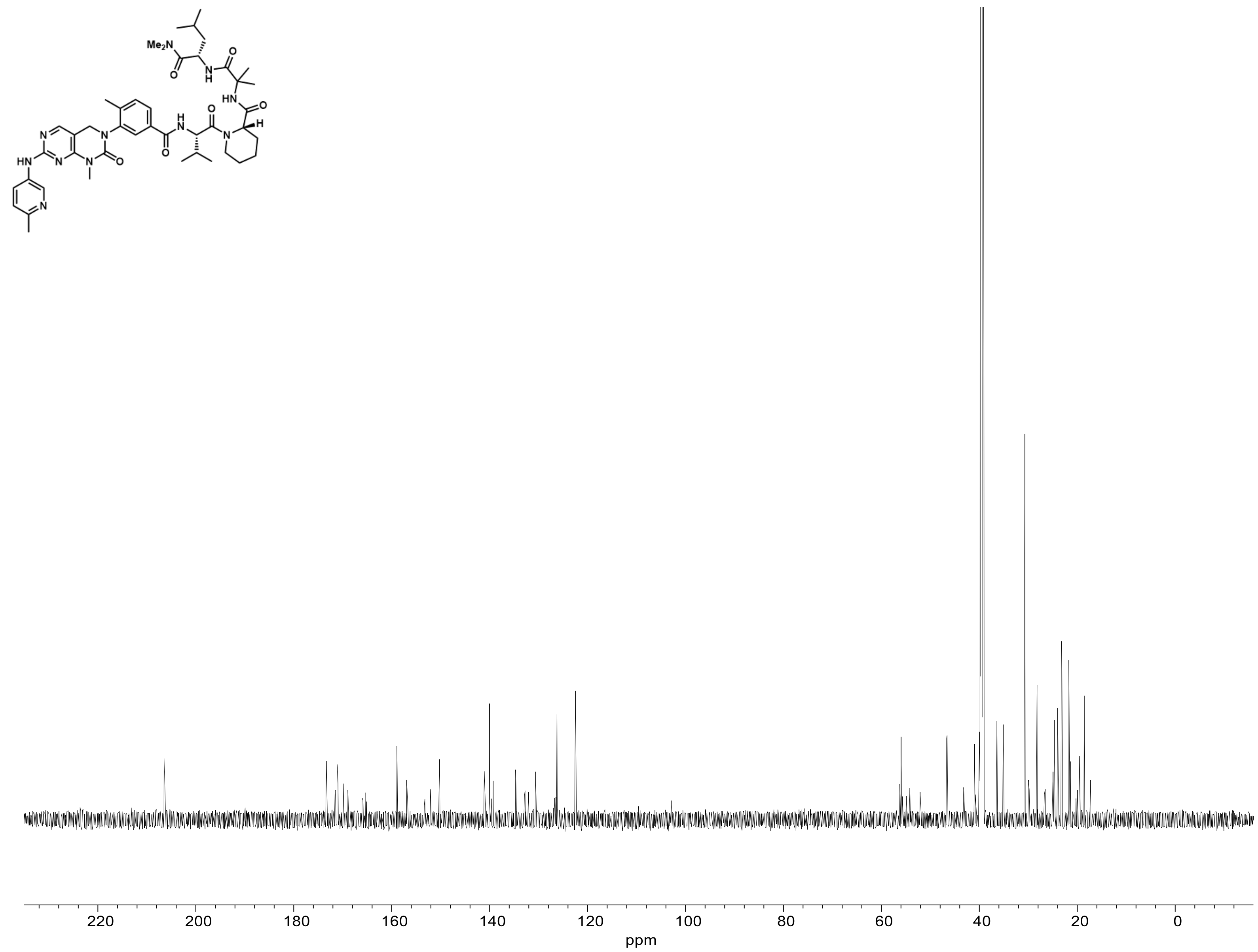

^{13}C NMR (400 MHz, DMSO) of compound **7j**.

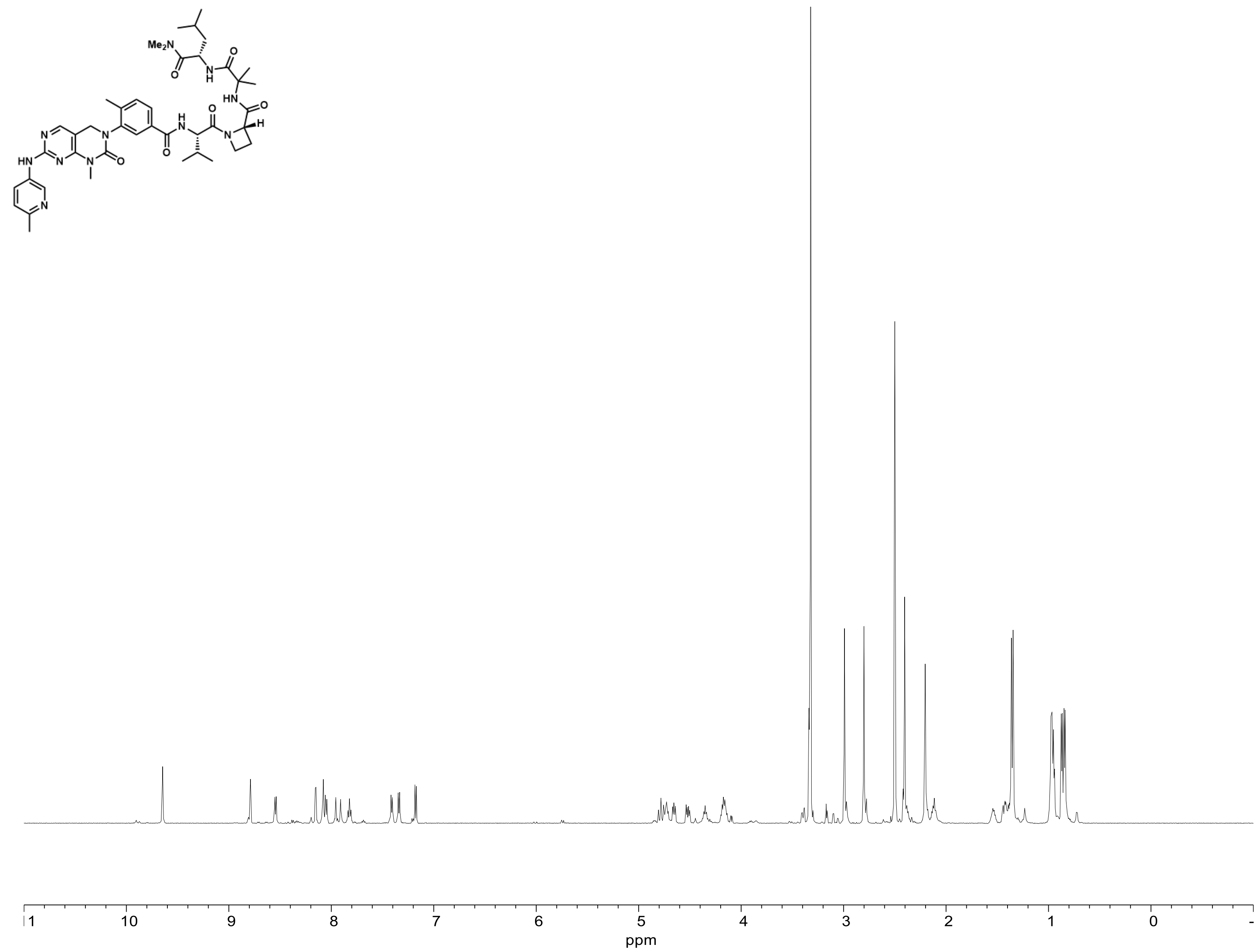

^{1}H NMR (400 MHz, DMSO) of compound **7k**.

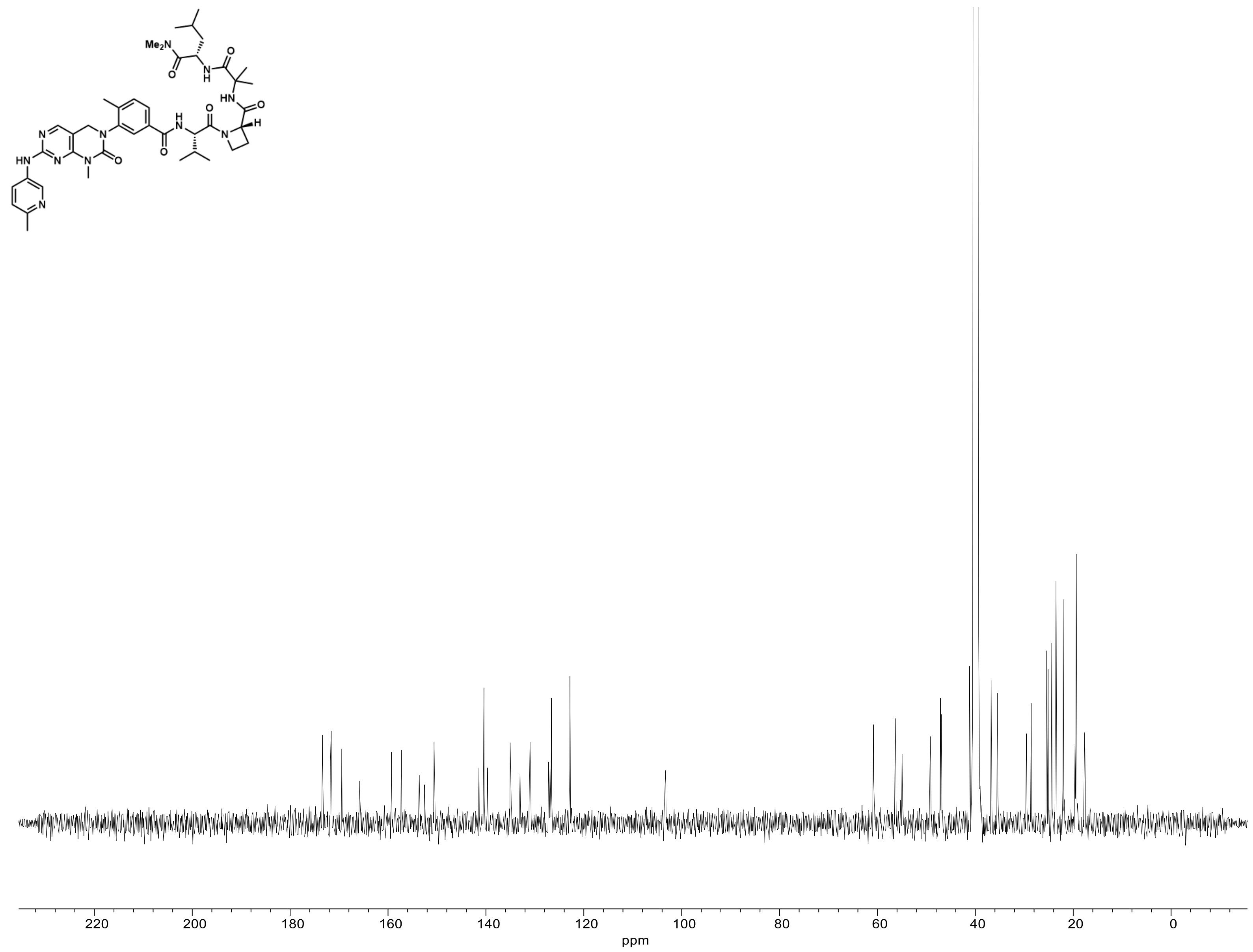

^{13}C NMR (400 MHz, DMSO) of compound **7k**.

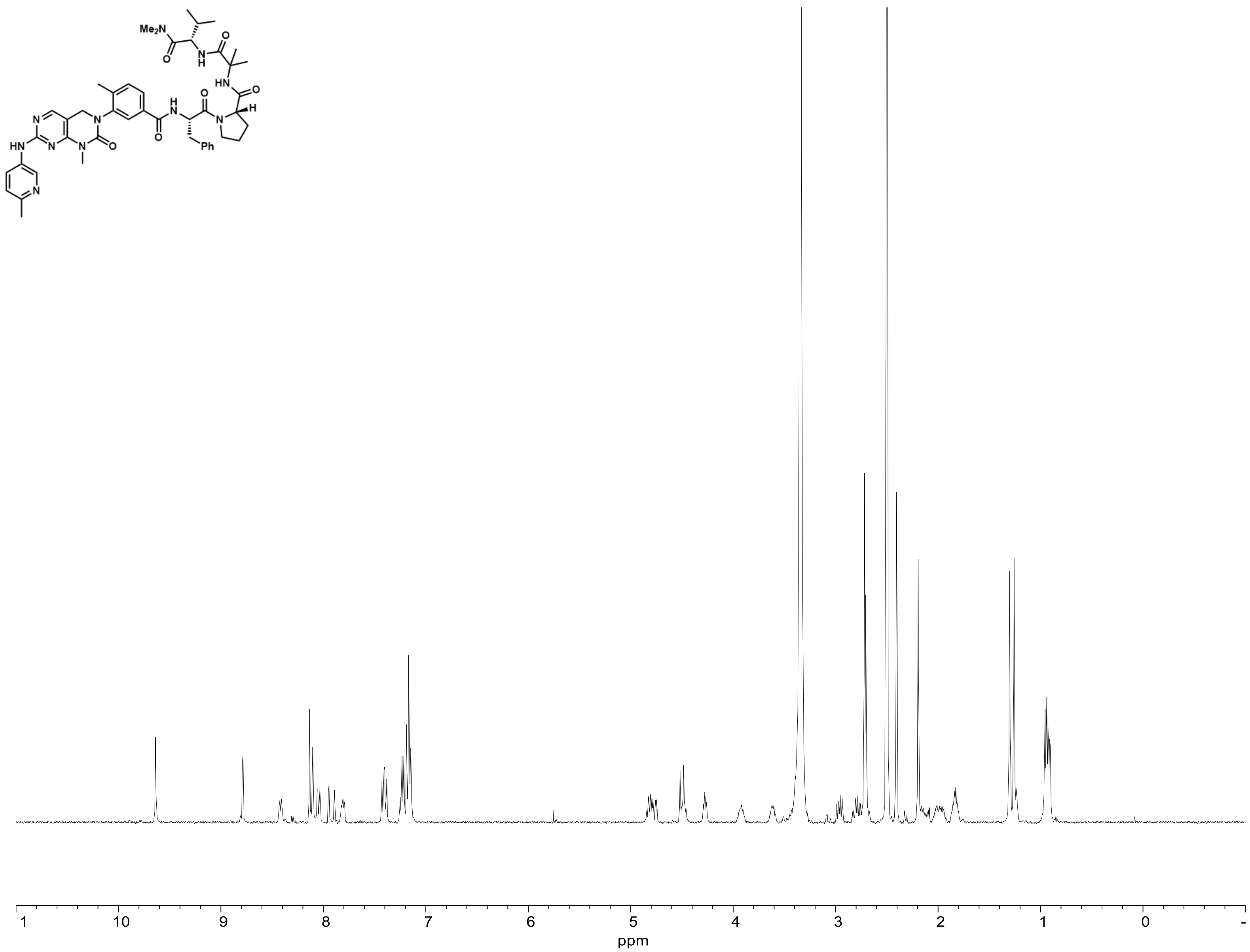

^{1}H NMR (400 MHz, DMSO) of compound **7l**.

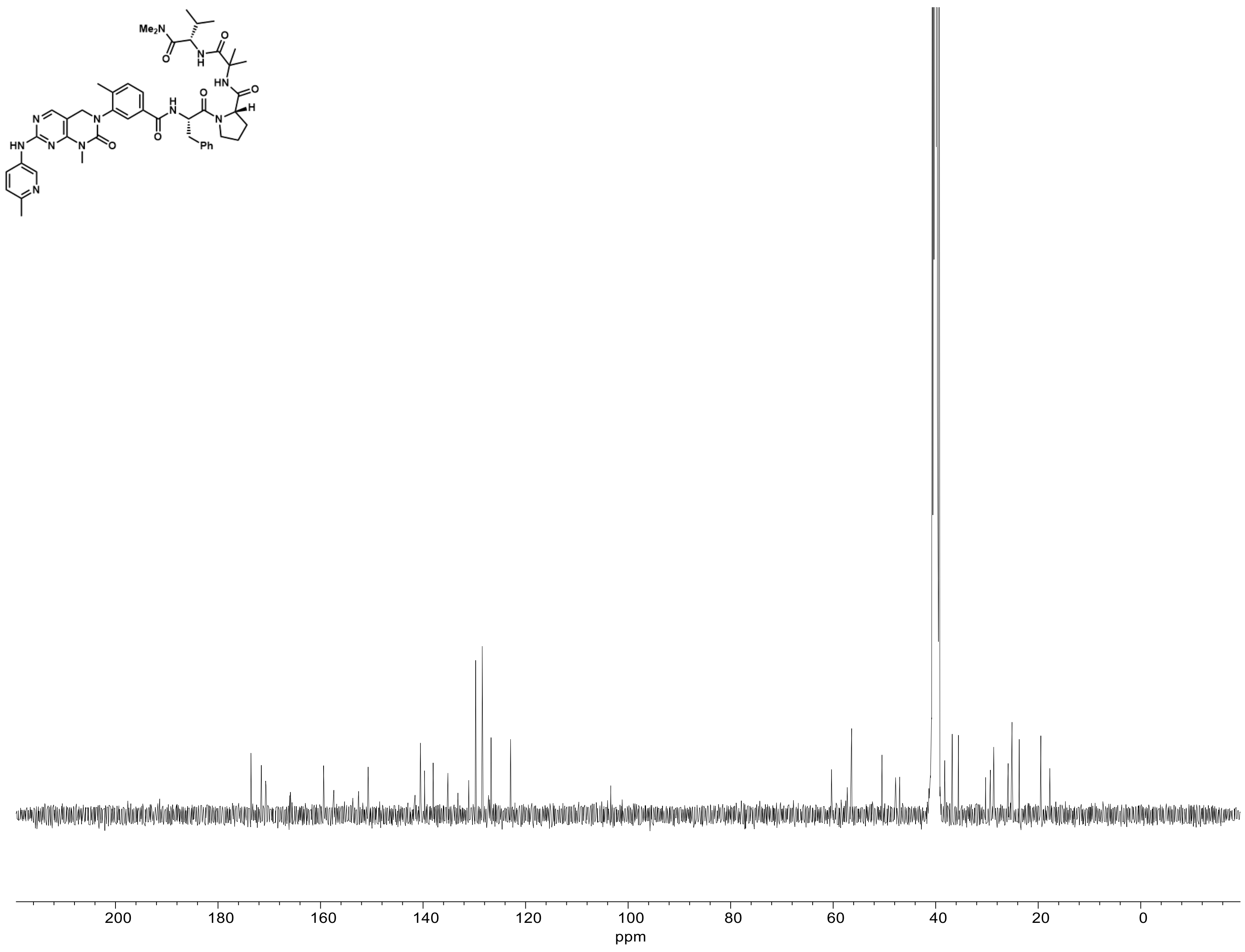

^{13}C NMR (400 MHz, DMSO) of compound **7l**.

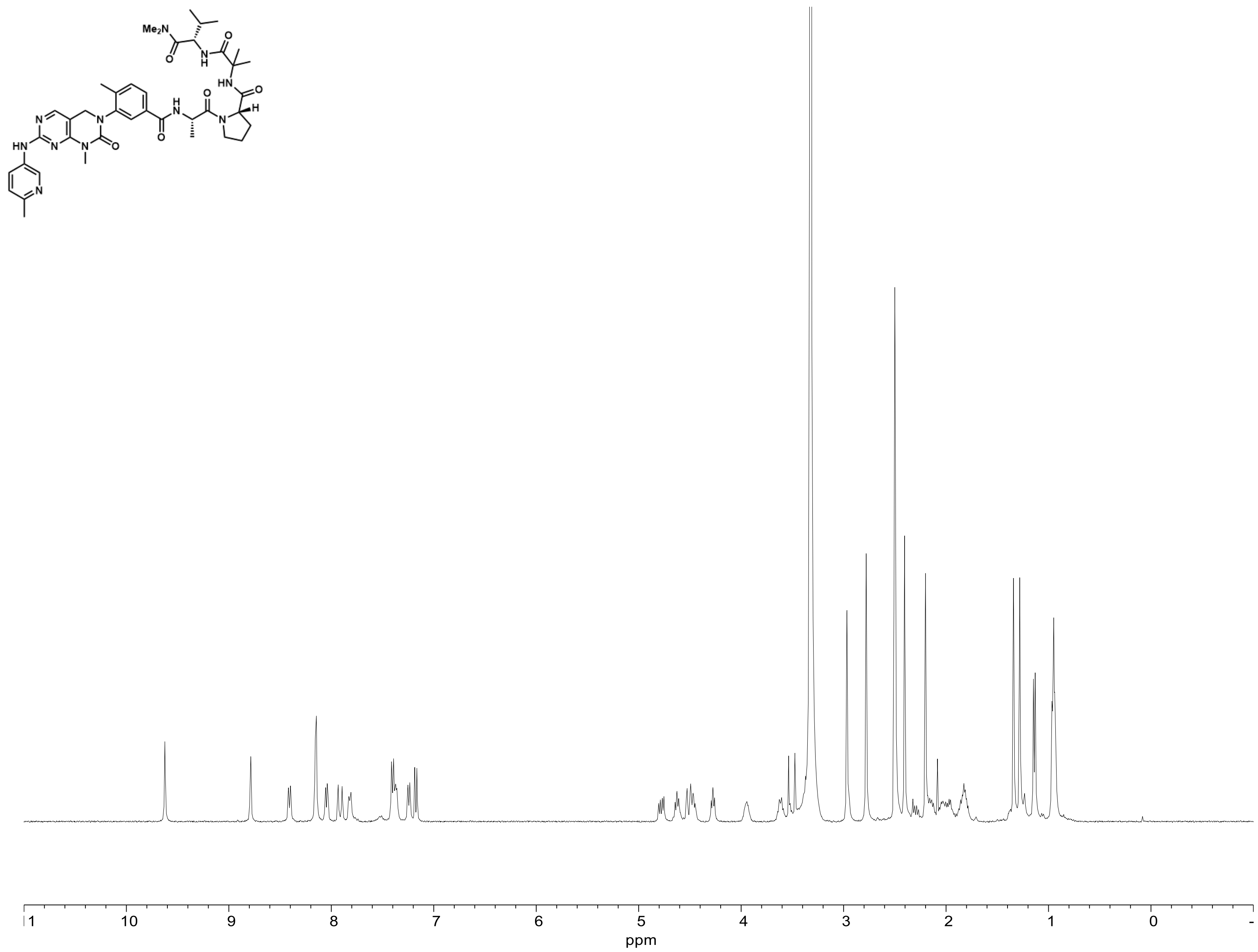

^{1}H NMR (400 MHz, DMSO) of compound **7m**.

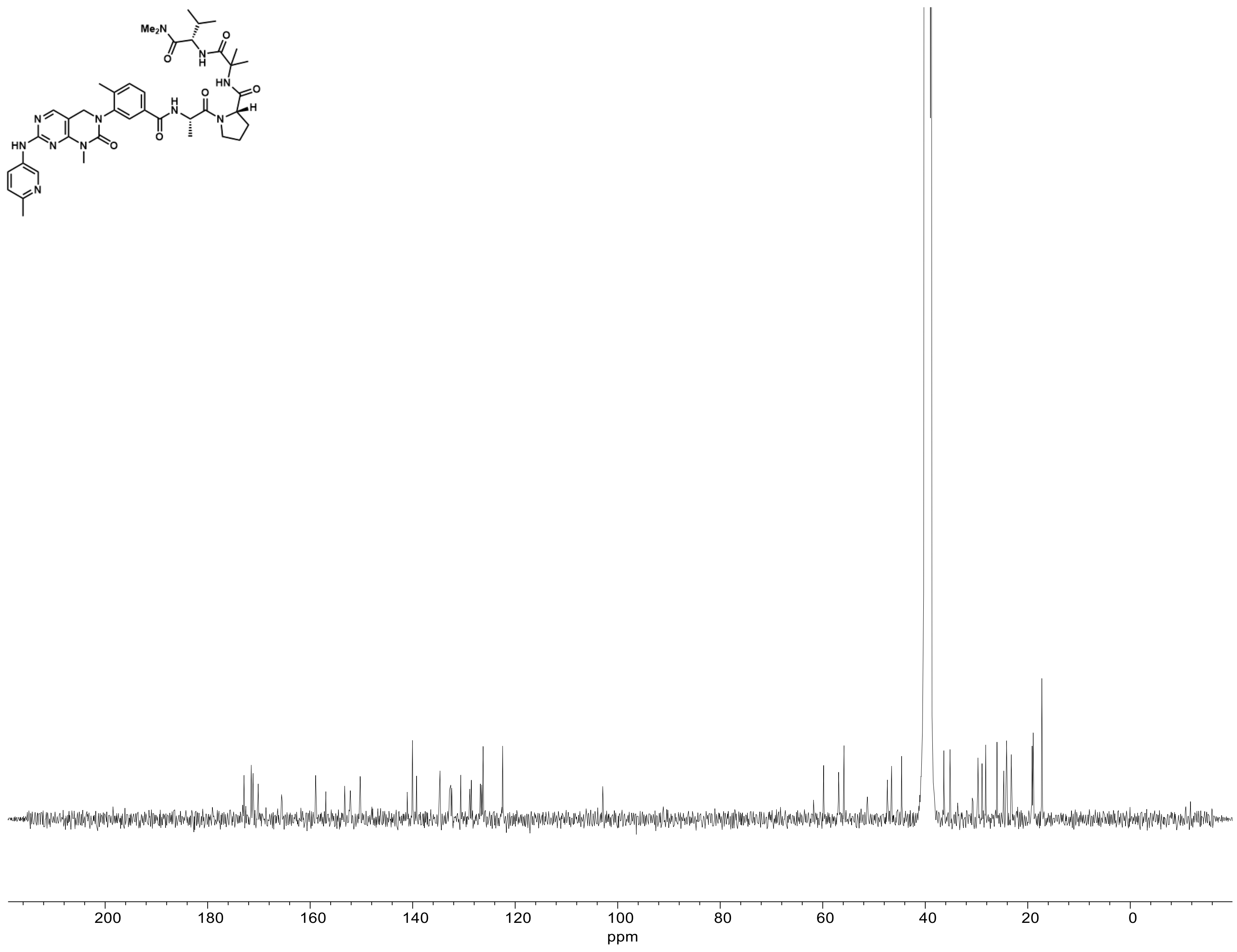

^{13}C NMR (400 MHz, DMSO) of compound **7m**.

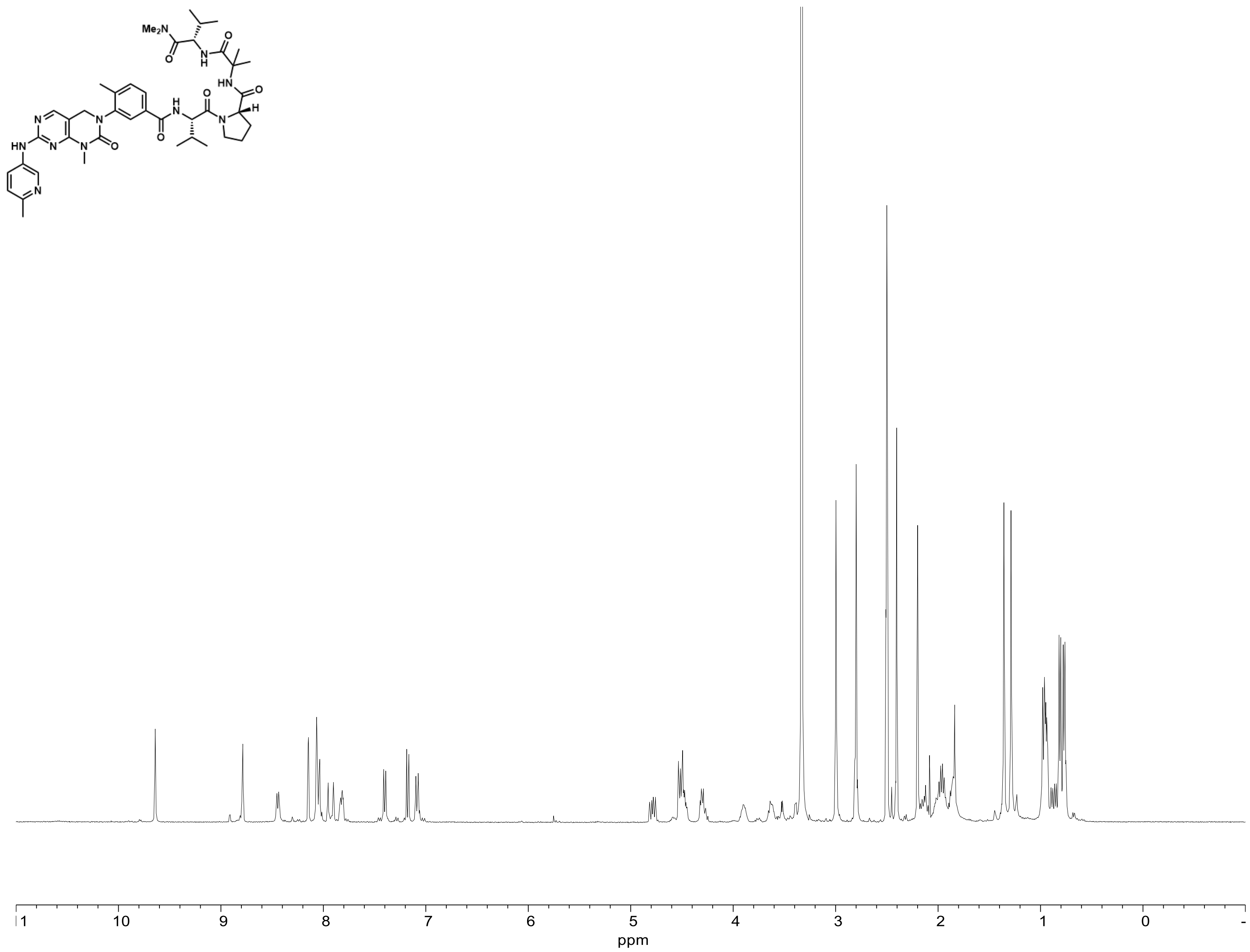

^{1}H NMR (400 MHz, DMSO) of compound **7n**.

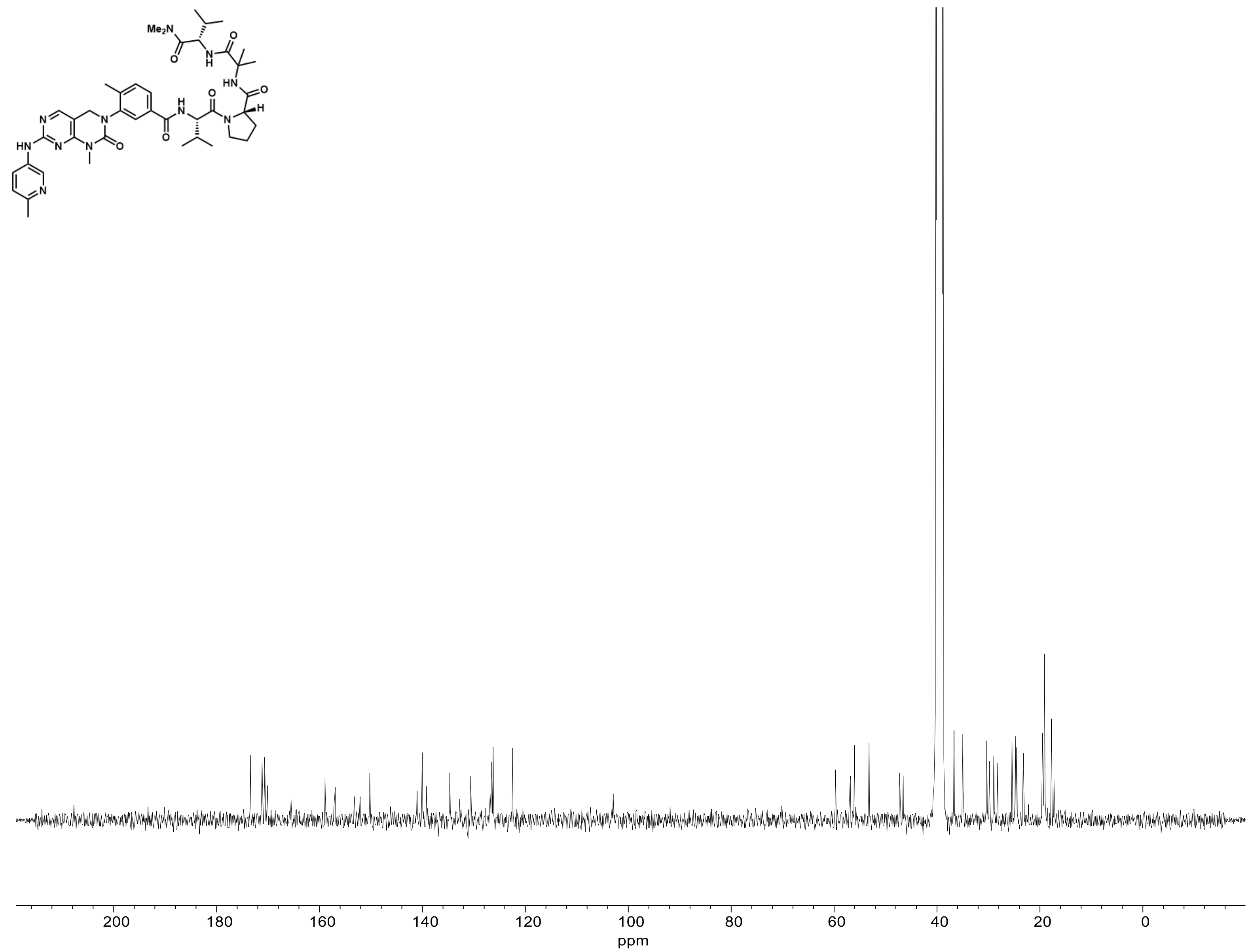

^{13}C NMR (400 MHz, DMSO) of compound **7n**.

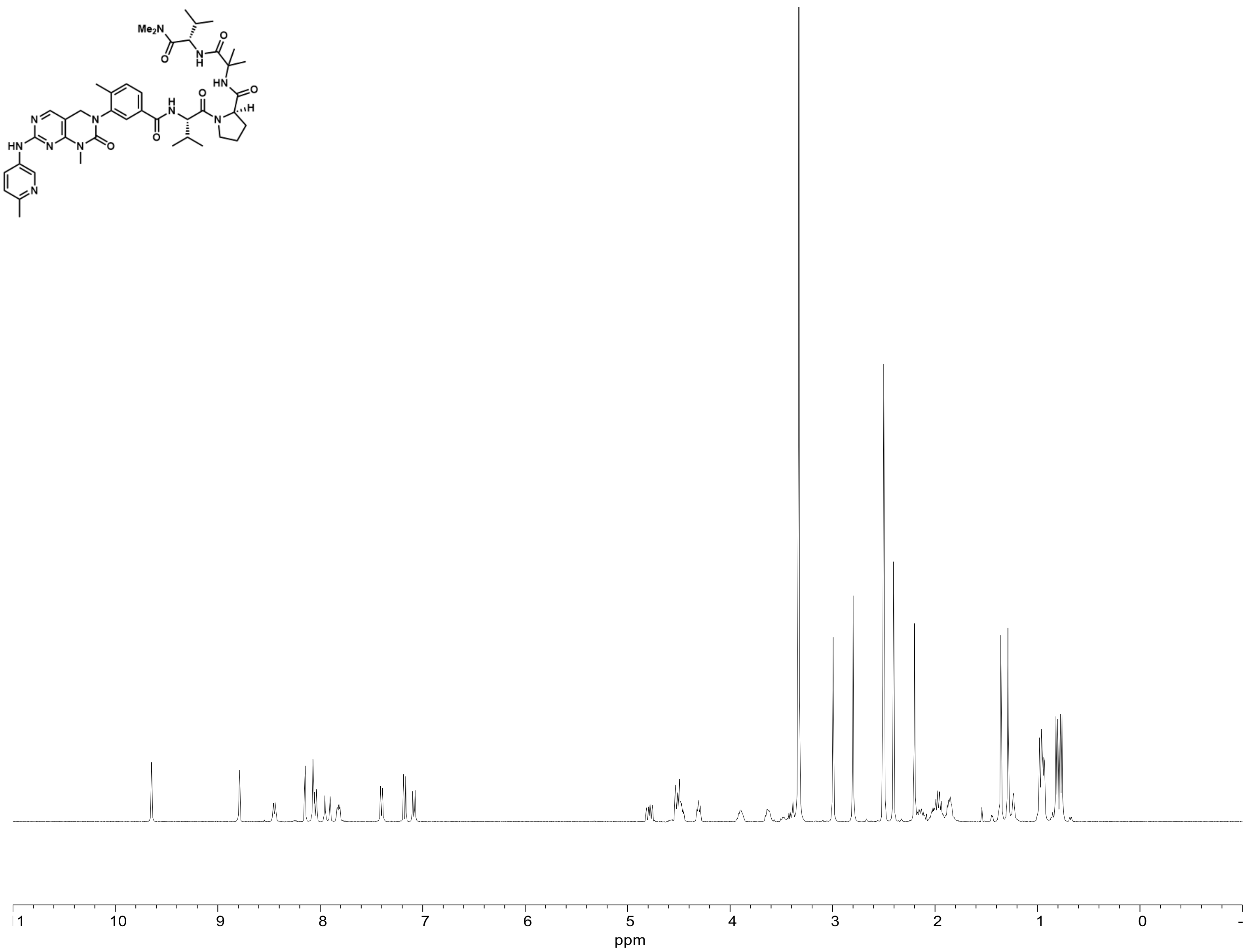

^{1}H NMR (400 MHz, DMSO) of compound **7o**.

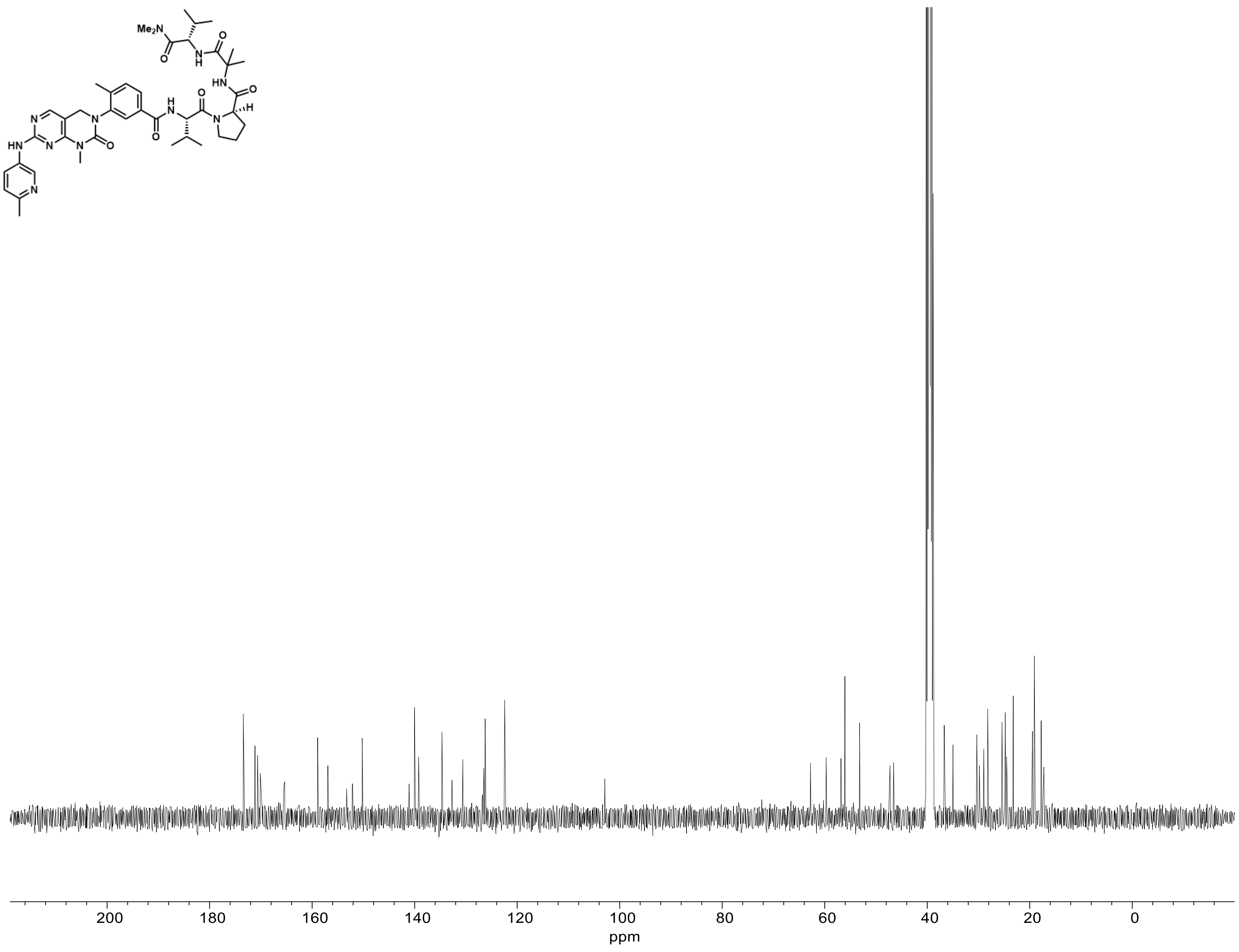

^{13}C NMR (400 MHz, DMSO) of compound **7o**.

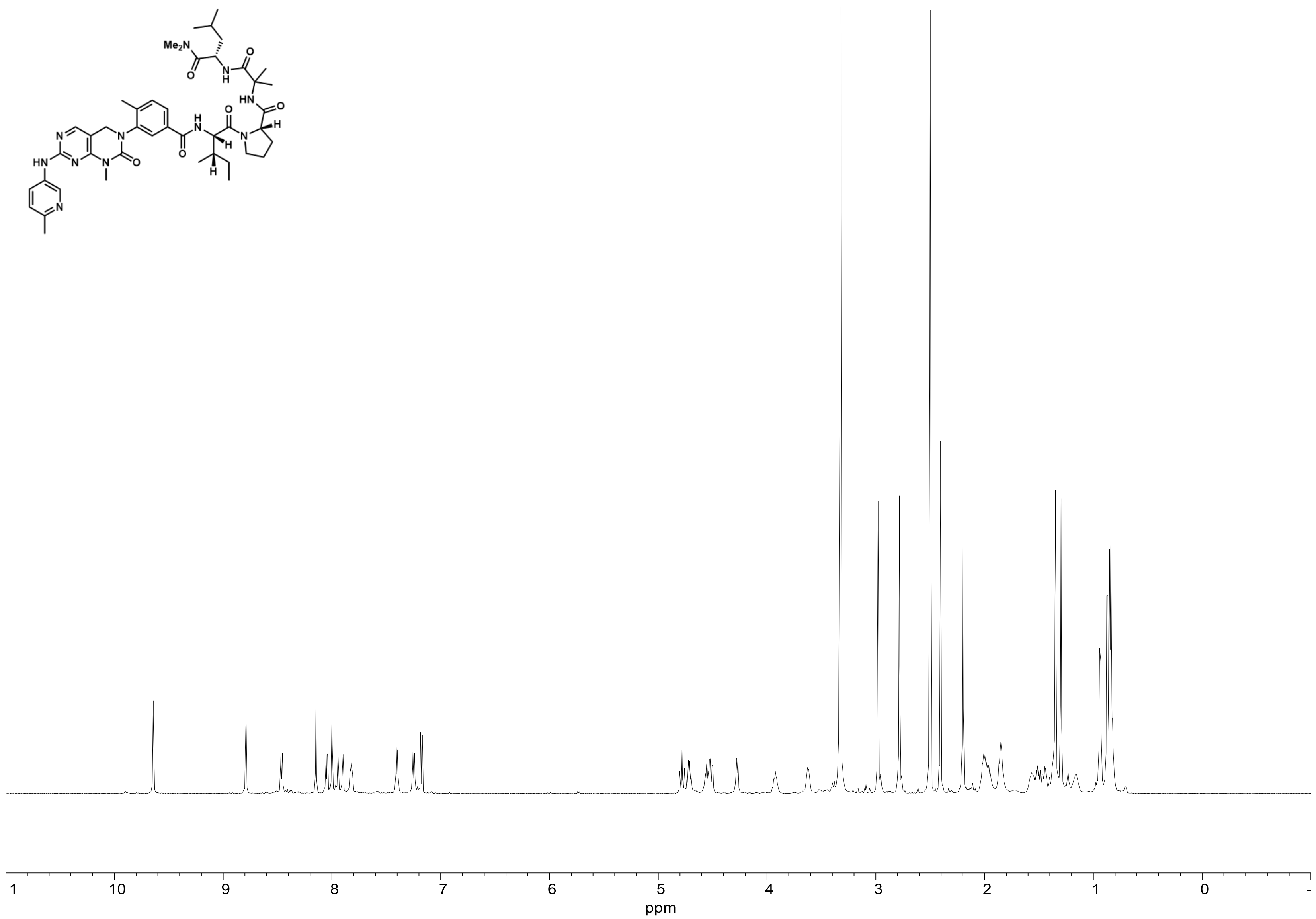

^{1}H NMR (400 MHz, DMSO) of compound **7p**.

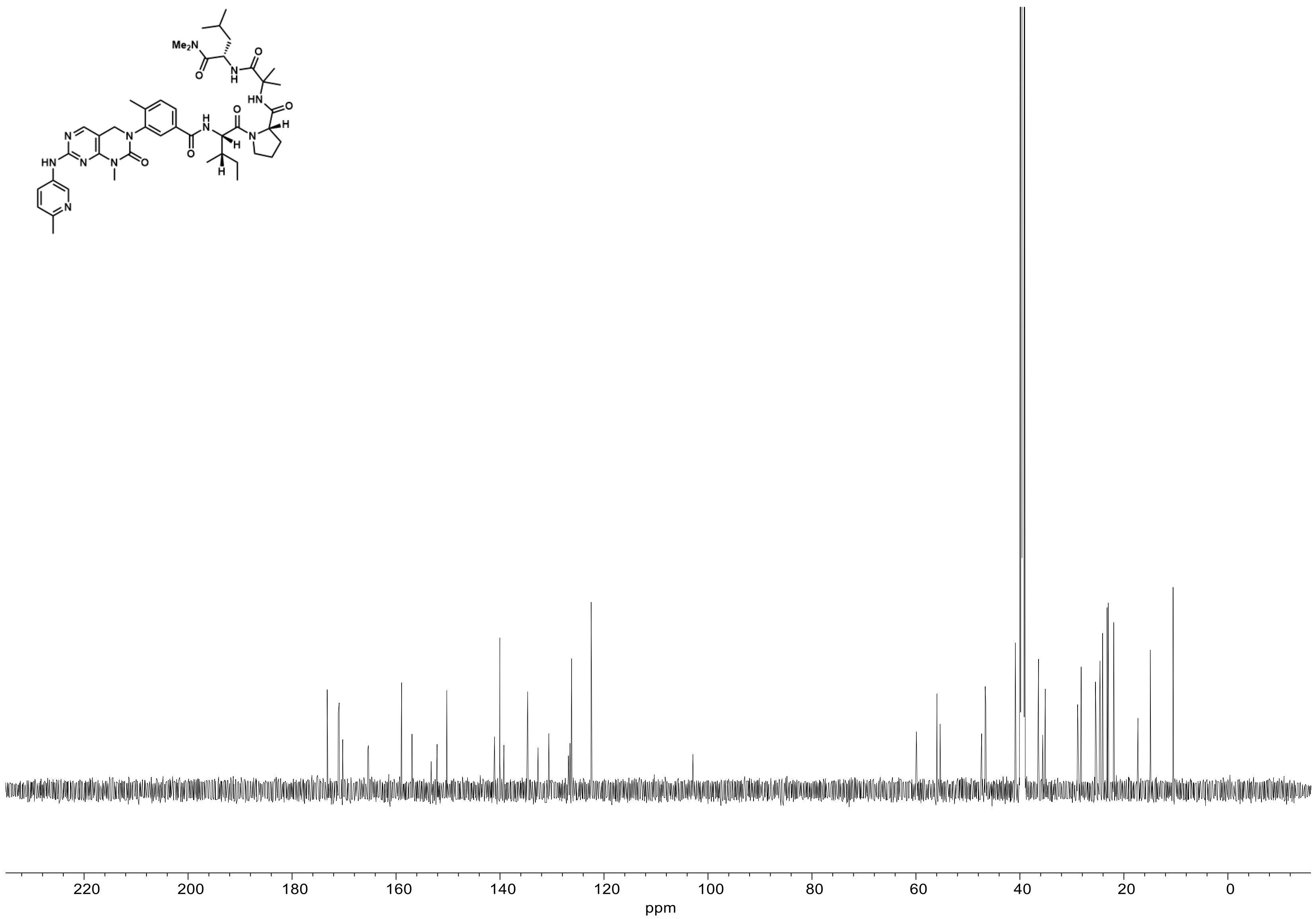

^{13}C NMR (400 MHz, DMSO) of compound **7p**.

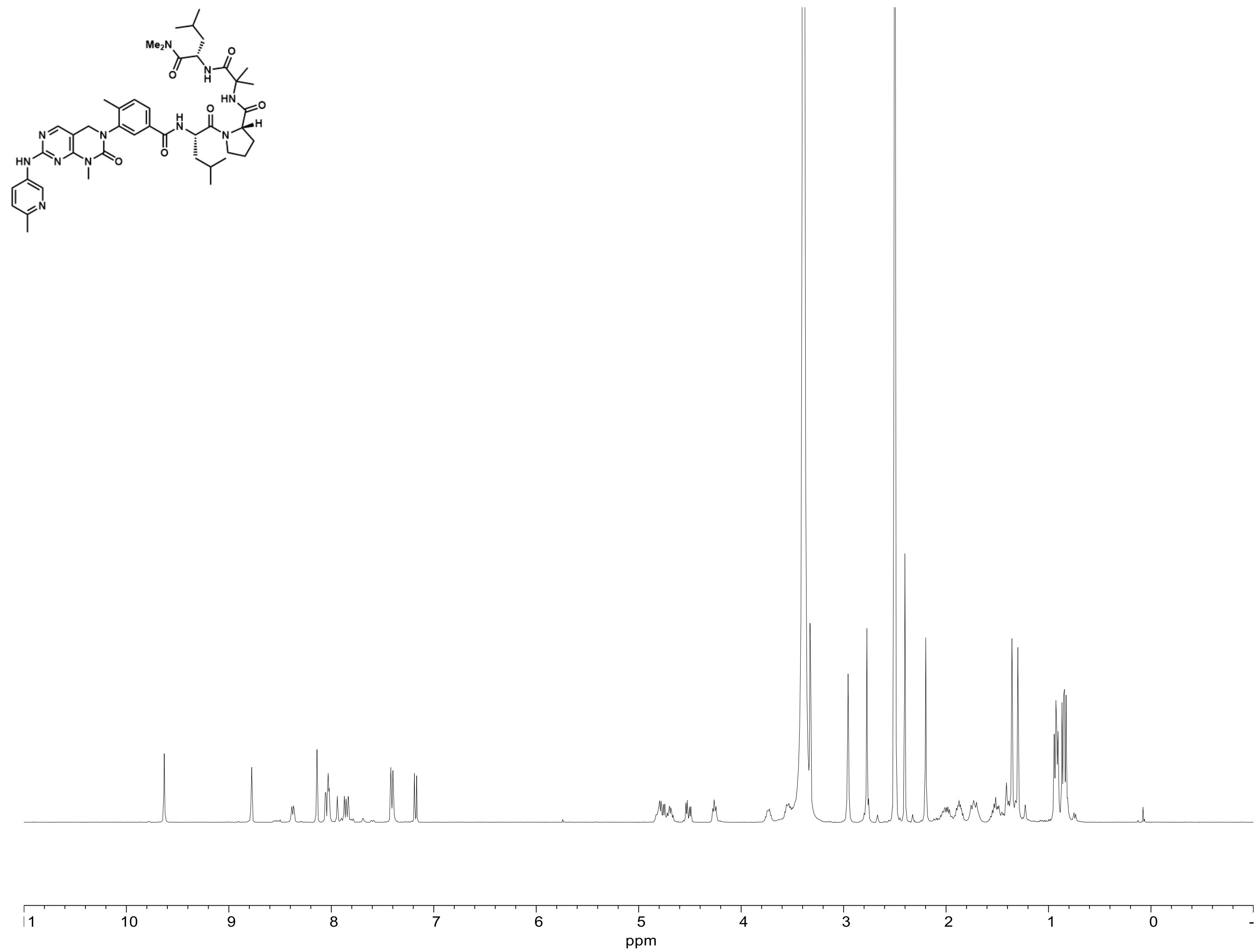

^{1}H NMR (400 MHz, DMSO) of compound **7q**.

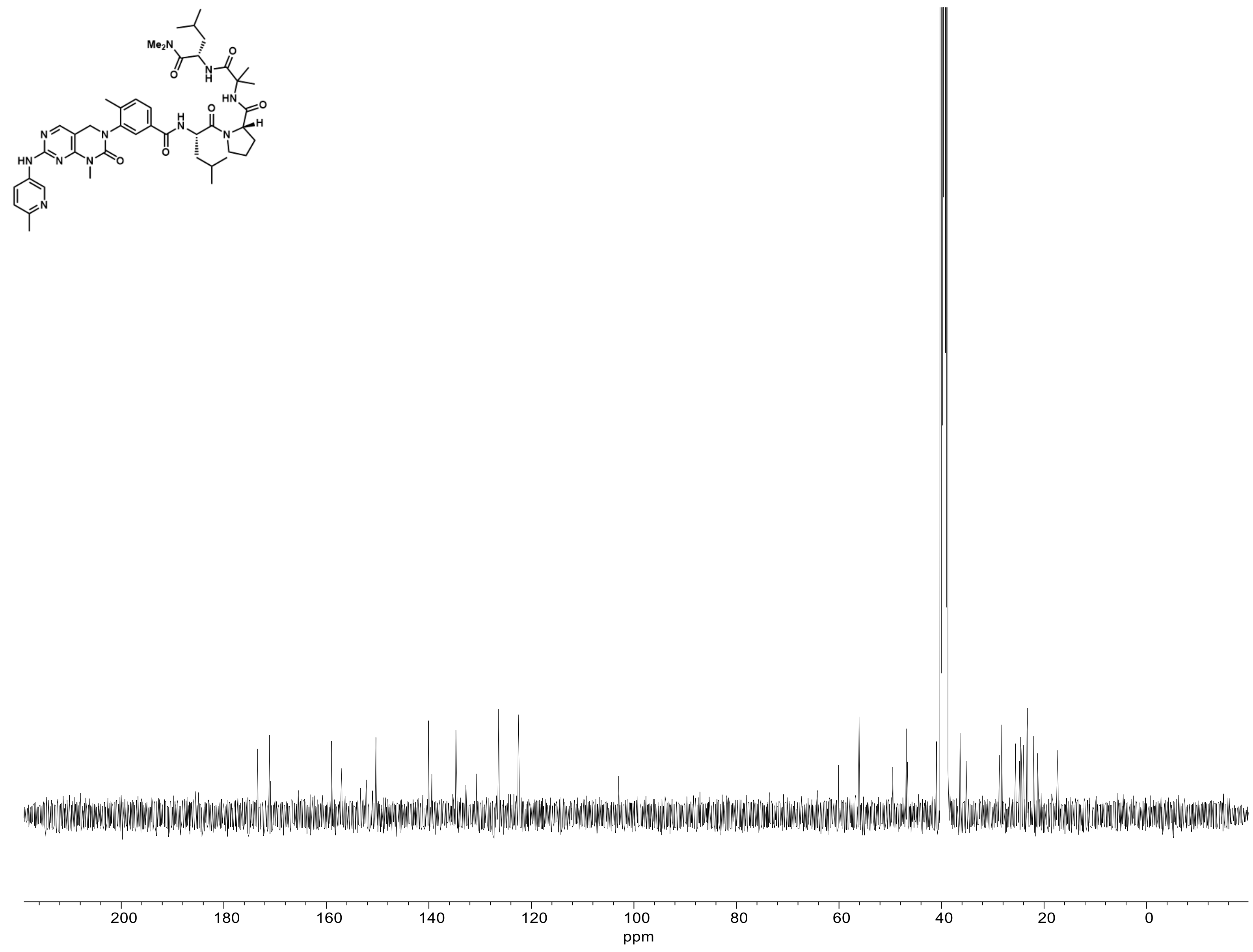

^{13}C NMR (400 MHz, DMSO) of compound **7q**.

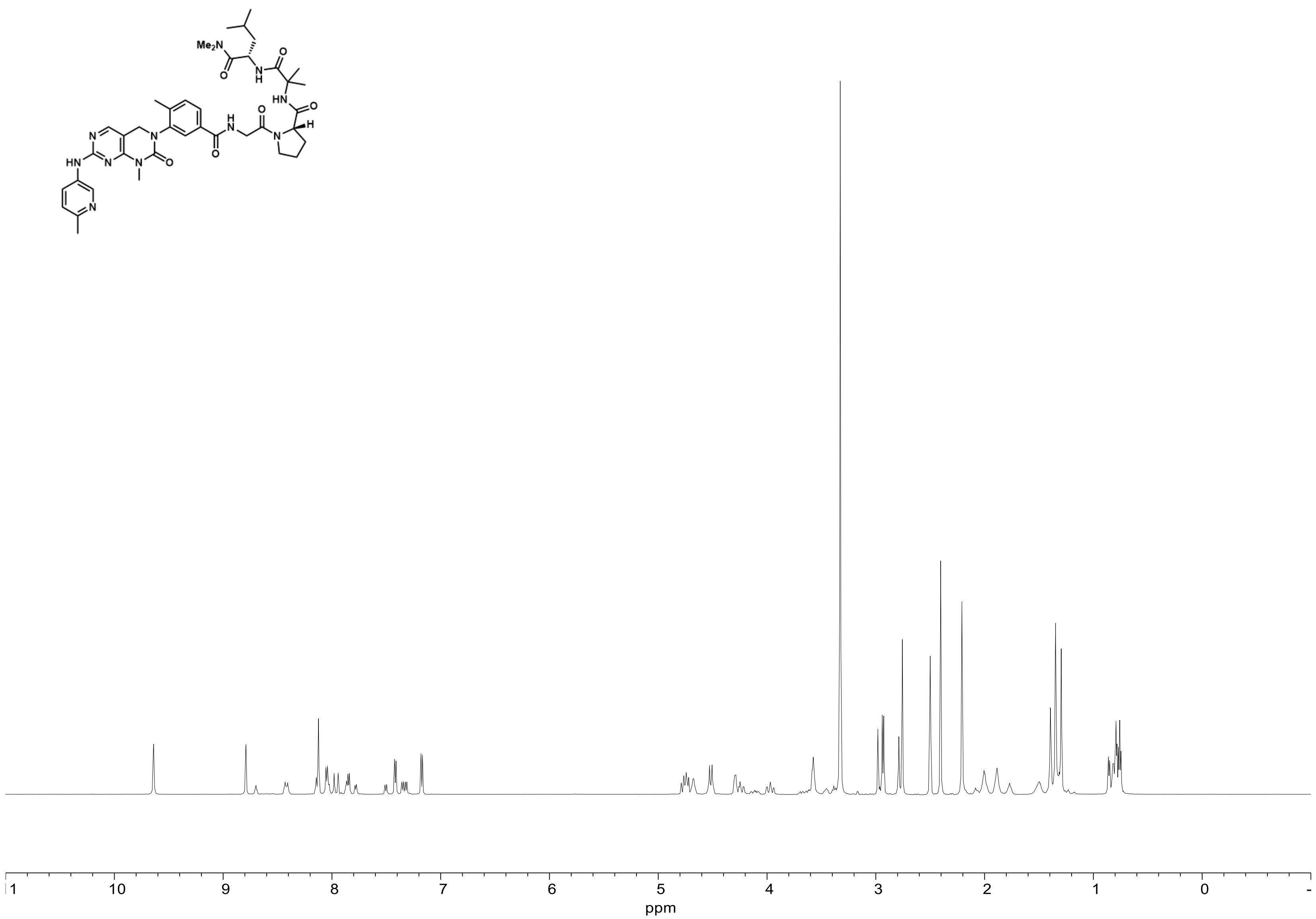

^{1}H NMR (400 MHz, DMSO) of compound **7r**.

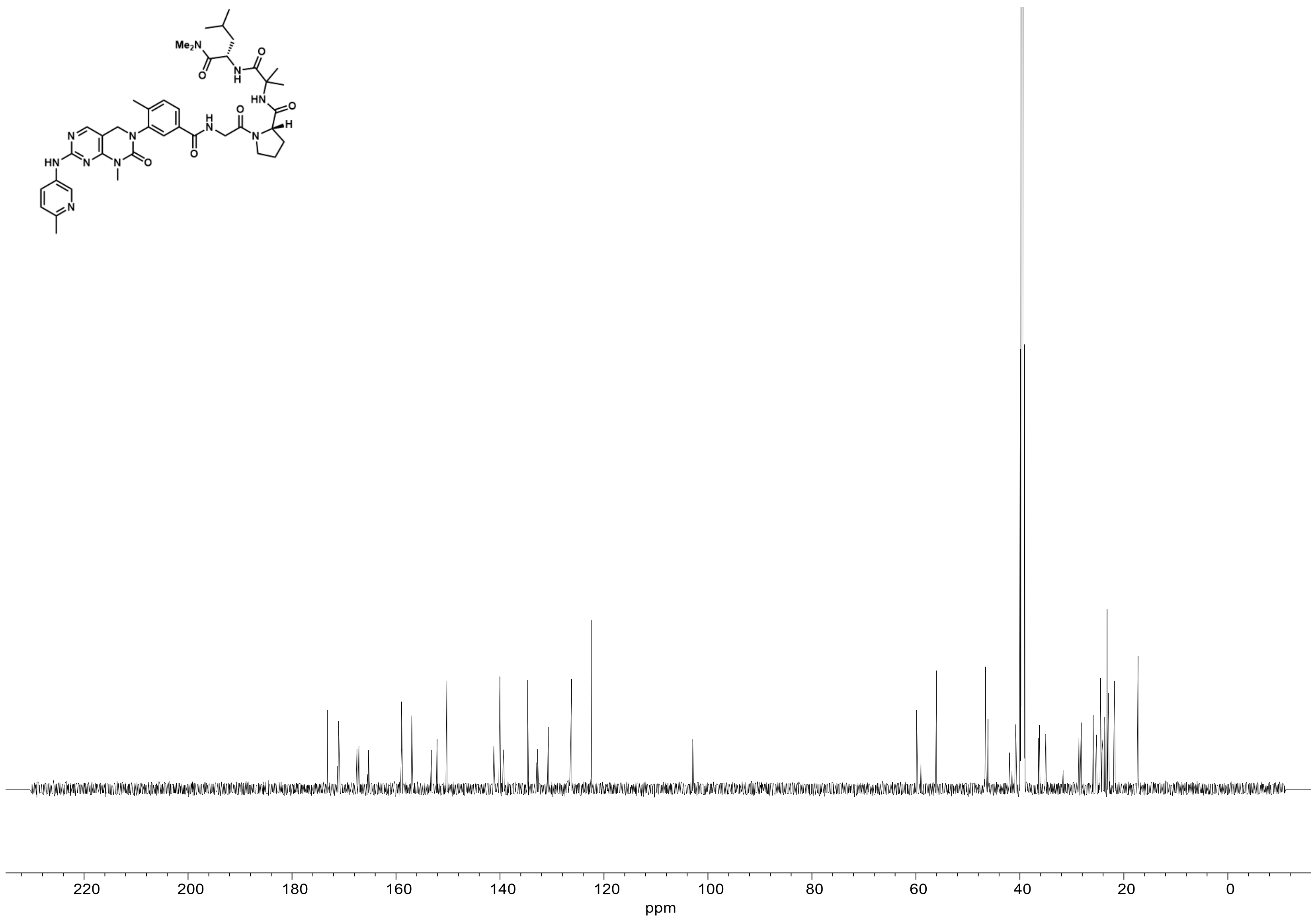

^{13}C NMR (400 MHz, DMSO) of compound **7r**.

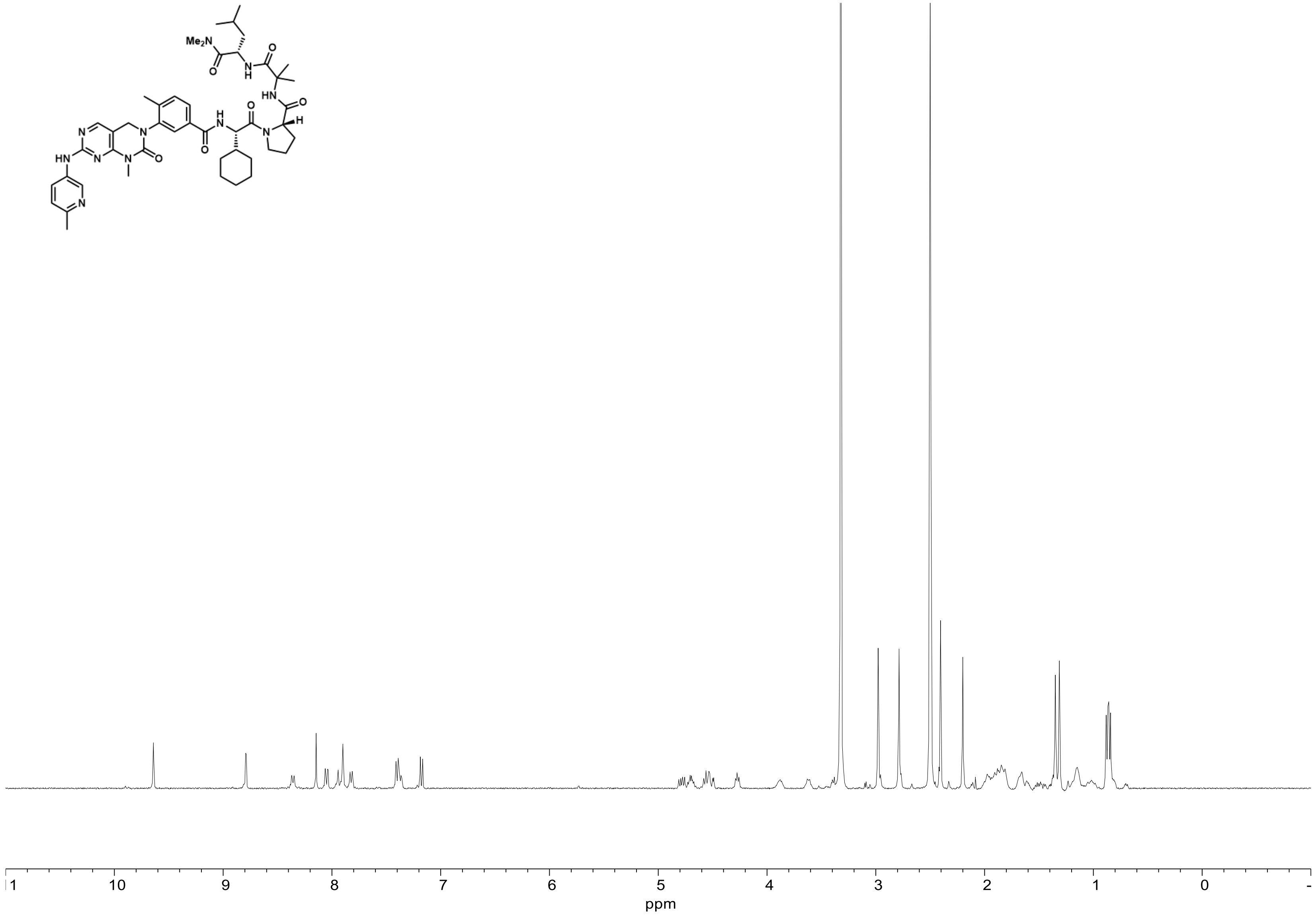

^{1}H NMR (400 MHz, DMSO) of compound **7s**.

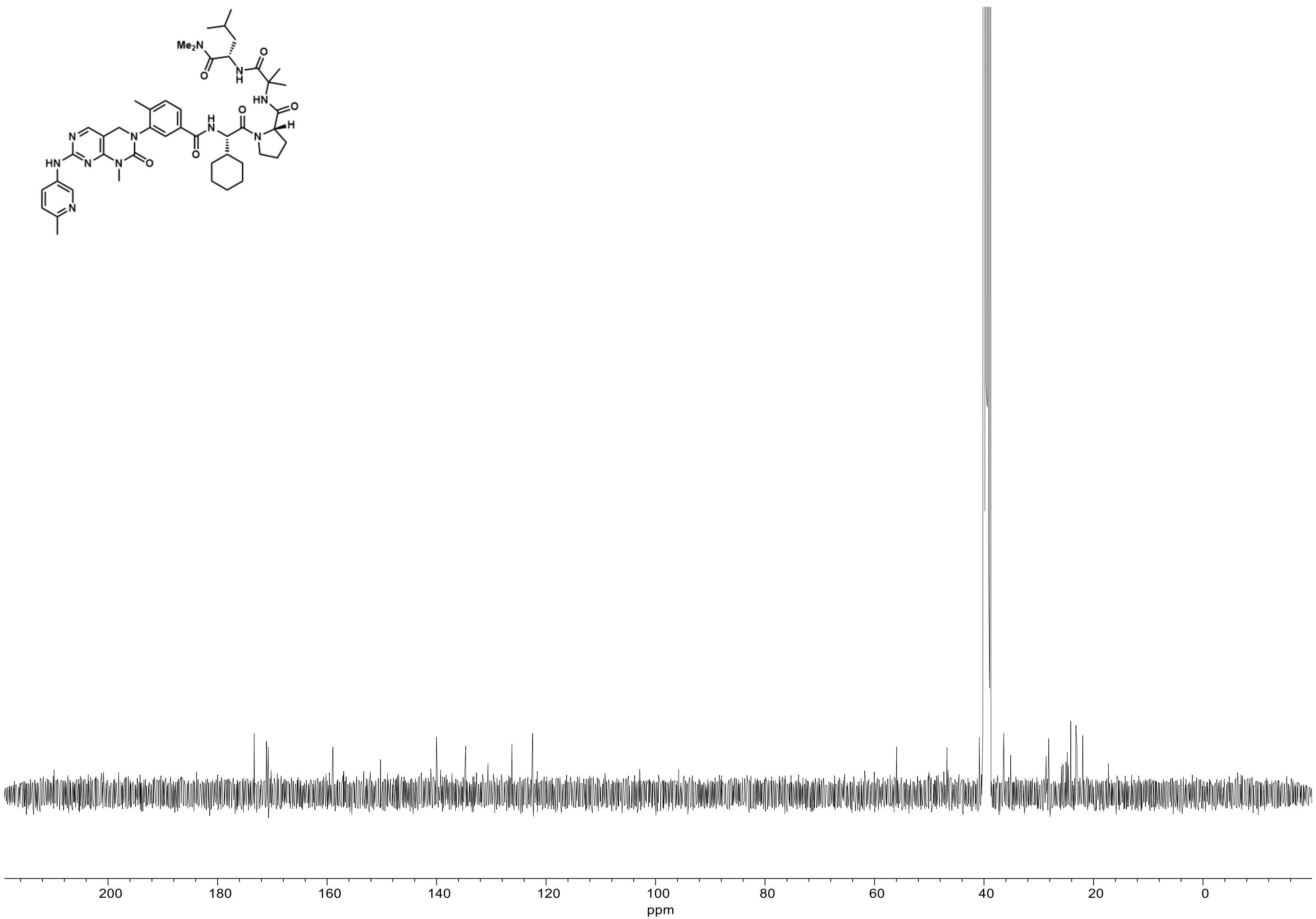

^{13}C NMR (400 MHz, DMSO) of compound **7s**.

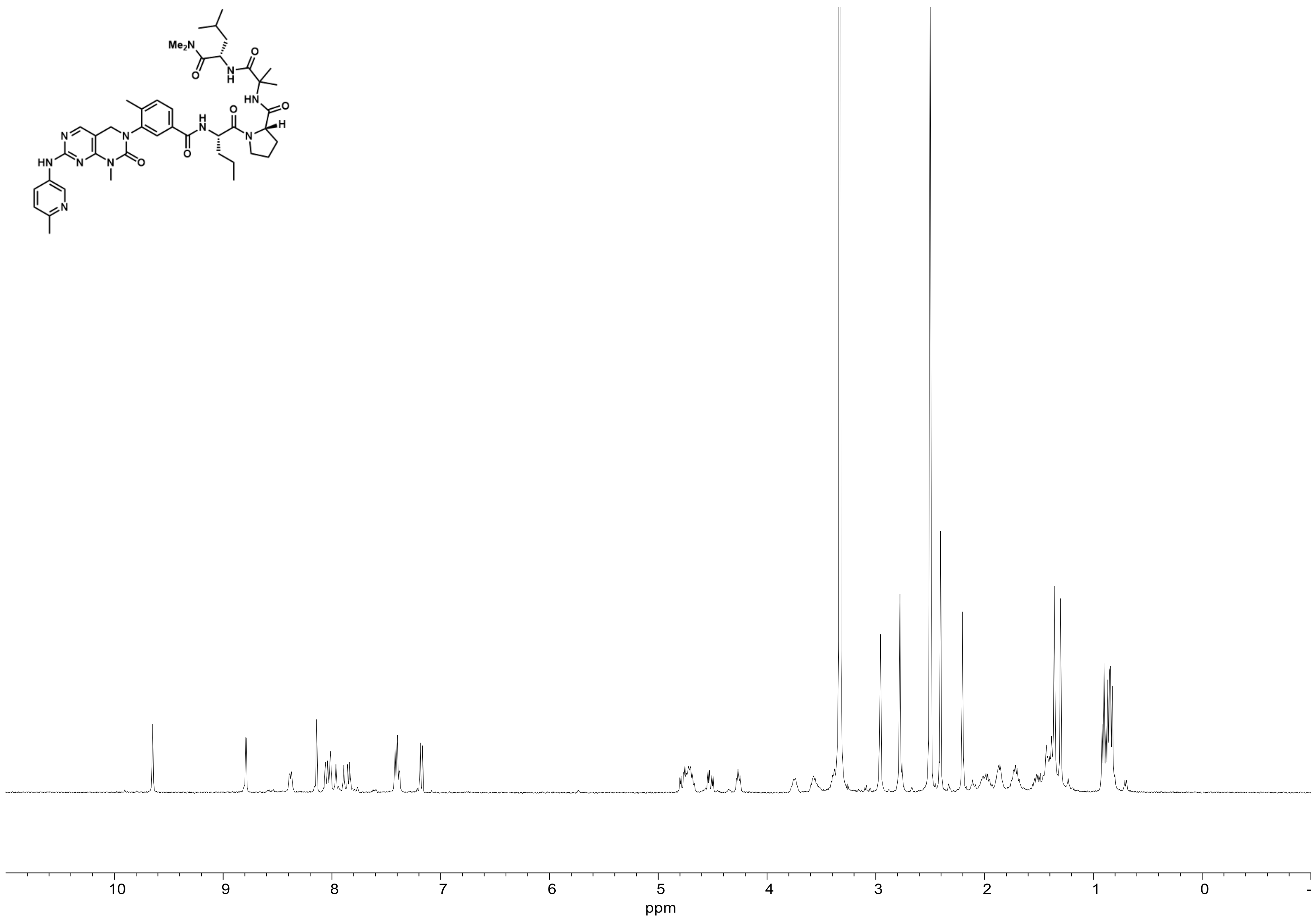

^{1}H NMR (400 MHz, DMSO) of compound **7t**.

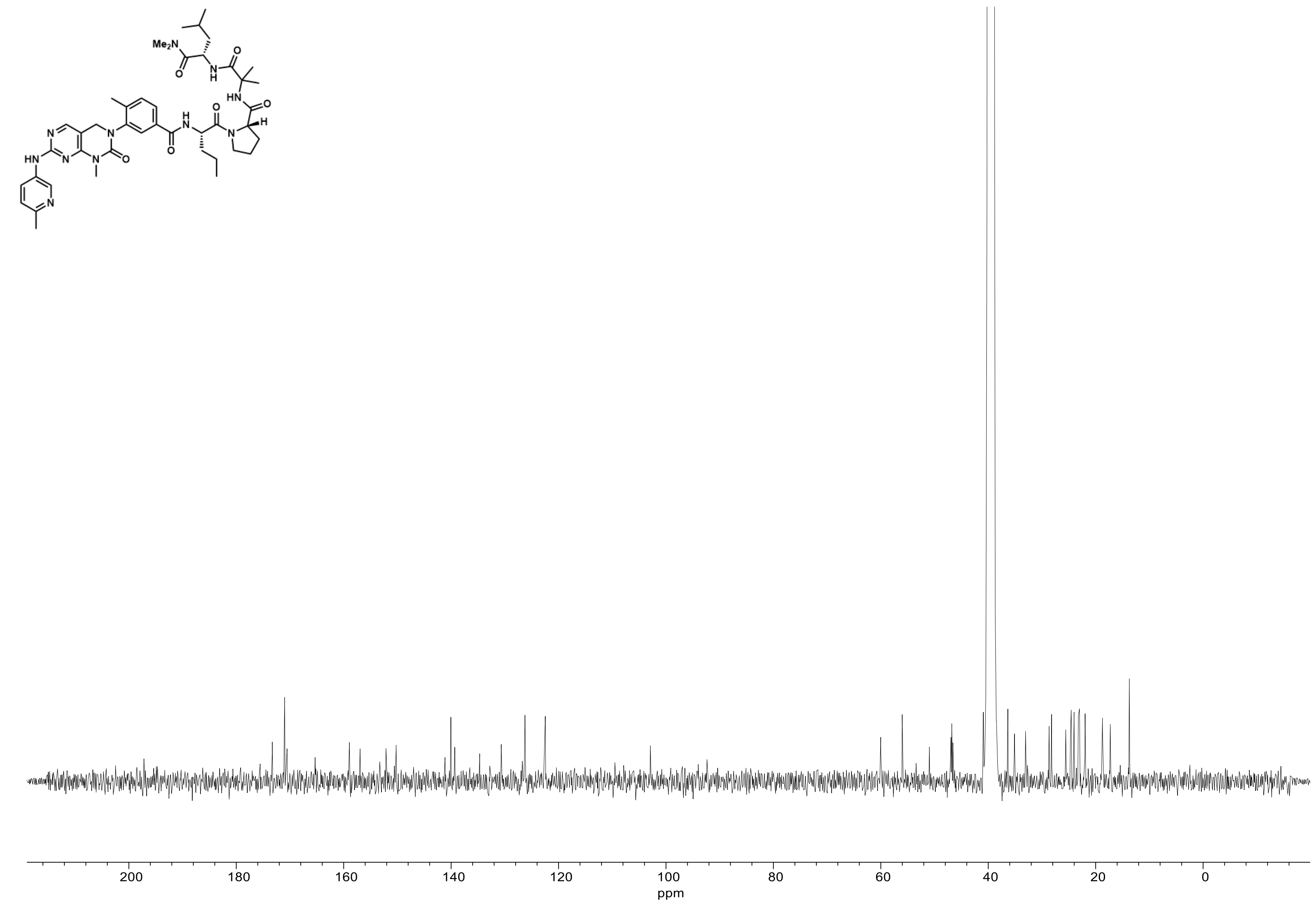

^{13}C NMR (400 MHz, DMSO) of compound **7t**.

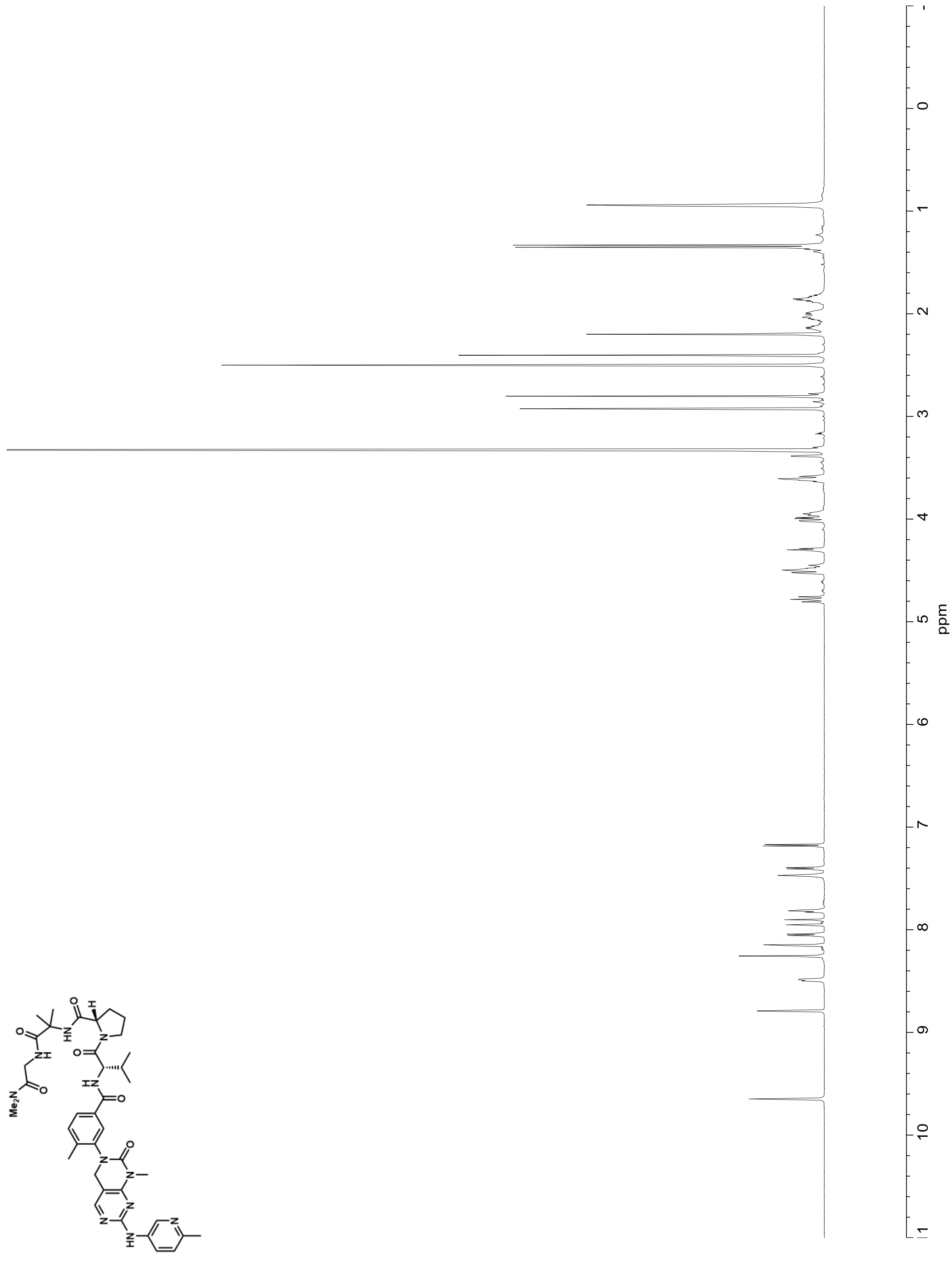

^{1}H NMR (400 MHz, DMSO) of compound **7u**.

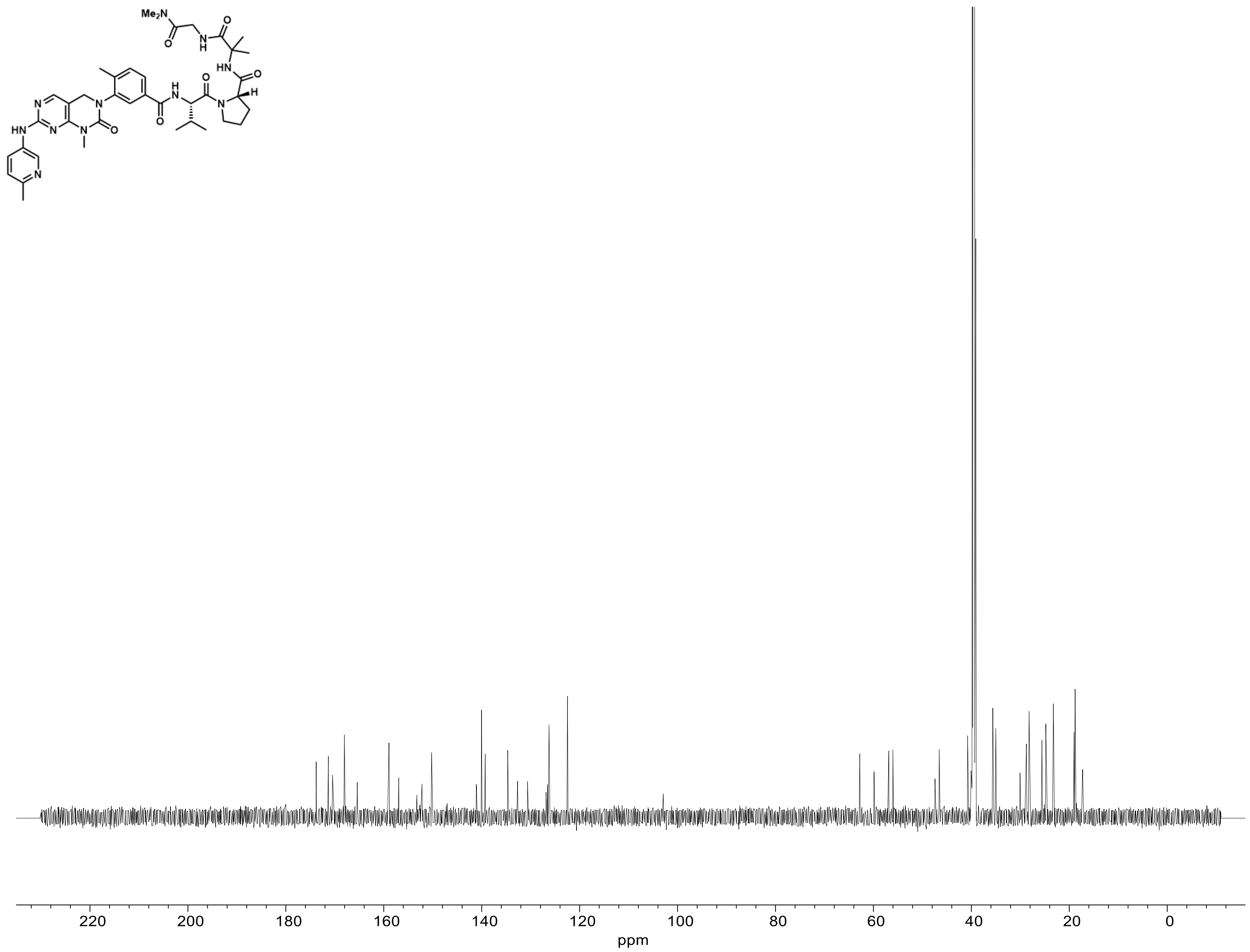

^{13}C NMR (400 MHz, DMSO) of compound **7u**.

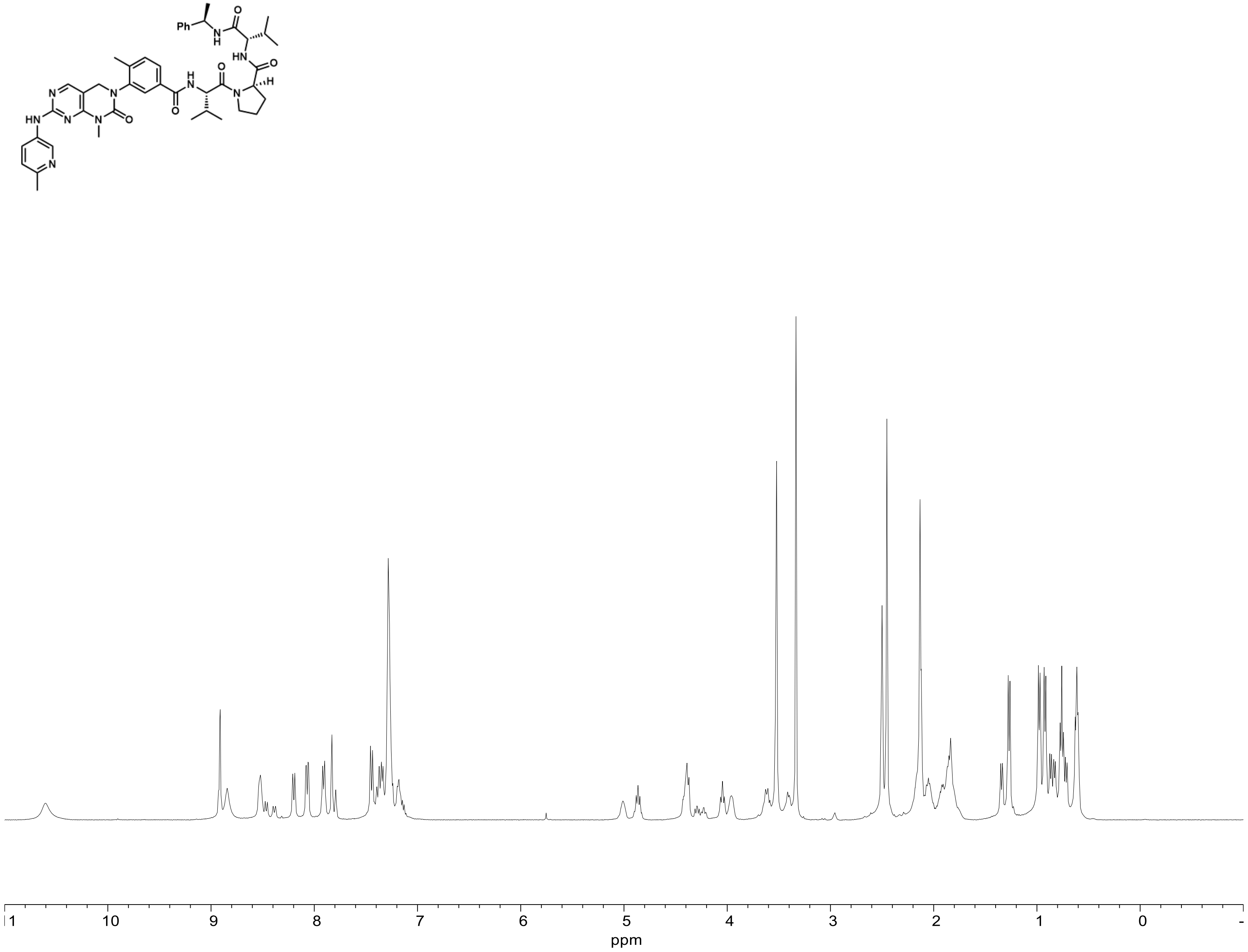

^1H NMR (400 MHz, DMSO) of compound **7v**.

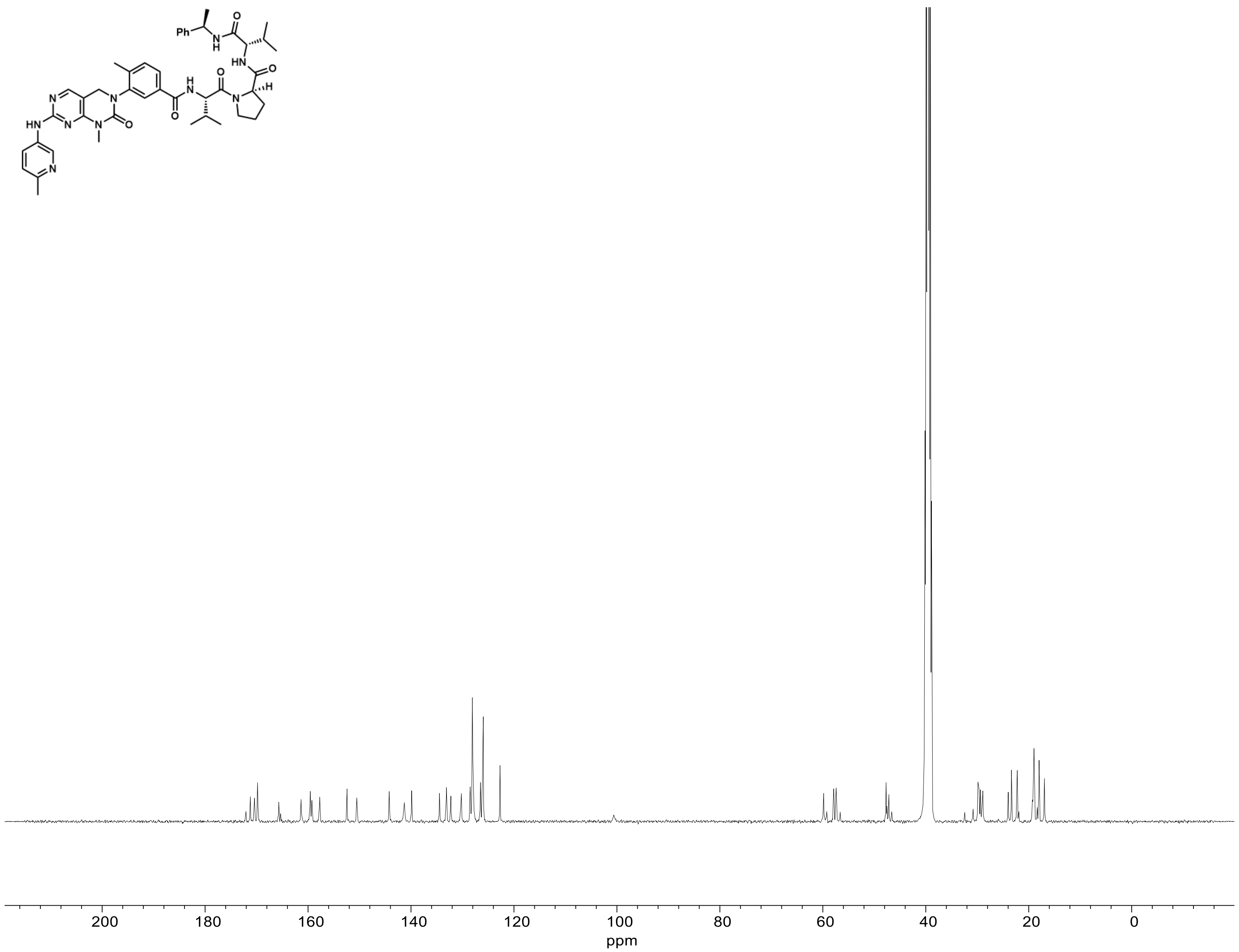

^{13}C NMR (400 MHz, DMSO) of compound **7v**.

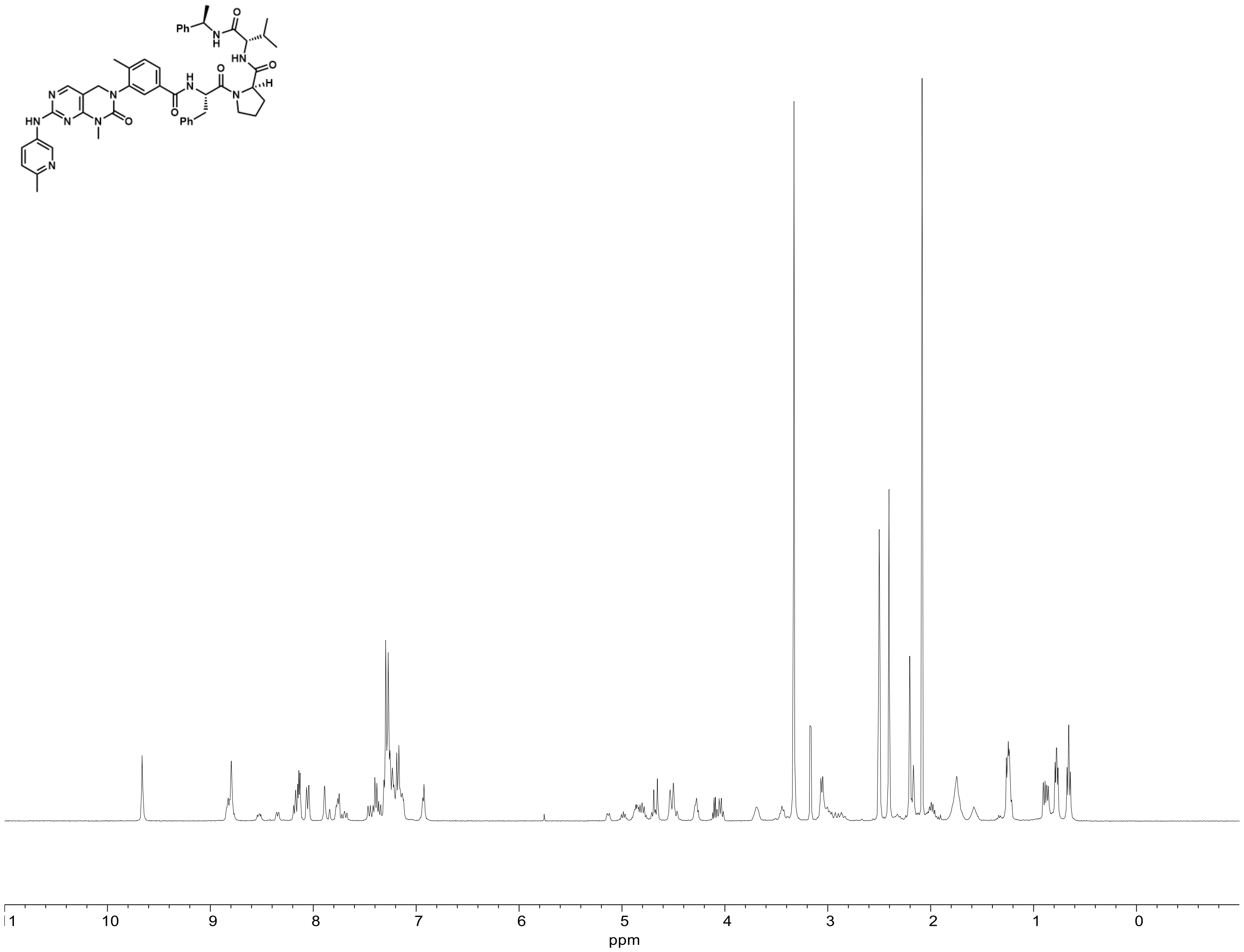

^{1}H NMR (400 MHz, DMSO) of compound **7w**.

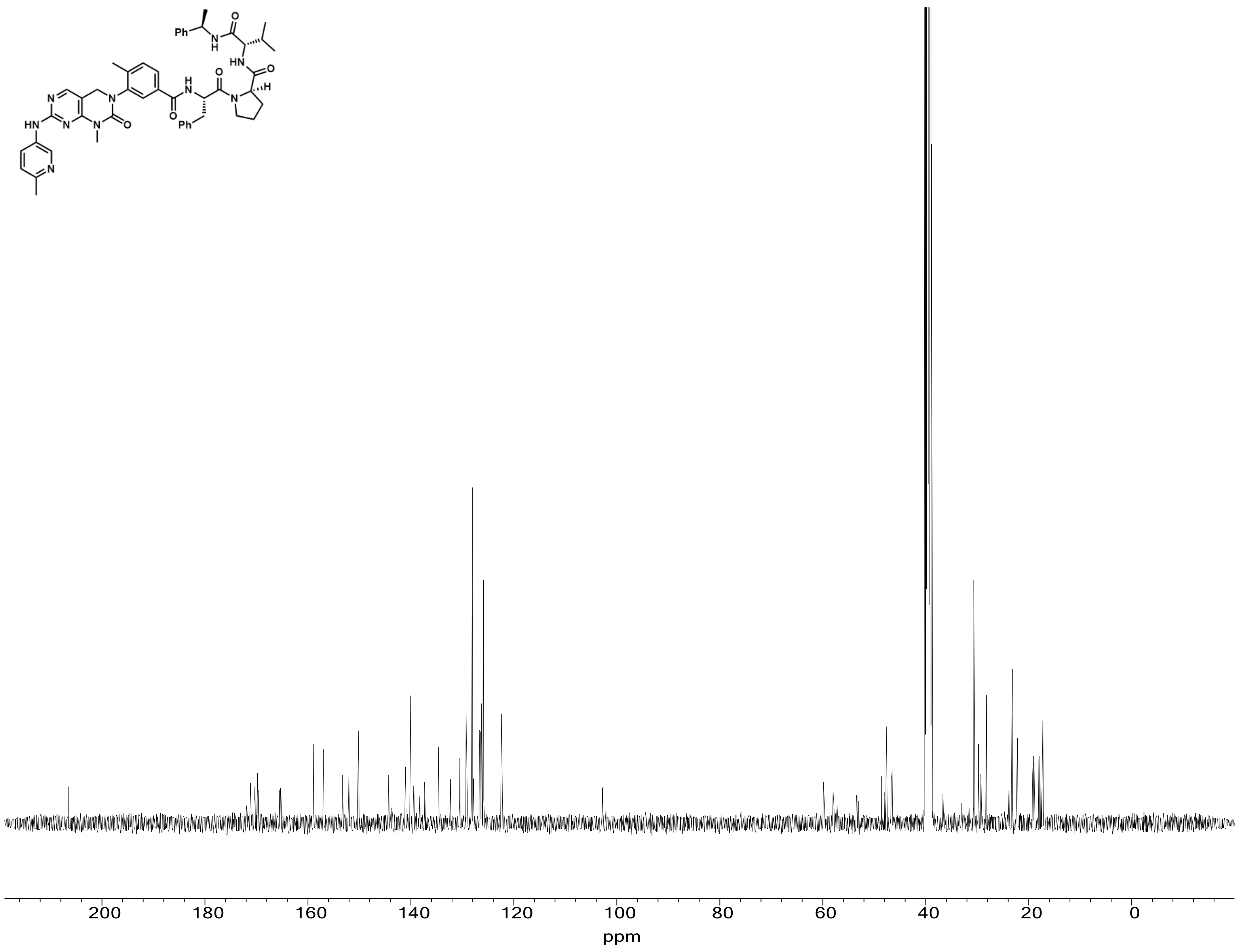

^{13}C NMR (400 MHz, DMSO) of compound **7w**.

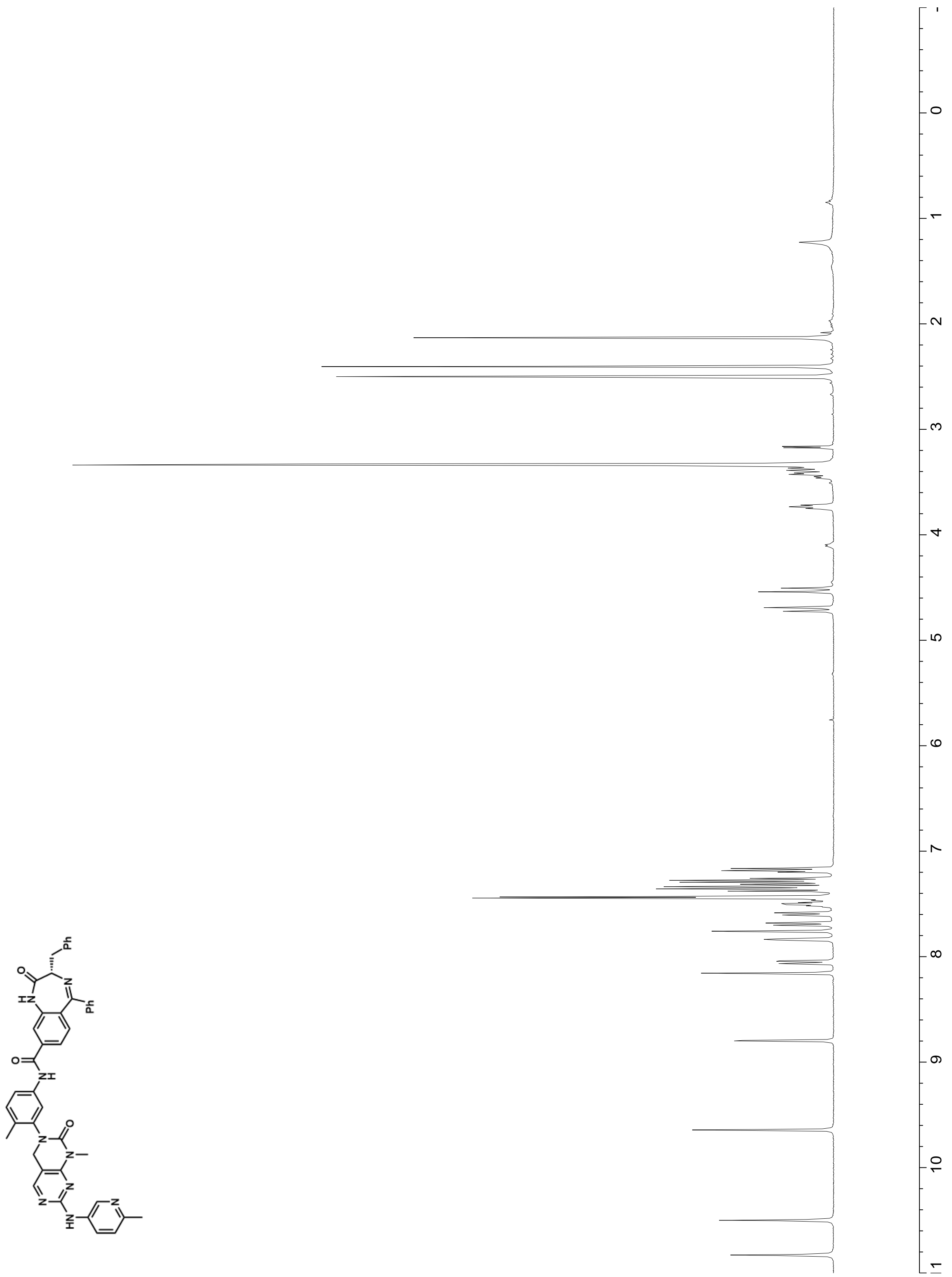

^{1}H NMR (400 MHz, DMSO) of compound **11a**.

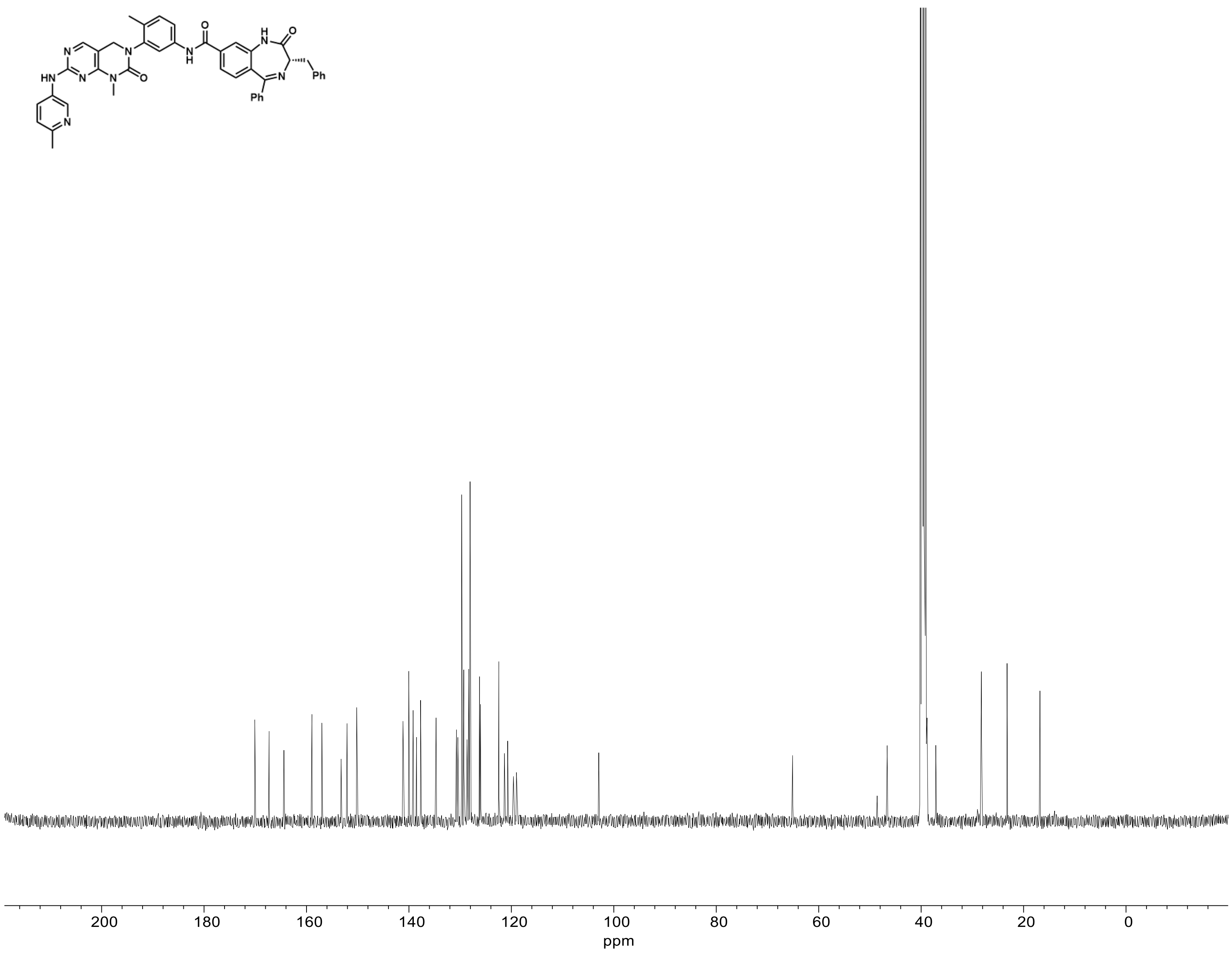

^{13}C NMR (101 MHz, DMSO) of compound **11a**

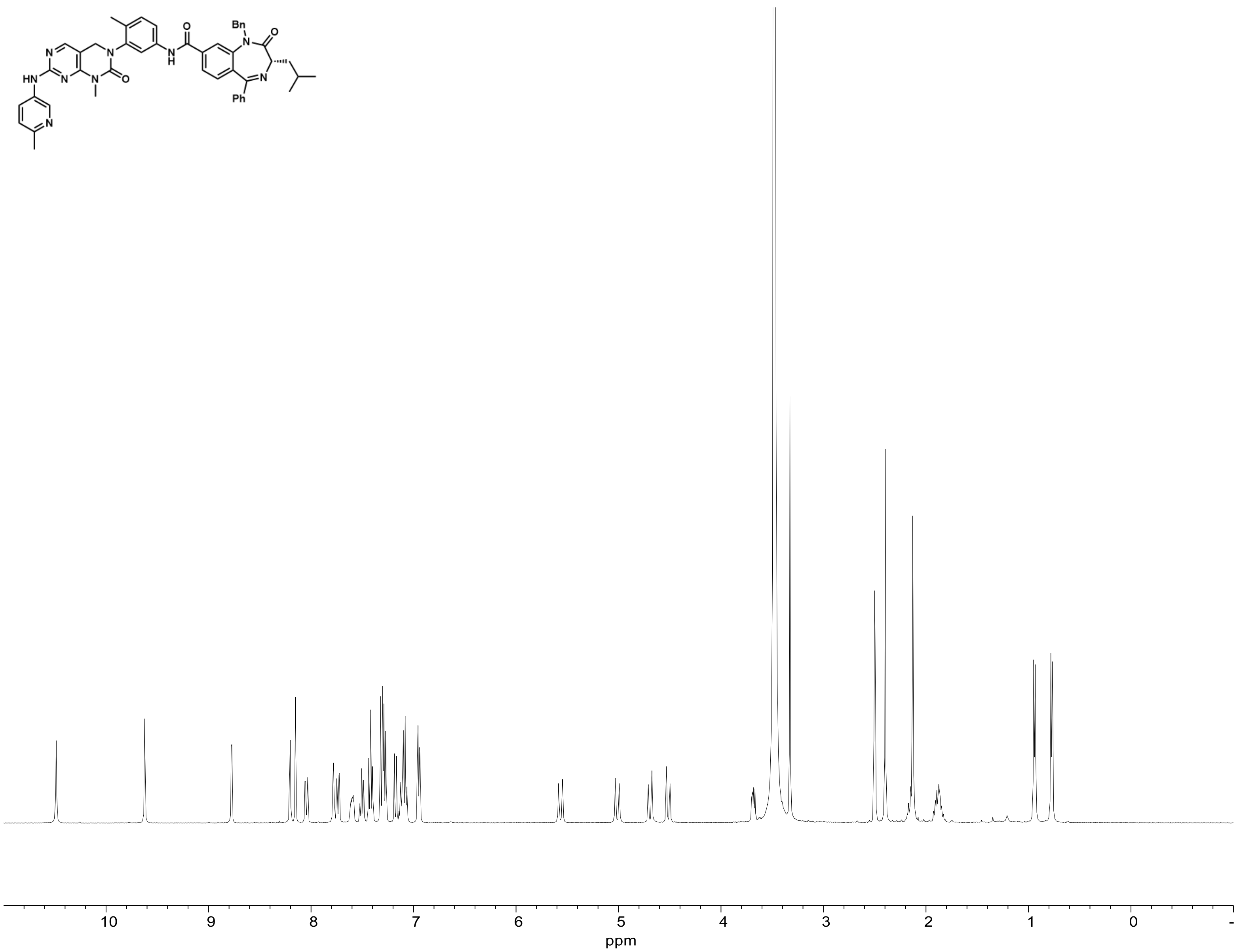

^{1}H NMR (400 MHz, DMSO) of compound **11b**.

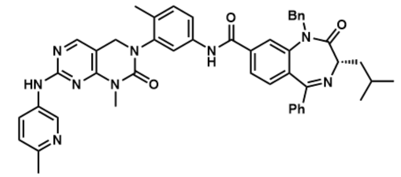

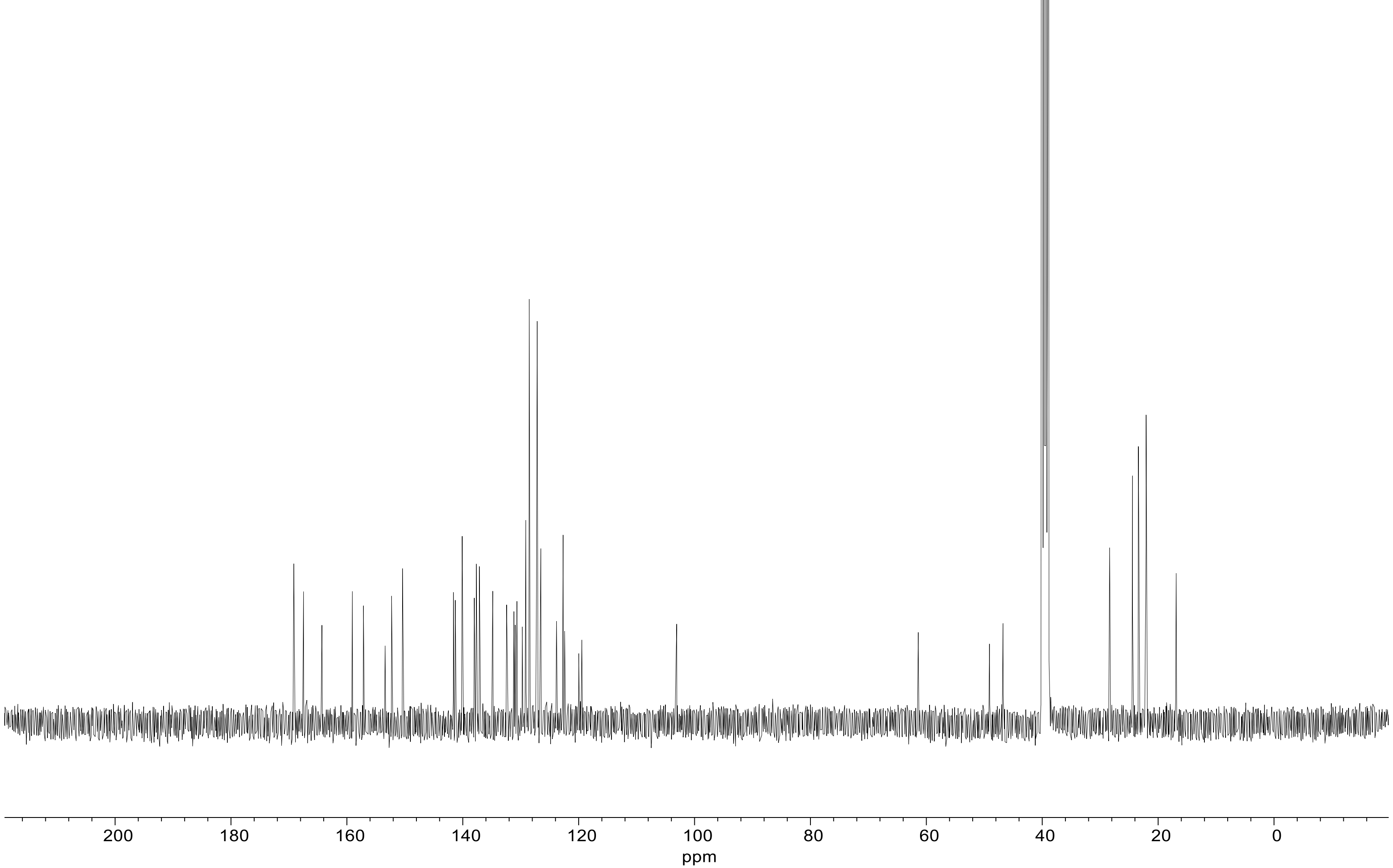

^{13}C NMR (101 MHz, DMSO) of compound **11b**.

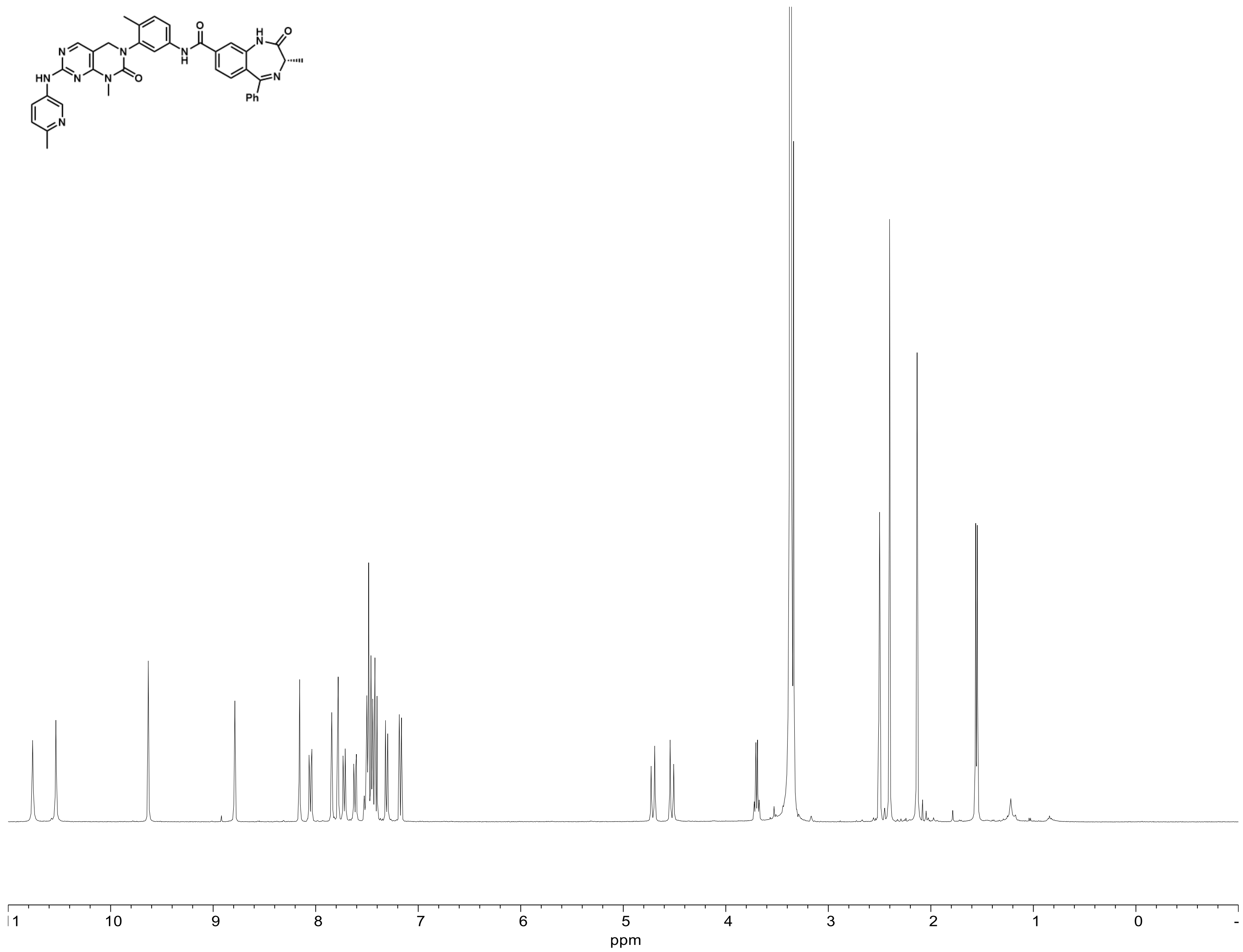

^{1}H NMR (400 MHz, DMSO) of compound **11c**.

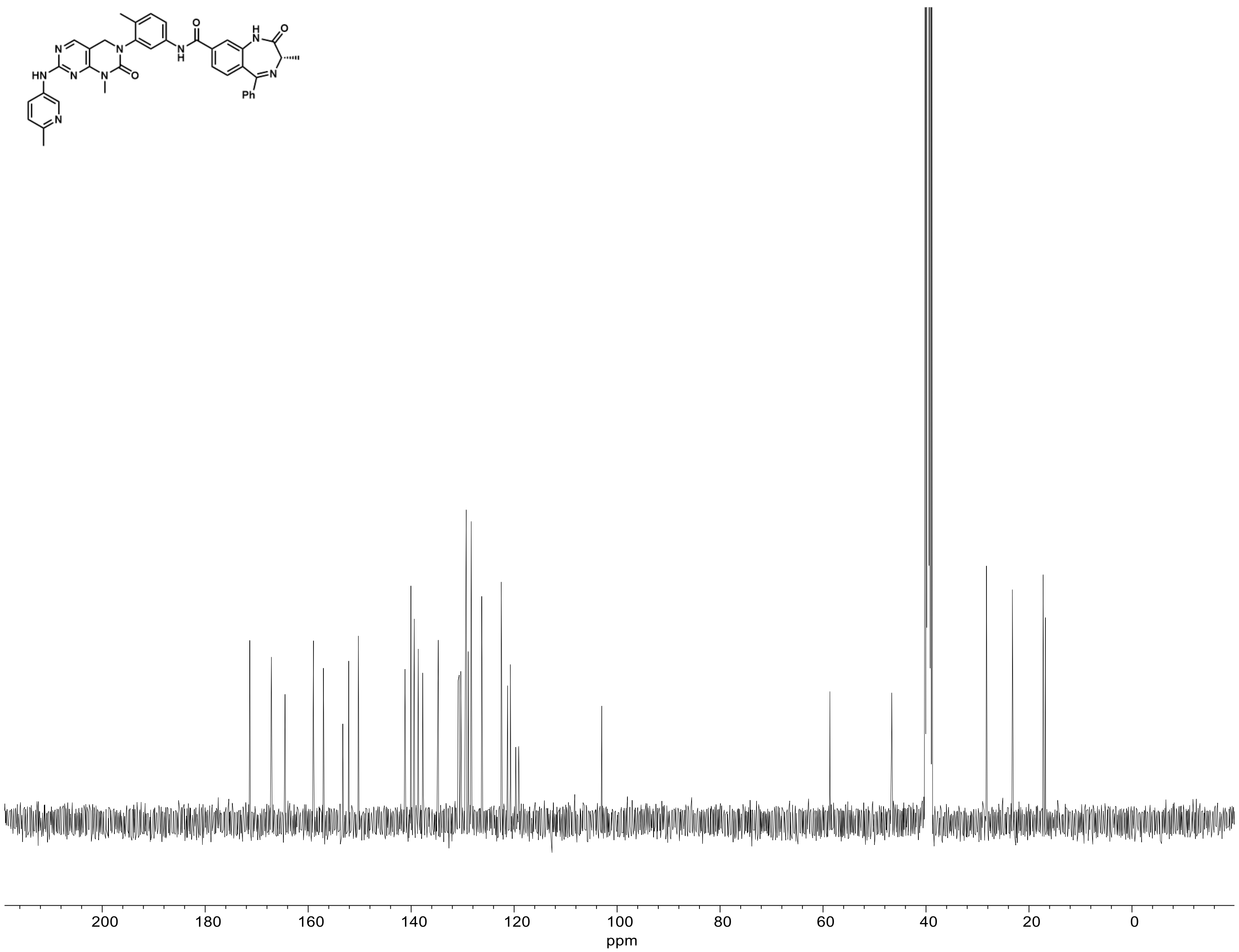

^{13}C NMR (101 MHz, DMSO) of compound **11c.**

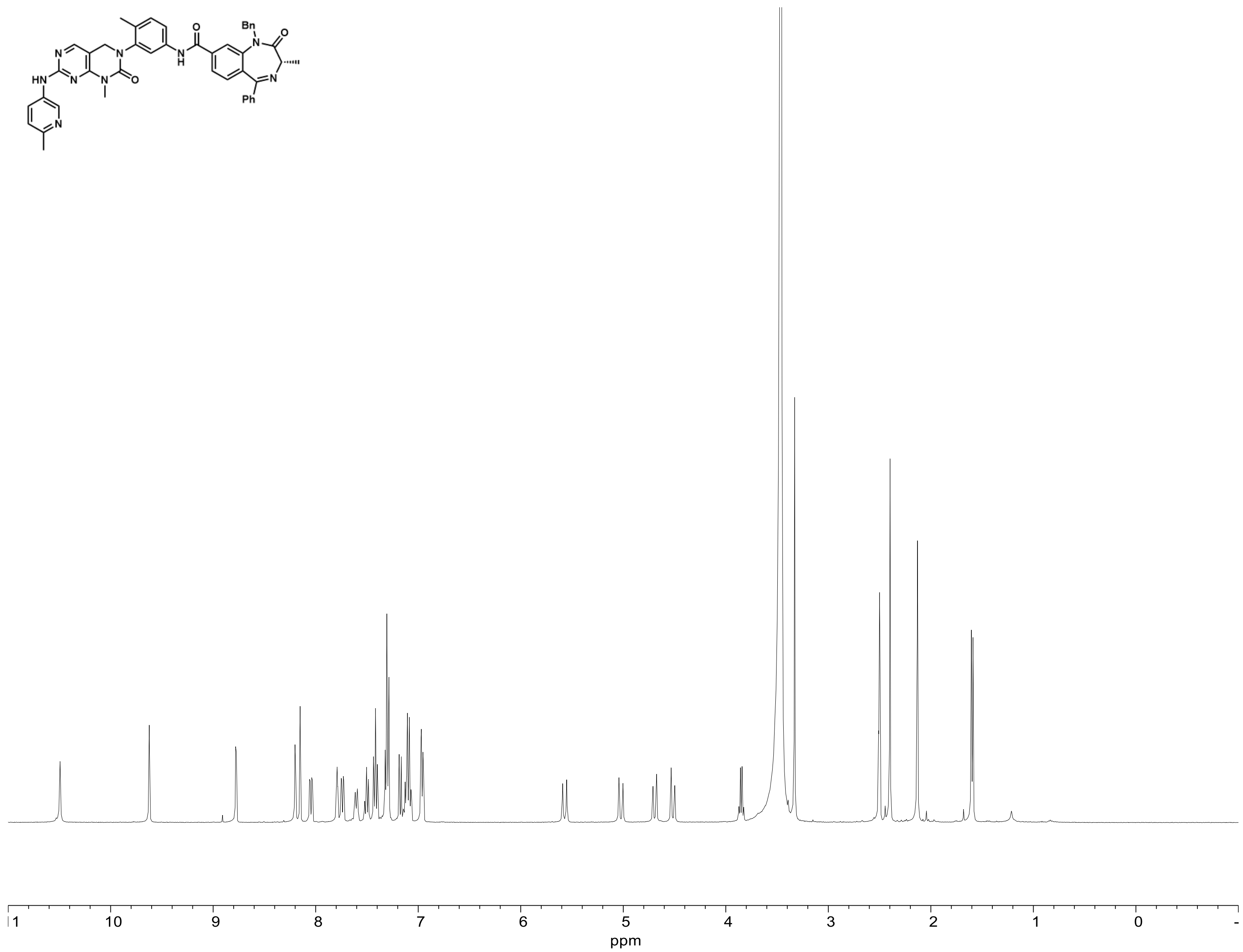

^{1}H NMR (600 MHz, DMSO) of compound **11d**.

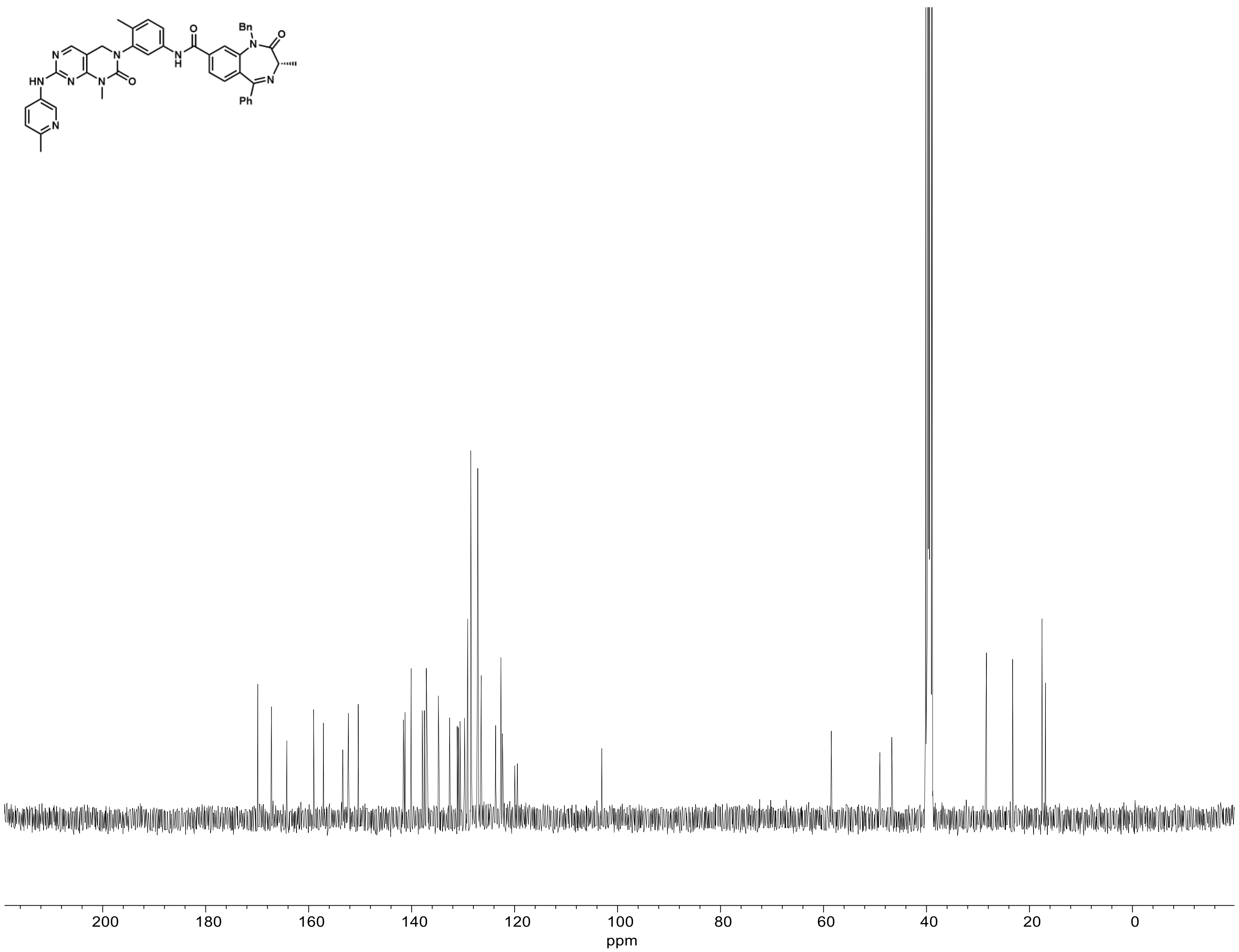

^{13}C NMR (151 MHz, DMSO) of compound **11d.**

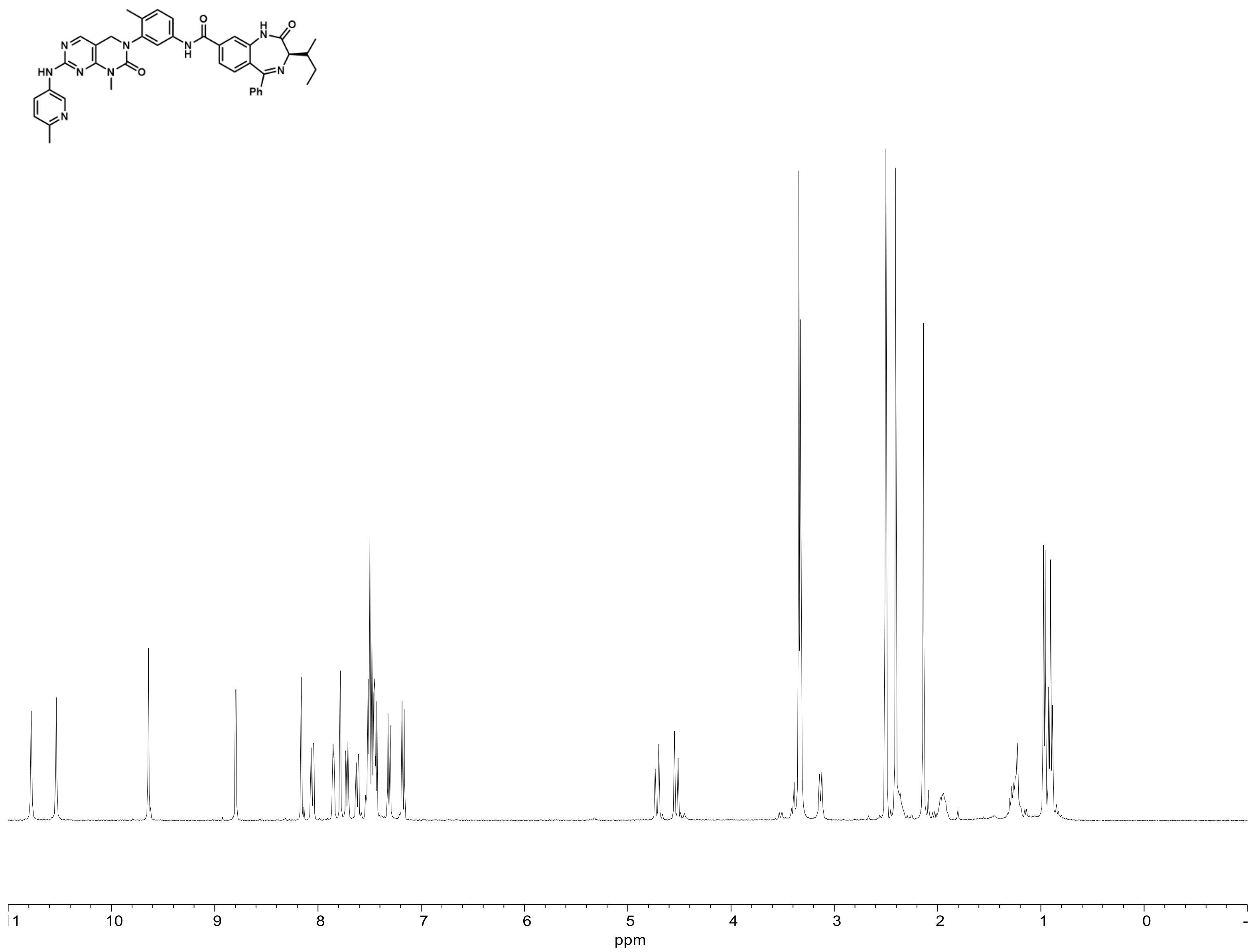

^{1}H NMR (400 MHz, DMSO) of compound **11e**.

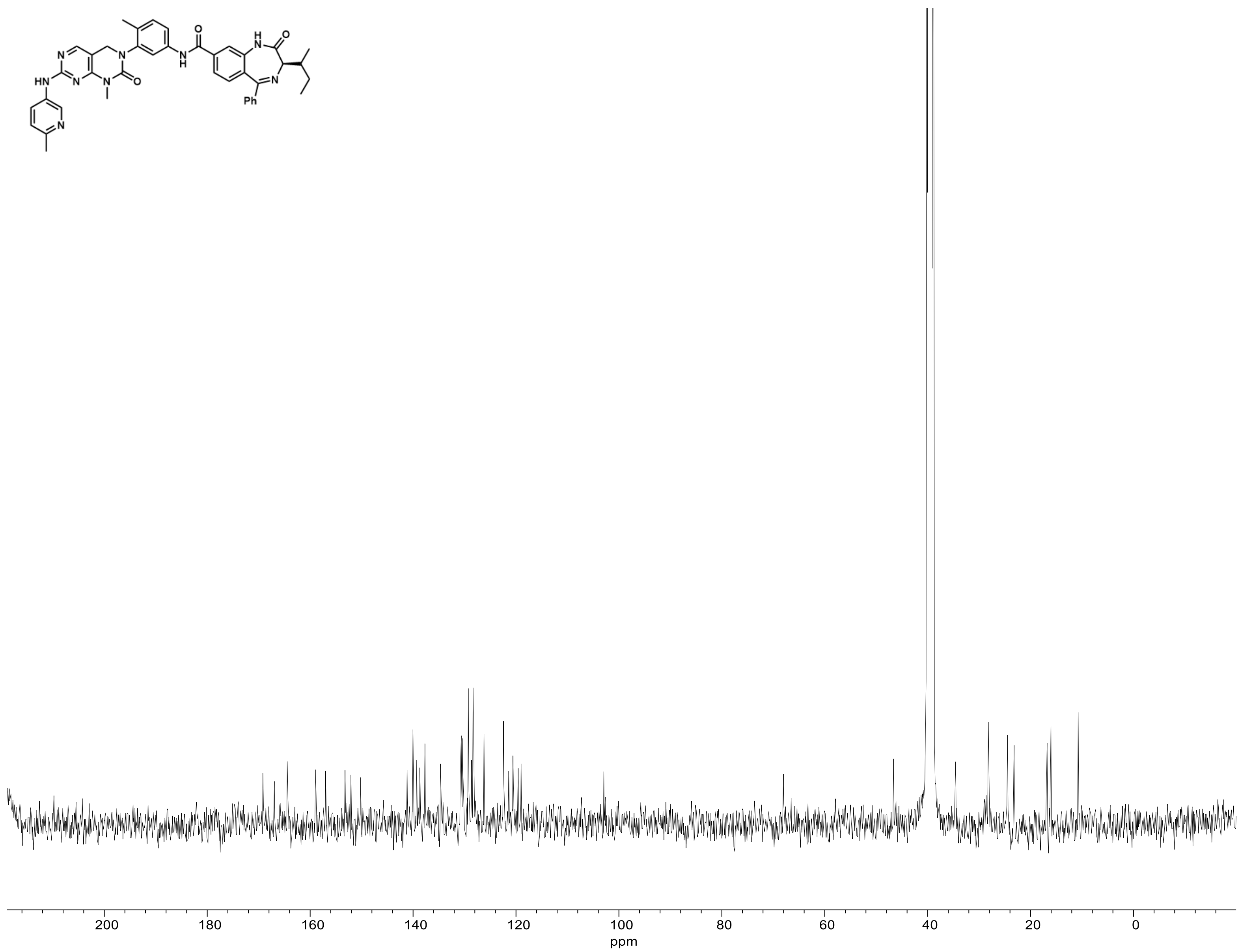

^{13}C NMR (101 MHz, DMSO) of compound **11e.**

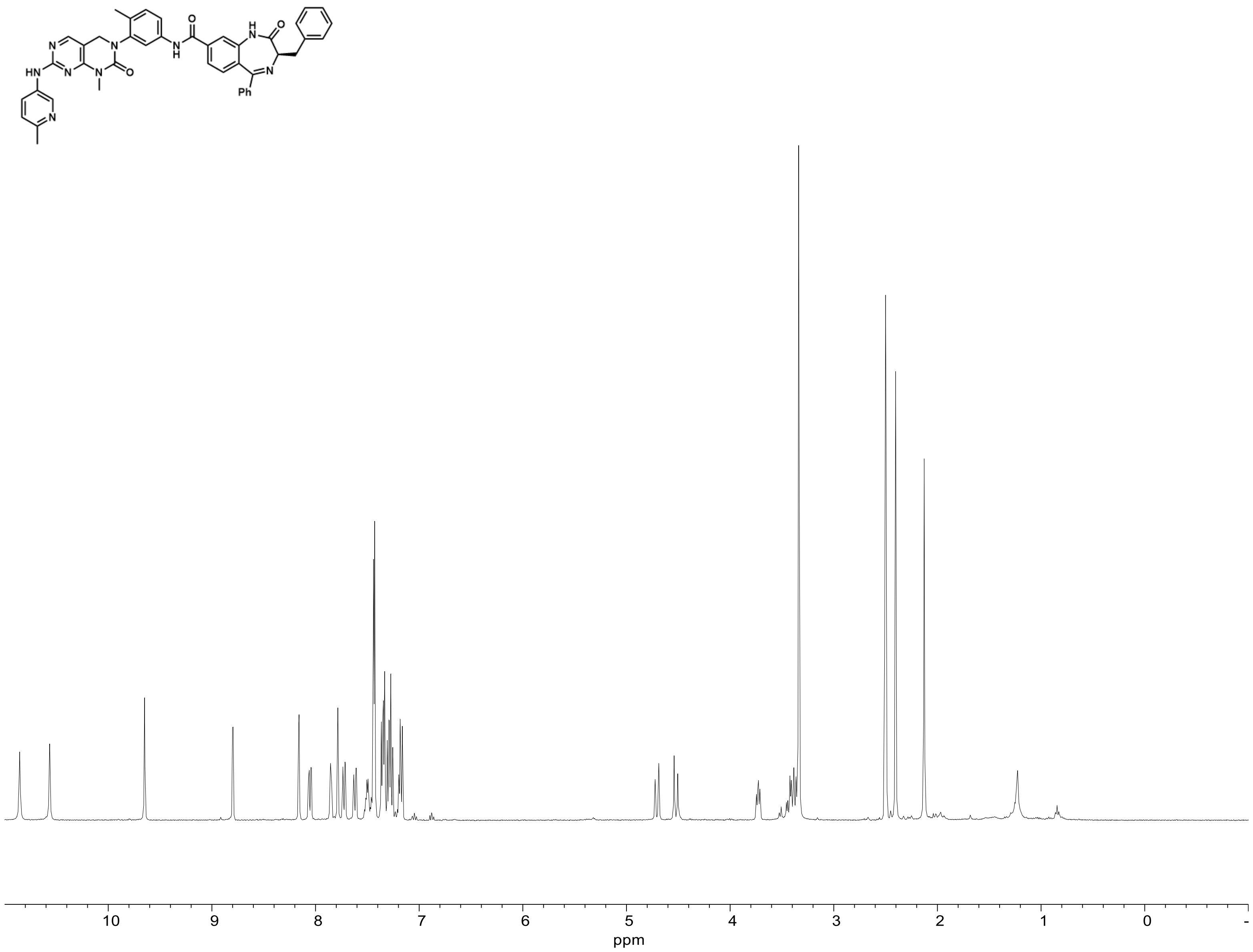

^{1}H NMR (400 MHz, DMSO) of compound **11f**.

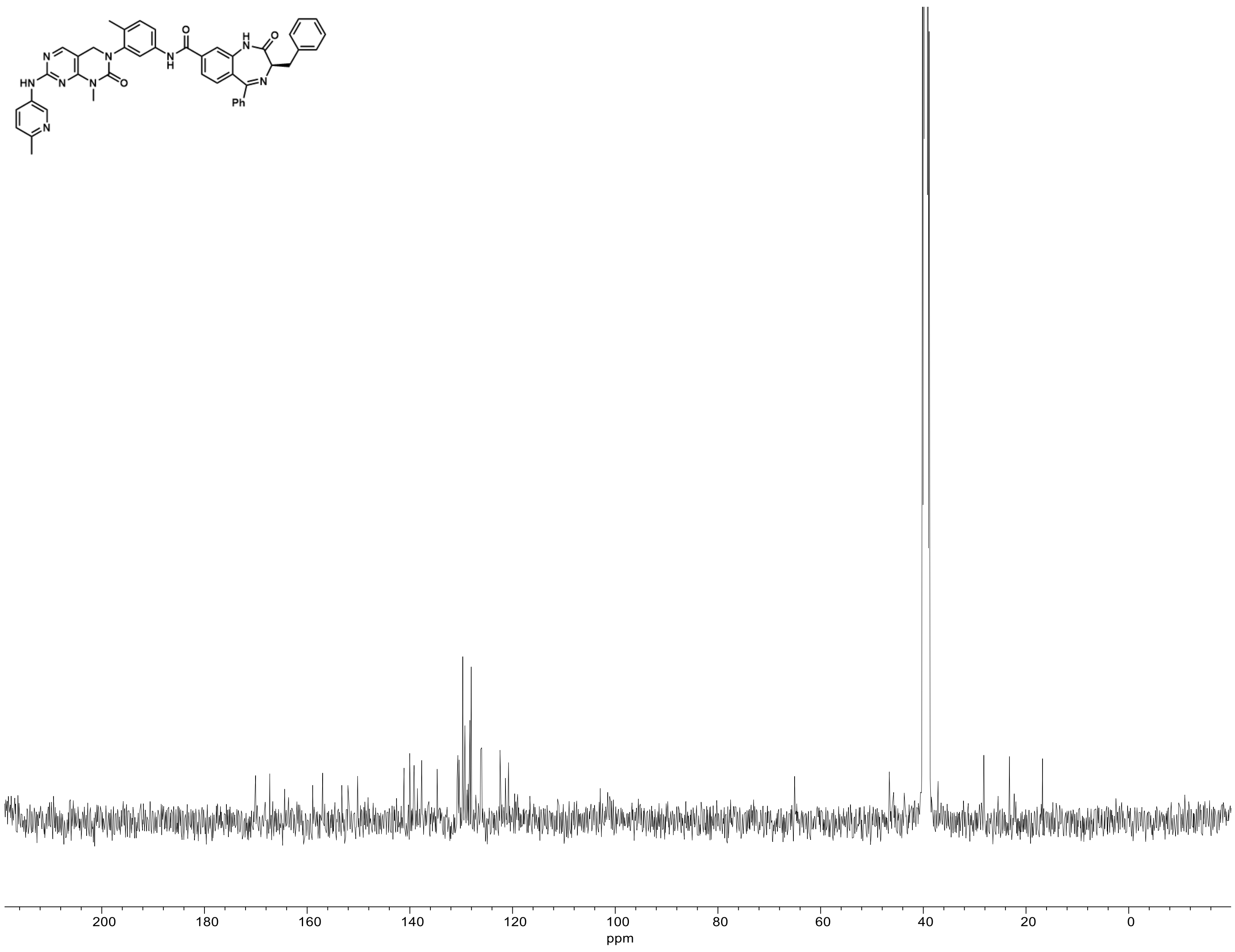

^{13}C NMR (010 MHz, DMSO) of compound **11f.**

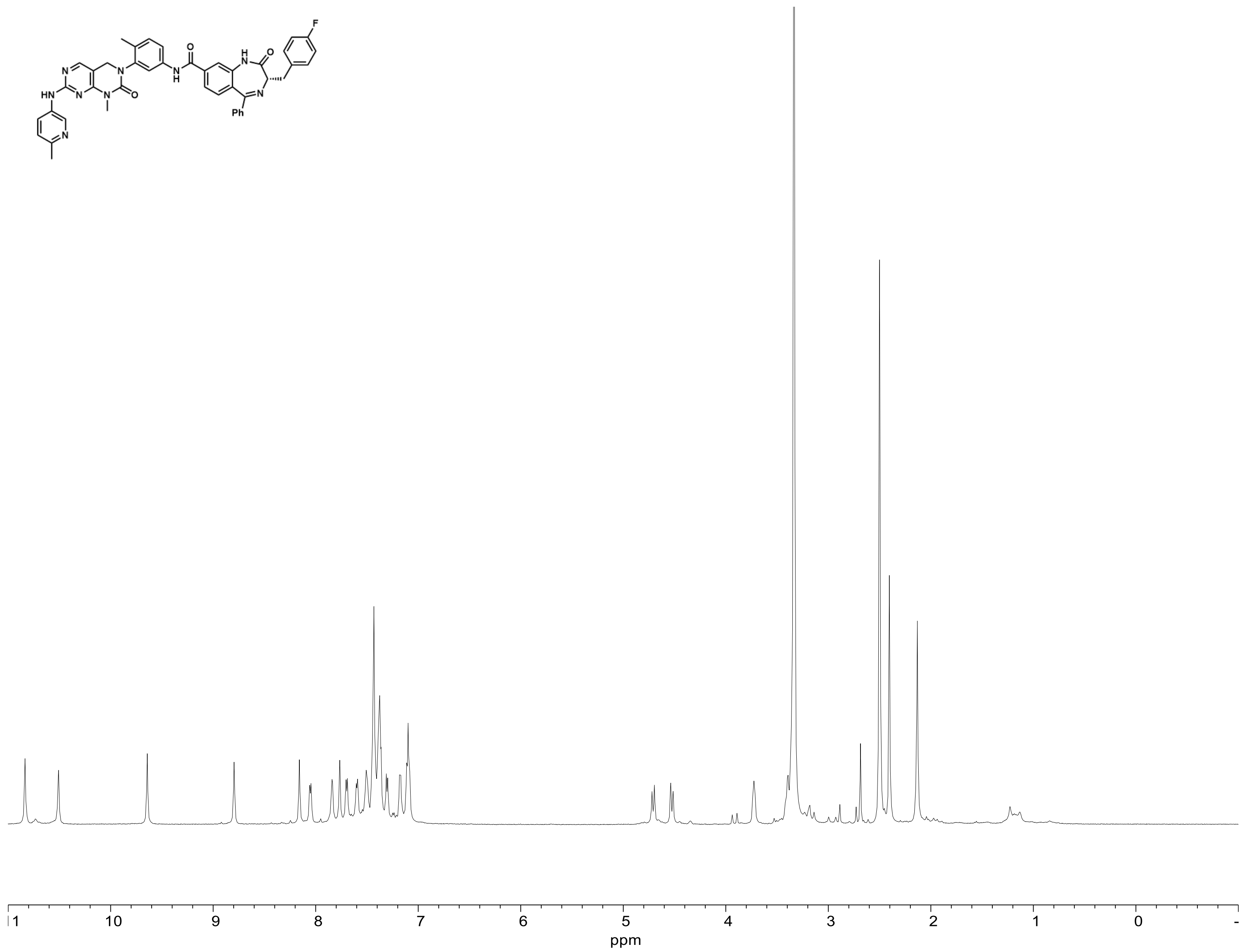

^{1}H NMR (600 MHz, DMSO) of compound **11g**.

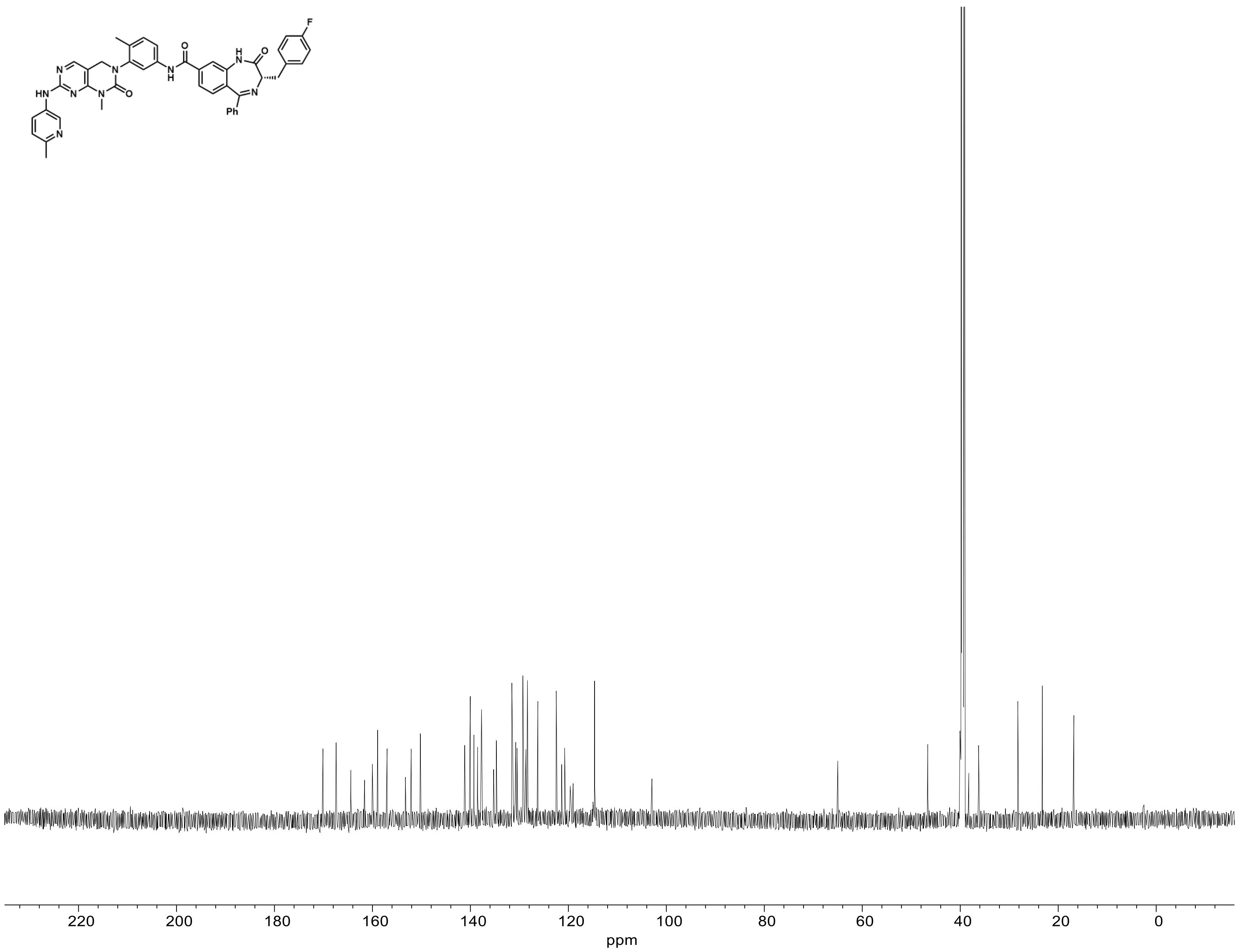

^{13}C NMR (151 MHz, DMSO) of compound **11g.**

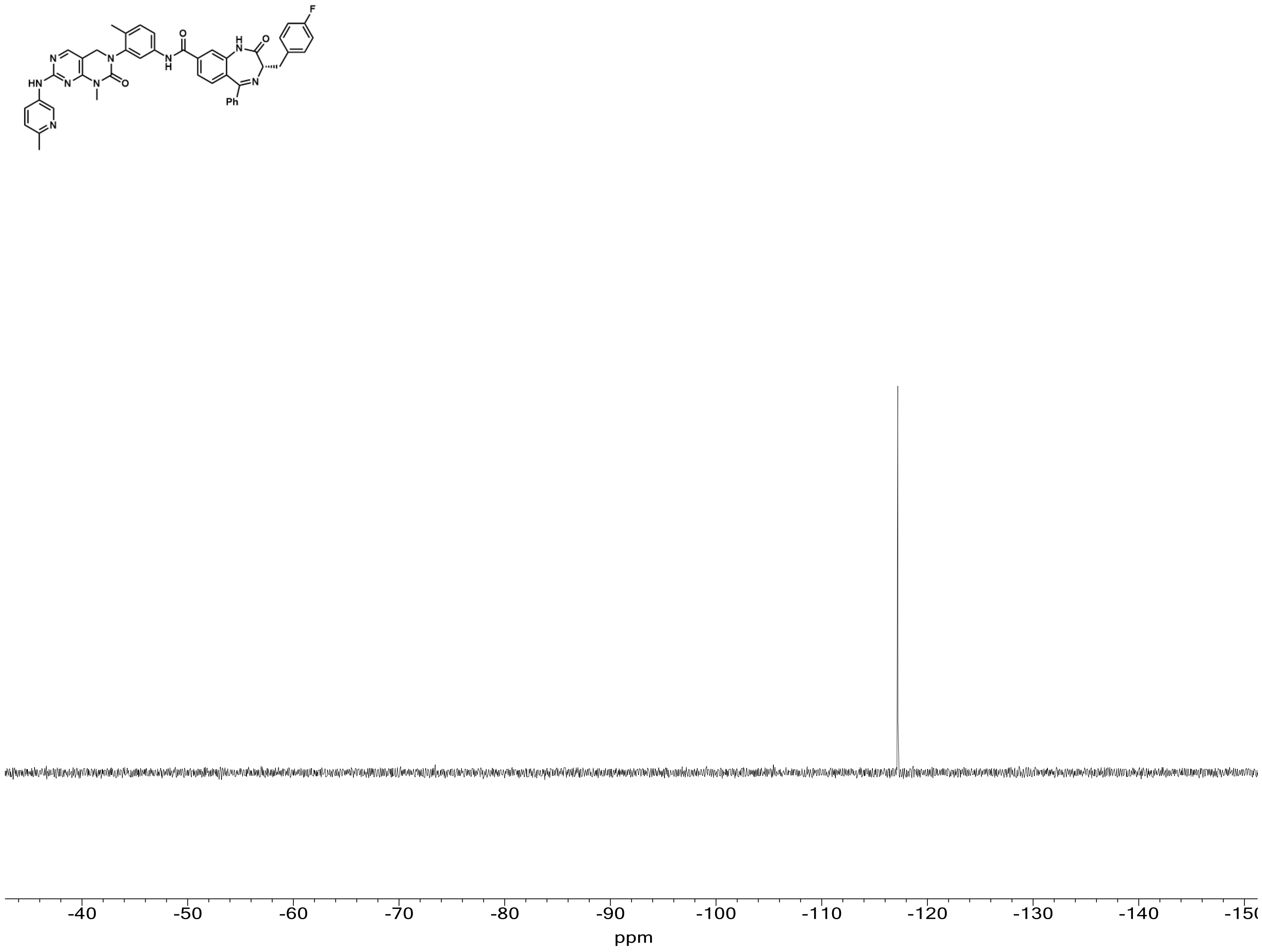

^{19}F NMR (377 MHz, DMSO) of compound **11g.**

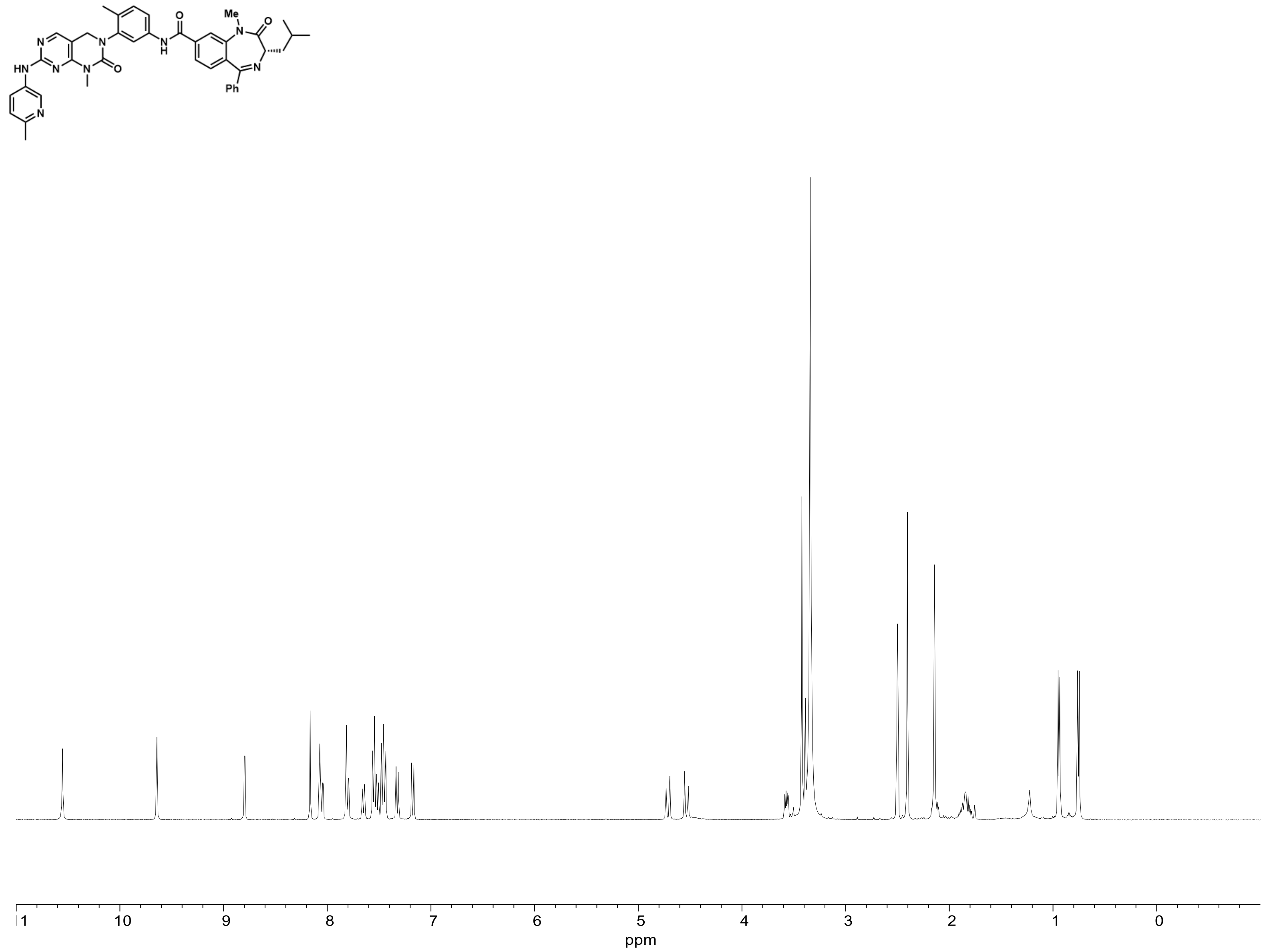

^{1}H NMR (00 MHz, DMSO) of compound **11h**.

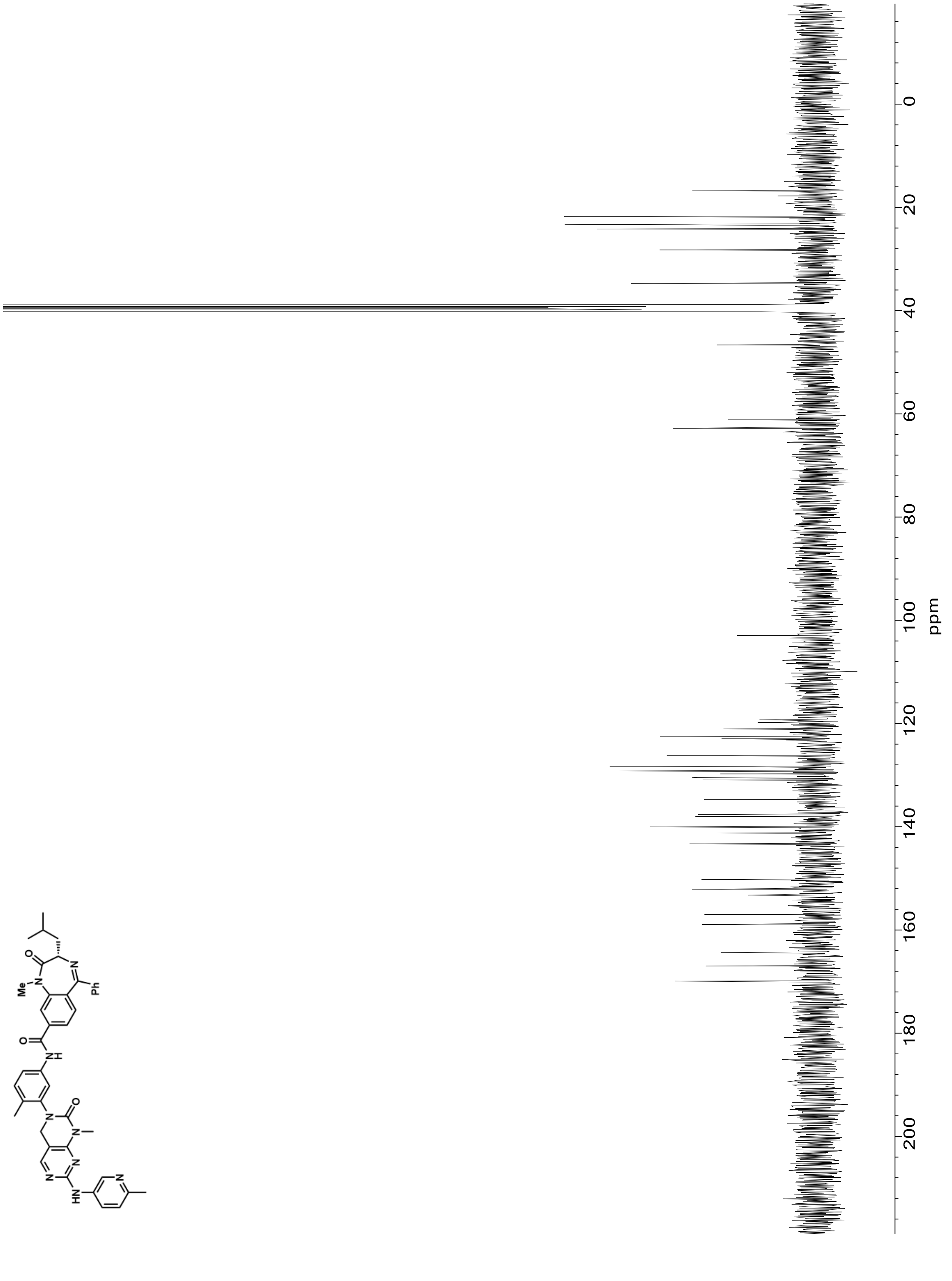

^{13}C NMR (125 MHz, DMSO) of compound **11h.**

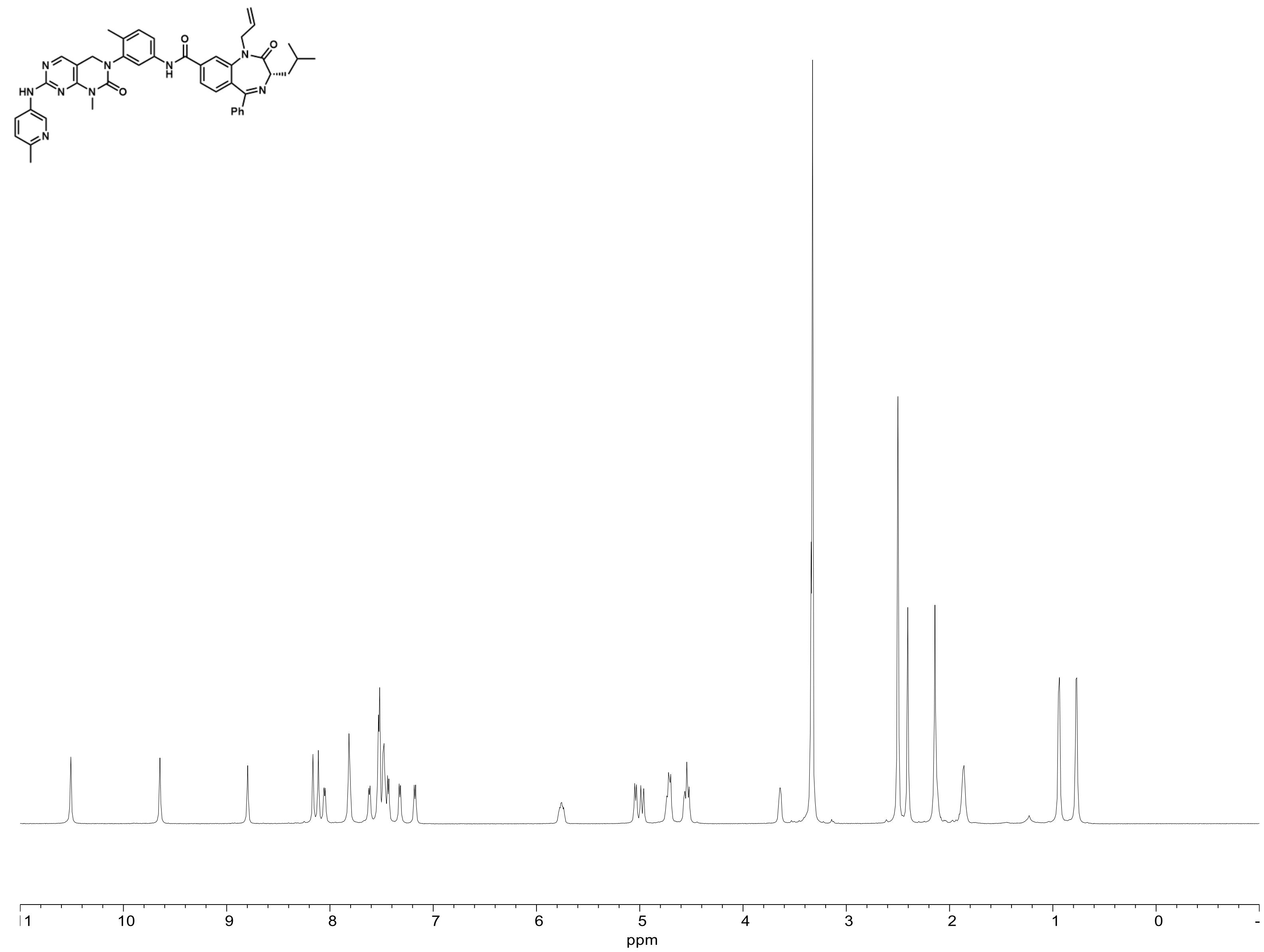

^{1}H NMR (600 MHz, DMSO) of compound **11i**.

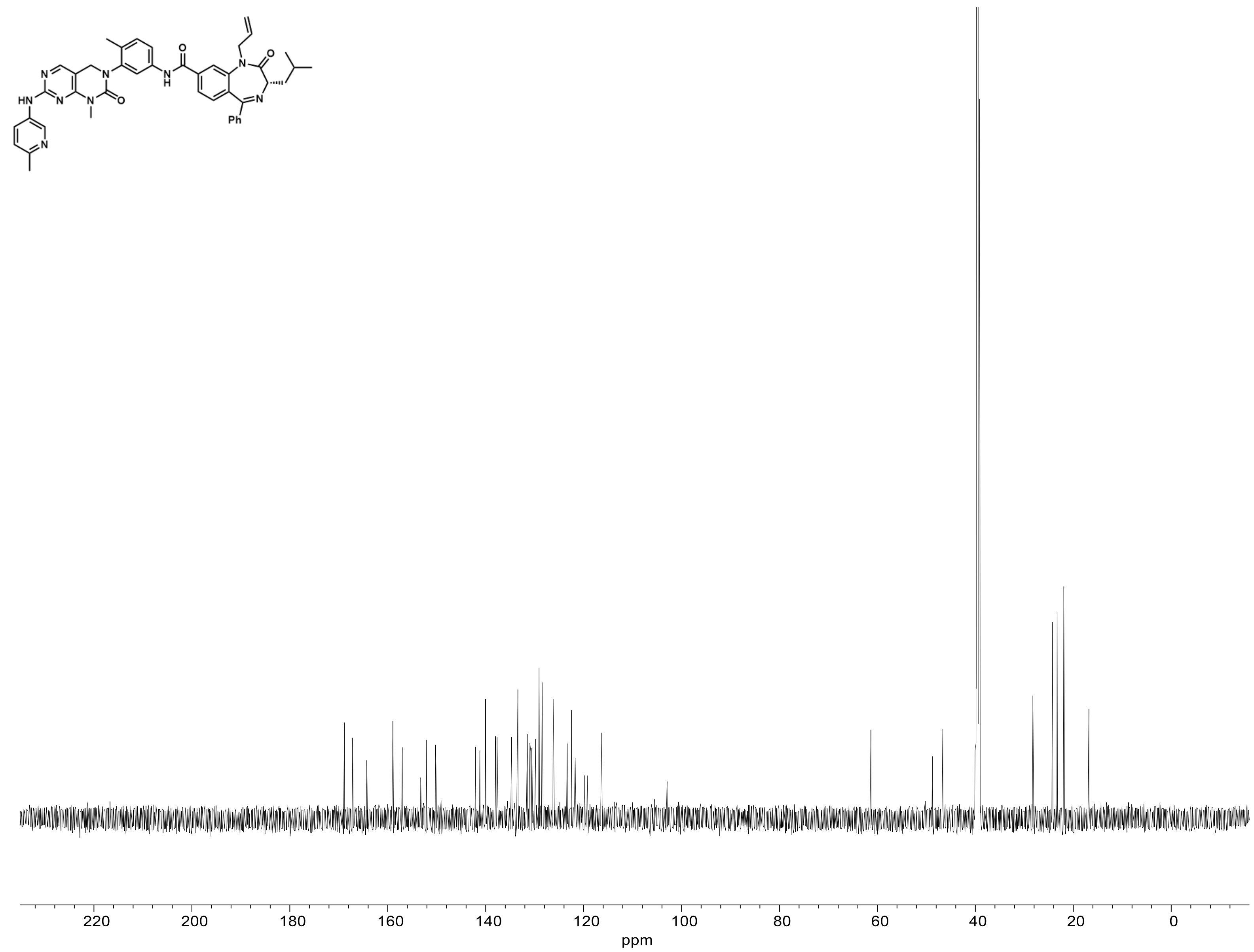

^{13}C NMR (151 MHz, DMSO) of compound **11i.**

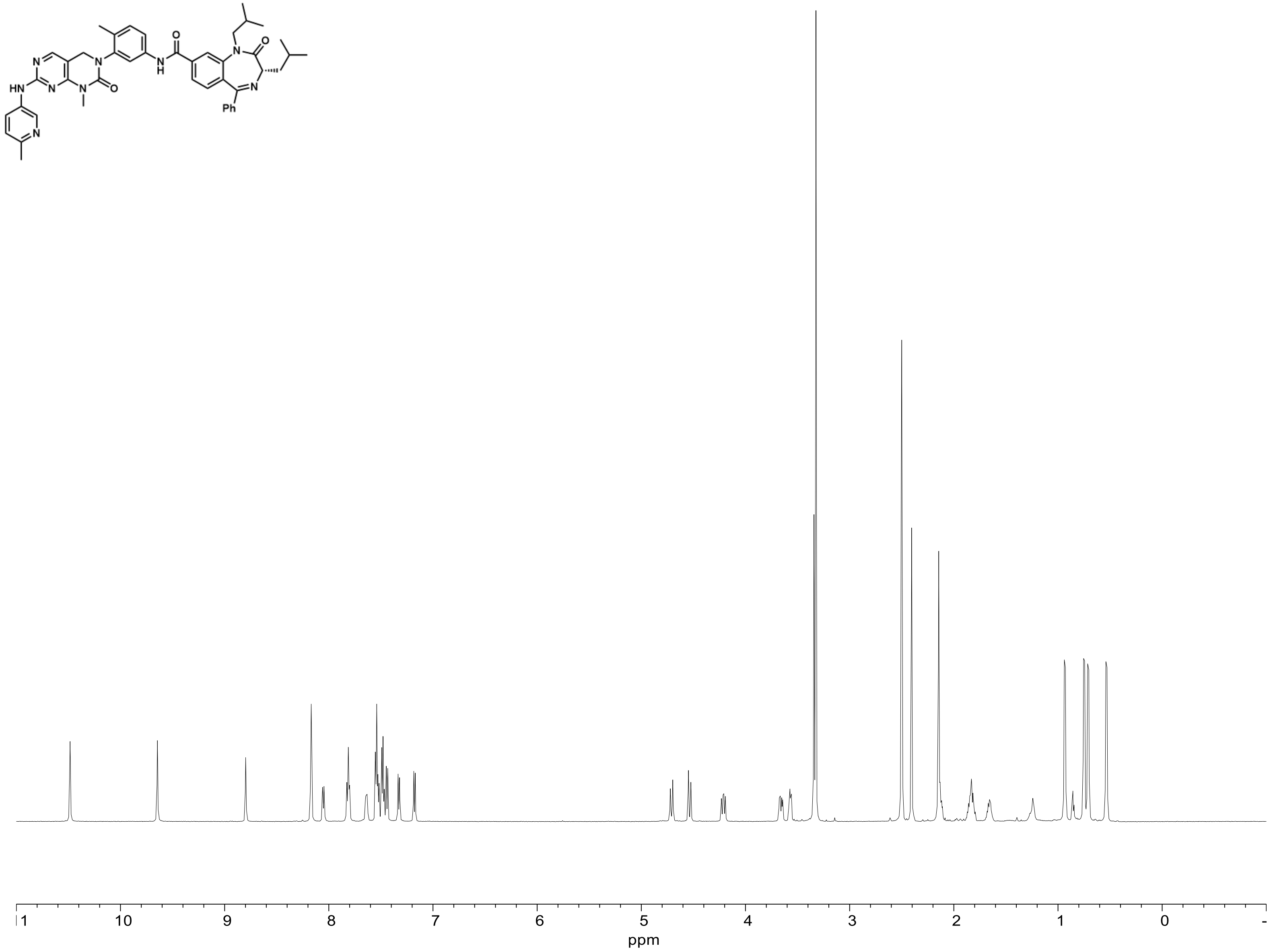

^{1}H NMR (600 MHz, DMSO) of compound **11j**.

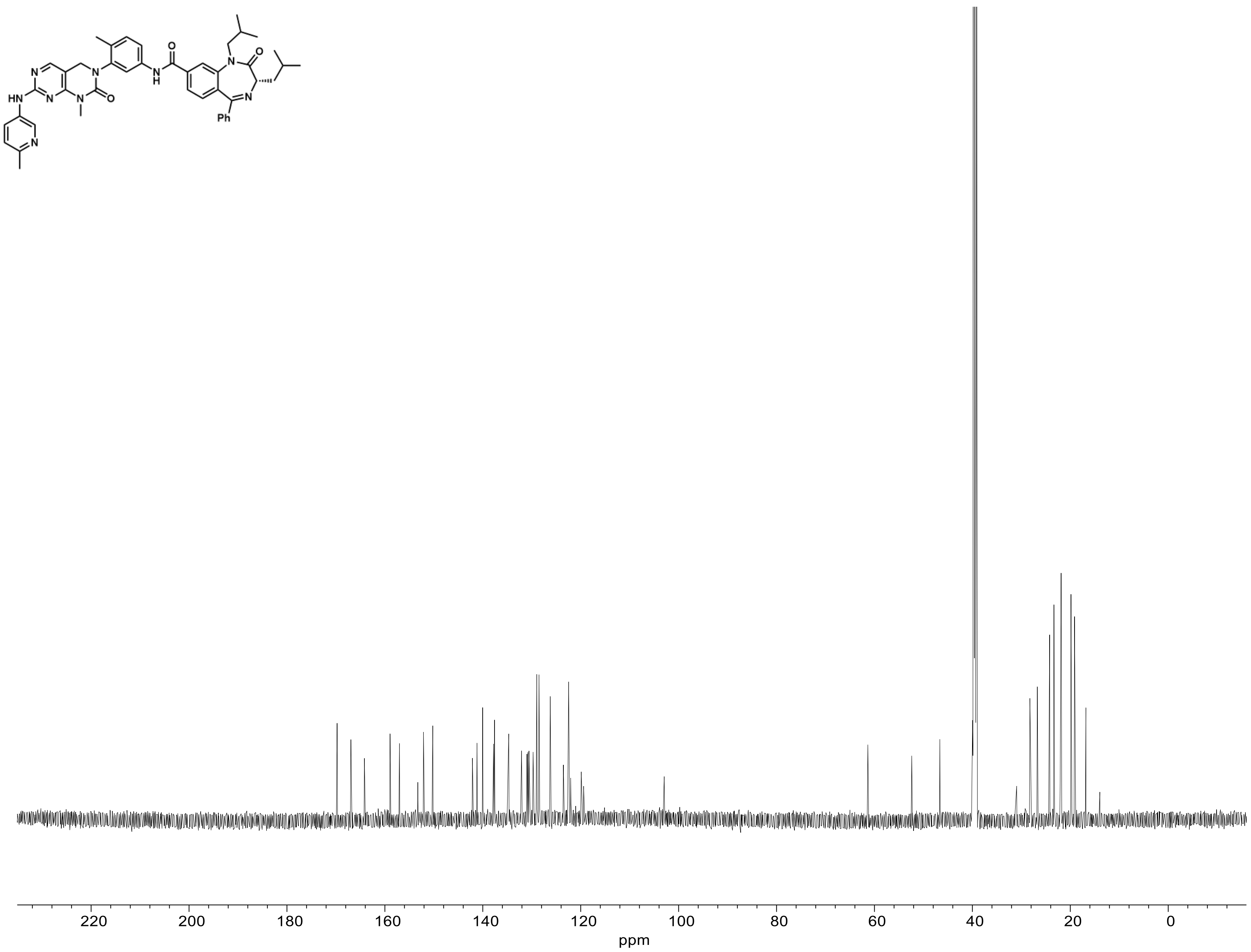

^{13}C NMR (151 MHz, DMSO) of compound **11j.**

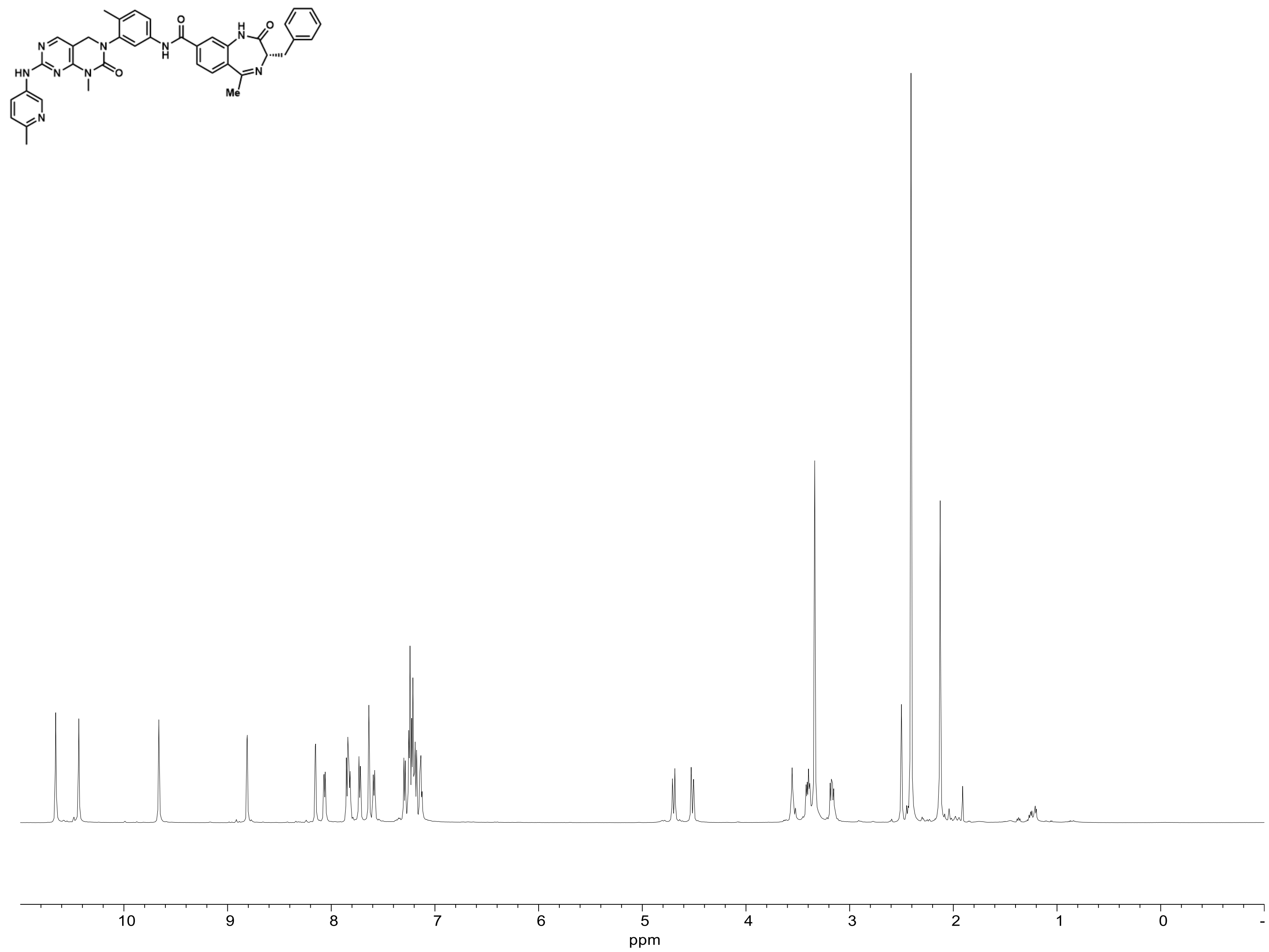

^{1}H NMR (600 MHz, DMSO) of compound **11k**.

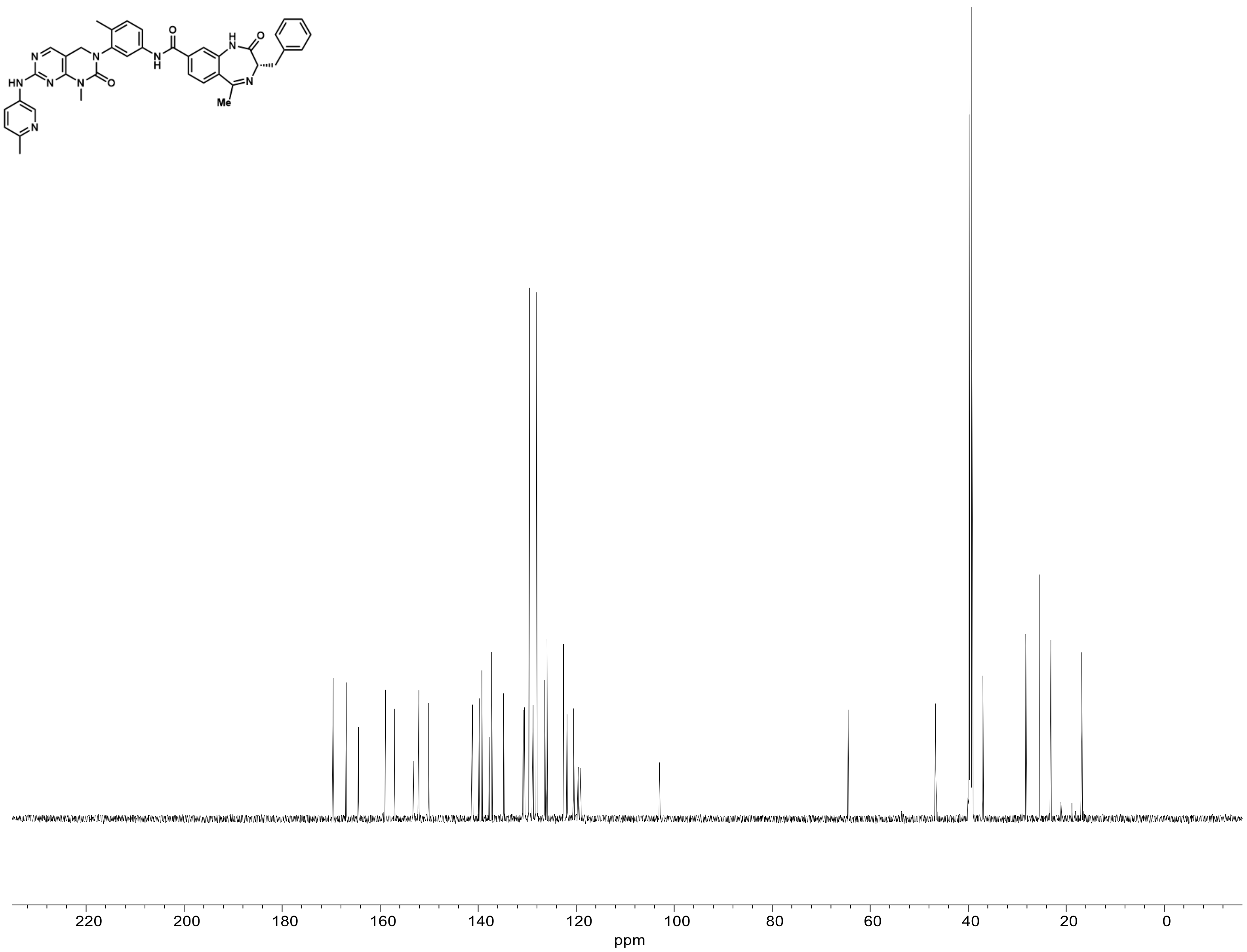

^{13}C NMR (151 MHz, DMSO) of compound **11k.**

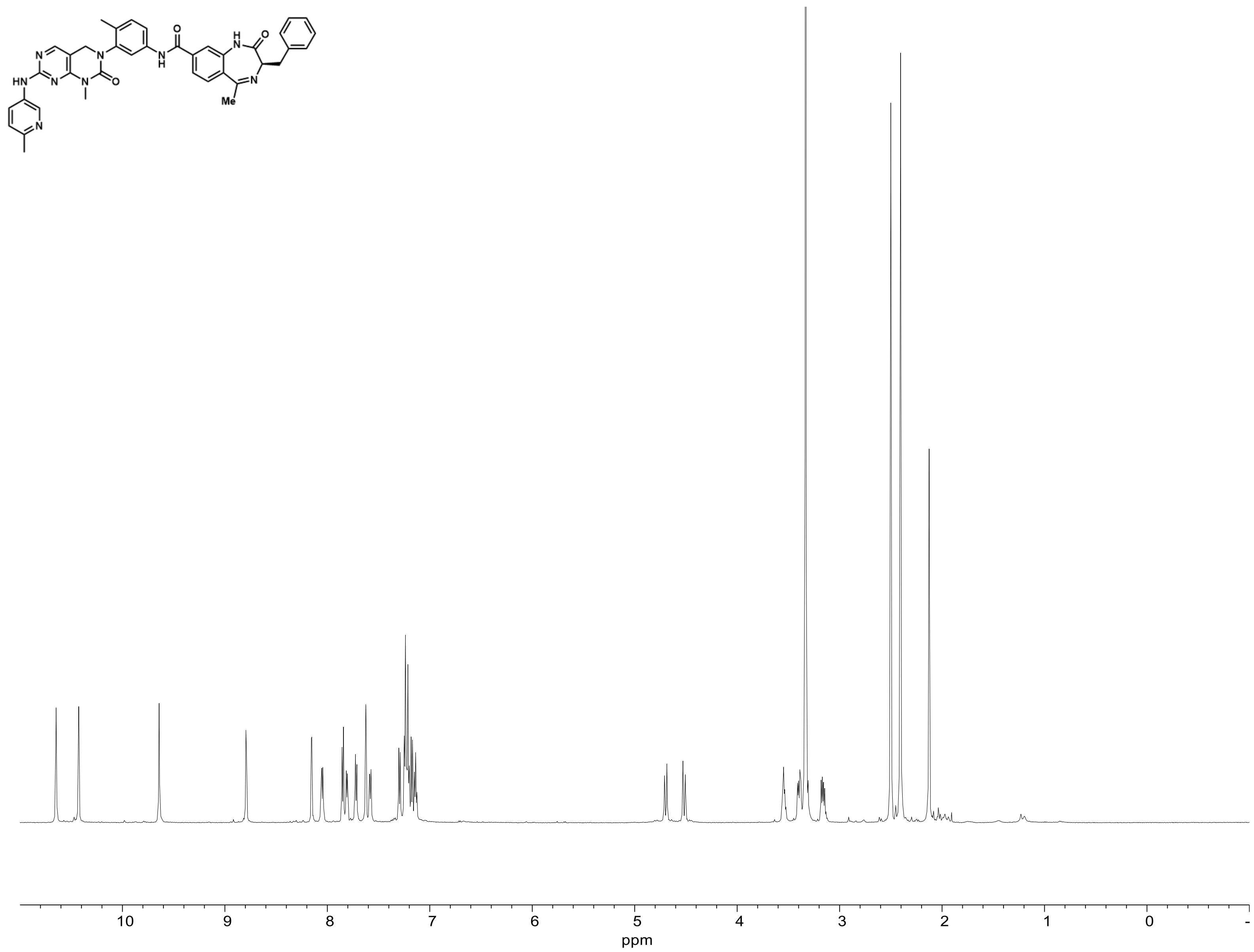

^{1}H NMR (600 MHz, DMSO) of compound **11l**.

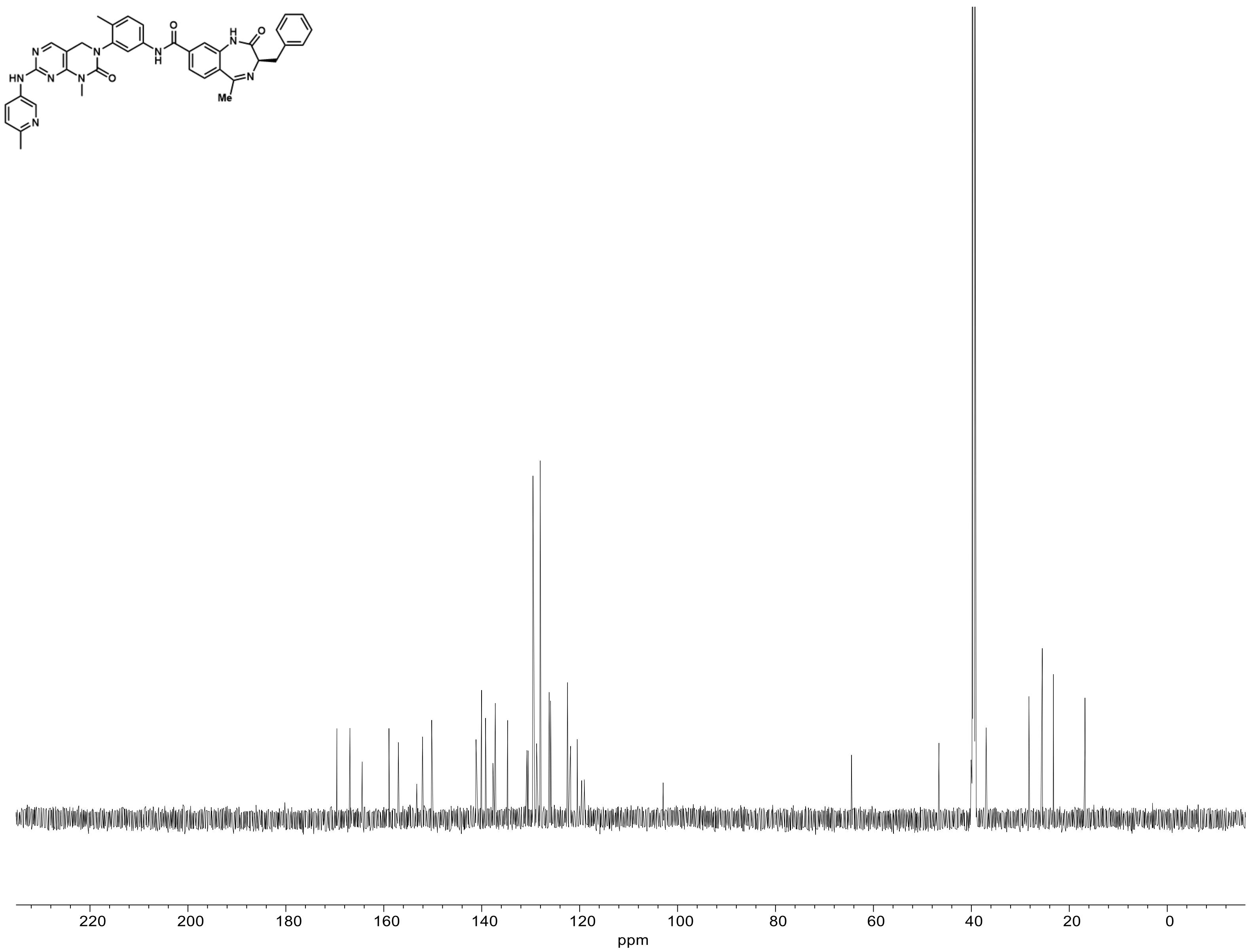

^{13}C NMR (151 MHz, DMSO) of compound **11l.**

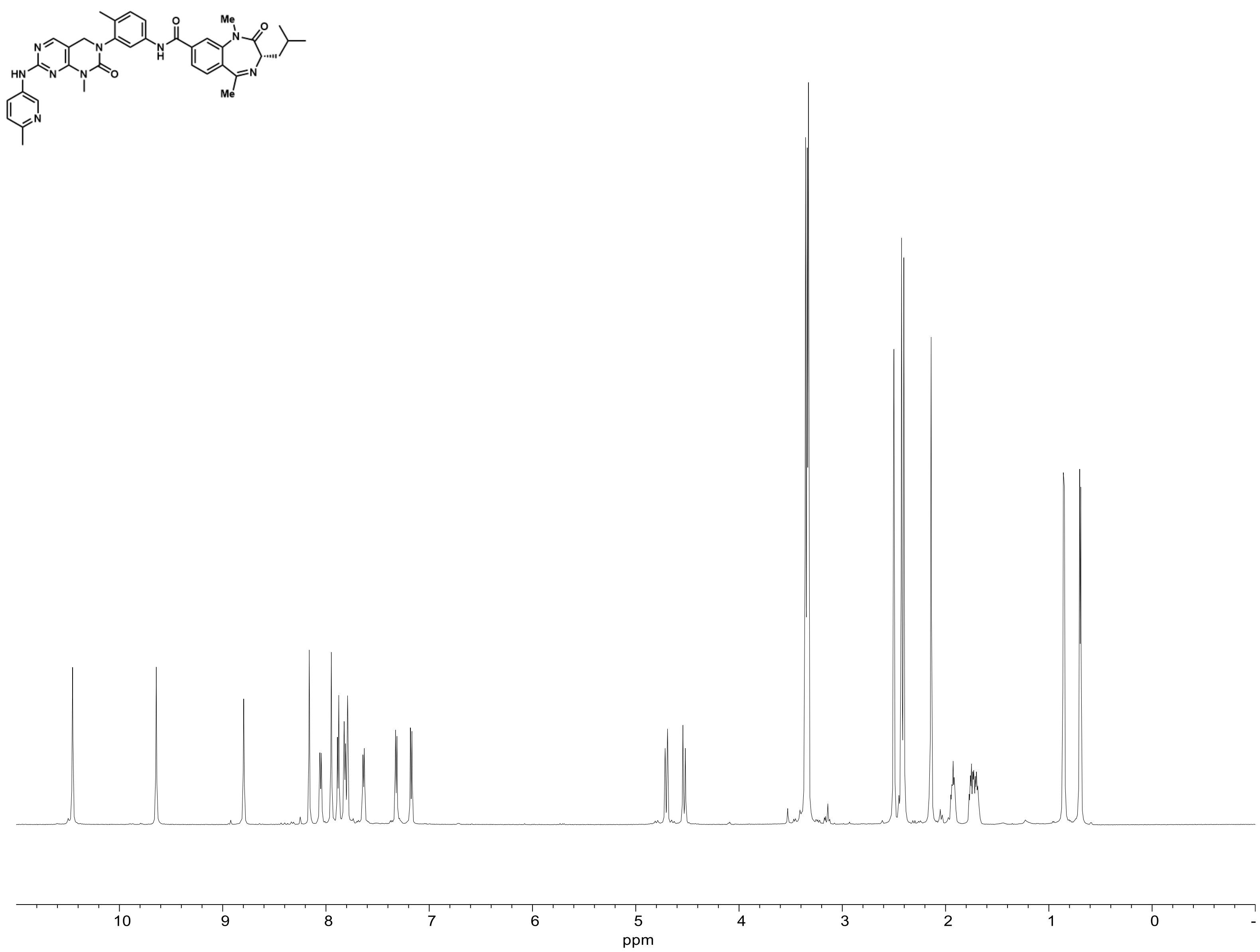

^{1}H NMR (600 MHz, DMSO) of compound **11m**.

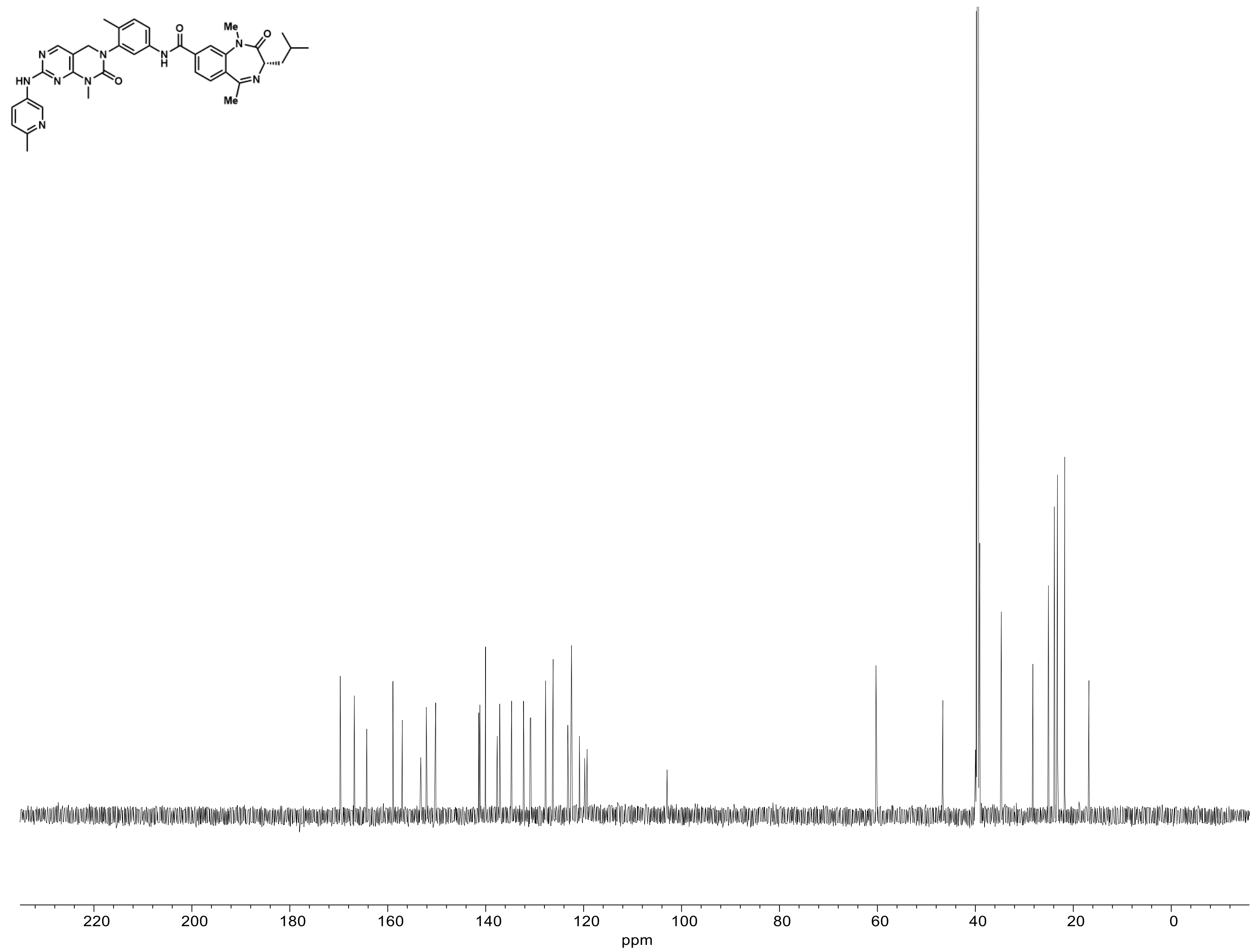

^{13}C NMR (151 MHz, DMSO) of compound **11m.**

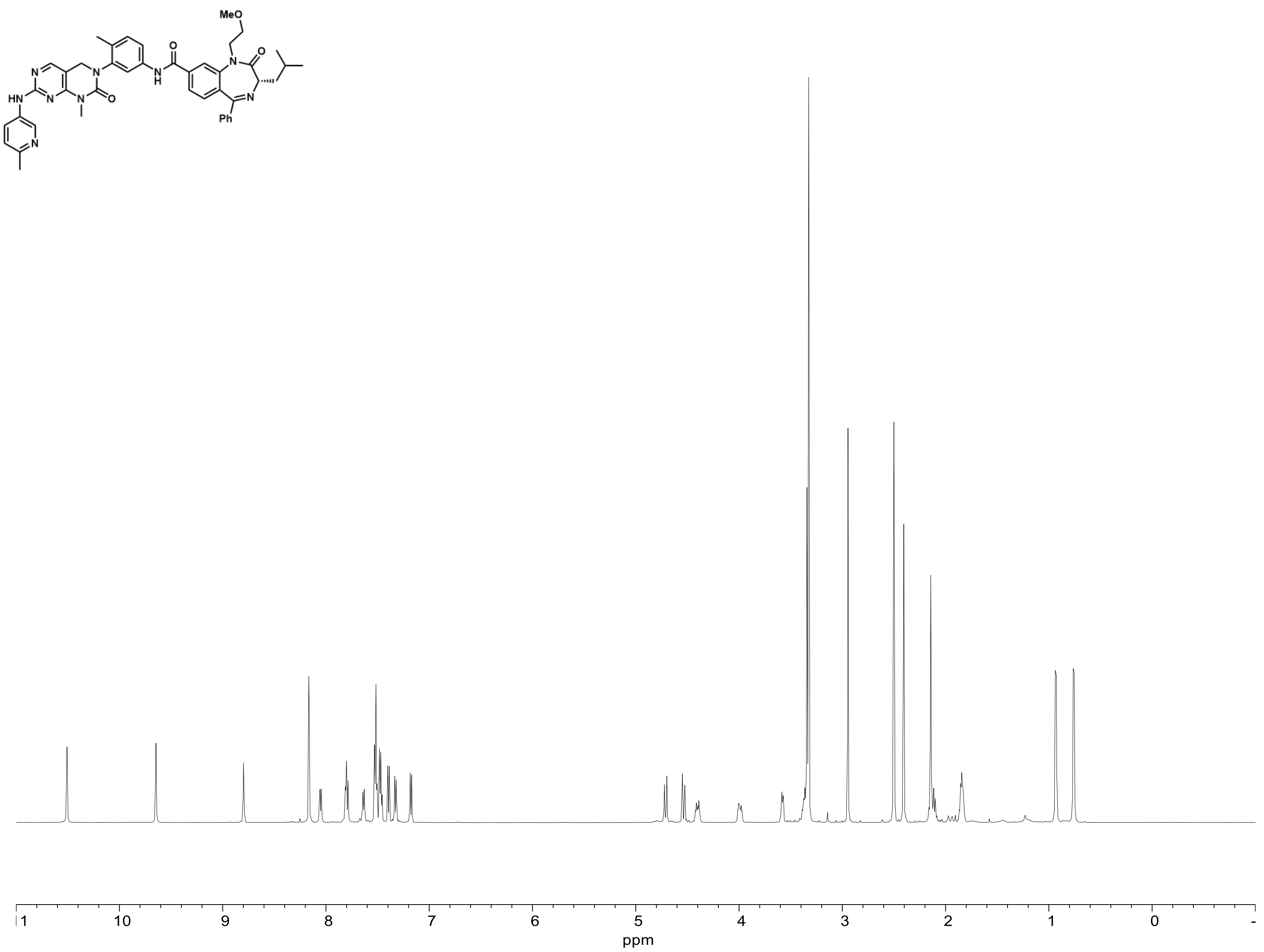

^{1}H NMR (600 MHz, DMSO) of compound **11n**.

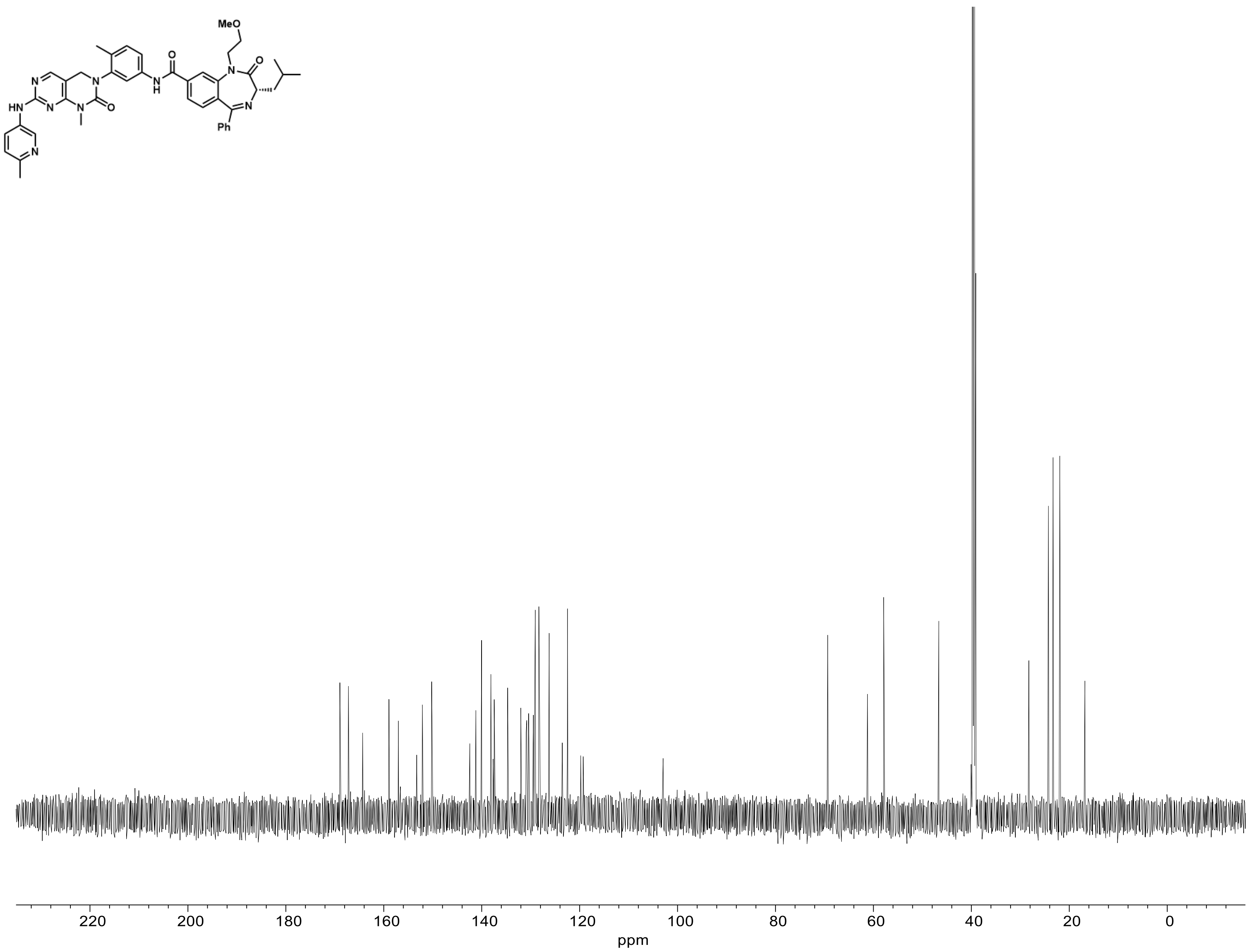

^{13}C NMR (151 MHz, DMSO) of compound **11n.**

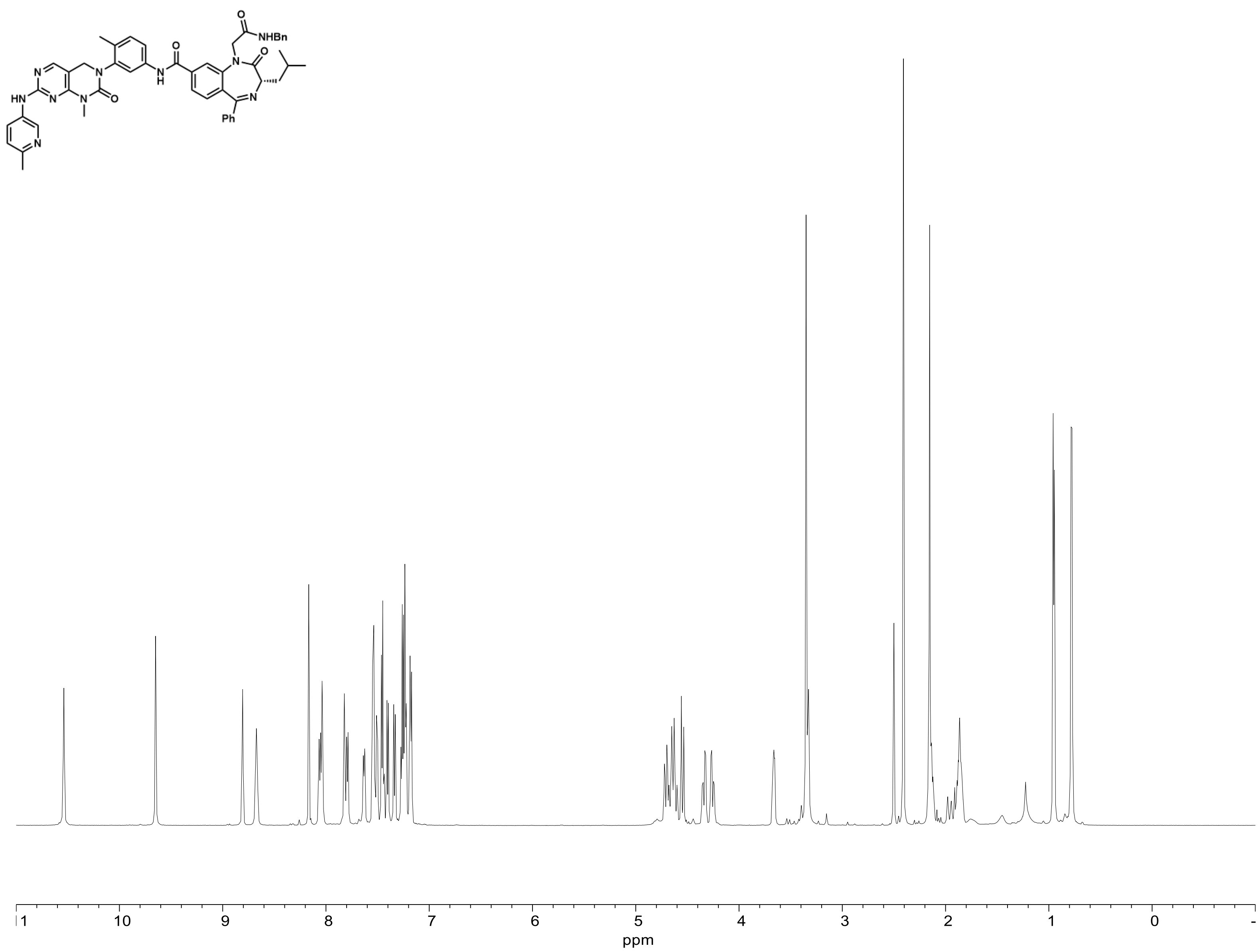

^{1}H NMR (600 MHz, DMSO) of compound **11o**.

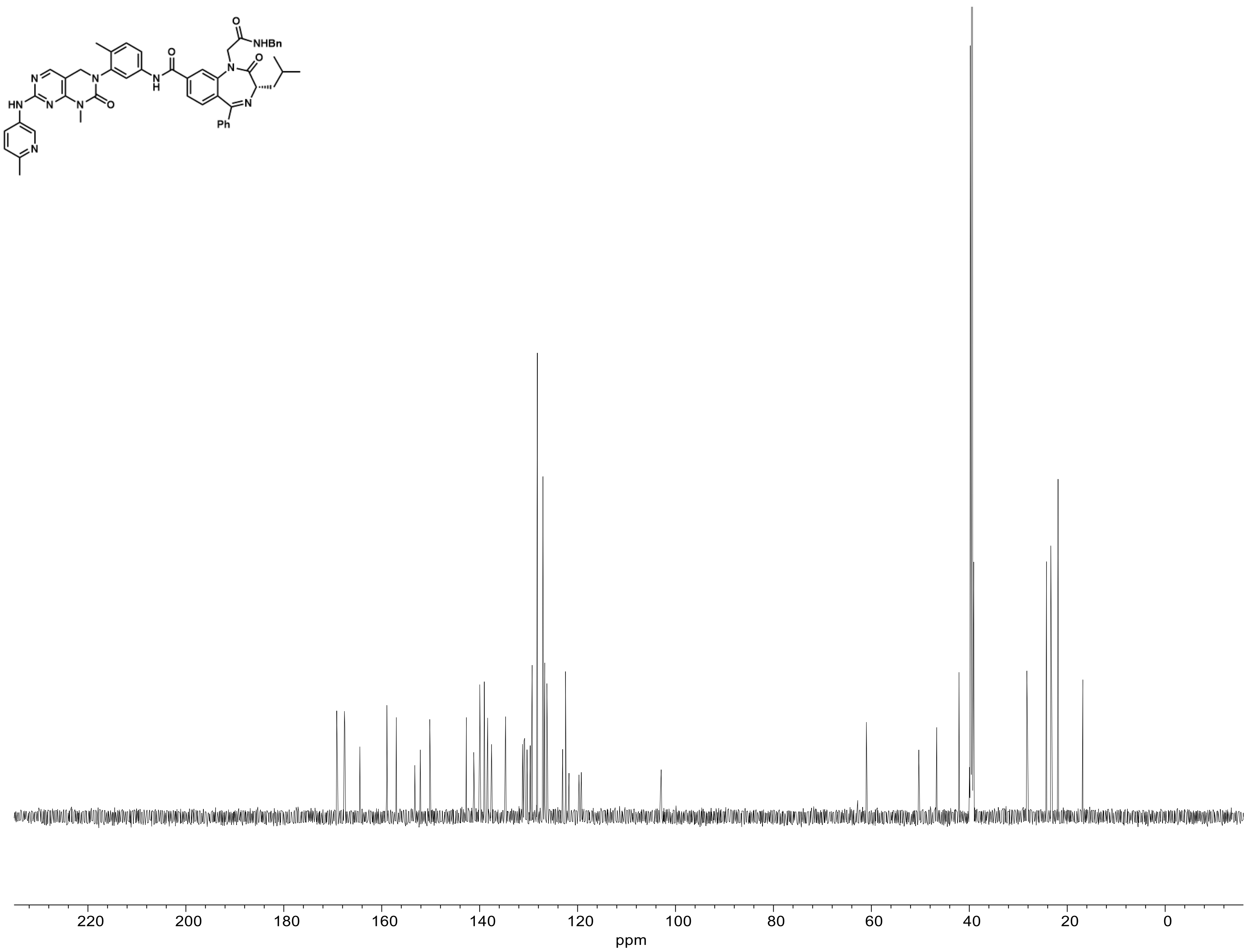

^{13}C NMR (151 MHz, DMSO) of compound **11o.**

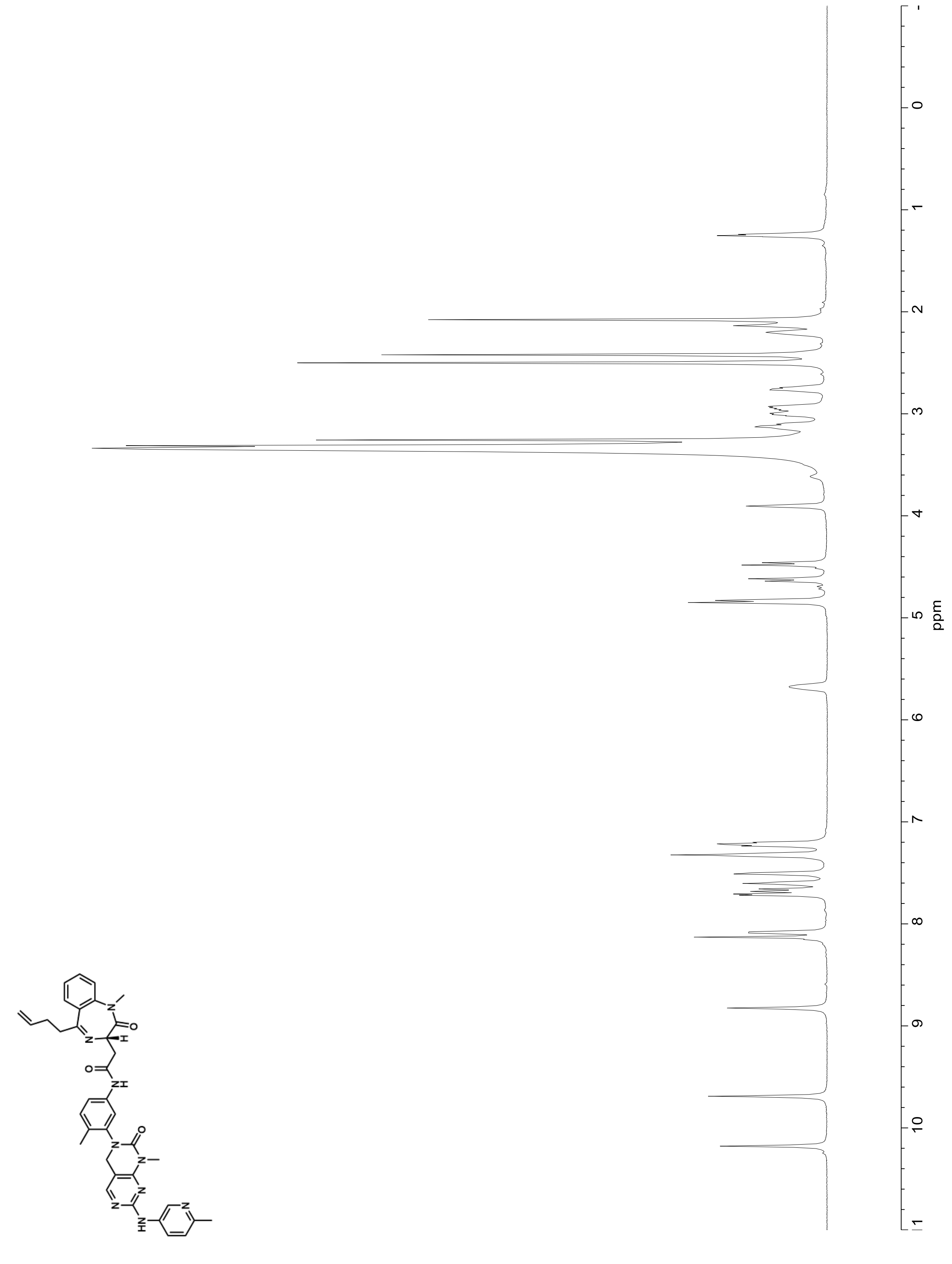

^{1}H NMR (600 MHz, DMSO) of compound **11p**.

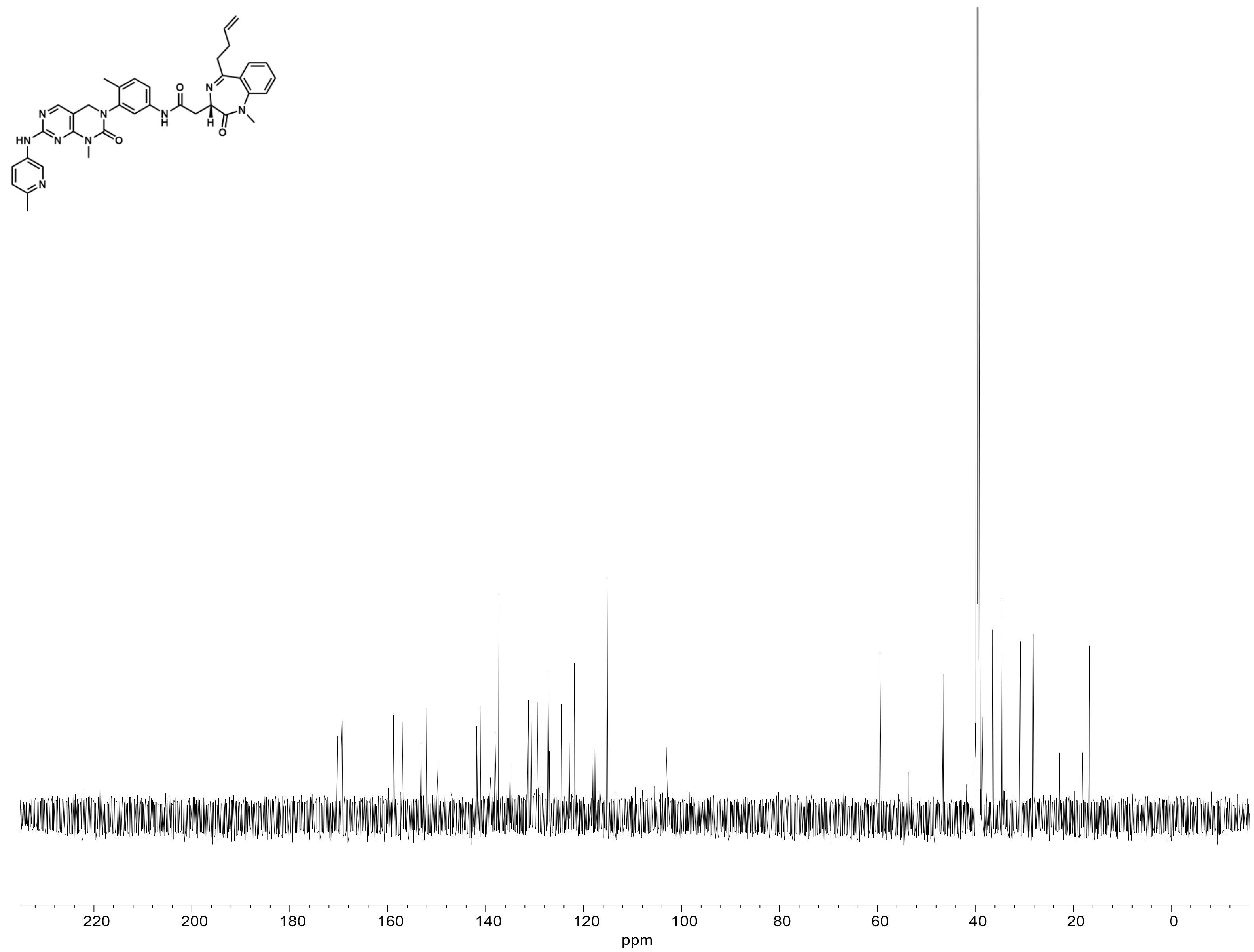

^{13}C NMR (151 MHz, DMSO) of compound **11p.**

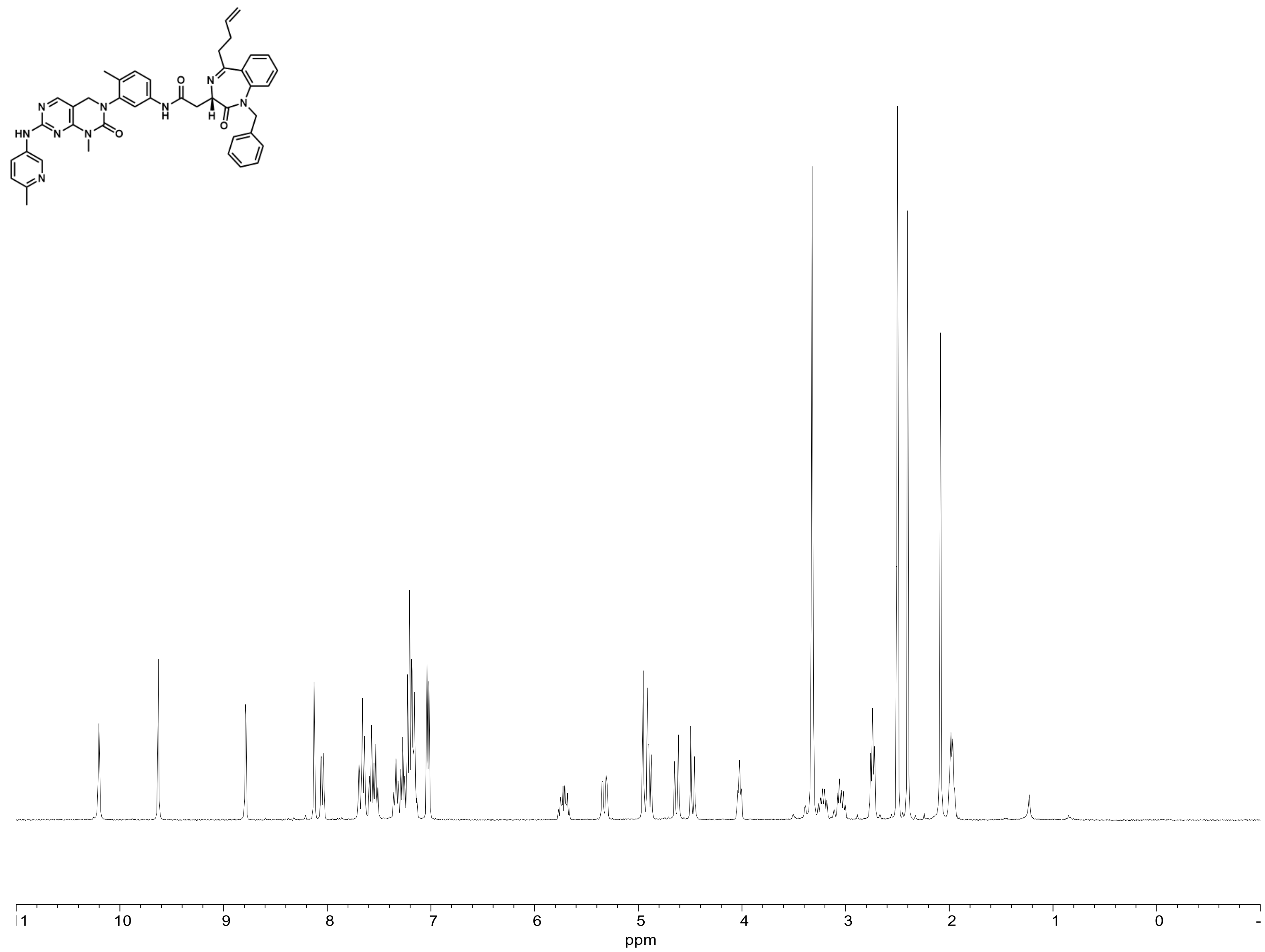

^{1}H NMR (400 MHz, DMSO) of compound **11q**.

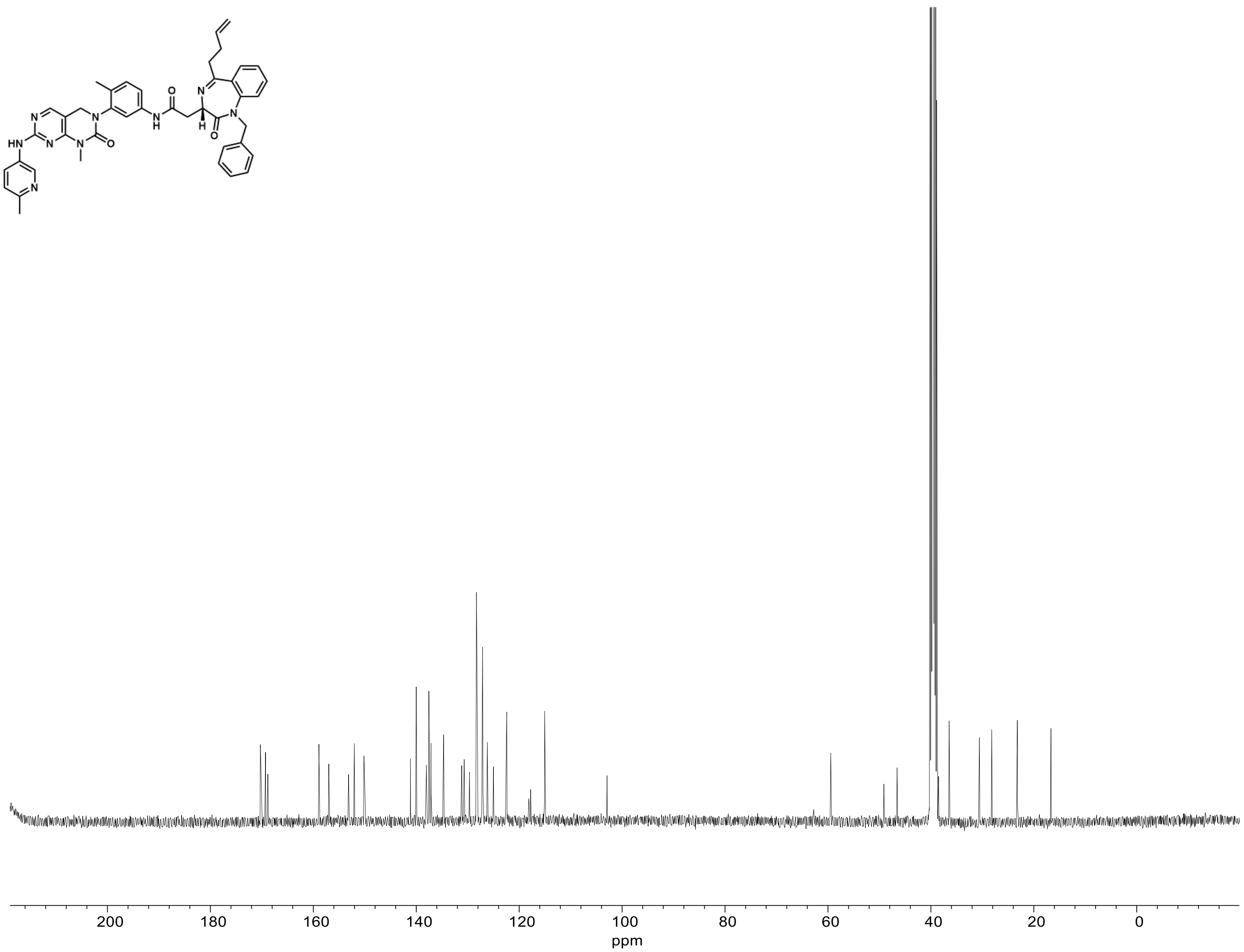

^{13}C NMR (101 MHz, DMSO) of compound **11q.**

HPLC traces of compound **7a**

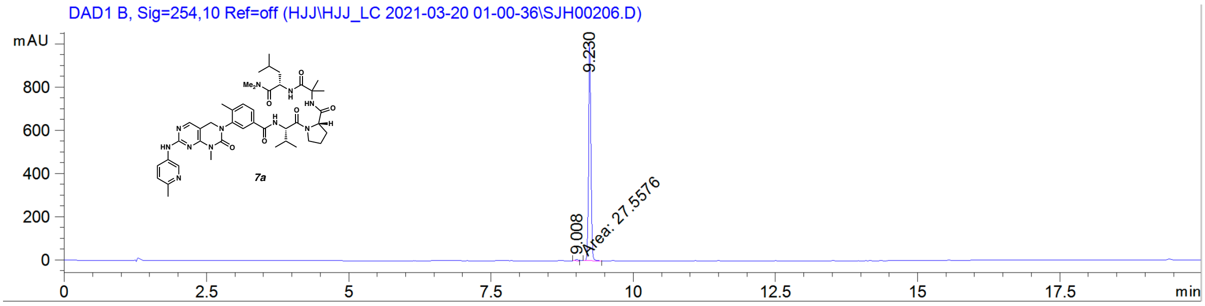

Peak #	RetTime [min]	Type	Width [min]	Area [mAU*s]	Height [mAU]	Area %
1	9.008	MM	0.0659	27.55755	6.96513	0.9492
2	9.230	VB	0.0433	2875.62305	1008.80975	99.0508

HPLC traces of compound **7b**

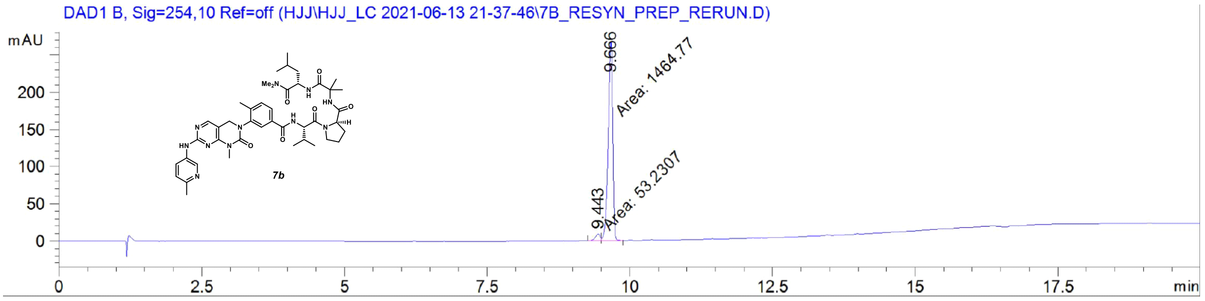

Peak #	RetTime [min]	Type	Width [min]	Area [mAU*s]	Height [mAU]	Area %
1	9.443	MF	0.0983	53.23068	9.02200	3.5066
2	9.666	FM	0.0916	1464.77197	266.44626	96.4934

HPLC traces of compound **11a**

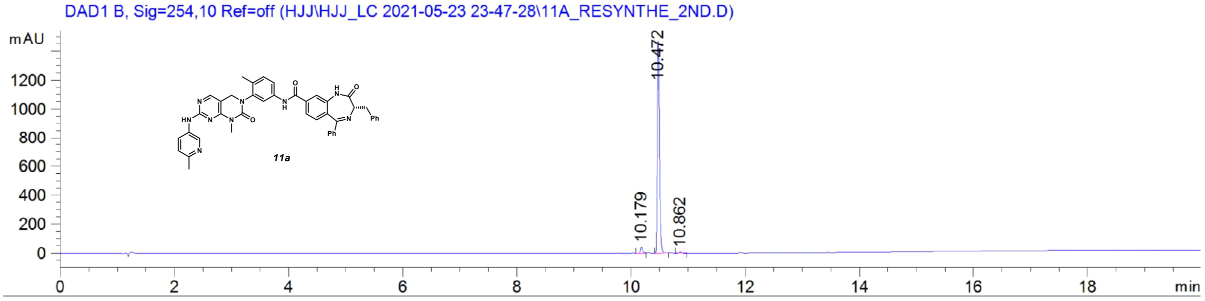

Peak #	RetTime [min]	Type	Width [min]	Area [mAU*s]	Height [mAU]	Area %
1	10.179	VV	0.0420	125.76517	44.45215	3.1725
2	10.472	BV	0.0402	3807.64746	1470.96448	96.0502
3	10.862	VB	0.0469	30.81253	9.48333	0.7773

HPLC traces of compound **11b**

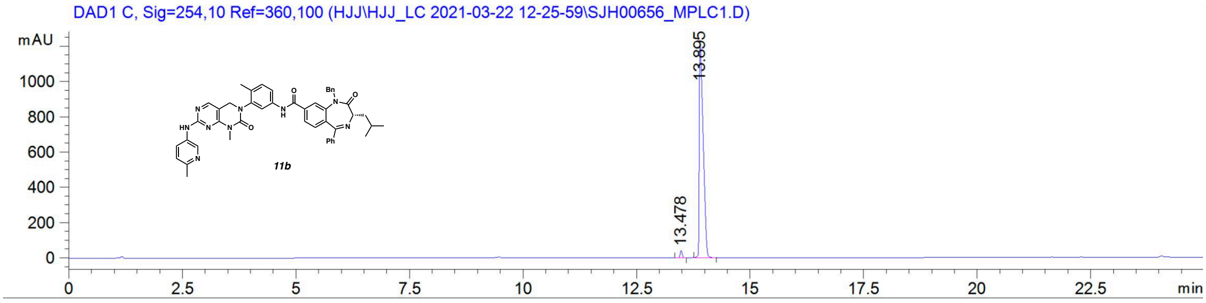

Peak #	RetTime [min]	Type	Width [min]	Area [mAU*s]	Height [mAU]	Area %
1	13.478	VB	0.0493	131.84203	41.19176	1.7660
2	13.895	VV	0.0879	7333.60938	1224.49756	98.2340

HPLC traces of compound **11c**

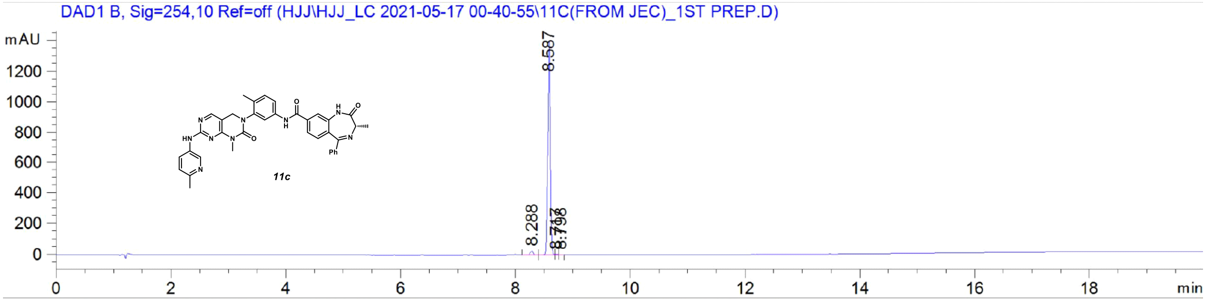

Peak #	RetTime [min]	Type	Width [min]	Area [mAU*s]	Height [mAU]	Area %
1	8.288	VV	0.0516	100.38359	26.84330	2.2865
2	8.587	VV	0.0437	4264.89453	1395.45886	97.1437
3	8.717	VV	0.0452	15.15791	4.75574	0.3453
4	8.798	VV	0.0483	9.85794	2.78444	0.2245

HPLC traces of compound **11d**

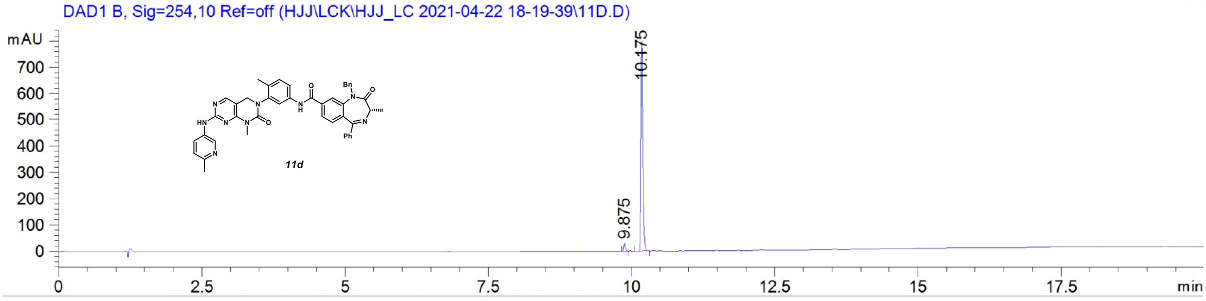

Peak #	RetTime [min]	Type	Width [min]	Area [mAU*s]	Height [mAU]	Area %
1	9.875	VV	0.0334	62.24129	28.62356	3.3586
2	10.175	VV	0.0342	1790.96863	798.67419	96.6414

HPLC traces of compound **11e**

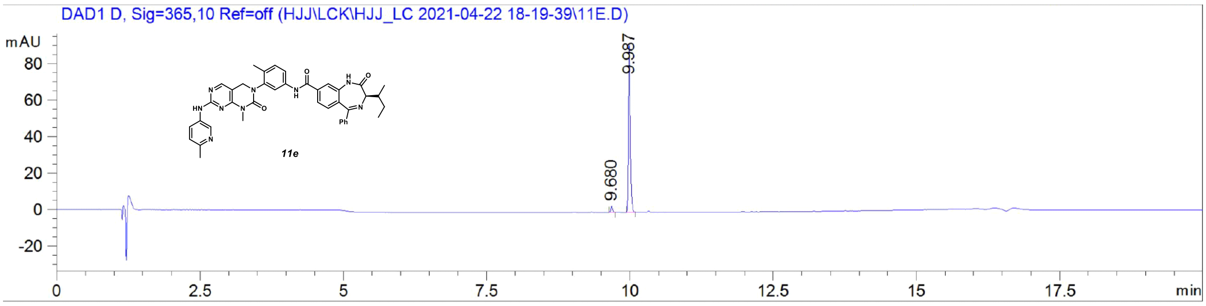

Peak #	RetTime [min]	Type	Width [min]	Area [mAU*s]	Height [mAU]	Area %
1	9.680	BB	0.0337	8.14019	3.69573	3.8109
2	9.987	BB	0.0340	205.46199	92.09421	96.1891

HPLC traces of compound **7c**

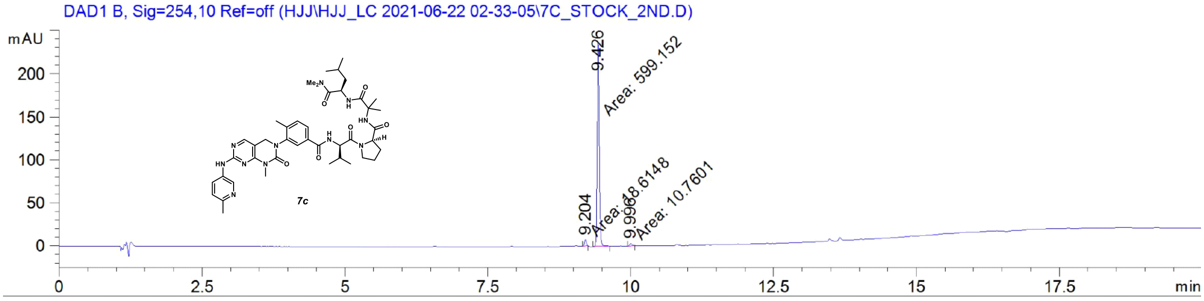

Peak #	RetTime [min]	Type	Width [min]	Area [mAU*s]	Height [mAU]	Area %
1	9.204	MM	0.0410	18.61475	7.56120	2.9616
2	9.426	MM	0.0418	599.15216	238.93765	95.3264
3	9.996	MM	0.0560	10.76012	3.19992	1.7120

HPLC traces of compound **7d**

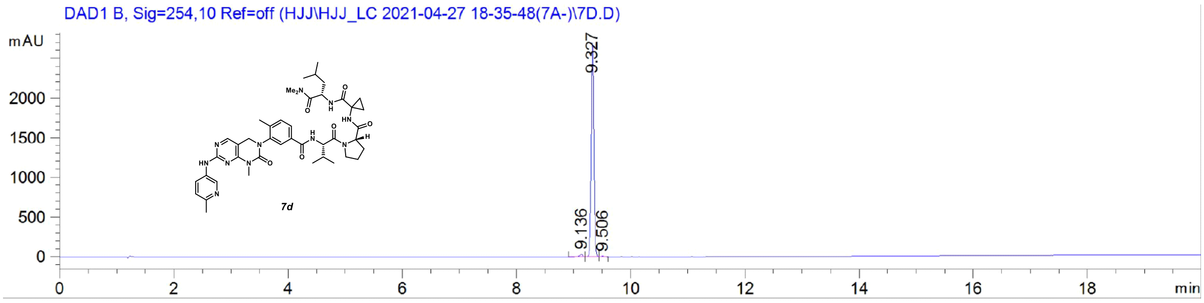

Peak #	RetTime [min]	Type	Width [min]	Area [mAU*s]	Height [mAU]	Area %
1	9.136	VV	0.0596	146.26076	33.73988	1.5837
2	9.327	VV	0.0523	9043.10645	2687.90894	97.9164
3	9.506	VV	0.0716	46.17181	8.88303	0.4999

HPLC traces of compound **7e**

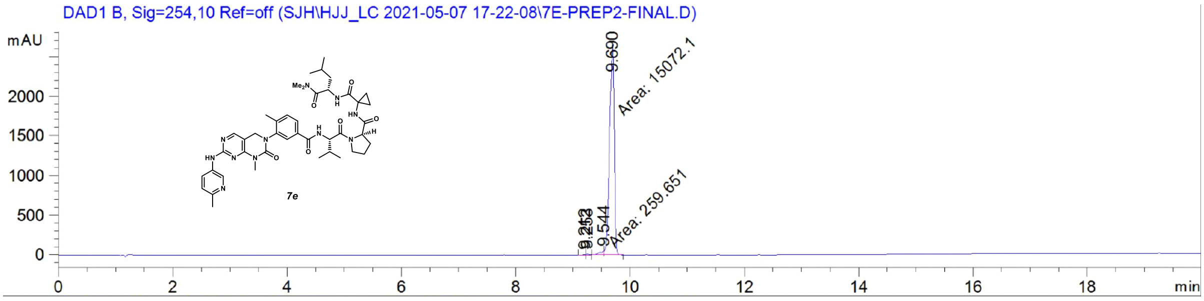

Peak #	RetTime [min]	Type	Width [min]	Area [mAU*s]	Height [mAU]	Area %
1	9.212	VV	0.0528	35.61513	9.71646	0.2310
2	9.253	VV	0.0478	51.43330	14.69552	0.3336
3	9.544	MF	0.0976	259.65070	44.35475	1.6840
4	9.690	FM	0.0937	1.50721e4	2681.05103	97.7515

HPLC traces of compound **7f**

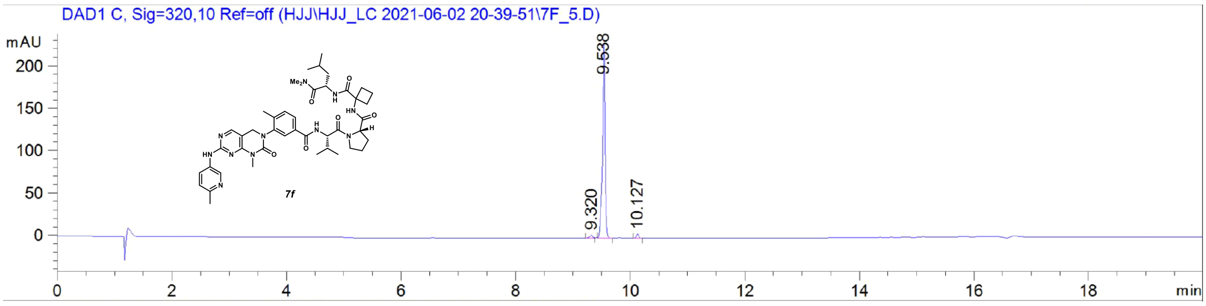

Peak #	RetTime [min]	Type	Width [min]	Area [mAU*s]	Height [mAU]	Area %
1	9.320	BV	0.0546	14.58645	3.81230	1.9049
2	9.538	VB	0.0466	736.40417	228.68266	96.1675
3	10.127	BB	0.0425	14.76134	5.13653	1.9277

HPLC traces of compound **7g**

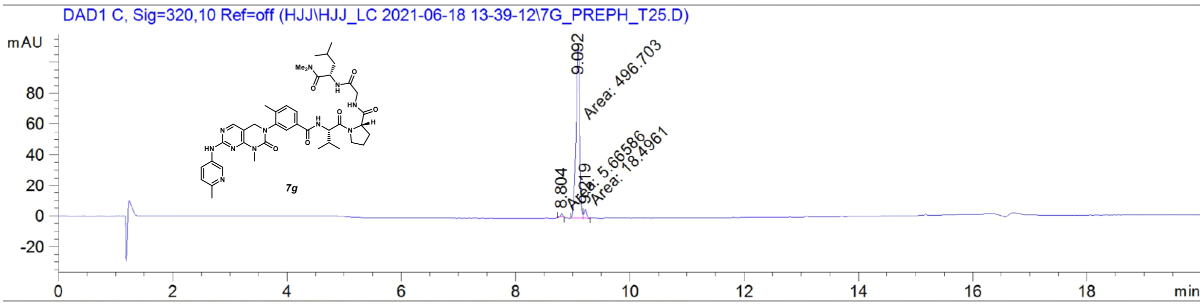

Peak #	RetTime [min]	Type	Width [min]	Area [mAU*s]	Height [mAU]	Area %
1	8.804	MM	0.0392	5.66586	2.40817	1.0878
2	9.092	MF	0.0732	496.70337	113.05464	95.3612
3	9.219	FM	0.0508	18.49609	6.06331	3.5510

HPLC traces of compound **7h**

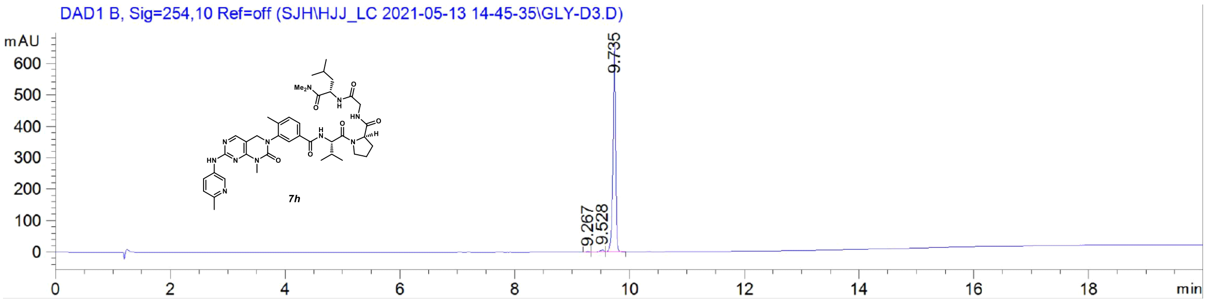

Peak #	RetTime [min]	Type	Width [min]	Area [mAU*s]	Height [mAU]	Area %
1	9.267	BV	0.0481	9.34000	2.65109	0.4268
2	9.528	VV	0.0692	36.41834	7.18092	1.6643
3	9.735	VB	0.0477	2142.49487	662.73029	97.9089

HPLC traces of compound **7i**

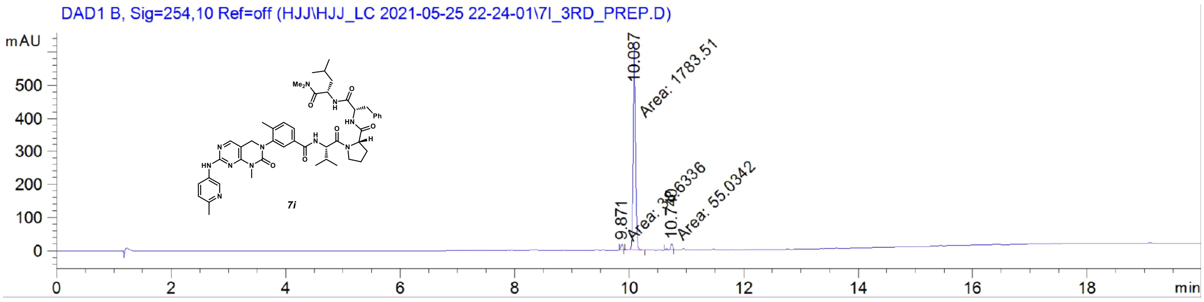

Peak #	RetTime [min]	Type	Width [min]	Area [mAU*s]	Height [mAU]	Area %
1	9.871	MM	0.0417	38.63363	15.45284	2.0581
2	10.087	MM	0.0476	1783.51135	625.01355	95.0102
3	10.740	MM	0.0530	55.03423	17.29789	2.9318

HPLC traces of compound **7j**

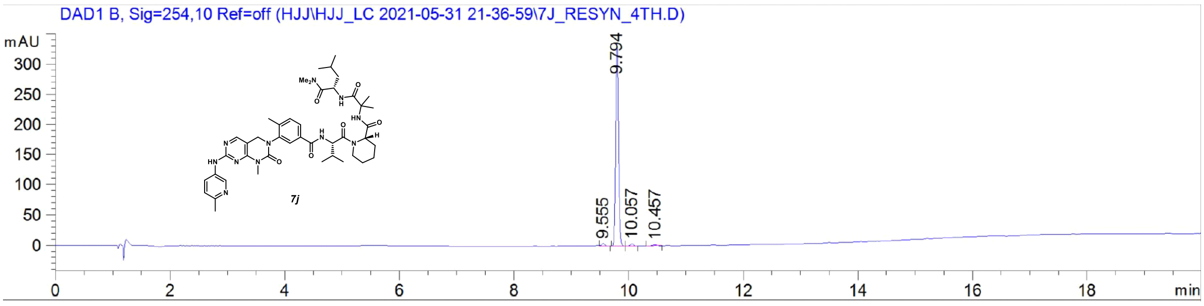

Peak #	RetTime [min]	Type	Width [min]	Area [mAU*s]	Height [mAU]	Area %
1	9.555	VB	0.0633	18.40376	4.27063	1.5295
2	9.794	BV	0.0534	1158.24963	334.22177	96.2621
3	10.057	VV	0.0651	16.03838	3.81193	1.3329
4	10.457	VV	0.0860	10.53312	1.75498	0.8754

HPLC traces of compound **7k**

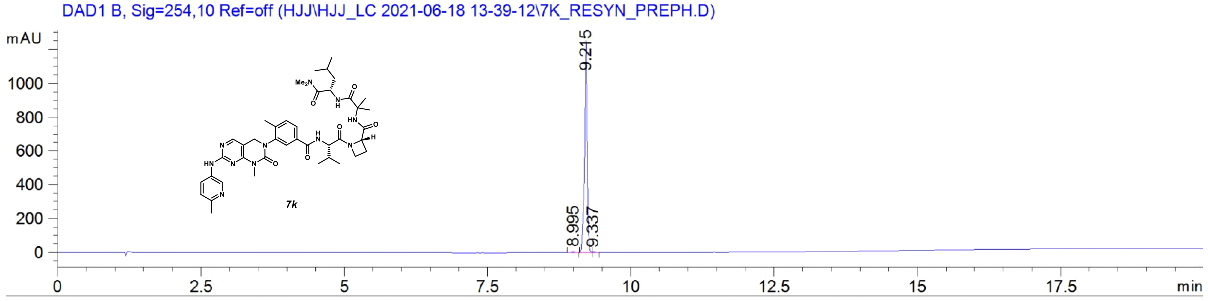

Peak #	RetTime [min]	Type	Width [min]	Area [mAU*s]	Height [mAU]	Area %
1	8.995	BB	0.0522	20.44724	5.52356	0.5069
2	9.215	BV	0.0463	4000.89990	1251.76660	99.1859
3	9.337	VB	0.0531	12.39043	3.13908	0.3072

HPLC traces of compound **7l**

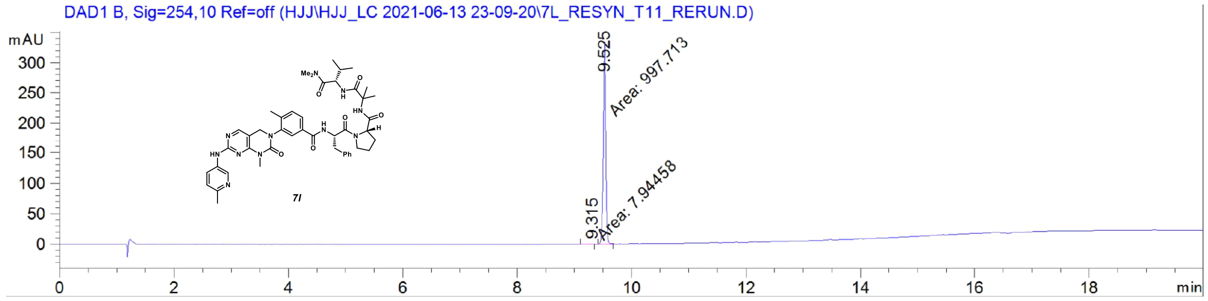

Peak #	RetTime [min]	Type	Width [min]	Area [mAU*s]	Height [mAU]	Area %
1	9.315	MM	0.2264	7.94458	5.84933e-1	0.7900
2	9.525	MM	0.0496	997.71277	335.31351	99.2100

HPLC traces of compound **7m**

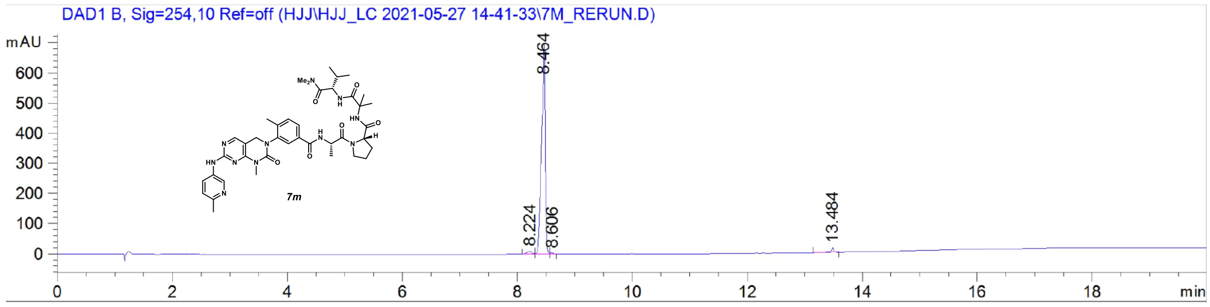

Peak #	RetTime [min]	Type	Width [min]	Area [mAU*s]	Height [mAU]	Area %
1	8.224	BV	0.0804	59.20930	9.67649	1.8811
2	8.464	VV	0.0579	3034.09229	695.17010	96.3942
3	8.606	VV	0.0542	14.50137	3.82716	0.4607
4	13.484	BB	0.0391	39.78504	15.41708	1.2640

HPLC traces of compound **7n**

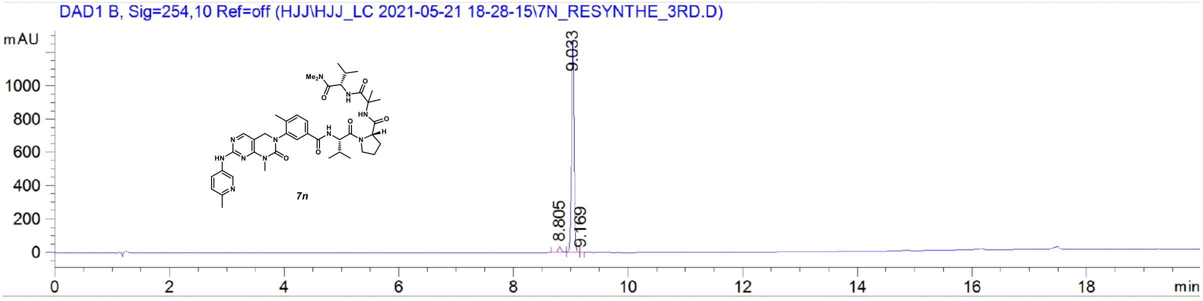

Peak #	RetTime [min]	Type	Width [min]	Area [mAU*s]	Height [mAU]	Area %
1	8.805	VV	0.0587	154.26880	36.97253	3.9047
2	9.033	VV	0.0449	3789.79810	1266.09363	95.9234
3	9.169	VV	0.0569	6.79159	1.84877	0.1719

HPLC traces of compound **7o**

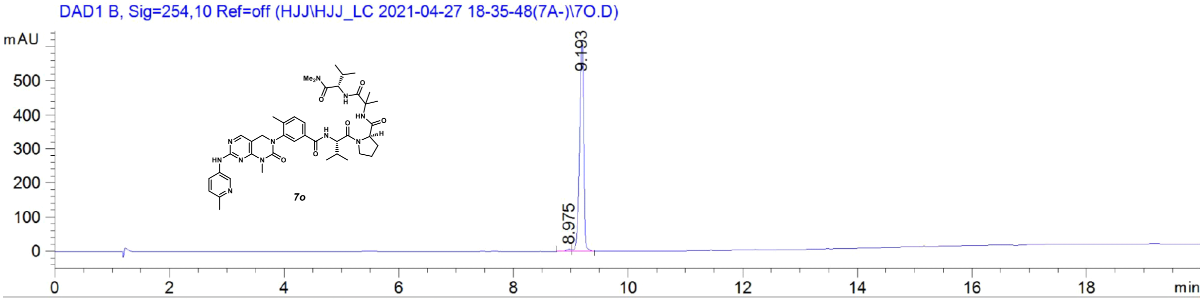

Peak #	RetTime [min]	Type	Width [min]	Area [mAU*s]	Height [mAU]	Area %
1	8.975	BV	0.0966	43.17449	6.75272	1.3606
2	9.193	VB	0.0793	3129.94434	615.63715	98.6394

HPLC traces of compound **7p**

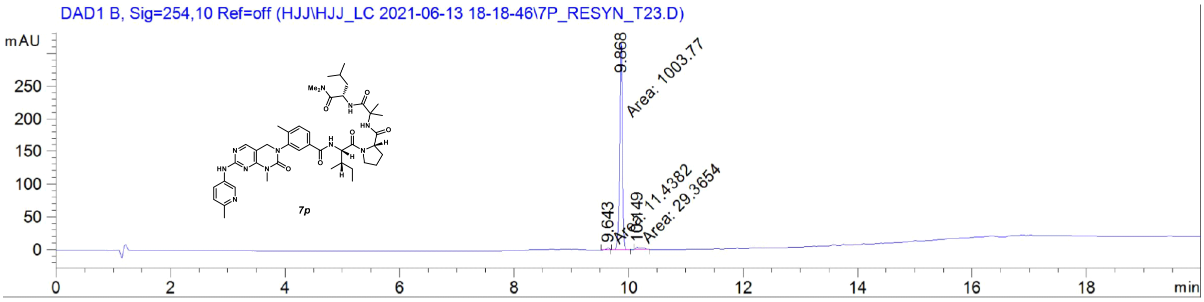

Peak #	RetTime [min]	Type	Width [min]	Area [mAU*s]	Height [mAU]	Area %
1	9.643	MM	0.0564	11.43824	3.38221	1.0950
2	9.868	MM	0.0528	1003.77295	316.56317	96.0938
3	10.149	MM	0.1311	29.36538	3.73358	2.8112

HPLC traces of compound **7q**

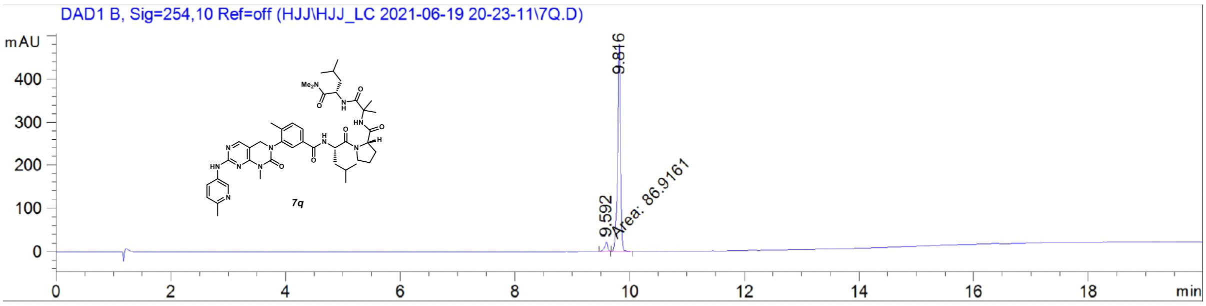

Peak #	RetTime [min]	Type	Width [min]	Area [mAU*s]	Height [mAU]	Area %
1	9.592	MM	0.0666	86.91608	21.74711	4.9741
2	9.816	VV	0.0503	1660.44238	480.10712	95.0259

HPLC traces of compound **7r**

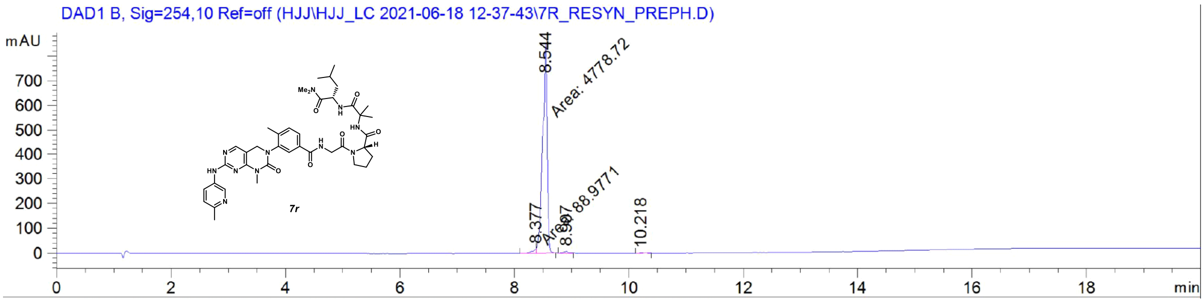

Peak #	RetTime [min]	Type	Width [min]	Area [mAU*s]	Height [mAU]	Area %
1	8.377	MF	0.0769	88.97707	19.29301	1.7994
2	8.544	FM	0.0934	4778.71826	852.99945	96.6386
3	8.907	VB	0.0829	57.91838	9.14822	1.1713
4	10.218	BV	0.1110	19.32523	2.62280	0.3908

HPLC traces of compound **7s**

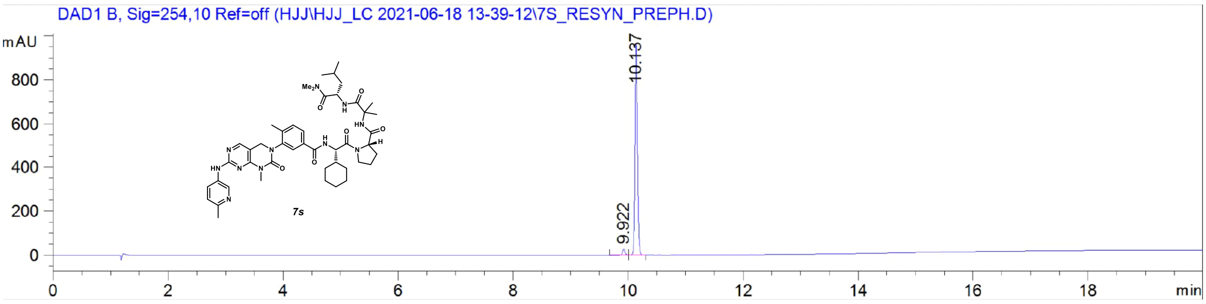

Peak #	RetTime [min]	Type	Width [min]	Area [mAU*s]	Height [mAU]	Area %
1	9.922	BV	0.0503	95.77647	27.74433	3.2895
2	10.137	VB	0.0451	2815.83887	962.54089	96.7105

HPLC traces of compound **7t**

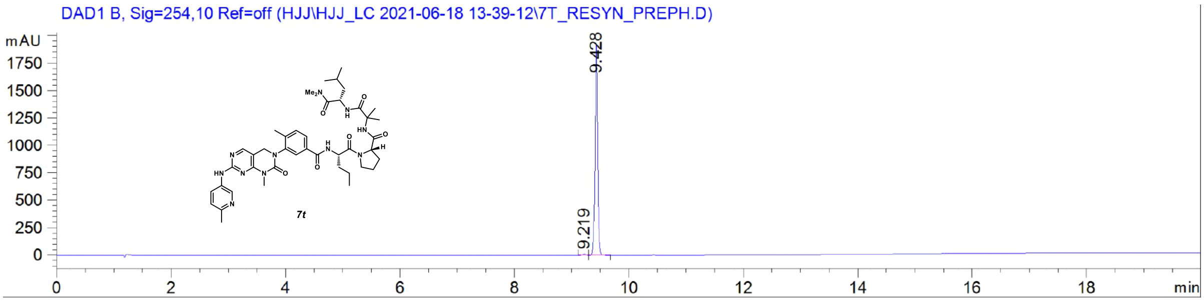

Peak #	RetTime [min]	Type	Width [min]	Area [mAU*s]	Height [mAU]	Area %
1	9.219	BV	0.0569	48.15348	12.23041	0.7823
2	9.428	VB	0.0469	6107.16260	1932.43384	99.2177

HPLC traces of compound **7u**

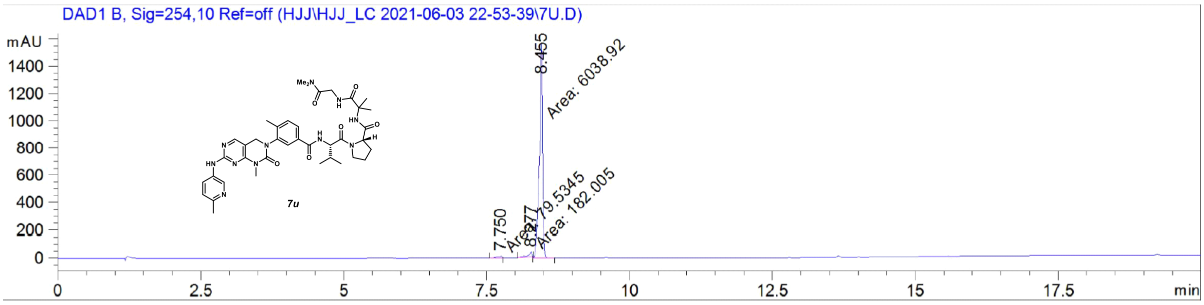

Peak #	RetTime [min]	Type	Width [min]	Area [mAU*s]	Height [mAU]	Area %
1	7.750	MM	0.0871	79.53450	15.22383	1.2624
2	8.277	MM	0.0788	182.00494	38.51012	2.8888
3	8.455	FM	0.0643	6038.92383	1566.29004	95.8489

HPLC traces of compound **7v**

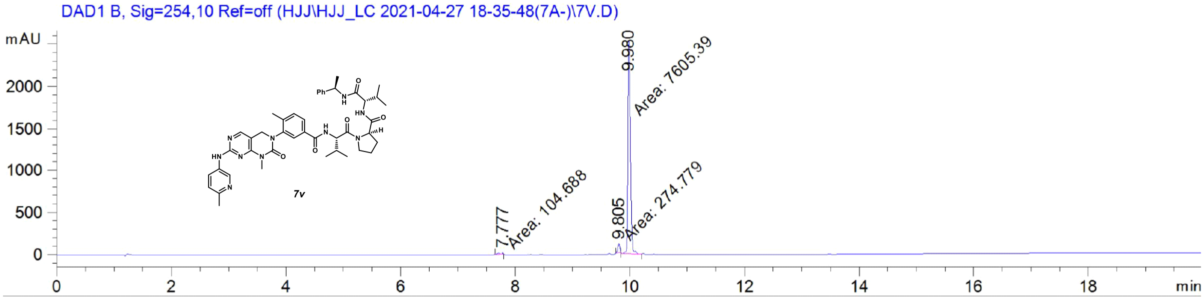

Peak #	RetTime [min]	Type	Width [min]	Area [mAU*s]	Height [mAU]	Area %
1	7.777	MM	0.0788	104.68790	22.14503	1.3111
2	9.805	MM	0.0429	274.77905	106.65346	3.4413
3	9.980	MM	0.0501	7605.38818	2530.95386	95.2477

HPLC traces of compound **7w**

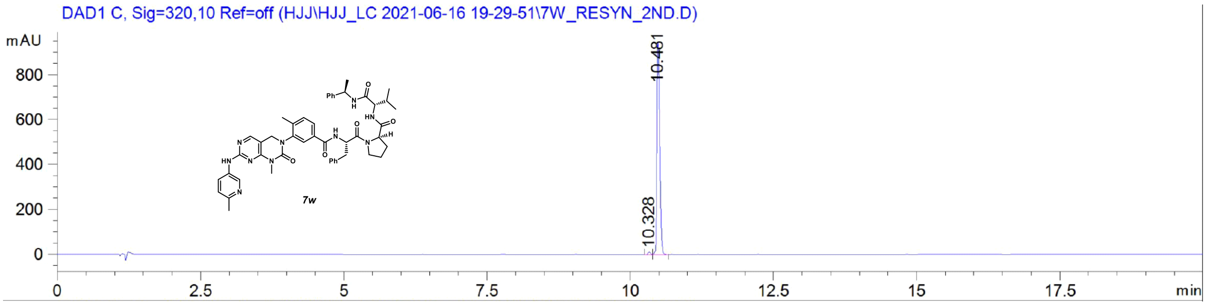

Peak #	RetTime [min]	Type	Width [min]	Area [mAU*s]	Height [mAU]	Area %
1	10.328	VV	0.0476	35.38710	11.27137	1.0942
2	10.481	VV	0.0525	3198.62866	944.24017	98.9058

HPLC traces of compound **11f**

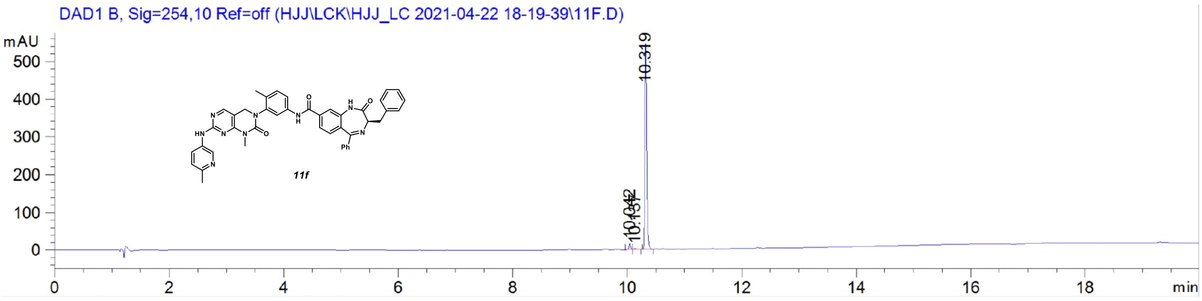

Peak #	RetTime [min]	Type	Width [min]	Area [mAU*s]	Height [mAU]	Area %
1	10.042	BV	0.0332	36.71595	16.98262	2.9429
2	10.137	VB	0.0461	11.85702	3.63308	0.9504
3	10.319	BV	0.0336	1199.02881	545.43951	96.1067

HPLC traces of compound **11g**

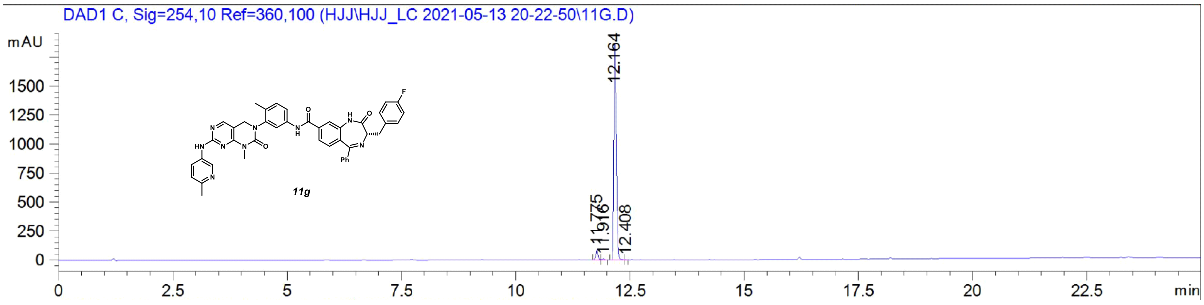

Peak #	RetTime [min]	Type	Width [min]	Area [mAU*s]	Height [mAU]	Area %
1	11.775	VV	0.0471	295.00531	92.67207	3.9110
2	11.916	VB	0.0543	33.45332	9.00924	0.4435
3	12.164	BV	0.0612	7203.42139	1860.75488	95.4996
4	12.408	VV	0.0552	11.00469	2.96934	0.1459

HPLC traces of compound **11h**

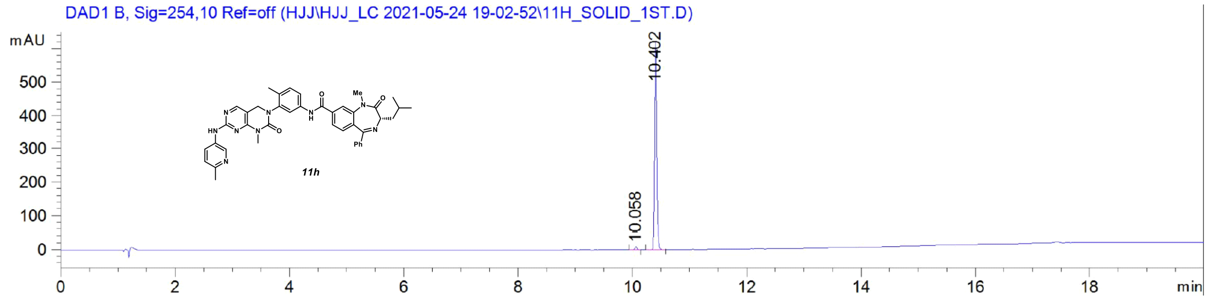

Peak #	RetTime [min]	Type	Width [min]	Area [mAU*s]	Height [mAU]	Area %
1	10.058	BB	0.0423	24.36575	8.81215	1.4672
2	10.402	VB	0.0409	1636.36023	617.77399	98.5328

HPLC traces of compound **11i**

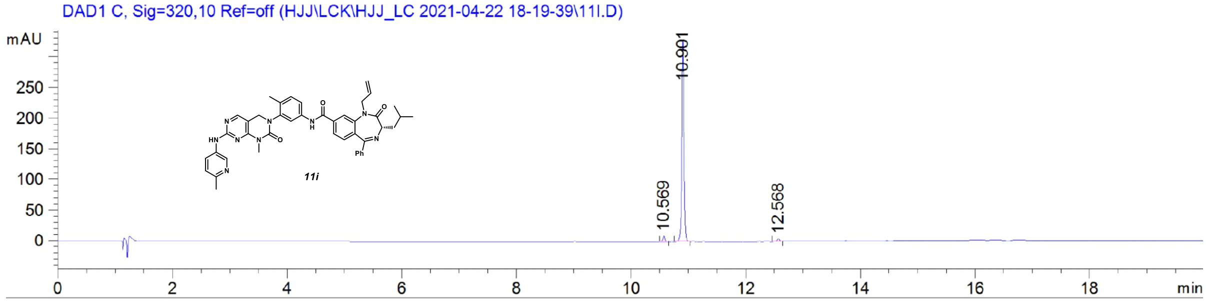

Peak #	RetTime [min]	Type	Width [min]	Area [mAU*s]	Height [mAU]	Area %
1	10.569	BB	0.0385	24.44312	9.65319	2.7738
2	10.901	VV	0.0392	843.28687	325.48248	95.6959
3	12.568	VB	0.0428	13.48572	4.80391	1.5304

HPLC traces of compound **11j**

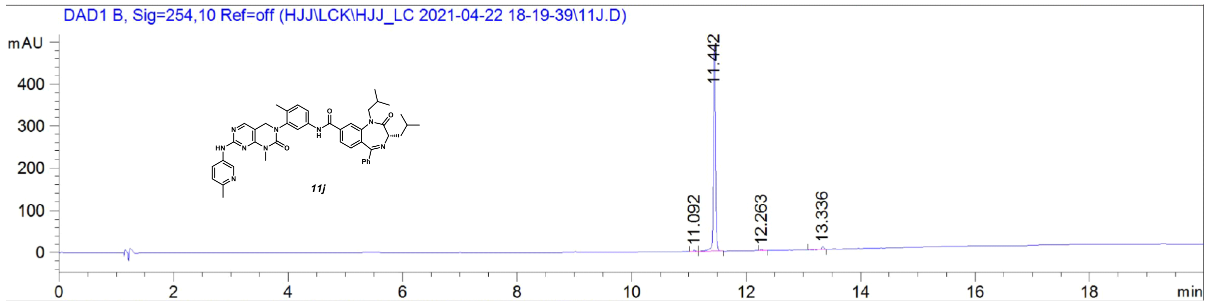

Peak #	RetTime [min]	Type	Width [min]	Area [mAU*s]	Height [mAU]	Area %
1	11.092	VV	0.0463	10.08756	3.07399	0.8059
2	11.442	VB	0.0379	1209.05823	488.81253	96.5948
3	12.263	VB	0.0535	10.50456	2.88097	0.8392
4	13.336	BV	0.0436	22.03045	7.43057	1.7601

HPLC traces of compound **11k**

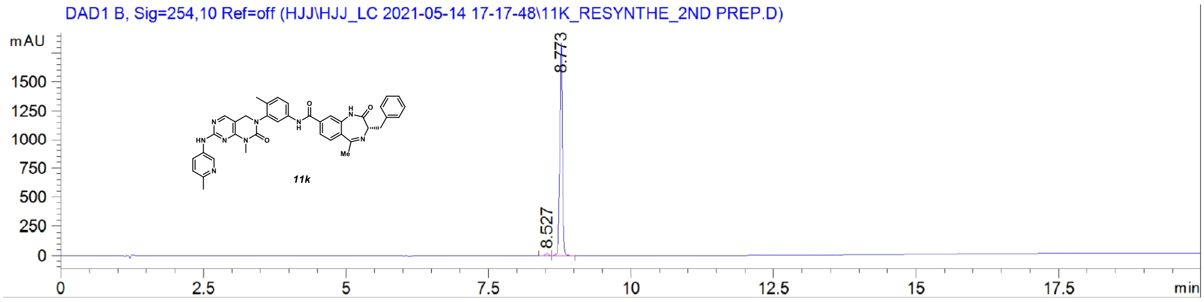

Peak #	RetTime [min]	Type	Width [min]	Area [mAU*s]	Height [mAU]	Area %
1	8.527	VV	0.0549	86.32736	21.49703	1.4982
2	8.773	VV	0.0441	5675.86963	1834.79102	98.5018

HPLC traces of compound **11l**

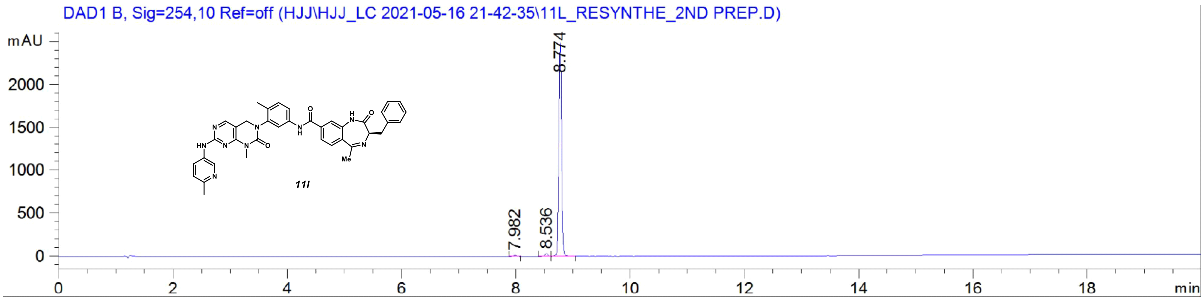

Peak #	RetTime [min]	Type	Width [min]	Area [mAU*s]	Height [mAU]	Area %
1	7.982	BV	0.0574	56.05490	14.08473	0.6629
2	8.536	VV	0.0527	120.13660	31.34749	1.4208
3	8.774	VB	0.0498	8279.62988	2489.16992	97.9163

HPLC traces of compound **11m**

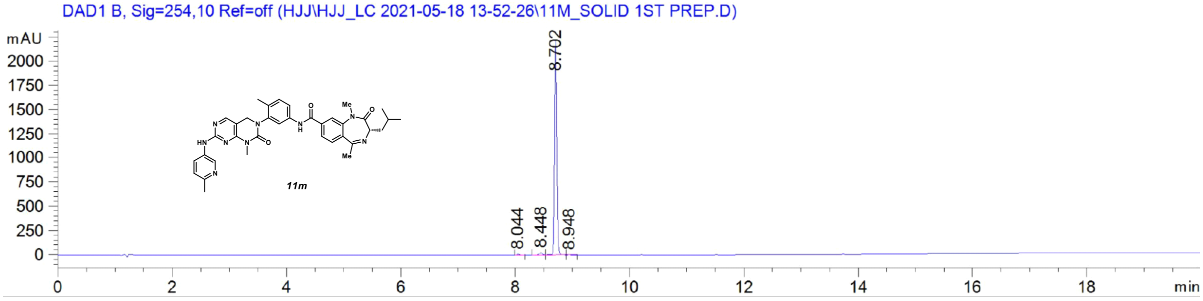

Peak #	RetTime [min]	Type	Width [min]	Area [mAU*s]	Height [mAU]	Area %
1	8.044	BB	0.0474	30.08302	9.13649	0.4677
2	8.448	VV	0.0594	101.12366	24.90970	1.5723
3	8.702	VV	0.0442	6282.35889	2209.77759	97.6803
4	8.948	VB	0.0969	17.98848	2.62612	0.2797

HPLC traces of compound **11n**

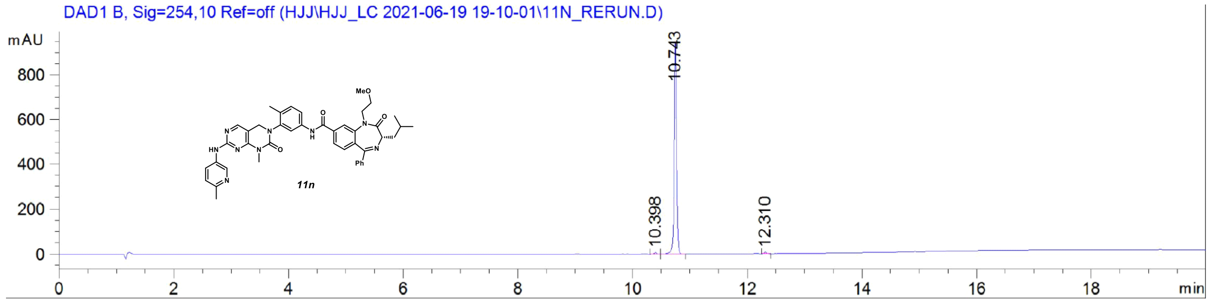

Peak #	RetTime [min]	Type	Width [min]	Area [mAU*s]	Height [mAU]	Area %
1	10.398	VV	0.0514	31.73823	8.95016	1.1305
2	10.743	VV	0.0430	2752.85034	944.88593	98.0521
3	12.310	VV	0.0446	22.94877	7.97874	0.8174

HPLC traces of compound **11o**

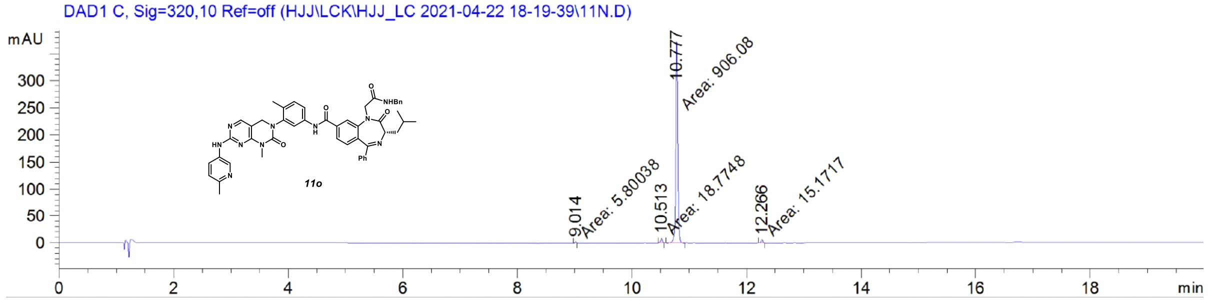

Peak #	RetTime [min]	Type	Width [min]	Area [mAU*s]	Height [mAU]	Area %
1	9.014	MM	0.0282	5.80038	3.42356	0.6133
2	10.513	MM	0.0368	18.77477	8.50128	1.9850
3	10.777	MM	0.0403	906.07990	374.60904	95.7977
4	12.266	MM	0.0377	15.17171	6.70844	1.6041

HPLC traces of compound **11p**

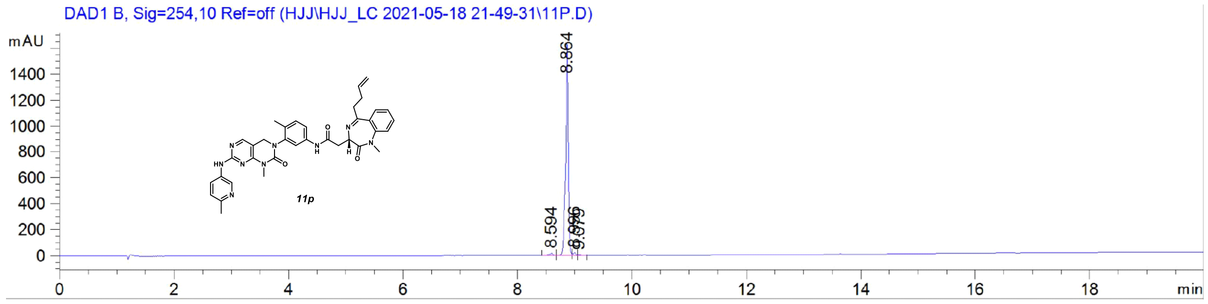

Peak #	RetTime [min]	Type	Width [min]	Area [mAU*s]	Height [mAU]	Area %
1	8.594	VV	0.0569	74.28070	17.72215	1.3824
2	8.864	VV	0.0463	5218.56689	1633.71033	97.1175
3	8.996	VV	0.0445	63.17330	20.21705	1.1757
4	9.079	VB	0.0539	17.43349	4.53171	0.3244

HPLC traces of compound **11q**

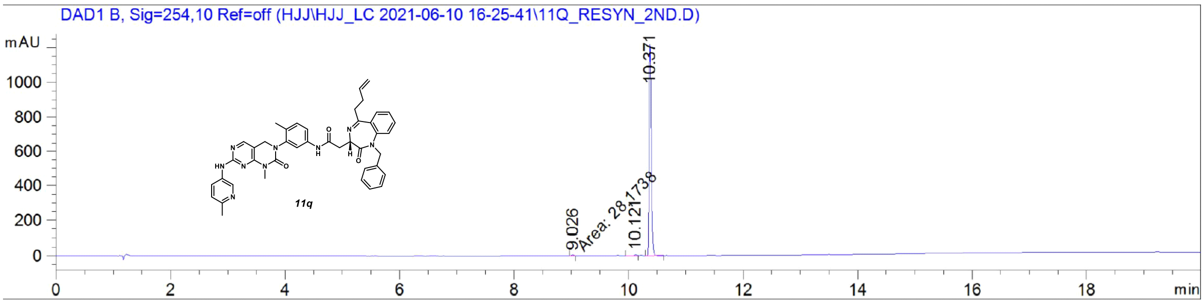

Peak #	RetTime [min]	Type	Width [min]	Area [mAU*s]	Height [mAU]	Area %
1	9.026	MM	0.0636	28.17377	7.38023	0.8668
2	10.121	VV	0.0432	21.17982	7.23171	0.6516
3	10.371	VB	0.0406	3201.14771	1218.76416	98.4817
